# Inferring the progression of multifocal liver cancer from spatial and temporal genomic heterogeneity

**DOI:** 10.18632/oncotarget.6558

**Published:** 2015-12-11

**Authors:** Jie-Yi Shi, Qingfeng Xing, Meng Duan, Zhi-Chao Wang, Liu-Xiao Yang, Ying-Jun Zhao, Xiao-Ying Wang, Yun Liu, Minghua Deng, Zhen-Bin Ding, Ai-Wu Ke, Jian Zhou, Jia Fan, Ya Cao, Jiping Wang, Ruibin Xi, Qiang Gao

**Affiliations:** ^1^ Liver Cancer Institute, Zhongshan Hospital, and Key Laboratory of Carcinogenesis and Cancer Invasion (Ministry of Education), Fudan University, Shanghai, P. R. China; ^2^ School of Mathematical Sciences and Center for Statistical Science, Peking University, Beijing, P. R. China; ^3^ Fudan University Shanghai Cancer Center and Institutes of Biomedical Sciences, Shanghai Medical College, Fudan University, Shangai, P. R. China; ^4^ Institute of Biomedical Sciences, Fudan University, Shanghai, P. R. China; ^5^ Cancer Research Institute, Xiangya School of Medicine, Central South University, Hunan, P. R. China; ^6^ Division of Surgical Oncology, Brigham and Women's Hospital, Harvard Medical School, Boston, MA, USA

**Keywords:** hepatocellular carcinoma, multifocal tumors, whole-exome sequencing, intratumor heterogeneity, FAT4

## Abstract

Multifocal tumors developed either as independent tumors or as intrahepatic metastases, are very common in primary liver cancer. However, their molecular pathogenesis remains elusive. Herein, a patient with synchronous two hepatocellular carcinoma (HCC, designated as HCC-A and HCC-B) and one intrahepatic cholangiocarcinoma (ICC), as well as two postoperative recurrent tumors, was enrolled. Multiregional whole-exome sequencing was applied to these tumors to delineate the clonality and heterogeneity. The three primary tumors showed almost no overlaps in mutations and copy number variations. Within each tumor, multiregional sequencing data showed varied intratumoral heterogeneity (21.6% in HCC-A, 20.4% in HCC-B, 53.2% in ICC). The mutational profile of two recurrent tumors showed obvious similarity with HCC-A (86.7% and 86.6% respectively), rather than others, indicating that they originated from HCC-A. The evolutionary history of the two recurrent tumors indicated that intrahepatic micro-metastasis could be an early event during HCC progression. Notably, *FAT4* was the only gene mutated in two primary HCCs and the recurrences. Mutation prevalence screen and functional experiments showed that FAT4, harboring somatic coding mutations in 26.7% of HCC, could potently inhibit growth and invasion of HCC cells. In HCC patients, both FAT4 expression and FAT4 mutational status significantly correlated with patient prognosis. Together, our findings suggest that spatial and temporal dissection of genomic alterations during the progression of multifocal liver cancer may help to elucidate the basis for its dismal prognosis. *FAT4* acts as a putative tumor suppressor that is frequently inactivated in human HCC.

## INTRODUCTION

Primary liver cancer (PLC), mainly hepatocellular carcinoma (HCC) and intrahepatic cholangiocarcinoma (ICC), is the second most deadly and fourth most common cancer worldwide [1]. Different from other cancers, multifocal tumors are very common in PLC [2, 3]. A recent national survey in Japan showed that half of the PLCs, especially HCC, were multiple lesions [4]. Chronic liver damage, such as that caused by chronic hepatitis and liver cirrhosis, is closely associated with the occurrence of both HCC and ICC [5]. As HCC and ICC have shared susceptibility factors, a particular type of multiple PLC is the coexistence of independent HCC and ICC in the liver, with an estimated incidence of 0.25% [6].

Multifocal PLCs, arising either synchronously or metachronously, may develop as independent tumors (i.e., multicentric occurrence) or as intrahepatic metastases (IMs) of the primary cancer [3, 7]. It is important to differentiate the two types of multifocal PLCs due to the significant differences in the pathogenesis, prognosis and treatment planning [8]. For example, patients with IMs could be treated by targeting the driver events of the primary tumor [9], while patients with multicentric tumors may be benefited from chemoprevention [10]. Clinicopathologic discriminators, such as tumor size, grade, nodule locations, vascular invasion and timing of recurrence, provide some crude values to clinical practice. Many studies have explored other genetic information, including HBV integration sites, *TP53* mutations and chromosomal aberrations, in differentiating IM from multiclonal PLCs but with limited success [2, 3]. Moreover, the molecular pathogenesis and genetic variability of multifocal PLC remains largely unknown, bringing a great challenge to effective molecularly targeted therapies in those patients. In this regard, next-generation sequencing was advocated to determine the genetic heterogeneity in different tumor sites or in multiple tumors to identify any driver event that may have functional, prognostic, and therapeutic implications [11].

Based on the above considerations, herein, we performed multiregional whole-exome sequencing (WES) on the synchronous multifocal PLCs and the metachronous recurrent tumors in one patient. Our results demonstrated that WES could delineate the clonality and heterogeneity of multifocal PLCs, as well as the evolution of the recurrent tumors. In addition, we identified *FAT4* as a putative tumor suppressor in HCC that was recurrently mutated, significantly down-regulated and had profound functional and prognostic importance.

## RESULTS

### Intertumor and intratumor heterogeneity among multifocal PLCs

**Figure 1:**
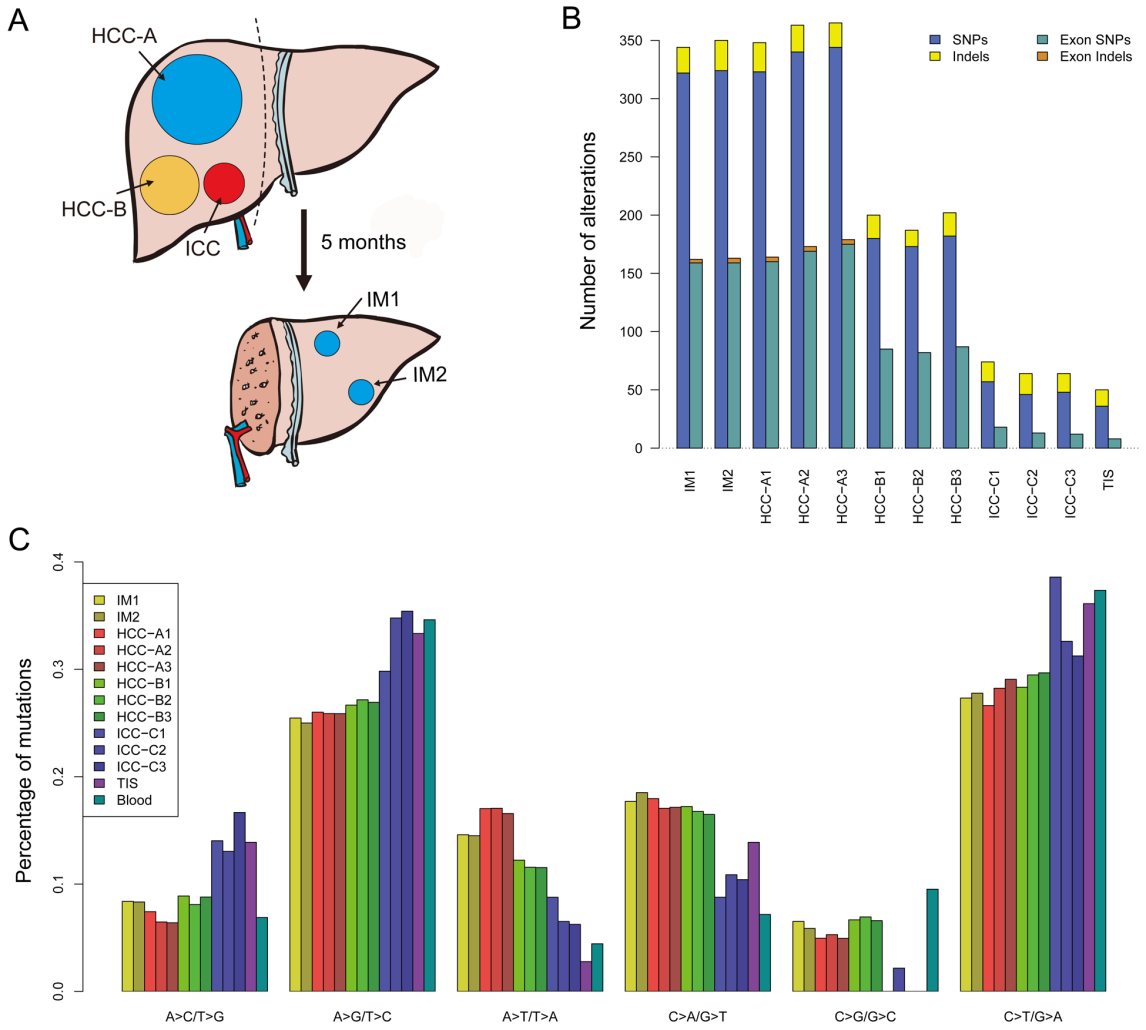
Selection of a male HBV case with multifocal liver cancer **A.** Schematic diagram of the three synchronous primary tumors and the two metachronous intrahepatic recurrent tumors. Dash line indicates liver resection. See Supplementary Figures 1-2 for the radiological and histological images. **B.** The total number of somatic mutations (SNVs and Indels) and exonic somatic mutations detected for each sample. **C.** Distribution of transition and transversion types for each sample.

**Figure 2:**
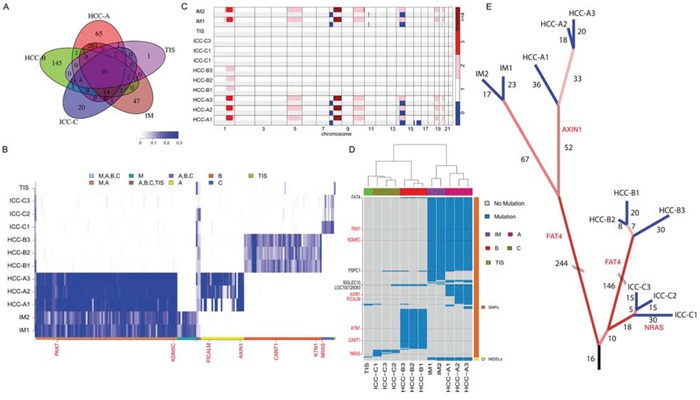
Intertumor genetic heterogeneity among the three primary tumors **A.** The Venn diagram of somatic mutations among the HCC-A, HCC-B, ICC and IM tumors. **B.** The VAF heatmap for the HCC-A, HCC-B, ICC, and IM tumors. VAFs of all non-synonymous SNVs with sequencing coverage above 10 across all samples were shown. The color keys correspond to mutations detected in different samples. **C.** The ASCNV of each sample. The two rows of each sample represent the copy numbers of the two alleles. **D.** The genes with nonsynonymous somatic mutations in the 12 different samples. Blue regions were mutations detected in a sample. The genes in red are known cancer-related genes. The clustering analysis was performed with the hierarchical clustering method. **E.** The phylogenetic tree constructed based on the somatic mutations detected with the in-house mutation detection method. The numbers indicate common somatic mutations shared by the tumors that were leafs of the branch. Mutations in the cirrhotic liver tissue provided a carcinogenic background, where three independent tumors occurred with profound intratumor heterogeneity.

WES was performed on synchronous multifocal PLC (2 HCC tumors, HCC-A and HCC-B; 1 ICC tumor; Figure 1A and Supplementary Figure 1) and two intrahepatic recurrent tumors (IM1 and IM2; Figure 1A and Supplementary Figure 2) of a patient for genetic comparisons (Supplementary Table 1 ). First, we combined the multiregional mutation data to evaluate intertumor heterogeneity among the three primary tumors. Significantly higher number of somatic single nucleotide variants (SNVs) was observed in HCC-A (*n* = 365) than that in HCC-B (*n* = 191) or ICC (*n* = 68) respectively (Figure 1B; Supplementary Tables 2–3). The numbers of indels were similar among them (33, 25 and 26 indels in HCC-A, HCC-B and ICC, respectively). The transition frequencies of HCC-A, HCC-B and ICC were ∼52%, ∼55% and >60%, respectively (Figure 1C). The majority of the somatic mutations, 82.1% (327/398), 67.1% (145/216) and 22.3% (21/94) in HCC-A, HCC-B and ICC respectively, were unique to each tumor (Figure 2A). The similar result was reported by Fujimoto et al. that no common somatic mutations were identified in the multicentric tumor pairs in HCC [12]. The heatmap of variant allele frequencies (VAF) of the SNVs revealed 3 clear blocks corresponding to SNVs only discovered in HCC-A, HCC-B and ICC, respectively (Figure 2B). Therefore, the results suggested that tumor cells at each primary tumor were vastly different and they may evolve under highly different carcinogenic processes. Furthermore, allelic specific copy number variation (ASCNV) analysis authenticated that the three tumors showed high degree of heterogeneity (Figure 2C and Supplementary Table 4). HCC-A harbored copy number gains on chromosomes 1q, 5p (containing *TERT,* a known driver in HCC [13]), 5q, 8q, 10p, 10q, 19p, 19q and 20q, and deletions on chromosomes 8p and 16q. Interestingly, for chromosome 14, the total copy numbers of HCC-A largely remained to be 2, but one copy of the 14q was actually duplicated and the other copy was deleted. In contrast, we only identified a copy number gain on chromosome 1q for HCC-B and no large CNVs for ICC.

The primary tumors showed various level of heterogeneity. Within individual tumors, 78.4% (312/398), 79.6% (172/216) and 46.8% (44/94) somatic mutations in HCC-A, HCC-B and ICC were common to their sub-regions, respectively (Supplementary Figure 3A). In HCC-A, HCC-A1 sub-region had distinct mutation profile comparing with HCC-A2 and HCC-A3 sub-regions (Supplementary Figure 3B). For most of the SNVs discovered in only HCC-A2 or HCC-A3, HCC-A1 had no reads supporting the alternative alleles (i.e. VAFs = 0). Considering that most SNVs at the three HCC-A sub-regions were common, there was probably only one major clone in HCC-A tumor at early stage and this founding clone subsequently evolved to at least 2 subclones (corresponding to HCC-A1 and HCC-A2/A3). HCC-B showed less level of heterogeneity than HCC-A and its VAF also showed no clear pattern (Supplementary Figure 3C). The VAF plot of ICC-C did not show any clear pattern (Supplementary Figure 3D), although ICC-C sub-regions had less common somatic mutations than HCC-A and HCC-B. This might be due to high level of normal contamination levels in ICC-C. In fact, ASCNV analysis showed that the normal contamination levels for ICC-C were >60%, for HCC-B were ∼50% and for HCC-A were <30%.

### Relationship of the primary and recurrent tumors

Recurrence of HCC after resection is attributed to either micro-metastasis within the remnant liver or occurrence of *de novo* primary lesions. Currently, there is lack of well-defined markers for differentiation of the two types of recurrence. Our WES data identified 344 and 350 somatic alterations for the recurrent tumors IM1 and IM2, respectively. The percentages of non-synonymous SNVs were 32.3% (104/322) and 31.8% (103/324) for IM1 and IM2, respectively. The majority of these somatic alterations (89.1% [327/367]) were shared by IM1 and IM2 (Supplementary Figure 4A), indicating that IM1 and IM2 may be derived from a common precursor. Interestingly, there were 86.1% (316/367), 15.2% (56/367) and 15.8% (58/367) of IM1/2 somatic alterations overlapping with HCC-A, HCC-B and ICC (Figure 2A) respectively, suggesting that IM1 and IM2 were most likely evolved from HCC-A. In fact, IM1 and IM2 genomes had almost zero VAFs for the most of HCC-B and ICC somatic SNVs (Figure 2B). ASCNV analysis also demonstrated that the genomes of the two intrahepatic recurrences were most similar to HCC-A genomes (Figure 2C).

On the other hand, 51 and 99 somatic SNVs were unique to IM1/2 and HCC-A, respectively. Among these, IM1 and IM2 had 46 and 47 SNVs not discovered in the HCC-A, while HCC-A1, HCC-A2, and HCC-A3 harbored 50, 64, and 65 somatic alterations not discovered in the IMs, respectively (Supplementary Figure 4A). Likewise, the VAF plot showed that the HCC-A had almost no supporting reads for the most of the IM-specific SNVs and *vice versa* (Figure 2B; Supplementary Figure 4B). In particular, the three HCC-A sub-regions had a shared mutation at the oncogene *AXIN1,* but the two IMs did not have this mutation and their VAFs were zero on this mutation (Figure 2B; Supplementary Figure 4C–4H). ASCNV analysis revealed that the copy number loss on chromosome 16 in HCC-A1 was absent in IM1 and IM2, and the small deletion on chromosome 11 in IM1 and IM2 were absent in HCC-A sub-regions (Figure 2C). Collectively, we concluded that IM1/2 and HCC-A were probably siblings that originated from the same founding clone (Figure 2D–2E), indicating that intrahepatic micro-metastasis could be an early event during HCC progression.

### Identification of cancer drivers common to the PLCs and IMs

Next, we tried to detect potential driver mutations in this special case. Figure 2D showed genes with non-synonymous somatic alterations of all samples. The genes in red are listed as census cancer genes in the Catalogue of Somatic Mutations in Cancer (COSMIC) database [14]. Among the cancer genes, only *AXIN1* is recurrently mutated in HCC according to COSMIC. The ICC-C1 harbored a somatic SNV at the oncogene *NRAS*, whose mutation frequency was around 5% in ICC. To further reveal the functional impact of these mutations, we performed functional annotation analysis using DAVID [15] (Supplementary Table 5 ). The top two enriched functional terms for HCC-A mutations were glycoprotein (P = 1.65 × 10^−5^) and EGF-like domain (P = 6.5 × 10^−5^; including *FAT4*, *HSPG2*, *etc*). The top enriched functional term for IM1/2 mutations was also EGF-like domain (P = 2.6 × 10^−5^) including *FAT4*. The top two enriched functional terms of HCC-B mutations were phosphoprotein (P = 3.7 × 10^−3^) and cell adhesion (P = 2.3 × 10^−2^; including *FAT4*, *CDH7*, etc). For ICC, DAVID did not report any significantly altered functional terms.

**Figure 3:**
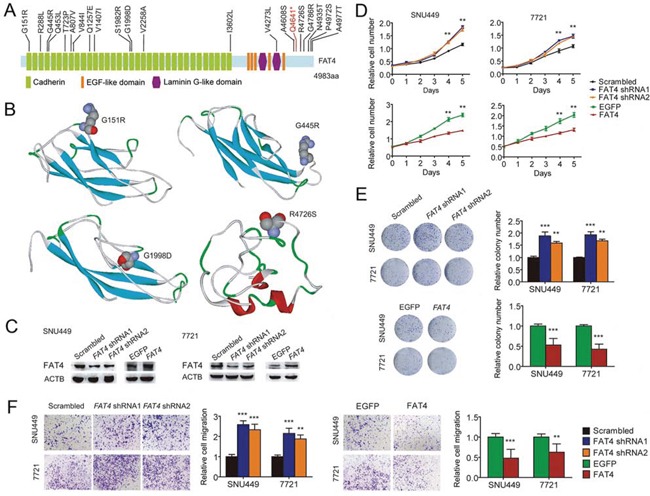
Identification of FAT4 as a tumor suppressor gene in HCC **A.** Schematics of protein alterations in FAT4 caused by somatic mutations. **B.** Structural modeling showing locations of the mutations G151R, G445R, G1998D, and R4672S. **C.** Protein blots showing FAT4 knockdown with shRNA and overexpression with TALE in indicated cells, compared with their respective controls. **D.** Growth curves showing accelerated growth with FAT4 knockdown and decelerated growth with FAT4 overexpression in indicated cells. **E.** Colony formation showing increased clones with FAT4 knockdown and decreased clones with FAT4 overexpression in indicated cells. **F.** Cell migration showing elevated migration with FAT4 knockdown and reduced migration with FAT4 overexpression in indicated cells. Experiments were performed in triplicate. *P < 0.05, **P < 0.01, ***P < 0.001.

Notably, the only gene that was commonly mutated among the two primary HCCs and two IMs was *FAT4*, which recurrently mutated in several other cancers and possibly acted as a tumor suppressor [16–19]. In our samples, three HCC-A sub-regions and IM1/2 had the c.G2530A mutation, while three HCC-B sub-regions had the c.A14804C mutation (all were Sanger validated) (Supplementary Figure 5). Although the putative effects of *FAT4* mutations in HCC has not been reported, our findings that the missense mutations of *FAT4* on c.G2530A and c.A14804C were located within an extracellular cadherin repeat and the cytoplasmic region respectively, implicated that *FAT4* may act as a tumor suppressor gene regulating cell contact and signal transduction, thus, the somatic inactivation of *FAT4* may be a key tumorigenic event in HCC. In addition, we performed immunohistochemical analyses on FAT4 protein levels in HCC-A, HCC-B and ICC. In Supplementary Figure 6, there were no significant differences of FAT4 expression levels in HCC-A and HCC-B (Score 1), and however, the expression level in ICC (Score 2) is higher than that in HCC. We further Sanger sequenced all protein-coding exons of *FAT4* gene in another 60 HBV-associated HCCs with paired normal controls as well as 25 HCC cell lines. In total, we identified 16 somatic nonsynonymous *FAT4* mutations in 16 of 60 HCCs (26.7%), comprising 15 missense variants and 1 nonsense variants, among which 2 were homozygous and 3 have been documented in COSMIC (Supplementary Table 6). In addition, 14 of 25 HCC cell lines had *FAT4* mutations, including 3 mutations that were not detected in the 60 HCC samples (Supplementary Table 7). *FAT4* mutations were located in the cadherin domains, the Laminin G like domain, or in the cytoplasmic region (Figure 3A). PolyPhen-2 analyses [20] revealed that 55% (11/20) of the missense mutations were predicted to adversely affect protein function. Structural modeling revealed that the mutations may undermine *FAT4* protein stability and thereby functions. For examples, G151R and G445R, located at the coil between two β-strands, were predicted to destabilize the protein. G1998, together with K1996 and N1997, constituted the β-turn that connected two β-strands, and thus G1998D substitution would severely break the stability or abrogated protein expression. In particular, R4726S substitution would definitely break the salt bridge formed between R4726 and E4720, disrupting interaction with the MPDZ domain (Figure 3B). Clinically, *FAT4* mutations were significantly enriched in patients with vascular invasion (P = 0.032) and advanced tumor stages (P = 0.088) (Supplementary Table 8 ), and correlated with increased recurrence (P = 0.041) (Supplementary Figure 7). An across database survey revealed that *FAT4* was mutated or deleted in various human cancers [21, 22] (Supplementary Figure 8).

### Functional significance and clinical implications of the FAT4 driver

Next, we aimed to uncover the biological role of *FAT4*, if any, in human HCC. We fist showed that all 25 human HCC cell lines constitutively expressed *FAT4* mRNA (Supplementary Table 7), whose expression level in HCC ranked as the top 9 among 35 cancer types in the CCLE database [23] (Supplementary Figure 9). Furthermore, we evaluated the impact of altered expression of *FAT4* in two HCC cell lines with wild type *FAT4*. *FAT4* knockdown by shRNA in SNU449 and SMMC-7721 cells significantly promoted in vitro cell proliferation, colony formation and migration compared to the controls (Figure 3C–3F). Over-expression of *FAT4* by Transcription activator like effectors (TALE) in SNU449 and SMMC-7721 cells showed markedly attenuated cell proliferation, as well as colony formation and migration *in vitro* as compared with controls (Figure 3C–3F). The results indicated that *FAT4* may function as a negative regulator of HCC cell growth and motility.

**Figure 4:**
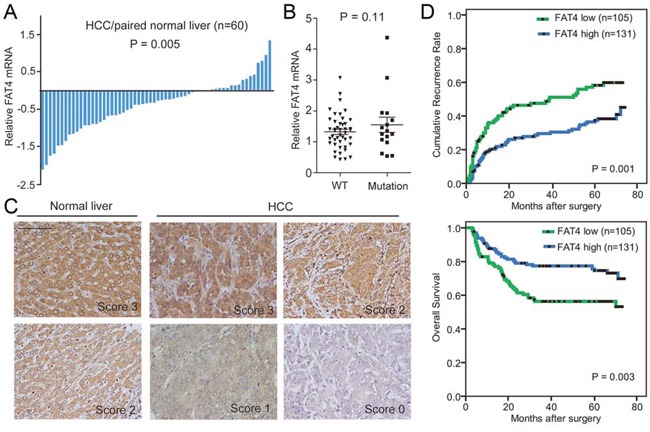
FAT4 was down-regulated in HCC and correlated with clinical outcome **A.** Bar plot showing *FAT4* mRNA expression in paired HCC and normal liver tissues (*n* = 60). **B.** Quantitative RT-PCR showing the difference in *FAT4* mRNA level between tumors with FAT4 mutations (*n* = 16) and those with WT (*n* = 44). **C.** Representative immunostaining images of FAT4 protein in HCC and normal liver tissues. Scale bar, 100 μm. **D.** Kaplan-Meier curves showing increased recurrence and dismal survival in HCC patients with low versus high FAT4 expression (log-rank test).

We then investigated clinical significance of *FAT4* expression in HCC patients. Among the 60 paired HCC and normal tissue samples, 70% (42/60) of the tumor showed down-regulation of *FAT4* mRNA by Qualitative RT-PCR analysis (P = 0.005) (Figure 4A). However, there were no obvious differences in *FAT4* mRNA levels between tumors with *FAT4* mutations (*n* = 16) and those with WT (*n* = 44) (Figure 4B). Available public data showed that *FAT4* expression was universally down-regulated in various human cancers (Supplementary Figure 10). Interestingly, our multiregional mRNA expression data showed that 18.2% (4/22) of HCC cases showed intratumor heterogeneous expression of FAT4 (i.e., co-existence of up- and down-regulation), while the remaining cases displayed homogeneous up- or down-regulation within each tumor (Supplementary Figure 11). At the protein level, immunostaining of FAT4 on tissue microarray containing a consecutive cohort of 236 HCCs was conducted. As shown in Figure 4C, FAT4 protein was presented in both cell membrane and cytoplasm. It was found that 44.5% (105/236) of HCCs showed low expression of FAT4 (scores 0 and 1). FAT4-low expression associated with larger tumor size (*p* = 0.041) and advanced tumor stage (P = 0.032) (Supplementary Table 9). In addition, patients with FAT4-low expression had significantly increased recurrence (P = 0.001) and poorer survival (P = 0.003) than those with FAT4-high expression (scores 2 and 3) (Figure 4D). The 5-year recurrence-free and overall survival rates were 39.8% and 54.0% for FAT4-low patients, and 59.2% and 71.1% for FAT4-high patients, respectively. On multivariable analyses, low FAT4 expression was confirmed as an independent prognostic factor for unfavorable recurrence (hazard ratio [HR], 1.98; 95% confidence interval [CI], 1.34–2.93; P= 0.001) and survival (HR, 2.44; 95% CI, 1.52–3.93; P < 0.001) (Table 1). These data indicated that, in parallel with the *in vitro* function and mutation prevalence, *FAT4* deficiency may lead to uncontrolled tumor progression and was detrimental to clinical outcome of HCC.

**Table 1:** Univariate and multivariate analyses of factors associated with time to recurrence and overall survival

Variables	Recurrence				Overall survival			
	Univariable		Multivariable		Univariable		Multivariable	
	*P*	HR	95% CI	*P*	*P*	*HR*	95% CI	*P*
Age, years (>51 vs.≤51 )	0.684			NA	0.917			NA
Gender (male vs. female)	0.896			NA	0.315			NA
Hepatitis history (yes vs. no)	0.286			NA	0.156			NA
α-Fetoprotein (ng/ml) (>20 vs. ≤20)	0.021	1.64	1.08-2.47	0.019	0.001	2.07	1.24-3.44	0.005
γ-Glutamyl transferase (U/l) (>54 vs. ≤54)	0.068			NA	0.043			NS
Liver cirrhosis yes vs. no)	0.638			NA	0.011	0.39	0.22-0.71	0.002
Tumor differentiation (poor vs. well)	0.055			NA	<0.0001	2.12	1.35-3.34	0.001
Tumor size (cm) (>5 vs. ≤5)	<0.0001			NS	<0.0001			NS
Tumor multiplicity (multiple vs. single )	0.073			NA	0.445			NA
Tumor encapsulation (none vs. complete)	0.083			NA	0.035			NS
Vascular invasion (yes vs. no)	<0.0001			NS	<0.0001			NS
TNM stage (III vs. II vs. I)	<0.0001	1.47	1.12-1.94	0.006	<0.0001	1.55	1.14-2.12	0.006
BCLC stage (B-C vs. 0-A)	<0.0001	2.22	1.30-3.77	0.003	<0.0001	3.92	1.80-8.52	0.001
FAT4 (Low vs. High)	0.001	1.98	1.34-2.93	0.001	0.003	2.44	1.52-3.93	<0.0001

Univariate analysis was calculated by the Kaplan—Meier method (log-rank test).

Multivariate analysis was done using the Cox multivariate proportional hazard regression model with stepwise manner (forward, likelihood ratio). Patients were classified into 2 groups according to the levels of FAT4.

Abbreviations: TNM, tumor-nodes-metastases; HR, hazard ratio; CI, confidential interval; NA, not adopted; NS, not significant.

## DISCUSSION

Patients with PLC frequently have multiple anatomically separate tumors. Whether individual tumors are derived from a common precancerous or cancerous ancestor or independently from liver progenitor cells is a major issue [2, 3, 7]. Herein, in a special case with multifocal PLC, we demonstrated that in this case individual primary tumors probably developed as multicentric occurrence, and we also deduced the evolution of the postoperative recurrent tumors by comprehensive genomic profiling. Meanwhile, we depicted the intertumor and intratumor heterogeneity of individual tumors, and identified *FAT4* as a recurrently mutated tumor suppressor gene in HCC.

The 2 primary HCCs and the ICC showed considerably different genomic landscapes with significantly different number of SNVs, distinctive SNV heatmap patterns, limited number of overlapping somatic mutations, and the unique ASCNV among them. The results indicated that the three tumors may evolve through an accumulation of highly different sets of genetic alterations. This study and others [24, 25] collectively indicated that the mutational profile was a valuable alternative to characterize the clonality and molecular pathogenesis of multiple PLC. Furthermore, intratumor heterogeneity may lead to regional biases and underestimation of a tumor's mutational landscape. Our multiregional sequencing data showed varied intratumor heterogeneity in each tumor, resulted in multiple regionally separated phenotypes. In addition, it has been proposed that intratumor heterogeneity increased with the number of biopsies and generally with no evidence of saturation [26]. In this study, only three separated regions were biopsied and sequenced for each tumor, possibly giving an underestimation of intratumor heterogeneity in this case.

The relationship between primary tumor and recurrent tumor has been an important clinical issue. We showed that somatic mutations and copy number alterations of the recurrent tumors IM1/2 showed high concordance with HCC-A rather than HCC-B and ICC. Therefore, IM1/2 were most likely evolved from intrahepatic metastases of HCC-A. This assumption was also in line with our previous report that gene expression signature of primary HCC was very similar to that of their corresponding metastases [27]. Interestingly, we found that HCC-A had the highest scale of intratumor heterogeneity and the highest proportion of non-synonymous SNVs among the primary tumors. It is postulated that the intratumor heterogeneity positively correlated with risk of recurrence [28] and prevalence of non-synonymous SNVs may covey a positive Darwinian-like somatic evolution driving tumor progression [29]. As such, HCC-A tumor may harbor profound survival and metastatic advantages over HCC-B and ICC tumors. Meanwhile, the spatial and longitudinal genomic information of HCC-A sub-regions and IM1/2 provided a typical example demonstrating the clonal evolution model in HCC.

Notably, we identified *FAT4* as a potential driver in hepatocarcinogenesis and tumor progression. *FAT4*, one of the human homologue of Drosophila Fat, encodes a cadherin-related protein regulating planar cell polarity and Hippo signaling [30]. Other members of FAT gene family, i.e., *FAT1*, *FAT2* and *FAT3*, have been extensively characterized in various cancers recently [16, 31, 32]. Our genetic, functional and clinical data clearly indicated that *FAT4* was a tumor suppressor in HCC. Consistently, *FAT4* gene polymorphisms were reported to be associated with the risks of esophageal cancer and male lung adenoma [33, 34]. Recurrent mutations of *FAT4* were detected in several human cancers, such as melanoma and colorectal cancer [16, 17]. The potential tumor suppressive role of *FAT4* was reported in breast and gastric cancers [18, 19]. Epigenetic mechanism, i.e. promoter hypermethylation, was involved in *FAT4* dysregulation in human breast and lung cancers [18, 35]. In addition, for the first time, we found that 18.2% of HCC showed intratumor heterogeneous expression of *FAT4*, similar to a recent study reporting intratumor heterogeneous mutation of *TP53* and *CTNNB1* in 22% of HCC [36].

The major limitation of this analysis is that it is only based on one patient. However, it is postulated that “less is more” and such “N-of-1 study” had the power of thorough analysis of one individual to identify and characterize rare disease subtypes [37]. In this study, the selected patient was in a rare clinical condition—he had 3 primary independent tumors and 2 recurrent tumors. This extreme case provided us a unique opportunity to uncover the characteristics of tumor initiation and evolution. We found that even under homogeneous genetic and environmental background, different tumors could develop by accumulating considerably different genetic alterations. Comparison of genetic alterations in the primary and recurrent tumors allowed us to identify the putative tumor suppressor *FAT4* in HCC.

In summary, our findings authenticated that multifocal synchronous PLCs, if they were multicentric, may develop through an accumulation of highly different sets of genetic alterations. In contrast, intrahepatic metachronous recurrent tumors, if they originated from micro-metastases rather than *de novo* carcinogenesis, may share similar genetic profiles to the primary tumor. The mutational landscape could provide an avenue to characterize the clonality of mutifocal PLC, and thus treatment planning. Undoubtedly, intertumor and intratumor heterogeneity could bring a great challenge to targeted therapy and risk stratification of PLC, especially occurring as multifocal nodules.

## MATERIALS AND METHODS

### Patients and sample collection

We selected a 65-year-old male HBV patient with synchronous multifocal PLC (2 HCC tumors, HCC-A and HCC-B; 1 ICC tumor) who received right tri-segmentectomy (Figure 1A and Supplementary Figure 1). Three biopsies were obtained from separate regions of each tumor. Peritumor noncancerous cirrhotic liver tissue (TIS) and blood sample were also obtained. Five months after operation, two intrahepatic recurrent tumors (IM1 and IM2) were detected by CT scan and were biopsied (Figure 1A and Supplementary Figure 2). WES, followed by Sanger validation (a validation rate of 90.4%), was performed on all those samples for genetic comparisons (Supplementary Table 1). WES data have been submitted to the European Genome-phenome Archive (https://www.ebi.ac.uk) with the accession number PRJEB8083. Details of other HCC samples used for mutation prevalence screen, qRT-PCR and immunohistochemistry can be found in the Supplementary Materials and Methods. Our study was conducted after obtaining written consents from patients and according to ethical approval from Zhongshan Hospital Research Ethics Committee.

### Other materials and methods

Details for WES, Sanger validation, tissue microarray construction, histologic examination, Western blot, qRT-PCR, shRNA, TALE, and *in vitro* functional assays including cell proliferation, colony formation and Transwell assays, were described in the Supplementary Materials and Methods.

### Statistical analysis

Statistical analysis was done with SPSS 19.0 (SPSS, IBM) and R software. Data were presented as the means ± standard deviation (SD). The Fisher's exact test, Students' *t* test and Mann–Whitney *U* test were used as appropriate. Kaplan-Meier curves (log-rank test) were used to describe recurrence and survival. Univariable analyses and multivariable analyses were based on the Cox proportional hazards regression model. Two-tailed P value < 0.05 indicates statistical significance.

## SUPPLEMENTARY FIGURES AND TABLES




























