# Supplementary material for: Inferring the progression of multifocal liver cancer from spatial and temporal genomic heterogeneity
Source: Oncotarget. 2015 Dec 11;7(3):2867–77. doi: 10.18632/oncotarget.6558 (PMC4823077; doi:10.18632/oncotarget.6558)
Supplement: Supplementary file 1 [file oncotarget-07-2867-s001.pdf]

SUPPLEMENTARY MATERIALS AND METHODS

Patients and sample collection

We selected a 65-year-old male patient with multifocal primary liver cancer (2 HCC and 1 ICC) who received right tri-segmentectomy and suffered from postoperative intrahepatic recurrence (Figure 1A; Supplementary Figures 1–2). The patient was HBV+HCV-, with no alcohol consumption or family cancer history, and no anticancer treatments were given before operation. Pre-operative alpha-fetoprotein was 1,006.0 ng/ml, HBV DNA was 1.3×10^4 copy/ml and liver function was normal. WES was performed on all the tumors, peritumor liver and blood samples.

For mutation prevalence screen, fresh frozen HCC and matched non-cancerous liver samples were obtained from 60 patients treated between Jan 1 and Mar 31, 2009 (Supplementary Table 8). The median duration of follow-up of this cohort was 51.0 months (range, 1.0–54.0 months; SD, 15.9 months). Among these patients, 22 had multiregional spatially separated tissue samples (6 areas for each tumor) for evaluating intratumor variation in FAT4 mRNA levels. A total of 25 liver cancer cell lines were also used for mutation screen (Supplementary Table 7). For tissue microarray construction and immunohistochemical analysis, archived paraffin-embedded HCC and paired non-cancerous liver tissues were obtained from 236 patients treated between Jan 2007 and Dec 2007 (Supplementary Table 9), as previously described [1]. The median follow-up of this cohort was 60.0 months (range, 3.0–74.0 months; SD, 25.5 months). All those patients received potentially curative resection for primary HCC at Zhongshan Hospital of Fudan University. The clinical data collection and postoperative surveillance according to a uniform guideline were described previously [1, 2]. Time to recurrence (TTR) and overall survival (OS) were the main end points as described previously [1, 2]. The study was approved by the Research Ethics Committee of Zhongshan Hospital, with written informed consent from each participant.

Exome capture and sequencing

Tumor tissues were analyzed by frozen section to assess neoplastic cellularity (Supplementary Figures 1C and 2B). Normal liver tissue was also analyzed by frozen section to confirm the presence of only non-neoplastic tissue. DNA isolation has been described previously [3], using DNeasy Blood and Tissue Kit (Qiagen). Whole-exome sequencing of DNA samples from the patient was performed essentially as previously described [3, 4]. DNA libraries were prepared according to the Illumina library generation protocol. Each sample was tagged with a custom-designed unique 4-base-long index within the Illumina adaptor, pooled, captured by Agilent SureSelect Human All Exon V4 kits and sequenced using Illumina HiSeq 2000 system. Paired-end sequencing (2 × 101 bp) was carried out using standard Illumina protocols. All samples were sequenced to an average depth of 98.07 × , ranging from 78.05× (96.99% of targeted bases covered at ≥ 10 ×) to 126.37× (98.03% of targeted bases covered at ≥ 10 ×) (Supplementary Table 1).

Mutation and indel detection

All short reads were aligned to the NCBI human reference genome hg19 (GRCh37/hg19) using Burrows-Wheeler Aligner (BWA) with default parameters [5]. SAMtools was used to remove non-uniquely mapped reads and possible PCR duplicates. SNPs and Indels were then called by the Genome Analysis Tool kit (GATK) HaplotypeCaller after local Indel realignment and base quality score recalibration [6]. The initial set S_0 of somatic SNPs/Indels was defined as the SNPs/Indels that were detected by GATK in the tumor or the TIS Exome-seq data but not in the blood data. This set S_0 may still contain germline mutations since germline mutations may be detected in tumor/cirrhotic liver genomes but not in blood genome. To remove these mutations, for each mutation m in in S_0, we calculated the local mapping error rate r (100bp neighborhood) of this mutation for the blood data and performed a binomial test to test if there is a mutation for blood with r as the success probability of the binomial distribution. If the P-value of this binomial test was less than 0.05, we viewed this mutation as a possible germline mutation and removed it from S_0. We also removed mutations in S_0 at which the read depth of blood was less than 10, because the mutation detection power of blood is insufficient at these positions and these mutations may still be germline mutations even though the corresponding P-value for blood is greater than 0.05.

We further removed any candidate somatic SNP in S_0 who had any Indel in its 100bp neighborhood since these were likely to be false positives. Similarly, a candidate Indel in S_0 was removed if there was another Indel in its 100bp neighborhood or there were at least 2 short reads supporting other types of Indels at this position. At last, we applied a quality filtering criterion to filter the low quality mutations. If we directly use a threshold to filter, a common mutation may be filtered in one sample but not filtered in another sample, thus leading us to a biased result for our phylogentic analysis. Therefore, we instead used the following procedure to filter low quality calls. If a mutation was detected in multiple samples, we kept it in our final call set only if the maximum quality score of this mutation across all the detected samples

was greater than a threshold. The threshold was chosen as 80 for SNPs and 100 for Indels. After these filtering steps, we obtain a somatic mutation call set S (which is a subset of S_0).

Since the mean read-depth and the level of normal DNA contamination varies across different samples, common mutations may be detected in one sample but not in another sample due to the detection power difference. We therefore developed an in-house method to detect somatic mutations more sensitively based on the somatic mutation call set S. Specifically, for each mutation m in S, suppose that its genomic location is s. We calculate the proportion of the mismatches (the mismatches that are the same as the mutation m are not counted) in a 100bp neighborhood of s for each sample. Then, for a sample in which the mutation m was not detected, we performed a Binomial test to test if there was such a mutation at the genomic positions. If the P-value of this test was less than 1×10^{-4}, we set this mutation as a candidate mutation for the sample. With this method, we were able to detect more mutations and there were more overlaps between mutation call sets of different samples, especially for the HCC-B tumors. We called this set of mutations S_e (the call set S was a subset of S_e).

Allelic specific copy number variation (ASCNV) analysis

In this section, we refer tumor genome to tumor or cirrhotic liver genome. The Allelic Specific Copy Number Variation (ASCNV) analysis was based on the heterozygous SNPs detected with the blood data. For each autosomal heterozygous SNP s in blood ($s = 1, \cdots, S$), let N_{As} and N_{Bs} be the number of short reads of blood supporting allele A_s and allele B_s, respectively. Similarly, define T_{As} and T_{Bs}. be the corresponding number of short reads for a tumor data. Assume that C_{As} and C_{Bs} be the copy number of the allele A_s and B_s in the tumor genome. Let Φ be the ploidy number of the tumor/cirrhotic liver genome and r be the proportion of tumor or cirrhotic liver DNAs in the sequenced library ($1 - r$ is the level of normal DNA contamination). We assume that the copy numbers of allele A and B for blood is 1. This assumption should hold for almost all germline heterozygous SNPs. Let R_N and R_T be the mean read depth of blood (normal) and tumor genome. Then, we have the mean of the ratio $\frac{T_a/R_T}{N_a/R_N}$ should equal to $\mu_a = \frac{rC_a + 1 - r}{r\Phi/2 + (1-r)}$ $(a = As, Bs)$. The ratio $Y_a = \frac{T_a/R_T}{N_a/R_N}$ can be viewed as following a normal distribution $N(\mu_a, \sigma^2_{C_a})$, where we assume that the variance $\sigma^2_{C_a}$ only depends on the allelic copy number C_a $(a = As, Bs)$. Then, the joint likelihood of $Y_s = (Y_{As}, Y_{Bs})$ can be written as $\phi(y_{A_s}|\mu_{As}, \sigma_{CAs})\phi(y_{Bs}|\mu_{Bs}, \sigma_{CBs})$, where $\phi(x|\mu, \sigma^2) = (2\pi\sigma^2)^{-1/2} e^{-(x-\mu)^2/(2\sigma^2)}$ is the density function of the normal distribution $N(\mu, \sigma^2)$.

To estimate the parameters in this model, we first performed segmentation with the BIC-seq algorithm (37) based on the read-depth data at the heterozygous SNPs. Suppose that we obtain K segments $I_1, \cdots, I_K$. We assume that the copy numbers of the two alleles at different positions remain constant in a segment. However, given heterozygous SNPs s in a segment, we do not know what the haplotype looks like. Thus, we have the new likelihood

$$\prod_{k=1}^{k} \prod_{s \in I_k} \frac{1}{2}\Big[\phi(y_{As}|\mu_{k1}, \sigma^2_{c_{k1}})\phi(y_{Bs}|\mu_{k2}, \sigma^2_{c_{k2}}) + \phi(y_{Bs}|\mu_{k1}, \sigma^2_{c_{k1}})\phi(y_{As}|\mu_{k2}, \sigma^2_{c_{k2}})\Big],$$

where C_{kj} is the copy number of the jth haplotyp at the kth segment and $\mu_{kj} = \frac{rC_{kj} + 1 - r}{r\Phi/2 + (1-r)}$. We use Bayesian method to estimate the parameters. The prior of the purity parameter r is chosen as the uniform distribution and the prior of the parameter $\Phi' = \frac{1}{r\Phi/2 + 1 - r} > 0$ is chosen as a truncated normal distribution (truncation at 0). The priors for σ^2_{Ckj} $(j = 1, 2)$ are chosen as inverse gamma distributions. With these priors, we can use Gibbs sampler to estimate the parameters r, Φ and the unknown copy numbers C_{kj}. For the ICC genomes and cirrhotic liver genome, BIC-seq segmentation results showed that there was almost no copy number change and we fixed their ploidy number Φ as 2 and used VAF to estimate the purity parameter r (r is estimated as the mean VAF of nonsynonymous SNPs). The copy numbers were then estimated with this Bayesian model.

Altered functional category analysis

We used DAVID [7] to analyze the enriched functional categories of somatic mutations in the tumor genomes. The nonsynonymous somatic mutations in the call set S of each tumor (HCC-A, HCC-B and ICC-C) were used for this analysis. Before performing the enrichment analysis, we first compared the nonsynonymous mutations with the COSMIC database. We only kept a gene for the enrichment analysis if it had a nonsynonymous mutation and there were more than 2 samples in the COSMIC database with a mutation at this gene. The category we compared with is the SP_PIR_KEYWORDS as provided by DAVID.

Statistical analysis of VAFs and the hierarchical tree construction

The confidence regions of mean VAFs of the IMs against HCC-A tumors were obtained as follows. For each class of mutations (e.g. IM1 only mutations or IM1 and HCC-A1 mutations), we calculated the mean $\hat{\mu}$ and variance-covariance $\hat{\Sigma}$ of VAFs of these

mutations. The 99% confidence region was set as $\left\{ z \in R^2 | (z - \hat{\mu})^t \hat{\sum}^{-1} (z - \hat{\mu}) \leq c \right\}$, where c was the constant such that $P(\sqrt{X^2 + Y^2} \leq c) = 0.99$ with (X, Y) having a standard normal distribution. Here, we only used mutations whose read depths across the samples were at least 20. The hierarchical tree in Figure 2E was constructed by the function hclust in R.

Verification of mutations using sanger sequencing

To evaluate the performance of the variant calling algorithm, 218 of the somatic SNVs identified in whole-exome sequencing were selected for validation using Sanger sequencing (primer sets listed in Supplementary Table 10), and we confirmed 197 (90.4%) somatic SNVs. The coding exons *FAT4* gene were further screened using primer sets listed in Supplementary Table 11 in the 60 HCC samples and matched controls, as well as 25 HCC cell lines. Sanger sequencing primers were designed using Primer3 software (http://frodo.wi.mit.edu/). All mutations identified in tumors were confirmed by independent PCR and Sanger sequencing in the specific tumors and their paired normal tissue to determine their somatic nature.

Quantitative reverse transcription PCR (qRT-PCR)

Total RNA isolation, purification and reverse transcription were conducted as previously described [2]. Real-time RT-PCR was performed using a SYBR-Green PCR master mix (Invitrogen) and ABI PRISM 7500 Sequence Detection System (Applied Biosystems), according to the manufacturers' instructions. The primers used were: *FAT4* 5′ AGGCACAAATGGACAGGTTC 3′ (forward) and 5′ AAGGTTTAGCGACAGTGATGG 3′ (reverse); *TBP* 5′ CTCTCACAACTGCACCCTTG 3′ (forward) and 5′ ATCCCAGAACTCTCCGAAGC 3′ (reverse). All samples were performed in triplicate and TBP values were used to normalize gene expression using $2^{-\Delta CT}$ method.

Tissue microarray and immunohistochemistry

Tissue microarrays were constructed as previously described [1]. Core samples were obtained from representative regions from each tumor on hematoxylin and eosin staining. Duplicate 1-mm cores were taken from different areas of the same tissue block for each case (tumor tissue and matched noncancerous liver tissue, i.e., a total of four cores). Tissue microarrays were constructed using an arraying machine (Beecher Instruments).

Immunohistochemistry for FAT4 was performed as previously described [2, 8]. Briefly, 4-μm sections were deparaffinized and subjected to antigen retrieval (citrate buffer, pH = 6.0). Sections were then incubated for 30 min with rabbit polyclonal antibody to FAT4 (1:150 dilution, Cat# NBP1–78381, Novus Biologicals). Reaction products were visualized with 3, 3′-diaminobenzidine tetrahydrochloride and counterstained with hematoxylin. FAT4 immunostaining intensities were semiquantitatively scored as: 0, negative; 1, weak; 2, moderate; 3, strong by two observers independently, and comparisons were made between tumor/normal pairs.

Cell culture and transfection

The human HCC cell lines, SNU-449 and SMMC-7721, were maintained in DMEM (Invitrogen) supplemented with 10% FBS and penicillin/streptomycin. Cell line authentication was done essentially as we previously described [9].

For shRNA mediated knockdown of *FAT4*, six19-nucleotide sequences targeting gene *FAT4* (Supplementary Table 12) were selected using the BLOCK-It siRNA design program (Invitrogen). A control sequence (5′-TTCTCCGAACGTGTCACGT-3′) was used. Then the fragments were subcloned into pLVTH (Addgene) vector using the restriction sites MluI-ClaI. Lentiviral stocks were prepared by co-transfecting HEK-293T cells with shRNA and standard virus packaging systems as we previously described [2, 10] . Target cells were infected with filtered lenti-virus plus 6 μg/mL polybrene (Sigma-Aldrich) to generate stable cell lines. Two sequences (shRNA1 and shRNA2) with the highest efficiency of knockdown were selected for subsequent experiments.

For over-expression of *FAT4*, artificial TALE transcription activators TALE-VP64 Vectors based on pXanthoTMV.basic.puro using for *FAT4* overexpression were purchased (Taileng Biotech. Inc., Shanghai, China). The pXanthoTMV.basic.puro vector is complementary to the TAL Effector Kit 2.0 (Addgene), from which the RVD modules were selected to assemble the TALE-VP64 by a Golden-Gate method. The expression of TALE-VP64 activators are driven by a ubiquitous CAG promoter while a puromycin resistant gene is coexpressed by a IRES element (Internal Ribosome Entry Site) under the same promoter. Five *FAT4* TALE-VP64 activators were designed targeting around the transcription start site (TSS) of *FAT4* (Supplementary Table 13). A TALE-VP64 activator was synthesized as the control vector with binding sites on EGFP. The EGFP-TALE-VP64 has a RVDs array as NG-HD-NI-HD-HD-NN-NN-NN-NN-NG- NN-NN-NG-NN-HD-HD-HD-NI-NG, targeting at TTCACCGGGGTGGTGCCCAT. Each of designed TALE-VP64 vectors was transfected into SNU-449 and SMMC-7721 cells in 6 Well Plate with lipofectamine 2000. Dozens of clones appeared after 4 weeks of

selection by 2ug/ml of puromycin. One vector that resulted in the highest level of *FAT4* expression was selected for subsequent experiments.

Proliferation, colony formation and migration assays

For proliferation assay, HCC cells were seeded in 96-well plates seeded at 250–500 cells/well according to the character of each cell line. Cell viability and growth was determined by Cell-Counting Kit (CCK)-8 (Dojindo), reading absorbance at 450 nm according to the manufacturer's instructions and as previously described [2, 11].

For colony formation assay, cells were seeded into 6-well plates at a concentration of 300–600 cells/well and cultured at 37°C for 12–14 days according to the character of each cell line. Then, the cells were fixed with 100% methanol and stained with 0.1% crystal violet. Megascopic cell colonies were counted by Image-Pro Plus 5.0 (Media Cybernetics).

In vitro migration assay was performed in chamber of 8-µm Transwell inserts (BD Falcon) essentially as described [2]. 5 × 10 [4] cells were placed into the top chamber of each insert. According to the character of the used cell lines, cells that migrated were fixed and stained in dye solution containing 0.1% crystal violet and 20% methanol after 18–28 hours of incubation at 37°C. The number of cells that had migrated was counted using an IX71 inverted microscope (Olympus Corp.).

All results are representative of three independent experiments performed in triplicate.

Western blot

Protein extracts were processed for protein blotting using established methods as described [2]. The primary antibodies used were mouse anti-human FAT4 (1:500 dilution, Cat# ab130076, Abcam) pAb. Different amount of protein samples were loaded in the context of *FAT4* knockdown (30 µg/well) or over-expression (20 µg/well). Horseradish peroxidase (HRP)-conjugated secondary antibodies and enhanced chemiluminescence (ECL) reagents were from Amersham. Beta-actin (1:2000 dilution, Sigma-Aldrich) was used as the loading control.

Protein structural analysis

Structure models for all wild type domains of *FAT4* were predicted by I-TASSER server (http://zhanglab.ccmb.med.umich.edu/I-TASSER/) with default parameters. The model with the best score for each domain was selected as the wild type structural model. Structural models for mutants were constructed by using the corresponding protocol of the Discovery Studio 2.5. Secondary structure intrinsic disordered region prediction was performed by using the PSIPRED and the DISOPRED3 server (http://bioinf.cs.ucl.ac.uk/psipred/) with default parameters, respectively. Differences in Folding free energy between the wild type and the mutants were calculated by FoldX software (http://foldx.crg.es), helping to judge how the mutants can influence the protein structure. Figures were prepared using PyMOL (http://www.pymol.org).

REFERENCES

1. Chen Z, Lu X, Wang Z, Jin G, Wang Q, Chen D, et al. Co-expression of PKM2 and TRIM35 predicts survival and recurrence in hepatocellular carcinoma. Oncotarget 2015;6:2538–2548.
2. Gao Q, Zhao YJ, Wang XY, Qiu SJ, Shi YH, Sun J, et al. CXCR6 upregulation contributes to a proinflammatory tumor microenvironment that drives metastasis and poor patient outcomes in hepatocellular carcinoma. Cancer Res 2012;72:3546–3556.
3. Gao Q, Zhao YJ, Wang XY, Guo WJ, Gao S, Wei L, et al. Activating Mutations in PTPN3 Promote Cholangiocarcinoma Cell Proliferation and Migration and Are Associated With Tumor Recurrence in Patients. Gastroenterology 2014;146:1397–1407.
4. Jia D, Dong R, Jing Y, Xu D, Wang Q, Chen L, et al. Exome sequencing of hepatoblastoma reveals novel mutations and cancer genes in the Wnt pathway and ubiquitin ligase complex. Hepatology 2014;60:1686–1696.
5. Li H and Durbin R. Fast and accurate short read alignment with Burrows-Wheeler transform. Bioinformatics 2009;25:1754–1760.
6. DePristo MA, Banks E, Poplin R, Garimella KV, Maguire JR, Hartl C, et al. A framework for variation discovery and genotyping using next-generation DNA sequencing data. Nat Genet 2011;43:491–498.
7. Huang da W, Sherman BT and Lempicki RA. Systematic and integrative analysis of large gene lists using DAVID bioinformatics resources. Nat Protoc 2009;4:44–57.
8. Gao Q, Wang XY, Qiu SJ, Yamato I, Sho M, Nakajima Y, et al. Overexpression of PD-L1 significantly associates with tumor aggressiveness and postoperative recurrence in human hepatocellular carcinoma. Clin Cancer Res 2009;15:971–979.
9. Zhou ZJ, Dai Z, Zhou SL, Hu ZQ, Chen Q, Zhao YM, et al. HNRNPAB induces epithelial-mesenchymal transition and promotes metastasis of hepatocellular carcinoma by transcriptionally activating SNAIL. Cancer Res 2014;74:2750–2762.
10. Zhao Y, Wang X, Wang T, Hu X, Hui X, Yan M, et al. Acetylcholinesterase, a key prognostic predictor for hepatocellular carcinoma, suppresses cell growth and induces chemosensitization. Hepatology 2011;53:493–503.
11. Jia D, Wei L, Guo W, Zha R, Bao M, Chen Z, et al. Genome-wide copy number analyses identified novel cancer genes in hepatocellular carcinoma. Hepatology 2011;54:1227–1236.

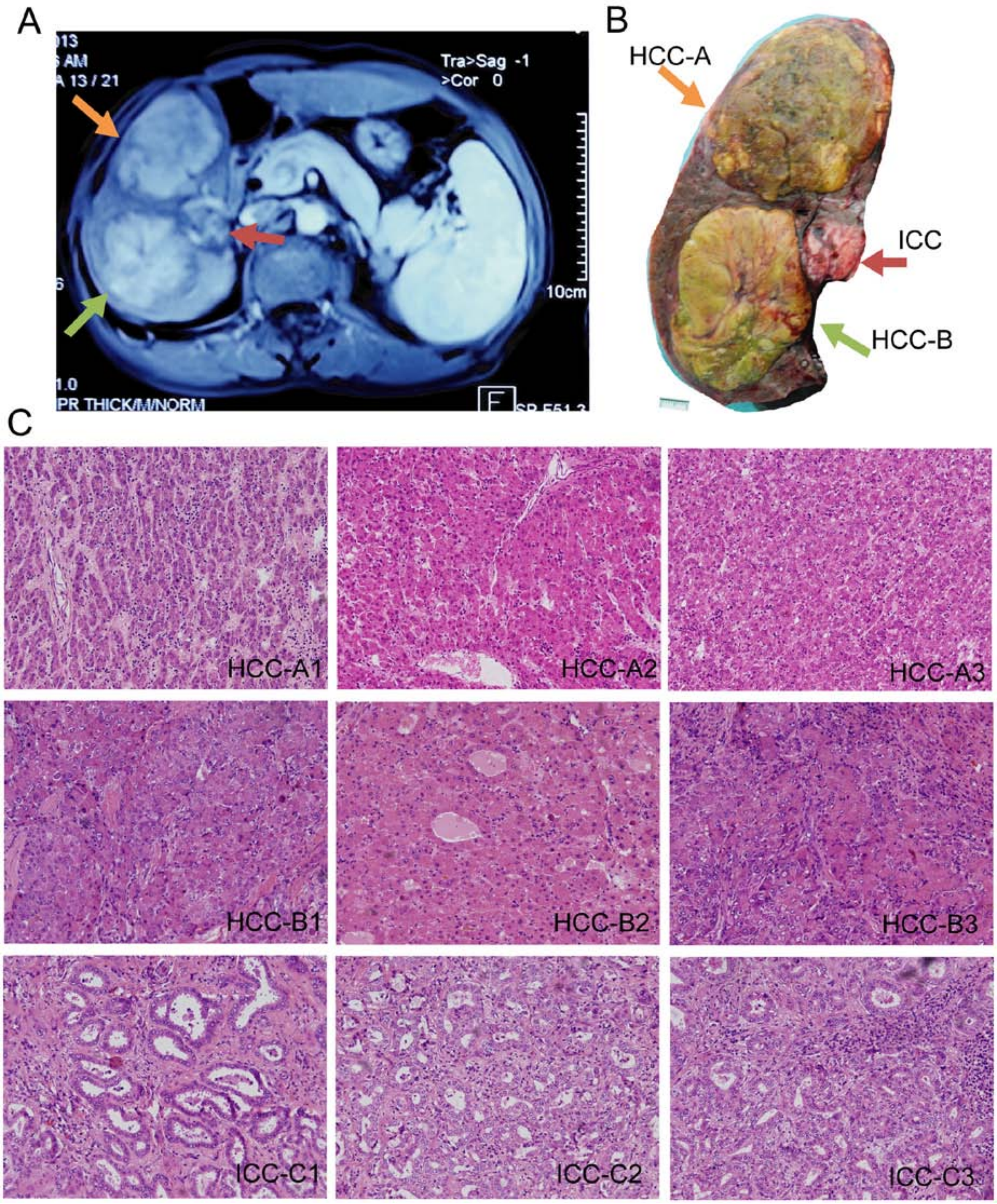

Supplementary Figure S1: Selection and morphologic features of one patient with multifocal primary liver cancer. A. Pre-operative MRI image showing three separated tumors within the liver. Orange arrow, HCC-A; Dark red arrow, HCC-B; Green arrow, ICC. **B.** Gross features of the resected three primary liver tumors. Scale bar, 1cm. **C.** Histological features of each sub-regoin from the three tumors by H.E. staining.

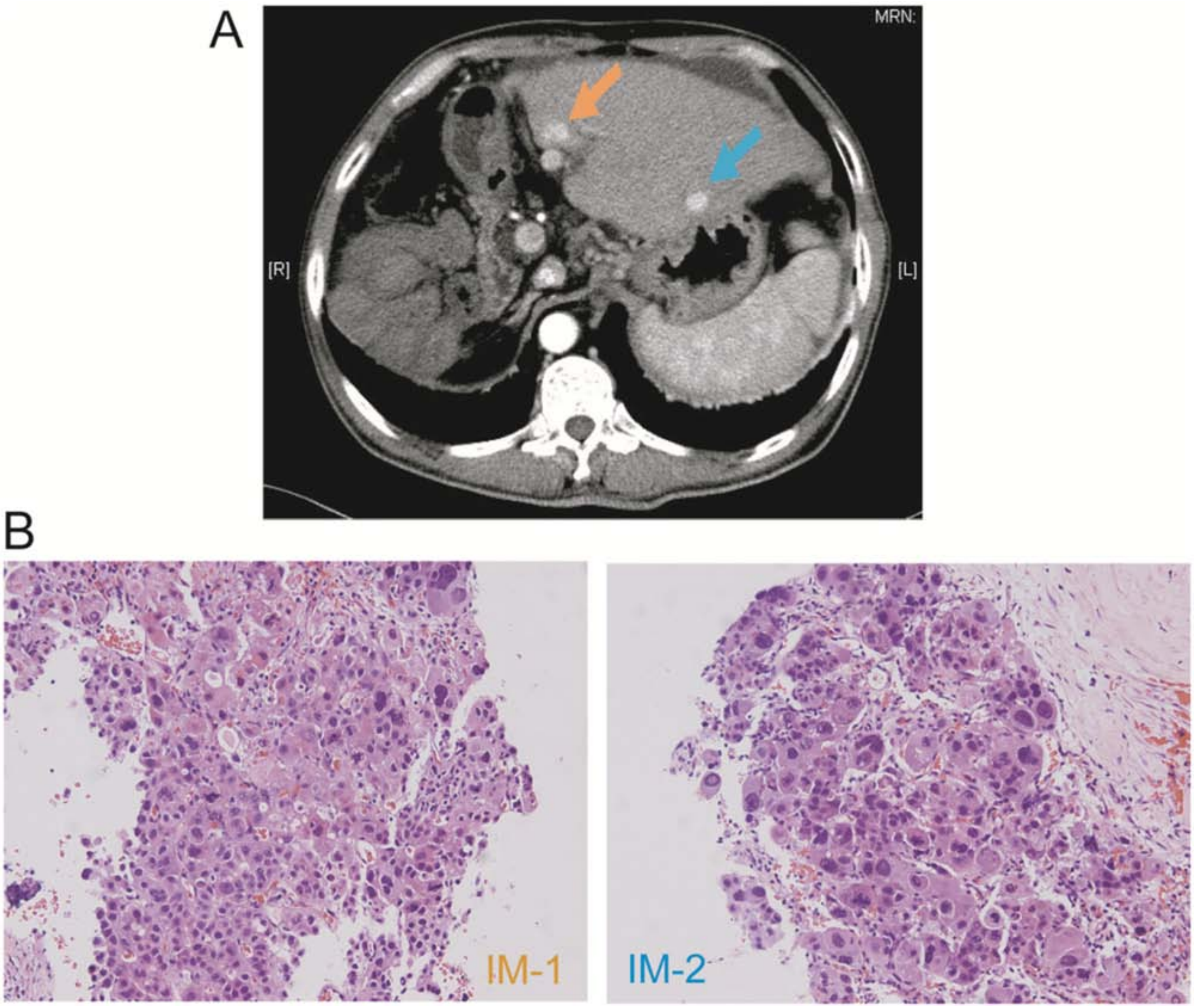

Supplementary Figure S2: Detection of two intrahepatic recurrent tumors in the patient 5 months after operation. A. CT scan image showing the two recurrent tumors within the liver. Orange arrow, recurrent tumor IM1; Blue arrow, recurrent tumor IM2. **B.** Histological features (H.E. staining) of the two recurrent tumors obtained by percutaneous needle biopsy.

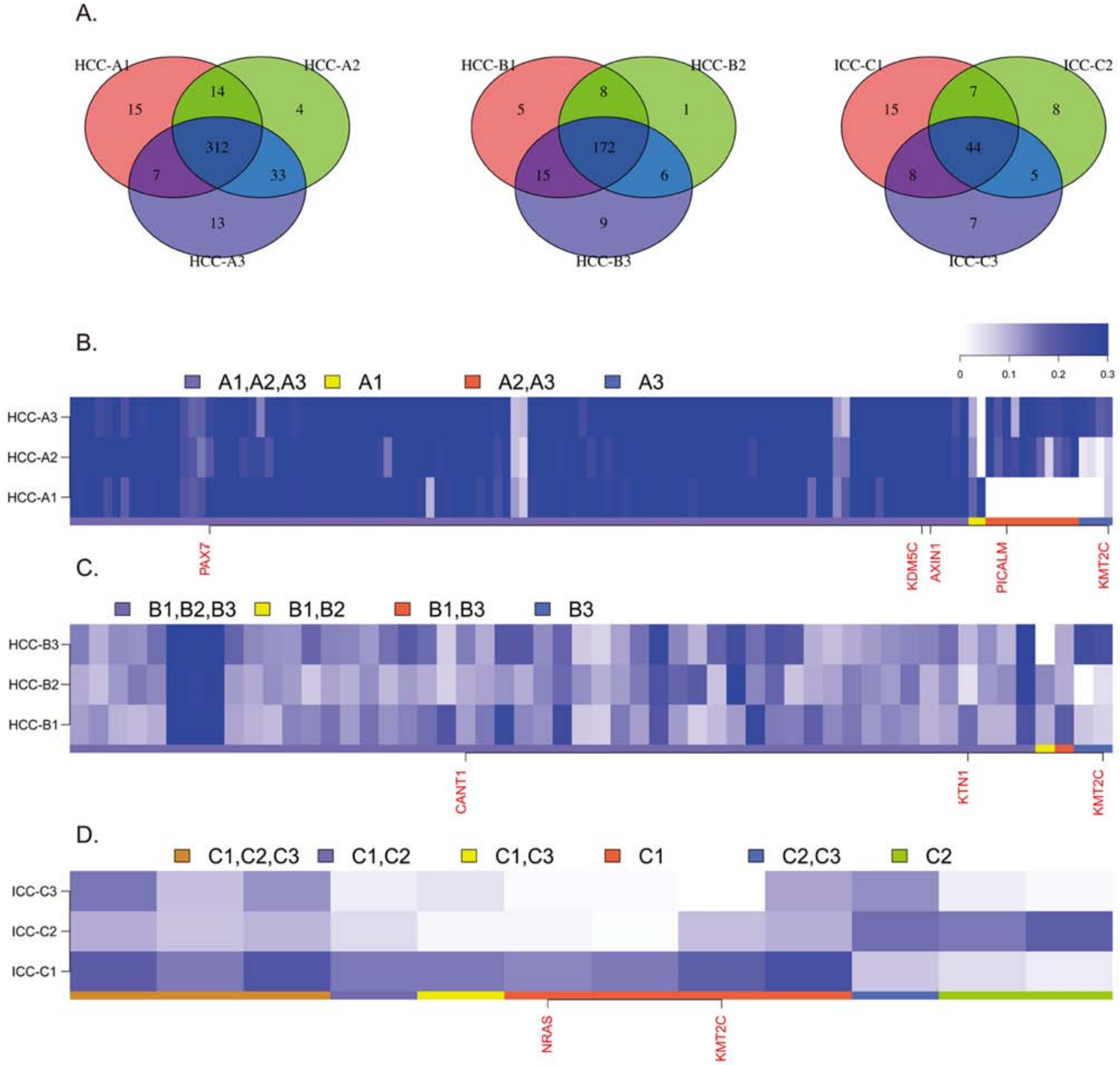

Supplementary Figure S3: Intratumor genetic heterogeneity among the three primary tumors. A. The Venn diagram of somatic mutations within the HCC-A, HCC-B and ICC sub-regions. **B, C.** and **D.** The VAF heatmaps of the HCC-A, HCC-B and ICC tumors, respectively.

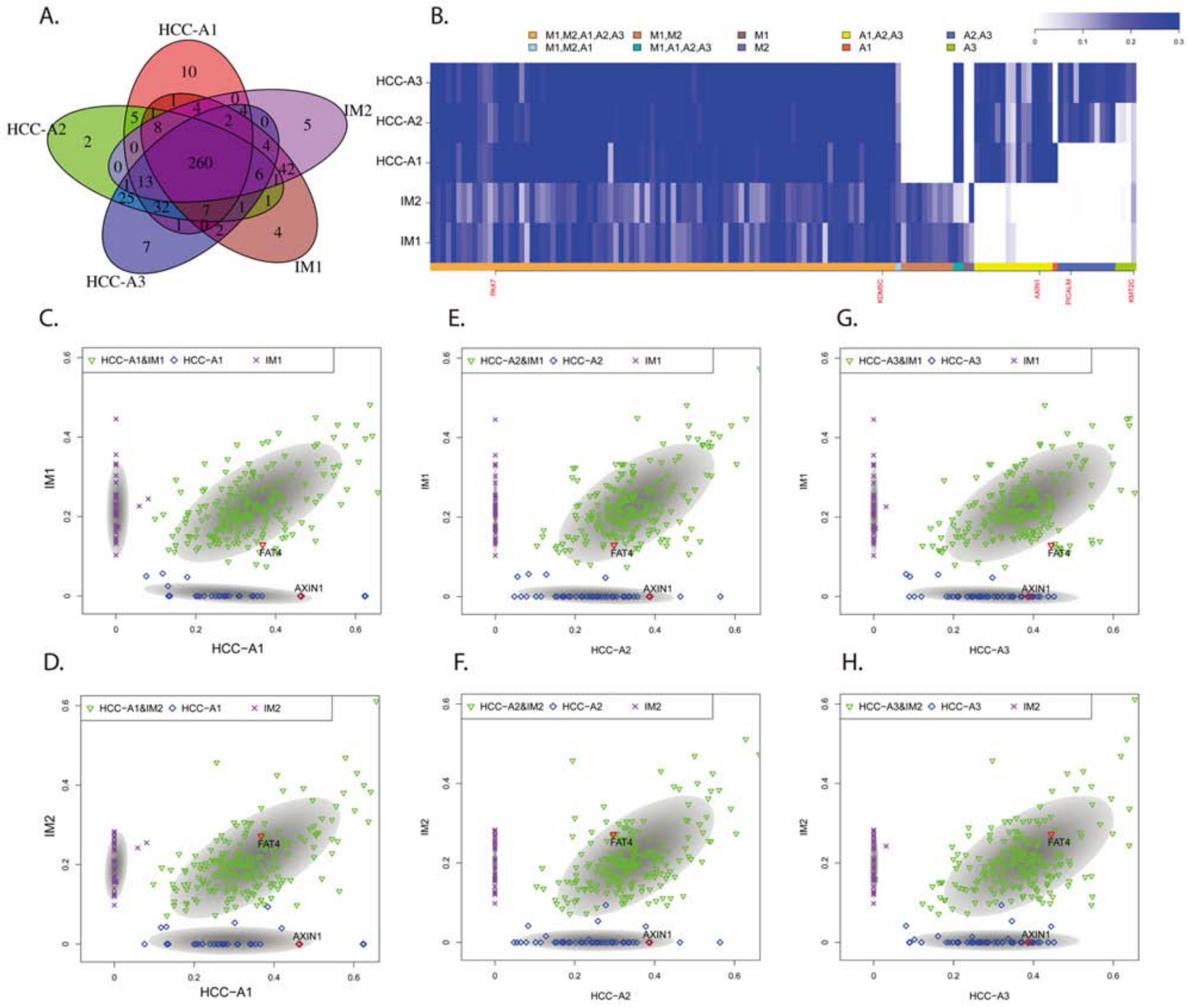

Supplementary Figure S4: Relationship between the recurrent tumors and the primary tumors. A. The Venn diagram of somatic mutations among HCC-A sub-regions and two recurrent tumors. A large fraction of mutations are shared by HCC-A and IM tumors, and both HCC-A and IM tumors have considerable amount of private mutations. **B.** The VAF heatmap of the HCC-A sub-regions and the two intrahepatic recurrent tumors. **C, D.** The scatter plots of VAFs at mutations detected in HCC-A1 and IM1 (C) and in HCC-A1 and IM2 (D) The mutations clearly clustered into 3 classes: the mutations only discovered in the IMs, the mutations only discovered in HCC-A1, and the mutations discovered in both IM and HCC-A1. For example, in (C), the crosses correspond to mutations detected in IM1 but not in HCC-A1 by the in-house method. The shaded regions are the 99% confidence regions of the mean VAFs of each class. The mutations are labeled by the in-house method. **E–H.** Similar to (C,D) but for HCC-A2 and HCC-A3 versus IM1/2.

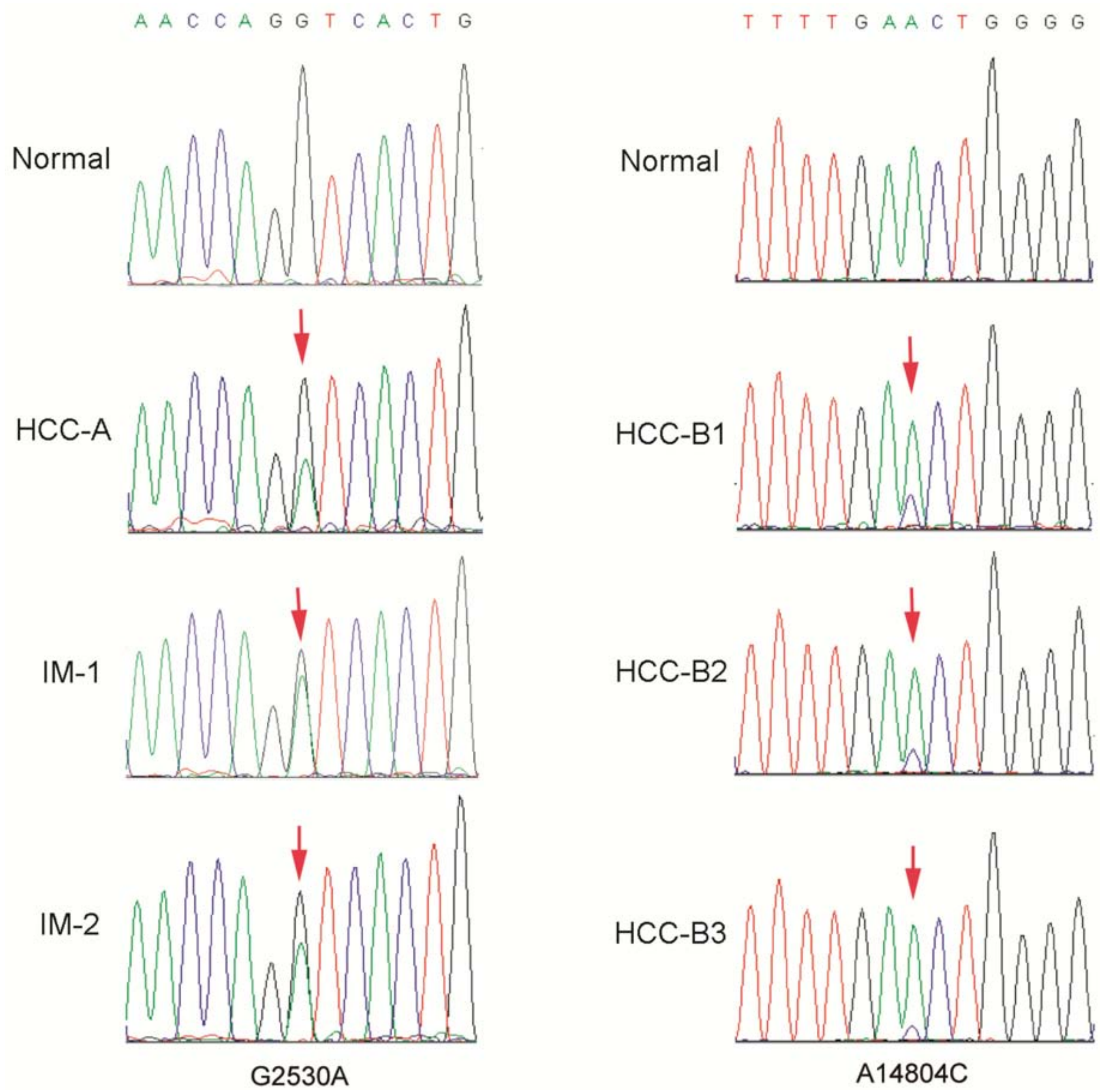

Supplementary Figure S5: Representative Sanger sequencing trace files of *FAT4* mutations detected in HCC-A, HCC-B and IMs. Three HCC-A sub-regions and IM1/2 had the c.G2530A mutation, while three HCC-B sub-regions had the c.A14804C mutation.

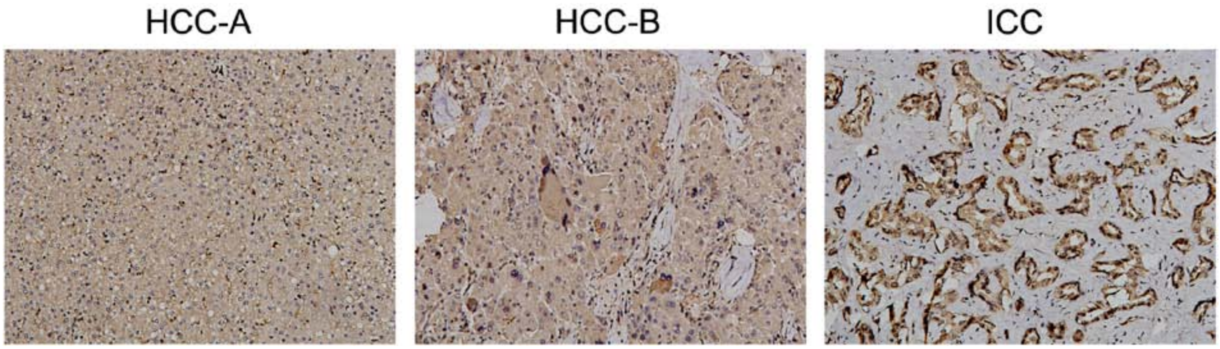

Supplementary Figure S6: Immunostaining of FAT4 in HCC-A, HCC-B and ICC. There were no significant differences of FAT4 expression levels in HCC-A and HCC-B (Score 1), however, the expression level in ICC (Score 2) is higher than that in HCC.

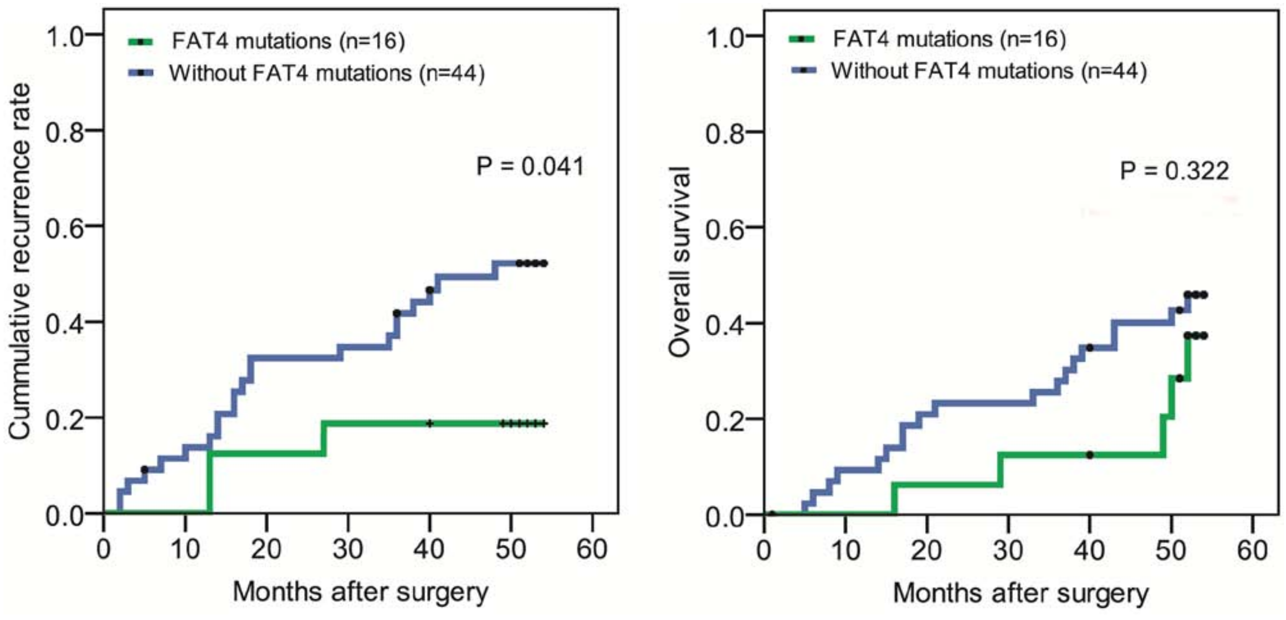

Supplementary Figure S7: Kaplan-Meier curves showing differences in recurrence and survival in HCC patients (*n* = 60), according to the mutation status of *FAT4* (log-rank test). The presence of *FAT4* mutations significantly correlated with increased recurrence.

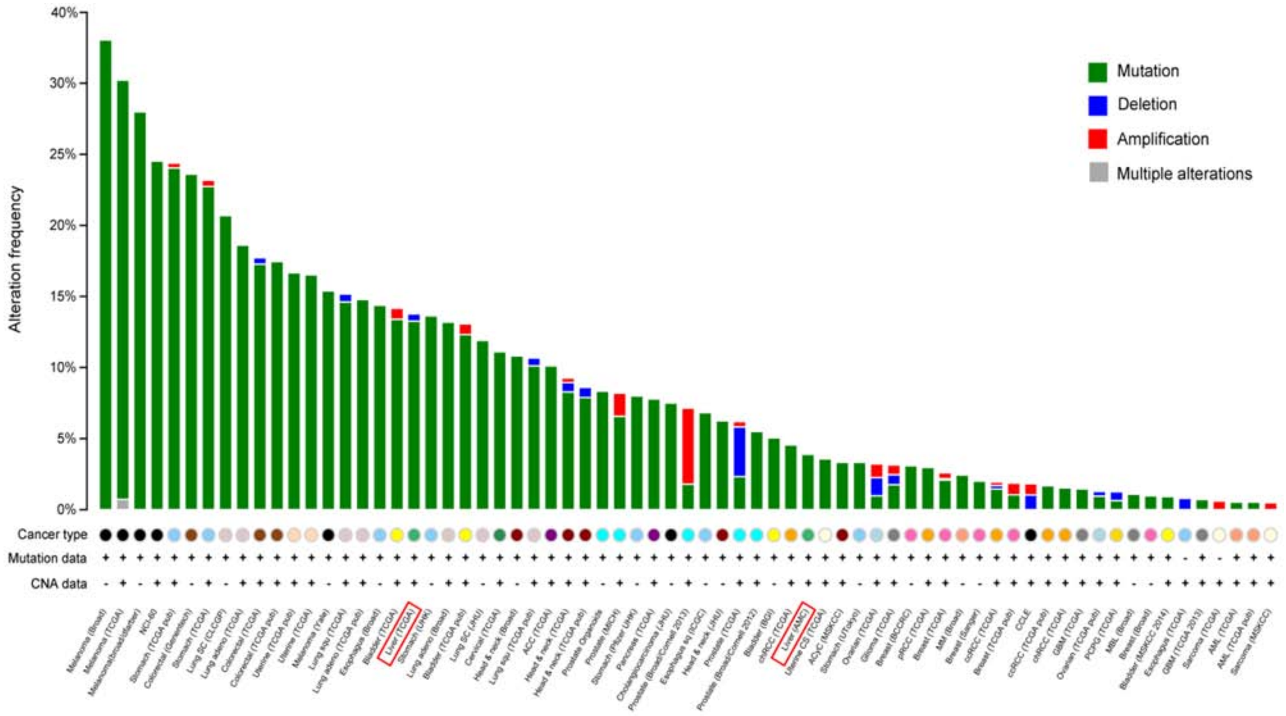

Supplementary Figure S8: Bar plot showing somatic mutations and copy number altration (CNA) in *FAT4* across different tumor types analyzed from cBioPortal database (http://www.cbioportal.org/public-portal). In hepatocellular carcinoma cases form the TCGA database, *FAT4* was mutated in 13.3% (26 cases) and deleted in 0.5% (1 cases) of 196 cases. In hepatocellular carcinoma cases form the AMC analysis (Hepatology 2014), *FAT4* was mutated in 3.9% (9 cases) of 231 cases.

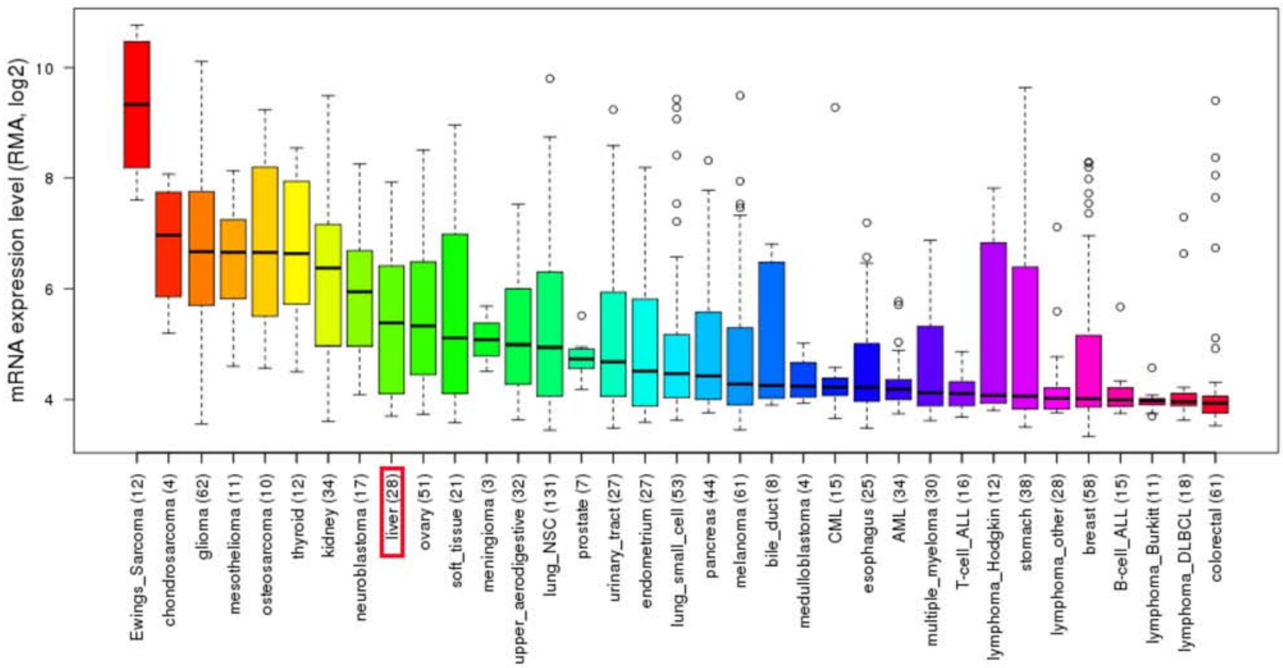

Supplementary Figure S9: Summary of relative *FAT4* mRNA expression among 910 human cancer cell lines from 35 cancer types derived from CCLE database (http://www.broadinstitute.org/ccle). *FAT4* mRNA level in hepatocellular carcinoma ranked as the top 9 among 35 cancer types. Number in parentheses indicates the cell line number of each cancer.

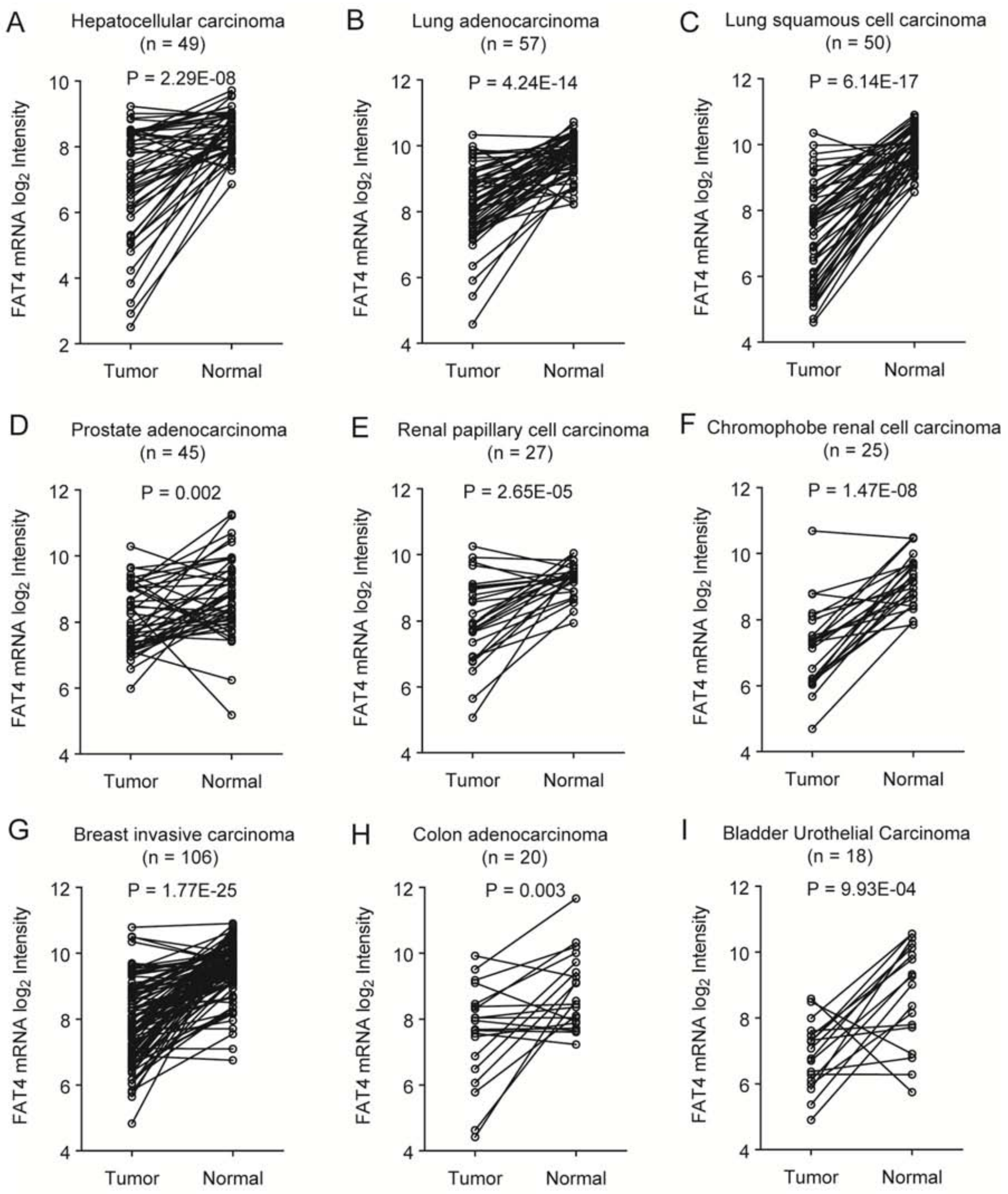

Supplementary Figure S10: Summary of relative *FAT4* mRNA level among 9 human cancers with paired tumor and normal samples analyzed from TCGA database (https://tcga-data.nci.nih.gov/tcga/tcgaHome2.jsp). *FAT4* mRNA expression was significantly down-regulated in hepatocellular carcinoma **A.** lung adenocarcinoma **B.** lung squamous cell carcinoma **C.** prostate adenocarcinoma **D.** renal papillary cell carcinoma **E.** chromophobe renal cell carcinoma **F.** breast carcinoma **G.** colon adenocarcinoma **H.** and bladder carcinoma **I.** Paired sample's *t* test.

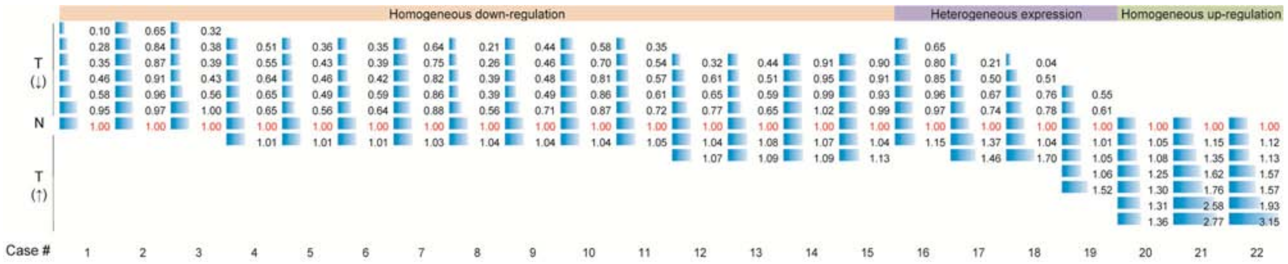

Supplementary Figure S11: Statistics for multiregional mRNA expression derived from 22 HCC. For each tumor, 6 spatially separated areas were sampled. Bar plots showed tumor sub-regional FAT4 mRNA expression levels relative to paired normal liver tissues. In case of co-existence of up- and down- regulation of *FAT4* within a tumor compared to paired normal tissue, 15% of increase or decrease than normal tissue was defined as intratumor heterogeneous expression. ↑, up-regulation of FAT4; ↓, down -regulation of FAT4.

Supplementary Table S1: Summary of whole-exome sequencing data

See Supplementary File 1

Supplementary Table S2: Summary of somatic substitutions and indels in the liver and tumor exomes

See Supplementary File 2

Supplementary Table S3: The list of non-synonymous somatic substitutions and small indels in coding and noncoding regions.

See Supplementary File 3

Supplementary Table S4: Regions with copy number variations.

See Supplementary File 4

Supplementary Table S5: Pathway enrichment of the genes with nonsynonymous somatic alterarions in coding regions of tumor exomes.

See Supplementary File 5

Supplementary Table S6: Somatic mutations of FAT4 in HCC samples and cell lines

See Supplementary File 6

Supplementary Table S7: HCC cell lines used in this study

See Supplementary File 7

Supplementary Table S8: Clinical characteristics of 60 HBV related-HCC patients for mutation prevelance screen

See Supplementary File 8

Supplementary Table S9: Clinical characteristics of 236 HBV related-HCC patients for immunostaining.

See Supplementary File 9

Supplementary Table S10: Primer sets used for PCR validation of somatic mutations.

See Supplementary File 10

Supplementary Table S11: List of PCR primers used for target sequencing of *FAT4*

See Supplementary File 11

Supplementary Table S12: Selection of anti-sequence for establishment of *FAT4* shRNA lentivirus

See Supplementary File 12

Supplementary Table S13: Selection of TALE-VP64 sequence for establishment of endogenous *FAT4* overexpression in HCC cells

See Supplementary File 13
