# Supplementary material for: Inferring the progression of multifocal liver cancer from spatial and temporal genomic heterogeneity
Source: Oncotarget. 2015 Dec 11;7(3):2867–77. doi: 10.18632/oncotarget.6558 (PMC4823077; doi:10.18632/oncotarget.6558)
Supplement: Supplementary file 2 [file oncotarget-07-2867-s002.docx]

| **Supplementary Table 1. Summary of whole-exome sequencing data.** | | | | | |  |  |  |
| --- | --- | --- | --- | --- | --- | --- | --- | --- |
| **Samples** | **Caculated tumor purity (%)** | **Total effective reads** | **Effective reads on target regions** | **Depth** | **Exome Cov.(%)** | **Cov. ≥10 reads** | **Cov. ≥20 reads** | **Ploidy** |
| Blood | / | 7,254,119,566 | 52,125,760 | 87.25 | 98.73% | 97.64% | 95.30% | 2.00 |
| TIS | 41% | 8,230,122,966 | 59,542,746 | 100.58 | 98.72% | 97.53% | 95.19% | 2.00 |
| HCC-A1 | 71% | 7,780,010,406 | 52,750,542 | 89.12 | 98.74% | 97.73% | 95.30% | 2.30 |
| HCC-A2 | 80% | 8,092,433,302 | 56,448,567 | 95.03 | 98.75% | 97.88% | 95.86% | 2.33 |
| HCC-A3 | 79% | 7,879,121,302 | 57,512,906 | 97.17 | 98.71% | 97.41% | 94.74% | 2.32 |
| HCC-B1 | 55% | 10,885,611,330 | 75,986,440 | 126.37 | 98.77% | 98.03% | 96.78% | 2.04 |
| HCC-B2 | 51% | 9,570,666,676 | 69,028,283 | 115.56 | 98.75% | 97.85% | 96.22% | 2.06 |
| HCC-B3 | 54% | 10,592,635,378 | 74,032,546 | 123.68 | 98.78% | 98.09% | 96.79% | 2.05 |
| ICC-1 | 32% | 8,041,634,544 | 51,452,954 | 86.30 | 98.77% | 97.84% | 95.67% | 2.00 |
| ICC-2 | 34% | 7,849,774,136 | 55,178,407 | 92.41 | 98.74% | 97.80% | 95.74% | 2.00 |
| ICC-3 | 31% | 7,511,455,850 | 53,601,058 | 89.73 | 98.74% | 97.71% | 95.53% | 2.00 |
| IM-1 | 45% | 6,984,630,558 | 52,516,746 | 78.05 | 98.68% | 96.99% | 93.11% | 2.32 |
| IM-2 | 44% | 8,328,990,856 | 58,012,502 | 93.62 | 98.71% | 97.50% | 94.87% | 2.32 |
|  | |  |  |  | | | | |
|  | | |  | | | | | |
|  | | |  | | | | | |
|  |  | |  | | | | | |
|  |  |  |  |  |  |  |  |  |
