# Supplementary material for: Inferring the progression of multifocal liver cancer from spatial and temporal genomic heterogeneity
Source: Oncotarget. 2015 Dec 11;7(3):2867–77. doi: 10.18632/oncotarget.6558 (PMC4823077; doi:10.18632/oncotarget.6558)
Supplement: Supplementary file 3 [file oncotarget-07-2867-s003.docx]

| **Supplementary Table 2. Summary of somatic substitutions and indels in the liver and tumor exomes.** | | | | | | | | | | | | |  |  |
| --- | --- | --- | --- | --- | --- | --- | --- | --- | --- | --- | --- | --- | --- | --- |
| **(a) Mutations detected by GATK** | | | | | | | | | | | | | | |
| **Sample** | **Missense** | **Stop Gained** | **Stop Lost** | **Near Splice** | **Synonymous SNV** | **Non-Coding SNVs** | **Coding SNVs** | **Total SNVs** | **Coding Indels** | **Non-coding Indels** | **Total Indels** | **FrameShift Indels** | **VAF Nonsynonymous SNVs** | **VAF all SNVs** |
| A1 | 102 | 5 | 0 | 19 | 46 | 140 | 153 | 293 | 4 | 7 | 11 | 4 | 0.35 | 0.36 |
| A2 | 109 | 5 | 0 | 18 | 48 | 134 | 162 | 296 | 4 | 9 | 13 | 4 | 0.34 | 0.35 |
| A3 | 112 | 5 | 0 | 19 | 48 | 147 | 165 | 312 | 4 | 8 | 12 | 4 | 0.37 | 0.38 |
| B1 | 26 | 2 | 0 | 7 | 21 | 59 | 49 | 108 | 0 | 6 | 6 | 0 | 0.18 | 0.23 |
| B2 | 25 | 2 | 0 | 5 | 17 | 51 | 44 | 95 | 0 | 2 | 2 | 0 | 0.17 | 0.22 |
| B3 | 33 | 2 | 0 | 5 | 26 | 64 | 61 | 125 | 0 | 6 | 6 | 0 | 0.18 | 0.23 |
| C1 | 9 | 0 | 0 | 1 | 5 | 22 | 14 | 36 | 0 | 8 | 8 | 0 | 0.16 | 0.33 |
| C2 | 2 | 0 | 0 | 1 | 3 | 17 | 5 | 22 | 0 | 6 | 6 | 0 | 0.17 | 0.49 |
| C3 | 2 | 0 | 0 | 0 | 4 | 15 | 6 | 21 | 0 | 5 | 5 | 0 | 0.15 | 0.43 |
| TIS | 3 | 0 | 0 | 1 | 2 | 12 | 5 | 17 | 0 | 0 | 0 | 0 | 0.21 | 0.49 |
| M1 | 96 | 2 | 0 | 15 | 53 | 133 | 151 | 284 | 1 | 5 | 6 | 1 | 0.24 | 0.27 |
| M2 | 88 | 2 | 0 | 15 | 45 | 132 | 135 | 267 | 0 | 7 | 7 | 0 | 0.23 | 0.26 |
| A | 117 | 5 | 0 | 19 | 53 | 169 | 175 | 344 | 4 | 15 | 19 | 4 | 0.36 | 0.36 |
| B | 48 | 4 | 0 | 8 | 34 | 92 | 86 | 178 | 0 | 12 | 12 | 0 | 0.18 | 0.23 |
| C | 11 | 0 | 0 | 2 | 8 | 34 | 19 | 53 | 0 | 16 | 16 | 0 | 0.16 | 0.40 |
| IM | 102 | 2 | 0 | 18 | 58 | 156 | 162 | 318 | 1 | 12 | 13 | 1 | 0.23 | 0.26 |
| **(b) Mutations detected by the in-house more sensitive method** | | | | | | | | | | | | | | |
| **Sample** | **Missense** | **Stop Gained** | **Stop Lost** | **Near Splice** | **Synonymous SNV** | **Non-Coding SNVs** | **Coding SNVs** | **Total SNVs** | **Coding Indels** | **Non-coding Indels** | **Total Indels** | **FrameShift Indels** | **VAF Nonsynonymous SNVs** | **VAF all SNVs** |
| A1 | 106 | 5 | 0 | 20 | 51 | 161 | 162 | 323 | 4 | 21 | 25 | 4 | 0.34 | 0.35 |
| A2 | 113 | 5 | 0 | 20 | 53 | 169 | 171 | 340 | 4 | 19 | 23 | 4 | 0.33 | 0.34 |
| A3 | 118 | 5 | 0 | 19 | 54 | 167 | 177 | 344 | 4 | 17 | 21 | 4 | 0.36 | 0.37 |
| B1 | 48 | 4 | 0 | 8 | 33 | 95 | 85 | 180 | 0 | 20 | 20 | 0 | 0.15 | 0.20 |
| B2 | 47 | 4 | 0 | 8 | 31 | 91 | 82 | 173 | 0 | 14 | 14 | 0 | 0.14 | 0.18 |
| B3 | 49 | 4 | 0 | 7 | 34 | 95 | 87 | 182 | 0 | 20 | 20 | 0 | 0.15 | 0.21 |
| C1 | 9 | 0 | 0 | 2 | 9 | 39 | 18 | 57 | 0 | 17 | 17 | 0 | 0.16 | 0.33 |
| C2 | 7 | 0 | 0 | 2 | 6 | 33 | 13 | 46 | 0 | 18 | 18 | 0 | 0.12 | 0.38 |
| C3 | 5 | 0 | 0 | 2 | 7 | 36 | 12 | 48 | 0 | 16 | 16 | 0 | 0.10 | 0.33 |
| TIS | 3 | 0 | 0 | 1 | 5 | 28 | 8 | 36 | 0 | 14 | 14 | 0 | 0.21 | 0.43 |
| M1 | 102 | 2 | 0 | 19 | 57 | 161 | 161 | 322 | 3 | 19 | 22 | 3 | 0.23 | 0.26 |
| M2 | 101 | 2 | 0 | 18 | 58 | 163 | 161 | 324 | 4 | 22 | 26 | 4 | 0.21 | 0.24 |
| A | 121 | 5 | 0 | 20 | 58 | 181 | 184 | 365 | 4 | 29 | 33 | 4 | 0.35 | 0.36 |
| B | 50 | 4 | 0 | 8 | 35 | 102 | 89 | 191 | 0 | 25 | 25 | 0 | 0.15 | 0.20 |
| C | 12 | 0 | 0 | 2 | 11 | 45 | 23 | 68 | 0 | 26 | 26 | 0 | 0.13 | 0.35 |
| IM | 104 | 2 | 0 | 19 | 60 | 170 | 166 | 336 | 4 | 27 | 31 | 4 | 0.22 | 0.25 |
|  |  |  |  |  |  |  |  |  |  |  |  |  |  |  |
|  |  |  |  |  |  |  |  |  |  |  |  |  |  |  |
