# Supplementary material for: Inferring the progression of multifocal liver cancer from spatial and temporal genomic heterogeneity
Source: Oncotarget. 2015 Dec 11;7(3):2867–77. doi: 10.18632/oncotarget.6558 (PMC4823077; doi:10.18632/oncotarget.6558)
Supplement: Supplementary file 4 [file oncotarget-07-2867-s004.docx]

| **Supplementary Table 3. The list of non-synonymous somatic substitutions and small indels in coding and noncoding regions.** | | | | | | | | | | |
| --- | --- | --- | --- | --- | --- | --- | --- | --- | --- | --- |
| **Sample** | **MutationType** | **Chromosome** | **Pos** | **Ref** | **Alt** | **Function GVS** | **Gene** | **Exon** | **Cancer Census Gene** | **GATK** |
| HCC-A1 | somaticSNP | 1 | 15855461 | T | G | intron | DNAJC16 |  |  | Y |
| HCC-A1 | somaticSNP | 1 | 17023483 | C | T | intron | ESPNP |  |  | N |
| HCC-A1 | somaticSNP | 1 | 19062153 | G | A | missense | PAX7 | PAX7 | PAX7 | Y |
| HCC-A1 | somaticSNP | 1 | 22158261 | T | C | missense | HSPG2 | HSPG2 |  | Y |
| HCC-A1 | somaticSNP | 1 | 26738094 | T | G | intron | LIN28A |  |  | Y |
| HCC-A1 | somaticSNP | 1 | 26873700 | C | T | 5-prime-UTR,synonymous | RPS6KA1 | RPS6KA1 |  | N |
| HCC-A1 | somaticSNP | 1 | 36939412 | T | G | synonymous | CSF3R | CSF3R | CSF3R | Y |
| HCC-A1 | somaticSNP | 1 | 41327871 | C | A | 5-prime-UTR | CITED4 | CITED4 |  | N |
| HCC-A1 | somaticSNP | 1 | 53279594 | A | G | intron | ZYG11B |  |  | Y |
| HCC-A1 | somaticSNP | 1 | 62503632 | A | G | intron | INADL |  |  | Y |
| HCC-A1 | somaticSNP | 1 | 78267110 | G | A | missense | FAM73A | FAM73A |  | Y |
| HCC-A1 | somaticSNP | 1 | 86814427 | G | T | 3-prime-UTR | ODF2L | ODF2L |  | N |
| HCC-A1 | somaticINDEL | 1 | 93159821 | C | -A | intron | EVI5 |  |  | N |
| HCC-A1 | somaticSNP | 1 | 103385782 | T | C | intron | COL11A1 |  |  | Y |
| HCC-A1 | somaticINDEL | 1 | 103471734 | A | +AAATAAATAAAT | intron | COL11A1 |  |  | N |
| HCC-A1 | somaticSNP | 1 | 110129627 | G | T | intron | GNAI3 |  |  | Y |
| HCC-A1 | somaticSNP | 1 | 111217382 | C | T | missense | KCNA3 | KCNA3 |  | N |
| HCC-A1 | somaticSNP | 1 | 145281824 | T | G | intron | NBPF20,NBPF9,NOTCH2NL |  |  | Y |
| HCC-A1 | somaticSNP | 1 | 153642826 | C | T | intron | ILF2 |  |  | Y |
| HCC-A1 | somaticSNP | 1 | 156845864 | C | A | intron-near-splice,synonymous-near-splice | NTRK1 |  | NTRK1 | Y |
| HCC-A1 | somaticSNP | 1 | 158435706 | G | T | missense | OR10K1 | OR10K1 |  | Y |
| HCC-A1 | somaticSNP | 1 | 158687102 | A | G | synonymous | OR6K3 | OR6K3 |  | Y |
| HCC-A1 | somaticSNP | 1 | 165180036 | T | C | intron | LMX1A |  |  | Y |
| HCC-A1 | somaticSNP | 1 | 166959161 | T | C | intron | MAEL |  |  | Y |
| HCC-A1 | somaticSNP | 1 | 186122844 | A | G | intron | HMCN1,MIR548F1 |  |  | Y |
| HCC-A1 | somaticSNP | 1 | 205034434 | G | T | intron | CNTN2 |  |  | Y |
| HCC-A1 | somaticSNP | 1 | 227227740 | A | C | intron | CDC42BPA |  |  | N |
| HCC-A1 | somaticSNP | 1 | 237773958 | A | T | intron | RYR2 |  |  | N |
| HCC-A1 | somaticSNP | 1 | 67559220 | T | C | 3-prime-UTR,missense,non-coding-exon | C1orf141 | C1orf141 |  | Y |
| HCC-A1 | somaticSNP | 1 | 227922773 | G | C | 5-prime-UTR,missense | SNAP47,JMJD4 | SNAP47,JMJD4 |  | Y |
| HCC-A1 | somaticSNP | 1 | 247921652 | G | A | intron,synonymous | OR1C1 | OR1C1 |  | Y |
| HCC-A1 | somaticSNP | 1 | 21012616 | G | A | missense | KIF17 | KIF17 |  | Y |
| HCC-A1 | somaticSNP | 1 | 34180246 | C | T | missense | CSMD2 | CSMD2 |  | Y |
| HCC-A1 | somaticSNP | 1 | 120306816 | T | C | missense | HMGCS2 | HMGCS2 |  | Y |
| HCC-A1 | somaticSNP | 1 | 157514235 | C | G | missense | FCRL5 | FCRL5 |  | Y |
| HCC-A1 | somaticSNP | 1 | 168250404 | G | A | missense | TBX19 | TBX19 |  | Y |
| HCC-A1 | somaticSNP | 1 | 228509727 | T | A | missense | OBSCN | OBSCN |  | Y |
| HCC-A1 | somaticSNP | 1 | 228547462 | C | T | intron,missense | OBSCN | OBSCN |  | Y |
| HCC-A1 | somaticSNP | 2 | 21239443 | G | T | missense | APOB | APOB |  | Y |
| HCC-A1 | somaticSNP | 2 | 31412096 | T | C | intron | CAPN14 |  |  | Y |
| HCC-A1 | somaticSNP | 2 | 54891911 | A | T | intron | SPTBN1 |  |  | Y |
| HCC-A1 | somaticSNP | 2 | 55433542 | T | C | intron | CLHC1 |  |  | N |
| HCC-A1 | somaticSNP | 2 | 61020681 | A | T | intron | PAPOLG |  |  | Y |
| HCC-A1 | somaticSNP | 2 | 86422665 | A | G | 5-prime-UTR | IMMT | IMMT |  | Y |
| HCC-A1 | somaticSNP | 2 | 98833151 | T | G | intron | VWA3B |  |  | Y |
| HCC-A1 | somaticSNP | 2 | 116447348 | A | G | intron | DPP10 |  |  | Y |
| HCC-A1 | somaticSNP | 2 | 118701541 | T | C | intron | CCDC93 |  |  | Y |
| HCC-A1 | somaticSNP | 2 | 128522167 | G | A | intron,synonymous | WDR33 | WDR33 |  | Y |
| HCC-A1 | somaticSNP | 2 | 151331371 | T | C | intron | RND3 |  |  | Y |
| HCC-A1 | somaticSNP | 2 | 153551068 | C | T | synonymous | PRPF40A | PRPF40A |  | Y |
| HCC-A1 | somaticSNP | 2 | 153574008 | C | A | 5-prime-UTR,missense | PRPF40A | PRPF40A |  | Y |
| HCC-A1 | somaticSNP | 2 | 166908325 | G | T | missense | SCN1A | SCN1A |  | Y |
| HCC-A1 | somaticSNP | 2 | 176857148 | T | A | splice-3 | KIAA1715 |  |  | N |
| HCC-A1 | somaticSNP | 2 | 177034400 | G | A | intron | HOXD3 |  |  | Y |
| HCC-A1 | somaticSNP | 2 | 201756675 | A | G | intron,synonymous | NIF3L1 | NIF3L1 |  | Y |
| HCC-A1 | somaticINDEL | 2 | 201778717 | T | +A | intron | ORC2 |  |  | N |
| HCC-A1 | somaticSNP | 2 | 210877639 | A | T | intron | RPE |  |  | Y |
| HCC-A1 | somaticSNP | 2 | 220358383 | G | T | upstream-gene |  | |  | Y |
| HCC-A1 | somaticSNP | 2 | 228846479 | T | G | missense | SPHKAP | SPHKAP |  | Y |
| HCC-A1 | somaticSNP | 2 | 231152571 | A | T | intron | SP140 |  |  | N |
| HCC-A1 | somaticSNP | 2 | 236708207 | A | G | intron | AGAP1 |  |  | Y |
| HCC-A1 | somaticSNP | 2 | 242800900 | G | A | intron | PDCD1 |  |  | Y |
| HCC-A1 | somaticSNP | 2 | 29379290 | A | C | missense | CLIP4 | CLIP4 |  | Y |
| HCC-A1 | somaticSNP | 3 | 195517843 | G | A | intron,missense | MUC4 | MUC4 |  | Y |
| HCC-A1 | somaticSNP | 3 | 403347 | A | G | intron | CHL1 |  |  | Y |
| HCC-A1 | somaticINDEL | 3 | 19389266 | C | +A | nonsynonymous,ins,frameshift | KCNH8 | KCNH8 |  | Y |
| HCC-A1 | somaticSNP | 3 | 38051009 | C | T | intron | PLCD1 |  |  | Y |
| HCC-A1 | somaticSNP | 3 | 39448229 | A | G | 5-prime-UTR | RPSA | RPSA |  | Y |
| HCC-A1 | somaticSNP | 3 | 44283610 | T | A | missense | TOPAZ1 | TOPAZ1 |  | Y |
| HCC-A1 | somaticSNP | 3 | 49311526 | T | A | missense | C3orf62 | C3orf62 |  | Y |
| HCC-A1 | somaticINDEL | 3 | 57108374 | T | -C | nonsynonymous,del,frameshift | SPATA12,ARHGEF3 | SPATA12 |  | Y |
| HCC-A1 | somaticSNP | 3 | 69112049 | C | T | intron | UBA3 |  |  | Y |
| HCC-A1 | somaticSNP | 3 | 97464291 | T | A | intron | EPHA6 |  |  | Y |
| HCC-A1 | somaticSNP | 3 | 100084408 | T | C | synonymous | TOMM70A | TOMM70A |  | Y |
| HCC-A1 | somaticSNP | 3 | 121132268 | T | A | intron | STXBP5L |  |  | N |
| HCC-A1 | somaticSNP | 3 | 124449461 | G | A | 5-prime-UTR,missense,non-coding-exon | UMPS | UMPS |  | Y |
| HCC-A1 | somaticSNP | 3 | 133877257 | C | T | intron | RYK |  |  | Y |
| HCC-A1 | somaticSNP | 3 | 142840069 | T | A | synonymous | CHST2 | CHST2 |  | Y |
| HCC-A1 | somaticINDEL | 3 | 150282061 | C | -T | intron | EIF2A |  |  | N |
| HCC-A1 | somaticSNP | 3 | 184299095 | A | C | synonymous | EPHB3 | EPHB3 |  | Y |
| HCC-A1 | somaticSNP | 3 | 185879581 | T | A | intron | DGKG |  |  | Y |
| HCC-A1 | somaticSNP | 3 | 193855537 | G | T | stop-gained | HES1 | HES1 |  | Y |
| HCC-A1 | somaticSNP | 3 | 194118705 | G | A | synonymous | GP5 | GP5 |  | Y |
| HCC-A1 | somaticSNP | 4 | 2900240 | G | A | synonymous | ADD1 | ADD1 |  | Y |
| HCC-A1 | somaticSNP | 4 | 10020564 | G | A | intron | SLC2A9 |  |  | N |
| HCC-A1 | somaticINDEL | 4 | 26241366 | G | +GGAGA | intron | RBPJ |  |  | N |
| HCC-A1 | somaticINDEL | 4 | 74281947 | A | -TT | intron | ALB |  |  | Y |
| HCC-A1 | somaticSNP | 4 | 90034354 | G | C | missense | TIGD2 | TIGD2 |  | Y |
| HCC-A1 | somaticSNP | 4 | 103556316 | T | C | intron | MANBA |  |  | Y |
| HCC-A1 | somaticSNP | 4 | 122068167 | T | C | intron | TNIP3 |  |  | Y |
| HCC-A1 | somaticSNP | 4 | 125631753 | T | C | 5-prime-UTR,intron | ANKRD50 | ANKRD50 |  | Y |
| HCC-A1 | somaticSNP | 4 | 73164017 | T | A | missense | ADAMTS3 | ADAMTS3 |  | Y |
| HCC-A1 | somaticSNP | 4 | 126240096 | G | A | missense | FAT4 | FAT4 |  | Y |
| HCC-A1 | somaticSNP | 4 | 155507484 | T | A | missense | FGA | FGA |  | Y |
| HCC-A1 | somaticSNP | 4 | 155533047 | A | G | missense | FGG | FGG |  | Y |
| HCC-A1 | somaticSNP | 5 | 16701535 | T | G | missense | MYO10 | MYO10 |  | Y |
| HCC-A1 | somaticSNP | 5 | 76714070 | T | A | stop-gained | PDE8B | PDE8B |  | Y |
| HCC-A1 | somaticSNP | 5 | 7414718 | A | G | synonymous | ADCY2 | ADCY2 |  | Y |
| HCC-A1 | somaticINDEL | 5 | 78416973 | C | -A | intron | BHMT |  |  | N |
| HCC-A1 | somaticSNP | 5 | 135396468 | T | A | intron | TGFBI |  |  | N |
| HCC-A1 | somaticSNP | 5 | 145531578 | G | A | intron | LARS |  |  | Y |
| HCC-A1 | somaticSNP | 5 | 149112300 | T | G | intron | PPARGC1B |  |  | N |
| HCC-A1 | somaticSNP | 5 | 169309494 | T | A | intron | DOCK2,FAM196B |  |  | Y |
| HCC-A1 | somaticSNP | 5 | 169412889 | C | T | missense | DOCK2 | DOCK2 |  | Y |
| HCC-A1 | somaticSNP | 5 | 172110938 | G | A | intron,missense | NEURL1B | NEURL1B |  | Y |
| HCC-A1 | somaticSNP | 6 | 5000779 | G | A | intron | RPP40 |  |  | N |
| HCC-A1 | somaticINDEL | 6 | 24651352 | T | -A | intron | TDP2 |  |  | N |
| HCC-A1 | somaticSNP | 6 | 28197605 | C | T | intron | ZSCAN9 |  |  | Y |
| HCC-A1 | somaticSNP | 6 | 33653560 | C | A | missense | ITPR3 | ITPR3 |  | Y |
| HCC-A1 | somaticSNP | 6 | 34029881 | C | T | intron | GRM4 |  |  | Y |
| HCC-A1 | somaticSNP | 6 | 71233675 | A | G | missense | FAM135A | FAM135A |  | Y |
| HCC-A1 | somaticSNP | 6 | 75801024 | G | T | intron | COL12A1 |  |  | Y |
| HCC-A1 | somaticSNP | 6 | 99848727 | T | C | missense | PNISR | PNISR |  | Y |
| HCC-A1 | somaticSNP | 6 | 108831414 | G | A | splice-acceptor | LACE1 | LACE1 |  | Y |
| HCC-A1 | somaticSNP | 6 | 111329423 | G | T | intron | RPF2 |  |  | Y |
| HCC-A1 | somaticSNP | 6 | 129799721 | T | C | intron | LAMA2 |  |  | Y |
| HCC-A1 | somaticSNP | 6 | 132874297 | T | A | missense | TAAR8 | TAAR8 |  | Y |
| HCC-A1 | somaticSNP | 6 | 144508629 | C | A | 3-prime-UTR | STX11 | STX11 |  | Y |
| HCC-A1 | somaticSNP | 6 | 167426949 | A | G | intron | FGFR1OP |  | FGFR1OP | Y |
| HCC-A1 | somaticSNP | 6 | 167446139 | A | G | intron | FGFR1OP |  | FGFR1OP | Y |
| HCC-A1 | somaticSNP | 6 | 170034675 | A | T | intron | WDR27 |  |  | Y |
| HCC-A1 | somaticSNP | 6 | 11104238 | C | A | stop-gained,intron | SMIM13,ERVFRD-1 | ERVFRD-1 |  | Y |
| HCC-A1 | somaticSNP | 6 | 149771895 | T | A | missense | ZC3H12D | ZC3H12D |  | Y |
| HCC-A1 | somaticSNP | 6 | 170049362 | C | A | missense | WDR27 | WDR27 |  | Y |
| HCC-A1 | somaticSNP | 7 | 1914350 | A | G | intron | MAD1L1 |  |  | N |
| HCC-A1 | somaticSNP | 7 | 4304941 | G | T | intron,missense | SDK1 | SDK1 |  | Y |
| HCC-A1 | somaticSNP | 7 | 12610475 | C | T | synonymous | SCIN | SCIN |  | Y |
| HCC-A1 | somaticSNP | 7 | 19156335 | G | T | 3-prime-UTR | TWIST1 | TWIST1 |  | Y |
| HCC-A1 | somaticINDEL | 7 | 22330862 | T | +A | intron | RAPGEF5 |  |  | Y |
| HCC-A1 | somaticINDEL | 7 | 23296415 | G | +A | intron | GPNMB |  |  | Y |
| HCC-A1 | somaticSNP | 7 | 26404329 | T | C | intron | SNX10 |  |  | Y |
| HCC-A1 | somaticSNP | 7 | 32914895 | T | G | intron | KBTBD2 |  |  | Y |
| HCC-A1 | somaticSNP | 7 | 44619235 | G | A | intron-near-splice | TMED4 |  |  | Y |
| HCC-A1 | somaticSNP | 7 | 48146967 | G | A | missense | UPP1 | UPP1 |  | Y |
| HCC-A1 | somaticINDEL | 7 | 48412112 | T | -A | intron | ABCA13 |  |  | N |
| HCC-A1 | somaticSNP | 7 | 63726690 | C | A | missense | ZNF679 | ZNF679 |  | Y |
| HCC-A1 | somaticSNP | 7 | 80374631 | A | G | intron-near-splice | SEMA3C |  |  | Y |
| HCC-A1 | somaticSNP | 7 | 99578370 | T | G | upstream-gene |  | |  | Y |
| HCC-A1 | somaticSNP | 7 | 99578381 | T | C | upstream-gene |  | |  | Y |
| HCC-A1 | somaticSNP | 7 | 99578411 | T | C | non-coding-exon | AZGP1P1 | AZGP1P1 |  | Y |
| HCC-A1 | somaticSNP | 7 | 107332509 | G | A | intron,missense | SLC26A4 |  |  | Y |
| HCC-A1 | somaticINDEL | 7 | 116409675 | C | -T | intron | MET |  | MET | N |
| HCC-A1 | somaticSNP | 7 | 131982688 | C | A | intron | PLXNA4 |  |  | Y |
| HCC-A1 | somaticSNP | 7 | 142045484 | C | A | intergenic |  | |  | Y |
| HCC-A1 | somaticSNP | 7 | 142561350 | G | T | intron | EPHB6 |  |  | Y |
| HCC-A1 | somaticSNP | 7 | 143095395 | C | T | intron | EPHA1 |  |  | Y |
| HCC-A1 | somaticSNP | 7 | 143573132 | C | T | intron,synonymous | FAM115A | FAM115A |  | Y |
| HCC-A1 | somaticSNP | 7 | 12409830 | T | C | missense | VWDE | VWDE |  | Y |
| HCC-A1 | somaticSNP | 8 | 105393433 | T | A | missense | DPYS | DPYS |  | Y |
| HCC-A1 | somaticSNP | 8 | 35579196 | T | C | intron | UNC5D |  |  | N |
| HCC-A1 | somaticSNP | 8 | 39103668 | C | G | missense | ADAM32 | ADAM32 |  | Y |
| HCC-A1 | somaticSNP | 8 | 69013781 | T | A | intron | PREX2 |  |  | Y |
| HCC-A1 | somaticSNP | 8 | 69351923 | A | C | intron | C8orf34 |  |  | Y |
| HCC-A1 | somaticSNP | 8 | 73150337 | A | T | non-coding-exon | LOC392232 | LOC392232 |  | Y |
| HCC-A1 | somaticSNP | 8 | 73479912 | T | A | 5-prime-UTR | KCNB2 | KCNB2 |  | Y |
| HCC-A1 | somaticSNP | 8 | 104897740 | A | G | missense | RIMS2 | RIMS2 |  | Y |
| HCC-A1 | somaticSNP | 8 | 113418760 | A | T | synonymous | CSMD3 | CSMD3 |  | Y |
| HCC-A1 | somaticSNP | 8 | 125047646 | C | T | intron,synonymous | FER1L6,FER1L6-AS1 | FER1L6 |  | Y |
| HCC-A1 | somaticSNP | 8 | 131964000 | C | A | intron | ADCY8 |  |  | Y |
| HCC-A1 | somaticSNP | 8 | 141712834 | T | C | intron | PTK2 |  |  | Y |
| HCC-A1 | somaticSNP | 8 | 143614849 | G | T | intron | BAI1 |  |  | Y |
| HCC-A1 | somaticSNP | 8 | 146220171 | T | C | intron | ZNF252P |  |  | Y |
| HCC-A1 | somaticSNP | 9 | 84202674 | C | G | synonymous | TLE1 | TLE1 |  | Y |
| HCC-A1 | somaticSNP | 9 | 91360763 | G | A | non-coding-exon | MIR4289 | MIR4289 |  | Y |
| HCC-A1 | somaticSNP | 9 | 130860849 | C | T | intron,missense,non-coding-exon | SLC25A25 | SLC25A25 |  | Y |
| HCC-A1 | somaticSNP | 9 | 98766997 | G | T | intron | ERCC6L2 |  |  | Y |
| HCC-A1 | somaticSNP | 9 | 99314276 | G | C | intron | CDC14B |  |  | Y |
| HCC-A1 | somaticSNP | 9 | 105763940 | T | C | intron | CYLC2 |  |  | Y |
| HCC-A1 | somaticSNP | 9 | 111693233 | C | A | intron | IKBKAP |  |  | Y |
| HCC-A1 | somaticSNP | 9 | 112172661 | G | C | missense,non-coding-exon | PTPN3 | PTPN3 |  | Y |
| HCC-A1 | somaticSNP | 9 | 113341468 | G | T | missense | SVEP1 | SVEP1 |  | Y |
| HCC-A1 | somaticSNP | 9 | 133554034 | G | C | intron-near-splice | PRDM12 |  |  | Y |
| HCC-A1 | somaticSNP | 9 | 139904603 | T | A | intron | ABCA2 |  |  | Y |
| HCC-A1 | somaticSNP | 10 | 16948243 | C | T | missense | CUBN | CUBN |  | Y |
| HCC-A1 | somaticSNP | 10 | 17085892 | T | C | missense | CUBN | CUBN |  | Y |
| HCC-A1 | somaticSNP | 10 | 27381387 | C | T | missense | ANKRD26 | ANKRD26 |  | Y |
| HCC-A1 | somaticSNP | 10 | 28023428 | A | T | synonymous | MKX | MKX |  | Y |
| HCC-A1 | somaticSNP | 10 | 29581055 | T | C | intron | LYZL1 |  |  | N |
| HCC-A1 | somaticSNP | 10 | 32326541 | A | T | intron-near-splice | KIF5B |  | KIF5B | Y |
| HCC-A1 | somaticINDEL | 10 | 60160134 | T | -AAA | intergenic |  | |  | N |
| HCC-A1 | somaticSNP | 10 | 75585233 | T | C | intron | CAMK2G |  |  | Y |
| HCC-A1 | somaticSNP | 10 | 82331347 | A | G | intron | SH2D4B |  |  | Y |
| HCC-A1 | somaticINDEL | 10 | 93776240 | C | +T | intron | BTAF1 |  |  | Y |
| HCC-A1 | somaticINDEL | 10 | 101978688 | T | -AAAG | intron | CHUK |  |  | Y |
| HCC-A1 | somaticSNP | 10 | 102744480 | C | T | 3-prime-UTR,intron,missense | MRPL43,SEMA4G | SEMA4G |  | Y |
| HCC-A1 | somaticSNP | 10 | 106039197 | G | A | intron,missense | GSTO2 | GSTO2 |  | Y |
| HCC-A1 | somaticSNP | 10 | 121287023 | C | T | intron | RGS10 |  |  | Y |
| HCC-A1 | somaticSNP | 10 | 125425914 | G | A | 5-prime-UTR | GPR26 | GPR26 |  | Y |
| HCC-A1 | somaticSNP | 10 | 127265198 | A | T | intron,3-prime-UTR | TEX36-AS1 |  |  | Y |
| HCC-A1 | somaticSNP | 10 | 129860116 | T | G | intron | PTPRE |  |  | Y |
| HCC-A1 | somaticSNP | 10 | 135368348 | C | T | intron,3-prime-UTR | SYCE1 | SYCE1 |  | Y |
| HCC-A1 | somaticSNP | 10 | 1405432 | G | C | missense | ADARB2 | ADARB2 |  | Y |
| HCC-A1 | somaticSNP | 10 | 116621277 | A | G | intron,missense | FAM160B1 | FAM160B1 |  | Y |
| HCC-A1 | somaticSNP | 11 | 125503061 | A | T | missense,non-coding-exon | CHEK1 | CHEK1 |  | Y |
| HCC-A1 | somaticSNP | 11 | 6412119 | A | G | synonymous | SMPD1 | SMPD1 |  | Y |
| HCC-A1 | somaticSNP | 11 | 8642032 | T | C | synonymous | TRIM66 | TRIM66 |  | Y |
| HCC-A1 | somaticSNP | 11 | 35747593 | C | T | missense | TRIM44 | TRIM44 |  | Y |
| HCC-A1 | somaticSNP | 11 | 57100642 | T | A | intron | SSRP1 |  |  | Y |
| HCC-A1 | somaticSNP | 11 | 61318960 | T | C | intron | SYT7 |  |  | Y |
| HCC-A1 | somaticSNP | 11 | 61912567 | A | C | intron-near-splice | INCENP |  |  | Y |
| HCC-A1 | somaticSNP | 11 | 64876028 | G | A | missense,non-coding-exon | VPS51 | VPS51 |  | Y |
| HCC-A1 | somaticSNP | 11 | 64939850 | T | C | intron | SPDYC |  |  | Y |
| HCC-A1 | somaticSNP | 11 | 73536698 | A | C | intron | MRPL48 |  |  | Y |
| HCC-A1 | somaticSNP | 11 | 77825342 | G | T | synonymous | ALG8,RNU6-83P | ALG8 |  | Y |
| HCC-A1 | somaticSNP | 11 | 101375549 | T | C | intron | TRPC6 |  |  | Y |
| HCC-A1 | somaticSNP | 11 | 106807392 | C | A | intron,missense | GUCY1A2 |  |  | Y |
| HCC-A1 | somaticSNP | 11 | 113239077 | A | T | intron-near-splice,intron,3-prime-UTR | TTC12 |  |  | Y |
| HCC-A1 | somaticSNP | 11 | 123847509 | A | G | missense | OR10S1 | OR10S1 |  | Y |
| HCC-A1 | somaticSNP | 11 | 130079615 | G | T | missense | ST14 | ST14 |  | Y |
| HCC-A1 | somaticSNP | 11 | 130731699 | T | A | intergenic |  | |  | Y |
| HCC-A1 | somaticSNP | 11 | 9075256 | T | C | intron,missense | SCUBE2 | SCUBE2 |  | Y |
| HCC-A1 | somaticSNP | 12 | 52882317 | C | T | missense | KRT6A | KRT6A |  | Y |
| HCC-A1 | somaticSNP | 12 | 104025383 | C | A | missense | STAB2 | STAB2 |  | Y |
| HCC-A1 | somaticSNP | 12 | 132502115 | A | C | missense | EP400 | EP400 |  | Y |
| HCC-A1 | somaticSNP | 12 | 8758100 | C | T | intron | AICDA |  |  | Y |
| HCC-A1 | somaticSNP | 12 | 10150721 | A | T | intron | CLEC1B |  |  | Y |
| HCC-A1 | somaticSNP | 12 | 21695584 | A | G | intron | GYS2 |  |  | N |
| HCC-A1 | somaticSNP | 12 | 29936449 | C | A | intron,missense | TMTC1 | TMTC1 |  | N |
| HCC-A1 | somaticSNP | 12 | 48370448 | C | T | intron | COL2A1 |  |  | Y |
| HCC-A1 | somaticSNP | 12 | 64488721 | C | A | missense | SRGAP1 | SRGAP1 |  | Y |
| HCC-A1 | somaticSNP | 12 | 72893286 | G | T | missense | TRHDE | TRHDE |  | Y |
| HCC-A1 | somaticSNP | 12 | 80732939 | A | T | missense | OTOGL | OTOGL |  | Y |
| HCC-A1 | somaticSNP | 12 | 93287162 | C | T | intron | EEA1 |  |  | Y |
| HCC-A1 | somaticSNP | 12 | 96932190 | T | A | missense | LOC101928871 | LOC101928871 |  | Y |
| HCC-A1 | somaticINDEL | 12 | 97311376 | C | -T | intron | NEDD1 |  |  | N |
| HCC-A1 | somaticSNP | 12 | 104174197 | C | T | intron | NT5DC3 |  |  | Y |
| HCC-A1 | somaticSNP | 13 | 20277413 | T | C | missense | PSPC1 | PSPC1 |  | N |
| HCC-A1 | somaticSNP | 13 | 26133834 | A | C | intron | ATP8A2 |  |  | Y |
| HCC-A1 | somaticSNP | 13 | 36424921 | A | G | intron,intron-near-splice | MIR548F5,DCLK1 |  |  | Y |
| HCC-A1 | somaticSNP | 13 | 37619503 | C | A | missense | SUPT20H | SUPT20H |  | Y |
| HCC-A1 | somaticSNP | 13 | 46104056 | G | A | intron-near-splice,intron | COG3 |  |  | Y |
| HCC-A1 | somaticSNP | 13 | 99540395 | A | G | intron | DOCK9 |  |  | N |
| HCC-A1 | somaticSNP | 13 | 26117437 | C | T | missense | ATP8A2 | ATP8A2 |  | Y |
| HCC-A1 | somaticSNP | 14 | 105613850 | T | A | missense | JAG2 | JAG2 |  | Y |
| HCC-A1 | somaticSNP | 14 | 22508913 | A | G | intergenic |  | |  | Y |
| HCC-A1 | somaticSNP | 14 | 22749767 | T | C | intergenic |  | |  | Y |
| HCC-A1 | somaticSNP | 14 | 23733725 | C | T | intron | C14orf164 |  |  | Y |
| HCC-A1 | somaticSNP | 14 | 23869882 | G | A | intron | MYH6 |  |  | Y |
| HCC-A1 | somaticINDEL | 14 | 30095782 | G | +A | intron | PRKD1,MIR548AI |  |  | N |
| HCC-A1 | somaticSNP | 14 | 37147839 | T | A | intron,3-prime-UTR | SLC25A21 | SLC25A21 |  | Y |
| HCC-A1 | somaticSNP | 14 | 61643154 | T | C | intergenic |  | |  | Y |
| HCC-A1 | somaticSNP | 14 | 70245100 | A | C | missense | SLC10A1 | SLC10A1 |  | Y |
| HCC-A1 | somaticSNP | 14 | 70826226 | C | G | intron | SYNJ2BP-COX16,COX16 |  |  | Y |
| HCC-A1 | somaticSNP | 14 | 72926278 | G | A | intron | RGS6 |  |  | Y |
| HCC-A1 | somaticSNP | 14 | 104199580 | A | C | 3-prime-UTR | ZFYVE21 | ZFYVE21 |  | N |
| HCC-A1 | somaticSNP | 15 | 23891689 | C | T | missense | MAGEL2 | MAGEL2 |  | Y |
| HCC-A1 | somaticSNP | 15 | 25449634 | A | T | upstream-gene |  | |  | Y |
| HCC-A1 | somaticSNP | 15 | 33893830 | T | A | intron | RYR3 |  |  | Y |
| HCC-A1 | somaticSNP | 15 | 33952558 | A | T | missense | RYR3 | RYR3 |  | Y |
| HCC-A1 | somaticSNP | 15 | 40846225 | G | A | intron,missense | C15orf57 | C15orf57 |  | Y |
| HCC-A1 | somaticINDEL | 15 | 41308142 | C | -A | intron | INO80 |  |  | N |
| HCC-A1 | somaticSNP | 15 | 43528016 | C | G | missense | TGM5 | TGM5 |  | Y |
| HCC-A1 | somaticSNP | 15 | 28375351 | T | C | missense | HERC2 | HERC2 |  | Y |
| HCC-A1 | somaticSNP | 15 | 43889786 | T | C | intron | RNU6-28P,CKMT1B |  |  | Y |
| HCC-A1 | somaticSNP | 15 | 48818257 | T | A | intron | FBN1 |  |  | Y |
| HCC-A1 | somaticSNP | 15 | 81572064 | A | T | missense | IL16 | IL16 |  | Y |
| HCC-A1 | somaticINDEL | 15 | 83241984 | A | -T | intron | CPEB1 |  |  | Y |
| HCC-A1 | somaticSNP | 15 | 79585665 | C | A | missense | ANKRD34C | ANKRD34C |  | Y |
| HCC-A1 | somaticSNP | 16 | 347179 | G | T | stop-gained | AXIN1 | AXIN1 | AXIN1 | Y |
| HCC-A1 | somaticSNP | 16 | 332316 | G | A | synonymous | ARHGDIG | ARHGDIG |  | Y |
| HCC-A1 | somaticSNP | 16 | 842643 | A | T | intron | CHTF18 |  |  | N |
| HCC-A1 | somaticSNP | 16 | 2821211 | T | C | 3-prime-UTR | SRRM2 | SRRM2 |  | Y |
| HCC-A1 | somaticSNP | 16 | 11556121 | A | G | synonymous | LOC400499 |  |  | N |
| HCC-A1 | somaticSNP | 16 | 15808975 | C | T | intron | NDE1,MYH11 |  | MYH11 | Y |
| HCC-A1 | somaticSNP | 16 | 30380445 | A | T | intron | TBC1D10B |  |  | Y |
| HCC-A1 | somaticSNP | 16 | 30941687 | A | G | synonymous,non-coding-exon | FBXL19 | FBXL19 |  | Y |
| HCC-A1 | somaticSNP | 16 | 46540233 | A | G | intron | ANKRD26P1 |  |  | Y |
| HCC-A1 | somaticSNP | 16 | 55617059 | T | A | 3-prime-UTR | LPCAT2 | LPCAT2 |  | Y |
| HCC-A1 | somaticSNP | 16 | 57168812 | C | A | intron | CPNE2 |  |  | Y |
| HCC-A1 | somaticSNP | 16 | 58568227 | C | T | missense,non-coding-exon | CNOT1 | CNOT1 |  | Y |
| HCC-A1 | somaticSNP | 16 | 71969447 | T | C | intron | PKD1L3 |  |  | Y |
| HCC-A1 | somaticSNP | 17 | 4719892 | G | T | intron | PLD2 |  |  | Y |
| HCC-A1 | somaticSNP | 17 | 8076698 | G | C | 3-prime-UTR | TMEM107 | TMEM107 |  | Y |
| HCC-A1 | somaticINDEL | 17 | 9142981 | C | -G | nonsynonymous,del,frameshift | NTN1 | NTN1 |  | Y |
| HCC-A1 | somaticSNP | 17 | 29161991 | G | A | missense | ATAD5 | ATAD5 |  | Y |
| HCC-A1 | somaticSNP | 17 | 29226536 | G | T | missense | TEFM | TEFM |  | Y |
| HCC-A1 | somaticSNP | 17 | 11671943 | G | T | missense-near-splice | DNAH9 | DNAH9 |  | Y |
| HCC-A1 | somaticSNP | 17 | 12852477 | C | T | synonymous | ARHGAP44 | ARHGAP44 |  | Y |
| HCC-A1 | somaticSNP | 17 | 16040527 | T | C | intron | NCOR1 |  |  | Y |
| HCC-A1 | somaticSNP | 17 | 27822745 | G | T | missense-near-splice | TAOK1 | TAOK1 |  | Y |
| HCC-A1 | somaticSNP | 17 | 38910804 | T | C | intron | KRT25 |  |  | Y |
| HCC-A1 | somaticSNP | 17 | 56429045 | C | A | intron | SUPT4H1,BZRAP1-AS1 |  |  | Y |
| HCC-A1 | somaticINDEL | 17 | 56693799 | C | +T | intron | TEX14 |  |  | N |
| HCC-A1 | somaticSNP | 17 | 57228448 | C | T | intron | SKA2 |  |  | Y |
| HCC-A1 | somaticSNP | 17 | 57886199 | C | A | missense | VMP1 | VMP1 |  | Y |
| HCC-A1 | somaticSNP | 17 | 71223299 | C | A | intron-near-splice | FAM104A |  |  | Y |
| HCC-A1 | somaticSNP | 17 | 74079838 | A | G | intron-near-splice | EXOC7 |  |  | Y |
| HCC-A1 | somaticSNP | 17 | 75834846 | C | T | intergenic |  | |  | Y |
| HCC-A1 | somaticSNP | 17 | 80129776 | T | C | intron | CCDC57 |  |  | Y |
| HCC-A1 | somaticSNP | 18 | 12067950 | C | T | intergenic |  | |  | Y |
| HCC-A1 | somaticSNP | 18 | 12103292 | T | G | intron | ANKRD62 |  |  | Y |
| HCC-A1 | somaticSNP | 18 | 74620187 | A | T | intron | ZNF236 |  |  | Y |
| HCC-A1 | somaticSNP | 19 | 9007560 | A | G | intron-near-splice | MUC16 |  |  | Y |
| HCC-A1 | somaticSNP | 19 | 10266472 | G | T | intron | DNMT1 |  |  | Y |
| HCC-A1 | somaticSNP | 19 | 15276007 | C | A | intron | NOTCH3 |  |  | Y |
| HCC-A1 | somaticSNP | 19 | 33359482 | C | T | 5-prime-UTR,intron | SLC7A9 | SLC7A9 |  | Y |
| HCC-A1 | somaticSNP | 19 | 34663663 | C | T | missense | LSM14A | LSM14A |  | Y |
| HCC-A1 | somaticSNP | 19 | 37368933 | A | T | stop-gained,intron | ZNF345 | ZNF345 |  | Y |
| HCC-A1 | somaticSNP | 19 | 34858156 | A | T | intron | GPI |  |  | Y |
| HCC-A1 | somaticSNP | 19 | 38692623 | C | T | synonymous | SIPA1L3 | SIPA1L3 |  | Y |
| HCC-A1 | somaticSNP | 19 | 39689787 | C | A | intron | NCCRP1 |  |  | Y |
| HCC-A1 | somaticSNP | 19 | 41916759 | G | A | intron | BCKDHA |  |  | Y |
| HCC-A1 | somaticSNP | 19 | 42814990 | G | A | upstream-gene |  | |  | Y |
| HCC-A1 | somaticSNP | 19 | 45147340 | G | T | 5-prime-UTR | PVR | PVR |  | N |
| HCC-A1 | somaticSNP | 19 | 47763712 | G | C | intron | CCDC9 |  |  | Y |
| HCC-A1 | somaticSNP | 19 | 49303120 | C | A | intron | BCAT2 |  |  | Y |
| HCC-A1 | somaticSNP | 19 | 50250504 | C | A | 5-prime-UTR,intron | TSKS |  |  | Y |
| HCC-A1 | somaticSNP | 19 | 50266498 | T | A | missense | TSKS | TSKS |  | Y |
| HCC-A1 | somaticSNP | 19 | 50463727 | G | A | intron | SIGLEC11 |  |  | N |
| HCC-A1 | somaticSNP | 19 | 51919279 | A | G | intron,synonymous,missense | SIGLEC10,LOC100129083 | SIGLEC10,LOC100129083 |  | Y |
| HCC-A1 | somaticSNP | 19 | 51919302 | T | C | intron,synonymous,missense | SIGLEC10,LOC100129083 | SIGLEC10,LOC100129083 |  | N |
| HCC-A1 | somaticSNP | 19 | 56436029 | A | C | intron-near-splice | NLRP13 |  |  | Y |
| HCC-A1 | somaticSNP | 20 | 3219894 | A | G | intron | SLC4A11 |  |  | N |
| HCC-A1 | somaticSNP | 20 | 9449200 | C | T | intron | PLCB4 |  |  | N |
| HCC-A1 | somaticSNP | 20 | 31887621 | A | T | intron | BPIFB1 |  |  | Y |
| HCC-A1 | somaticSNP | 20 | 32868834 | T | A | 3-prime-UTR | AHCY | AHCY |  | Y |
| HCC-A1 | somaticSNP | 20 | 34204981 | C | T | intron | SPAG4 |  |  | Y |
| HCC-A1 | somaticINDEL | 20 | 43249004 | A | -TCT | nonsynonymous,del,frameshift | ADA | ADA |  | Y |
| HCC-A1 | somaticINDEL | 20 | 46281139 | A | -T | intron | NCOA3 |  |  | Y |
| HCC-A1 | somaticSNP | 20 | 46295240 | C | T | intron-near-splice | SULF2 |  |  | Y |
| HCC-A1 | somaticSNP | 20 | 49202537 | G | C | downstream-gene |  | |  | Y |
| HCC-A1 | somaticSNP | 20 | 49225000 | C | A | synonymous | FAM65C | FAM65C |  | Y |
| HCC-A1 | somaticSNP | 20 | 55908207 | T | C | intron | SPO11 |  |  | Y |
| HCC-A1 | somaticSNP | 20 | 58425592 | T | C | downstream-gene |  | |  | Y |
| HCC-A1 | somaticSNP | 20 | 6060118 | T | C | missense | FERMT1 | FERMT1 |  | Y |
| HCC-A1 | somaticSNP | 20 | 37529300 | G | A | missense | PPP1R16B | PPP1R16B |  | Y |
| HCC-A1 | somaticSNP | 20 | 55803351 | C | T | missense | BMP7 | BMP7 |  | Y |
| HCC-A1 | somaticSNP | 21 | 41719719 | G | T | missense,non-coding-exon | DSCAM | DSCAM |  | Y |
| HCC-A1 | somaticSNP | 21 | 16340367 | C | A | missense | NRIP1 | NRIP1 |  | Y |
| HCC-A1 | somaticSNP | 21 | 19642346 | G | A | synonymous | TMPRSS15 | TMPRSS15 |  | Y |
| HCC-A1 | somaticSNP | 21 | 31798199 | G | C | missense | KRTAP13-3 | KRTAP13-3 |  | Y |
| HCC-A1 | somaticSNP | 21 | 43541206 | A | C | missense | UMODL1 | UMODL1 |  | Y |
| HCC-A1 | somaticSNP | 21 | 45706413 | A | T | intron | AIRE |  |  | Y |
| HCC-A1 | somaticSNP | 22 | 25603127 | G | A | missense | CRYBB3 | CRYBB3 |  | Y |
| HCC-A1 | somaticSNP | 22 | 31335601 | C | A | intron-near-splice | MORC2 |  |  | Y |
| HCC-A1 | somaticSNP | 22 | 36690459 | T | C | intron | MYH9 |  | MYH9 | Y |
| HCC-A1 | somaticSNP | 22 | 38622957 | C | A | intron | TMEM184B |  |  | Y |
| HCC-A1 | somaticSNP | 22 | 42415519 | A | G | intron | WBP2NL |  |  | Y |
| HCC-A1 | somaticSNP | 22 | 44594723 | T | C | intron | PARVG |  |  | Y |
| HCC-A1 | somaticSNP | X | 27998336 | G | C | missense | DCAF8L1 | DCAF8L1 |  | Y |
| HCC-A1 | somaticSNP | X | 53250052 | G | A | 5-prime-UTR,intron,missense | KDM5C | KDM5C | KDM5C | Y |
| HCC-A1 | somaticSNP | X | 117815120 | A | G | missense | DOCK11 | DOCK11 |  | Y |
| HCC-A1 | somaticSNP | X | 118921327 | T | C | intron,non-coding-exon | SNORA69,RPL39 | SNORA69 |  | Y |
| HCC-A1 | somaticSNP | X | 49851018 | C | T | missense,non-coding-exon | CLCN5 | CLCN5 |  | Y |
| HCC-A1 | somaticSNP | X | 50053087 | T | A | intron,missense | CCNB3 | CCNB3 |  | Y |
| HCC-A1 | somaticSNP | X | 125686072 | G | T | missense | DCAF12L1 | DCAF12L1 |  | Y |
| HCC-A1 | somaticSNP | X | 153236100 | C | T | synonymous-near-splice | HCFC1 | HCFC1 |  | Y |
| HCC-A1 | somaticSNP | Y | 14532150 | G | A | non-coding-exon | GYG2P1 | GYG2P1 |  | Y |
| HCC-A2 | somaticSNP | 1 | 15855461 | T | G | intron | DNAJC16 |  |  | Y |
| HCC-A2 | somaticSNP | 1 | 17023483 | C | T | intron | ESPNP |  |  | Y |
| HCC-A2 | somaticSNP | 1 | 17267496 | C | T | intron | CROCC |  |  | N |
| HCC-A2 | somaticSNP | 1 | 19062153 | G | A | missense | PAX7 | PAX7 | PAX7 | Y |
| HCC-A2 | somaticSNP | 1 | 22158261 | T | C | missense | HSPG2 | HSPG2 |  | Y |
| HCC-A2 | somaticINDEL | 1 | 25780889 | A | +TG | intron | TMEM57 |  |  | N |
| HCC-A2 | somaticSNP | 1 | 26738094 | T | G | intron | LIN28A |  |  | Y |
| HCC-A2 | somaticSNP | 1 | 26873700 | C | T | 5-prime-UTR,synonymous | RPS6KA1 | RPS6KA1 |  | N |
| HCC-A2 | somaticSNP | 1 | 29611123 | C | T | intron | PTPRU |  |  | N |
| HCC-A2 | somaticSNP | 1 | 36939412 | T | G | synonymous | CSF3R | CSF3R | CSF3R | Y |
| HCC-A2 | somaticSNP | 1 | 41327871 | C | A | 5-prime-UTR | CITED4 | CITED4 |  | N |
| HCC-A2 | somaticSNP | 1 | 53279594 | A | G | intron | ZYG11B |  |  | Y |
| HCC-A2 | somaticSNP | 1 | 62503632 | A | G | intron | INADL |  |  | Y |
| HCC-A2 | somaticSNP | 1 | 78267110 | G | A | missense | FAM73A | FAM73A |  | Y |
| HCC-A2 | somaticSNP | 1 | 86814427 | G | T | 3-prime-UTR | ODF2L | ODF2L |  | Y |
| HCC-A2 | somaticINDEL | 1 | 100680593 | C | +T | intron | DBT |  |  | Y |
| HCC-A2 | somaticSNP | 1 | 103385782 | T | C | intron | COL11A1 |  |  | Y |
| HCC-A2 | somaticSNP | 1 | 110129627 | G | T | intron | GNAI3 |  |  | Y |
| HCC-A2 | somaticSNP | 1 | 120306816 | T | C | missense | HMGCS2 | HMGCS2 |  | Y |
| HCC-A2 | somaticSNP | 1 | 145281824 | T | G | intron | NBPF20,NBPF9,NOTCH2NL |  |  | Y |
| HCC-A2 | somaticSNP | 1 | 153642826 | C | T | intron | ILF2 |  |  | Y |
| HCC-A2 | somaticSNP | 1 | 156845864 | C | A | intron-near-splice,synonymous-near-splice | NTRK1 |  | NTRK1 | Y |
| HCC-A2 | somaticSNP | 1 | 157514235 | C | G | missense | FCRL5 | FCRL5 |  | Y |
| HCC-A2 | somaticSNP | 1 | 158435706 | G | T | missense | OR10K1 | OR10K1 |  | Y |
| HCC-A2 | somaticSNP | 1 | 158687102 | A | G | synonymous | OR6K3 | OR6K3 |  | Y |
| HCC-A2 | somaticSNP | 1 | 165180036 | T | C | intron | LMX1A |  |  | Y |
| HCC-A2 | somaticSNP | 1 | 166959161 | T | C | intron | MAEL |  |  | Y |
| HCC-A2 | somaticSNP | 1 | 168250404 | G | A | missense | TBX19 | TBX19 |  | Y |
| HCC-A2 | somaticSNP | 1 | 181732615 | G | A | missense | CACNA1E | CACNA1E |  | Y |
| HCC-A2 | somaticSNP | 1 | 186122844 | A | G | intron | HMCN1,MIR548F1 |  |  | Y |
| HCC-A2 | somaticSNP | 1 | 205034434 | G | T | intron | CNTN2 |  |  | Y |
| HCC-A2 | somaticSNP | 1 | 227227740 | A | C | intron | CDC42BPA |  |  | Y |
| HCC-A2 | somaticSNP | 1 | 227922773 | G | C | 5-prime-UTR,missense | SNAP47,JMJD4 | SNAP47,JMJD4 |  | Y |
| HCC-A2 | somaticSNP | 1 | 228509727 | T | A | missense | OBSCN | OBSCN |  | Y |
| HCC-A2 | somaticSNP | 1 | 237773958 | A | T | intron | RYR2 |  |  | Y |
| HCC-A2 | somaticSNP | 1 | 247921652 | G | A | intron,synonymous | OR1C1 | OR1C1 |  | Y |
| HCC-A2 | somaticSNP | 2 | 21239443 | G | T | missense | APOB | APOB |  | Y |
| HCC-A2 | somaticSNP | 2 | 31412096 | T | C | intron | CAPN14 |  |  | N |
| HCC-A2 | somaticSNP | 2 | 54891911 | A | T | intron | SPTBN1 |  |  | Y |
| HCC-A2 | somaticSNP | 2 | 55433542 | T | C | intron | CLHC1 |  |  | Y |
| HCC-A2 | somaticSNP | 2 | 61020681 | A | T | intron | PAPOLG |  |  | Y |
| HCC-A2 | somaticSNP | 2 | 61697887 | C | G | upstream-gene |  | |  | N |
| HCC-A2 | somaticSNP | 2 | 71635939 | C | G | intron | ZNF638 |  |  | Y |
| HCC-A2 | somaticSNP | 2 | 98833151 | T | G | intron | VWA3B |  |  | Y |
| HCC-A2 | somaticSNP | 2 | 116447348 | A | G | intron | DPP10 |  |  | Y |
| HCC-A2 | somaticSNP | 2 | 118701541 | T | C | intron | CCDC93 |  |  | Y |
| HCC-A2 | somaticSNP | 2 | 128522167 | G | A | intron,synonymous | WDR33 | WDR33 |  | Y |
| HCC-A2 | somaticSNP | 2 | 133014556 | C | T | intron,non-coding-exon | MIR663B,ANKRD30BL | MIR663B |  | N |
| HCC-A2 | somaticSNP | 2 | 151331371 | T | C | intron | RND3 |  |  | N |
| HCC-A2 | somaticSNP | 2 | 153551068 | C | T | synonymous | PRPF40A | PRPF40A |  | Y |
| HCC-A2 | somaticSNP | 2 | 153574008 | C | A | 5-prime-UTR,missense | PRPF40A | PRPF40A |  | Y |
| HCC-A2 | somaticSNP | 2 | 166908325 | G | T | missense | SCN1A | SCN1A |  | Y |
| HCC-A2 | somaticSNP | 2 | 176857148 | T | A | splice-3 | KIAA1715 |  |  | N |
| HCC-A2 | somaticSNP | 2 | 177034400 | G | A | intron | HOXD3 |  |  | Y |
| HCC-A2 | somaticSNP | 2 | 197712586 | G | T | intron | PGAP1 |  |  | N |
| HCC-A2 | somaticSNP | 2 | 201756675 | A | G | intron,synonymous | NIF3L1 | NIF3L1 |  | Y |
| HCC-A2 | somaticSNP | 2 | 210877639 | A | T | intron | RPE |  |  | Y |
| HCC-A2 | somaticSNP | 2 | 219558631 | A | G | missense | STK36 | STK36 |  | N |
| HCC-A2 | somaticSNP | 2 | 220358383 | G | T | upstream-gene |  | |  | Y |
| HCC-A2 | somaticSNP | 2 | 231152571 | A | T | intron | SP140 |  |  | Y |
| HCC-A2 | somaticSNP | 2 | 236708207 | A | G | intron | AGAP1 |  |  | Y |
| HCC-A2 | somaticSNP | 2 | 242800900 | G | A | intron | PDCD1 |  |  | N |
| HCC-A2 | somaticINDEL | 3 | 19389266 | C | +A | nonsynonymous,ins,frameshift | KCNH8 | KCNH8 |  | Y |
| HCC-A2 | somaticSNP | 3 | 38051009 | C | T | intron | PLCD1 |  |  | N |
| HCC-A2 | somaticSNP | 3 | 39448229 | A | G | 5-prime-UTR | RPSA | RPSA |  | Y |
| HCC-A2 | somaticSNP | 3 | 44283610 | T | A | missense | TOPAZ1 | TOPAZ1 |  | Y |
| HCC-A2 | somaticSNP | 3 | 48594104 | C | G | intron | PFKFB4 |  |  | Y |
| HCC-A2 | somaticINDEL | 3 | 57108374 | T | -C | nonsynonymous,del,frameshift | SPATA12,ARHGEF3 | SPATA12 |  | Y |
| HCC-A2 | somaticSNP | 3 | 69112049 | C | T | intron | UBA3 |  |  | Y |
| HCC-A2 | somaticSNP | 3 | 97464291 | T | A | intron | EPHA6 |  |  | Y |
| HCC-A2 | somaticSNP | 3 | 100084408 | T | C | synonymous | TOMM70A | TOMM70A |  | Y |
| HCC-A2 | somaticSNP | 3 | 121132268 | T | A | intron | STXBP5L |  |  | N |
| HCC-A2 | somaticSNP | 3 | 124449461 | G | A | 5-prime-UTR,missense,non-coding-exon | UMPS | UMPS |  | Y |
| HCC-A2 | somaticSNP | 3 | 133877257 | C | T | intron | RYK |  |  | N |
| HCC-A2 | somaticSNP | 3 | 142840069 | T | A | synonymous | CHST2 | CHST2 |  | Y |
| HCC-A2 | somaticINDEL | 3 | 150282061 | C | -T | intron | EIF2A |  |  | N |
| HCC-A2 | somaticSNP | 3 | 171965580 | C | T | intron | FNDC3B |  |  | N |
| HCC-A2 | somaticSNP | 3 | 184299095 | A | C | synonymous | EPHB3 | EPHB3 |  | Y |
| HCC-A2 | somaticSNP | 3 | 185879581 | T | A | intron | DGKG |  |  | Y |
| HCC-A2 | somaticSNP | 3 | 190995829 | A | T | intron | UTS2B |  |  | Y |
| HCC-A2 | somaticSNP | 3 | 193855537 | G | T | stop-gained | HES1 | HES1 |  | Y |
| HCC-A2 | somaticSNP | 3 | 194118705 | G | A | synonymous | GP5 | GP5 |  | Y |
| HCC-A2 | somaticSNP | 4 | 2900240 | G | A | synonymous | ADD1 | ADD1 |  | Y |
| HCC-A2 | somaticSNP | 4 | 10020564 | G | A | intron | SLC2A9 |  |  | N |
| HCC-A2 | somaticINDEL | 4 | 26241366 | G | +GGAGA | intron | RBPJ |  |  | Y |
| HCC-A2 | somaticINDEL | 4 | 74281947 | A | -TT | intron | ALB |  |  | Y |
| HCC-A2 | somaticSNP | 4 | 90034354 | G | C | missense | TIGD2 | TIGD2 |  | Y |
| HCC-A2 | somaticSNP | 4 | 103556316 | T | C | intron | MANBA |  |  | N |
| HCC-A2 | somaticINDEL | 4 | 106317370 | T | +ATAAA | intron | PPA2 |  |  | Y |
| HCC-A2 | somaticSNP | 4 | 122068167 | T | C | intron | TNIP3 |  |  | Y |
| HCC-A2 | somaticSNP | 4 | 125631753 | T | C | 5-prime-UTR,intron | ANKRD50 | ANKRD50 |  | Y |
| HCC-A2 | somaticSNP | 4 | 155533047 | A | G | missense | FGG | FGG |  | Y |
| HCC-A2 | somaticSNP | 5 | 172110938 | G | A | intron,missense | NEURL1B | NEURL1B |  | Y |
| HCC-A2 | somaticSNP | 6 | 99848727 | T | C | missense | PNISR | PNISR |  | Y |
| HCC-A2 | somaticSNP | 5 | 7414718 | A | G | synonymous | ADCY2 | ADCY2 |  | Y |
| HCC-A2 | somaticSNP | 5 | 76714070 | T | A | stop-gained | PDE8B | PDE8B |  | Y |
| HCC-A2 | somaticINDEL | 5 | 78416973 | C | -A | intron | BHMT |  |  | N |
| HCC-A2 | somaticSNP | 5 | 135396468 | T | A | intron | TGFBI |  |  | N |
| HCC-A2 | somaticSNP | 5 | 145531578 | G | A | intron | LARS |  |  | Y |
| HCC-A2 | somaticSNP | 5 | 149112300 | T | G | intron | PPARGC1B |  |  | N |
| HCC-A2 | somaticSNP | 5 | 169309494 | T | A | intron | DOCK2,FAM196B |  |  | Y |
| HCC-A2 | somaticSNP | 5 | 169412889 | C | T | missense | DOCK2 | DOCK2 |  | Y |
| HCC-A2 | somaticSNP | 5 | 178506468 | G | T | missense | ZNF354C | ZNF354C |  | Y |
| HCC-A2 | somaticSNP | 6 | 5000779 | G | A | intron | RPP40 |  |  | N |
| HCC-A2 | somaticSNP | 6 | 28197605 | C | T | intron | ZSCAN9 |  |  | Y |
| HCC-A2 | somaticSNP | 6 | 34029881 | C | T | intron | GRM4 |  |  | Y |
| HCC-A2 | somaticSNP | 6 | 71233675 | A | G | missense | FAM135A | FAM135A |  | Y |
| HCC-A2 | somaticSNP | 6 | 75801024 | G | T | intron | COL12A1 |  |  | Y |
| HCC-A2 | somaticSNP | 6 | 76720848 | T | A | intron | IMPG1 |  |  | N |
| HCC-A2 | somaticSNP | 6 | 108831414 | G | A | splice-acceptor | LACE1 | LACE1 |  | Y |
| HCC-A2 | somaticSNP | 6 | 111329423 | G | T | intron | RPF2 |  |  | Y |
| HCC-A2 | somaticSNP | 6 | 129799721 | T | C | intron | LAMA2 |  |  | Y |
| HCC-A2 | somaticSNP | 6 | 132874297 | T | A | missense | TAAR8 | TAAR8 |  | Y |
| HCC-A2 | somaticSNP | 6 | 144508629 | C | A | 3-prime-UTR | STX11 | STX11 |  | Y |
| HCC-A2 | somaticSNP | 6 | 149771895 | T | A | missense | ZC3H12D | ZC3H12D |  | Y |
| HCC-A2 | somaticSNP | 6 | 167426949 | A | G | intron | FGFR1OP |  | FGFR1OP | Y |
| HCC-A2 | somaticSNP | 6 | 167446139 | A | G | intron | FGFR1OP |  | FGFR1OP | Y |
| HCC-A2 | somaticSNP | 6 | 170034675 | A | T | intron | WDR27 |  |  | Y |
| HCC-A2 | somaticSNP | 6 | 170049362 | C | A | missense | WDR27 | WDR27 |  | Y |
| HCC-A2 | somaticSNP | 7 | 330562 | G | A | intron | WI2-2373I1.2 |  |  | Y |
| HCC-A2 | somaticSNP | 7 | 1914350 | A | G | intron | MAD1L1 |  |  | Y |
| HCC-A2 | somaticSNP | 7 | 12409830 | T | C | missense | VWDE | VWDE |  | Y |
| HCC-A2 | somaticSNP | 7 | 12610475 | C | T | synonymous | SCIN | SCIN |  | Y |
| HCC-A2 | somaticSNP | 7 | 19156335 | G | T | 3-prime-UTR | TWIST1 | TWIST1 |  | Y |
| HCC-A2 | somaticINDEL | 7 | 23296415 | G | +A | intron | GPNMB |  |  | Y |
| HCC-A2 | somaticSNP | 7 | 26404329 | T | C | intron | SNX10 |  |  | Y |
| HCC-A2 | somaticSNP | 7 | 44619235 | G | A | intron-near-splice | TMED4 |  |  | Y |
| HCC-A2 | somaticSNP | 7 | 48146967 | G | A | missense | UPP1 | UPP1 |  | Y |
| HCC-A2 | somaticSNP | 7 | 63726690 | C | A | missense | ZNF679 | ZNF679 |  | Y |
| HCC-A2 | somaticSNP | 7 | 80374631 | A | G | intron-near-splice | SEMA3C |  |  | Y |
| HCC-A2 | somaticSNP | 7 | 83035341 | T | G | missense | SEMA3E | SEMA3E |  | Y |
| HCC-A2 | somaticSNP | 7 | 99578381 | T | C | upstream-gene |  | |  | N |
| HCC-A2 | somaticSNP | 7 | 99578411 | T | C | non-coding-exon | AZGP1P1 | AZGP1P1 |  | Y |
| HCC-A2 | somaticSNP | 7 | 107332509 | G | A | intron,missense | SLC26A4 |  |  | Y |
| HCC-A2 | somaticSNP | 7 | 131982688 | C | A | intron | PLXNA4 |  |  | N |
| HCC-A2 | somaticSNP | 7 | 142045484 | C | A | intergenic |  | |  | Y |
| HCC-A2 | somaticSNP | 7 | 142561350 | G | T | intron | EPHB6 |  |  | Y |
| HCC-A2 | somaticSNP | 7 | 143095395 | C | T | intron | EPHA1 |  |  | Y |
| HCC-A2 | somaticSNP | 7 | 143573132 | C | T | intron,synonymous | FAM115A | FAM115A |  | Y |
| HCC-A2 | somaticSNP | 8 | 35579196 | T | C | intron | UNC5D |  |  | Y |
| HCC-A2 | somaticSNP | 8 | 39103668 | C | G | missense | ADAM32 | ADAM32 |  | Y |
| HCC-A2 | somaticSNP | 8 | 39862909 | T | C | intron | IDO2 |  |  | N |
| HCC-A2 | somaticSNP | 8 | 69013781 | T | A | intron | PREX2 |  |  | Y |
| HCC-A2 | somaticSNP | 8 | 69351923 | A | C | intron | C8orf34 |  |  | N |
| HCC-A2 | somaticSNP | 8 | 73150337 | A | T | non-coding-exon | LOC392232 | LOC392232 |  | Y |
| HCC-A2 | somaticSNP | 8 | 73479912 | T | A | 5-prime-UTR | KCNB2 | KCNB2 |  | Y |
| HCC-A2 | somaticSNP | 8 | 104897740 | A | G | missense | RIMS2 | RIMS2 |  | Y |
| HCC-A2 | somaticSNP | 8 | 113418760 | A | T | synonymous | CSMD3 | CSMD3 |  | Y |
| HCC-A2 | somaticSNP | 8 | 125047646 | C | T | intron,synonymous | FER1L6,FER1L6-AS1 | FER1L6 |  | Y |
| HCC-A2 | somaticSNP | 8 | 131964000 | C | A | intron | ADCY8 |  |  | N |
| HCC-A2 | somaticSNP | 8 | 141712834 | T | C | intron | PTK2 |  |  | Y |
| HCC-A2 | somaticSNP | 8 | 143614849 | G | T | intron | BAI1 |  |  | Y |
| HCC-A2 | somaticSNP | 8 | 146220171 | T | C | intron | ZNF252P |  |  | Y |
| HCC-A2 | somaticSNP | 9 | 14784688 | A | G | intron | FREM1 |  |  | Y |
| HCC-A2 | somaticSNP | 9 | 84202674 | C | G | synonymous | TLE1 | TLE1 |  | Y |
| HCC-A2 | somaticSNP | 9 | 91360763 | G | A | non-coding-exon | MIR4289 | MIR4289 |  | Y |
| HCC-A2 | somaticSNP | 9 | 98766997 | G | T | intron | ERCC6L2 |  |  | Y |
| HCC-A2 | somaticSNP | 9 | 99314276 | G | C | intron | CDC14B |  |  | Y |
| HCC-A2 | somaticSNP | 9 | 105763940 | T | C | intron | CYLC2 |  |  | Y |
| HCC-A2 | somaticSNP | 9 | 111693233 | C | A | intron | IKBKAP |  |  | Y |
| HCC-A2 | somaticSNP | 9 | 112141909 | T | C | missense,non-coding-exon | PTPN3 | PTPN3 |  | Y |
| HCC-A2 | somaticSNP | 9 | 113341468 | G | T | missense | SVEP1 | SVEP1 |  | Y |
| HCC-A2 | somaticSNP | 9 | 133554034 | G | C | intron-near-splice | PRDM12 |  |  | Y |
| HCC-A2 | somaticSNP | 9 | 139904603 | T | A | intron | ABCA2 |  |  | Y |
| HCC-A2 | somaticSNP | 10 | 28023428 | A | T | synonymous | MKX | MKX |  | Y |
| HCC-A2 | somaticSNP | 10 | 32326541 | A | T | intron-near-splice | KIF5B |  | KIF5B | Y |
| HCC-A2 | somaticINDEL | 10 | 60160134 | T | -AAA | intergenic |  | |  | N |
| HCC-A2 | somaticSNP | 10 | 75585233 | T | C | intron | CAMK2G |  |  | Y |
| HCC-A2 | somaticSNP | 10 | 82331347 | A | G | intron | SH2D4B |  |  | Y |
| HCC-A2 | somaticINDEL | 10 | 93776240 | C | +T | intron | BTAF1 |  |  | N |
| HCC-A2 | somaticINDEL | 10 | 101978688 | T | -AAAG | intron | CHUK |  |  | Y |
| HCC-A2 | somaticSNP | 10 | 102744480 | C | T | 3-prime-UTR,intron,missense | MRPL43,SEMA4G | SEMA4G |  | Y |
| HCC-A2 | somaticSNP | 10 | 106039197 | G | A | intron,missense | GSTO2 | GSTO2 |  | Y |
| HCC-A2 | somaticSNP | 10 | 121287023 | C | T | intron | RGS10 |  |  | Y |
| HCC-A2 | somaticSNP | 10 | 125425914 | G | A | 5-prime-UTR | GPR26 | GPR26 |  | Y |
| HCC-A2 | somaticSNP | 10 | 127265198 | A | T | intron,3-prime-UTR | TEX36-AS1 |  |  | Y |
| HCC-A2 | somaticSNP | 10 | 135368348 | C | T | intron,3-prime-UTR | SYCE1 | SYCE1 |  | N |
| HCC-A2 | somaticSNP | 10 | 1405432 | G | C | missense | ADARB2 | ADARB2 |  | Y |
| HCC-A2 | somaticSNP | 10 | 16948243 | C | T | missense | CUBN | CUBN |  | Y |
| HCC-A2 | somaticSNP | 10 | 17085892 | T | C | missense | CUBN | CUBN |  | Y |
| HCC-A2 | somaticSNP | 10 | 27381387 | C | T | missense | ANKRD26 | ANKRD26 |  | Y |
| HCC-A2 | somaticSNP | 10 | 116621277 | A | G | intron,missense | FAM160B1 | FAM160B1 |  | Y |
| HCC-A2 | somaticSNP | 11 | 35747593 | C | T | missense | TRIM44 | TRIM44 |  | Y |
| HCC-A2 | somaticSNP | 11 | 64876028 | G | A | missense,non-coding-exon | VPS51 | VPS51 |  | Y |
| HCC-A2 | somaticSNP | 11 | 106807392 | C | A | intron,missense | GUCY1A2 |  |  | Y |
| HCC-A2 | somaticSNP | 11 | 125503061 | A | T | missense,non-coding-exon | CHEK1 | CHEK1 |  | Y |
| HCC-A2 | somaticSNP | 11 | 6412119 | A | G | synonymous | SMPD1 | SMPD1 |  | Y |
| HCC-A2 | somaticSNP | 11 | 8642032 | T | C | synonymous | TRIM66 | TRIM66 |  | Y |
| HCC-A2 | somaticSNP | 11 | 26563468 | A | T | intron | ANO3 |  |  | Y |
| HCC-A2 | somaticSNP | 11 | 57100642 | T | A | intron | SSRP1 |  |  | Y |
| HCC-A2 | somaticSNP | 11 | 61318960 | T | C | intron | SYT7 |  |  | Y |
| HCC-A2 | somaticSNP | 11 | 61912567 | A | C | intron-near-splice | INCENP |  |  | Y |
| HCC-A2 | somaticSNP | 11 | 64939850 | T | C | intron | SPDYC |  |  | Y |
| HCC-A2 | somaticSNP | 11 | 73536698 | A | C | intron | MRPL48 |  |  | N |
| HCC-A2 | somaticSNP | 11 | 77825342 | G | T | synonymous | ALG8,RNU6-83P | ALG8 |  | Y |
| HCC-A2 | somaticSNP | 11 | 85685824 | G | A | intron,missense | PICALM | PICALM | PICALM | Y |
| HCC-A2 | somaticSNP | 11 | 86666063 | C | T | missense | FZD4 | FZD4 |  | Y |
| HCC-A2 | somaticSNP | 11 | 89429907 | A | G | intron | FOLH1B |  |  | Y |
| HCC-A2 | somaticSNP | 11 | 101375549 | T | C | intron | TRPC6 |  |  | N |
| HCC-A2 | somaticSNP | 11 | 113239077 | A | T | intron-near-splice,intron,3-prime-UTR | TTC12 |  |  | Y |
| HCC-A2 | somaticSNP | 11 | 118499010 | C | T | missense | PHLDB1 | PHLDB1 |  | Y |
| HCC-A2 | somaticSNP | 11 | 130079615 | G | T | missense | ST14 | ST14 |  | Y |
| HCC-A2 | somaticSNP | 11 | 130731699 | T | A | intergenic |  | |  | Y |
| HCC-A2 | somaticSNP | 12 | 8758100 | C | T | intron | AICDA |  |  | Y |
| HCC-A2 | somaticSNP | 12 | 10150721 | A | T | intron | CLEC1B |  |  | Y |
| HCC-A2 | somaticSNP | 12 | 21695584 | A | G | intron | GYS2 |  |  | N |
| HCC-A2 | somaticSNP | 12 | 25101801 | T | C | intron | BCAT1 |  |  | N |
| HCC-A2 | somaticSNP | 12 | 48370448 | C | T | intron | COL2A1 |  |  | Y |
| HCC-A2 | somaticSNP | 12 | 72893286 | G | T | missense | TRHDE | TRHDE |  | Y |
| HCC-A2 | somaticSNP | 12 | 93287162 | C | T | intron | EEA1 |  |  | Y |
| HCC-A2 | somaticSNP | 12 | 96932190 | T | A | missense | LOC101928871 | LOC101928871 |  | Y |
| HCC-A2 | somaticINDEL | 12 | 97311376 | C | -T | intron | NEDD1 |  |  | N |
| HCC-A2 | somaticSNP | 12 | 104025383 | C | A | missense | STAB2 | STAB2 |  | Y |
| HCC-A2 | somaticSNP | 12 | 132502115 | A | C | missense | EP400 | EP400 |  | Y |
| HCC-A2 | somaticSNP | 13 | 26133834 | A | C | intron | ATP8A2 |  |  | Y |
| HCC-A2 | somaticSNP | 13 | 36424921 | A | G | intron,intron-near-splice | MIR548F5,DCLK1 |  |  | Y |
| HCC-A2 | somaticSNP | 13 | 46104056 | G | A | intron-near-splice,intron | COG3 |  |  | Y |
| HCC-A2 | somaticSNP | 13 | 99540395 | A | G | intron | DOCK9 |  |  | N |
| HCC-A2 | somaticSNP | 14 | 22508913 | A | G | intergenic |  | |  | Y |
| HCC-A2 | somaticSNP | 14 | 22749767 | T | C | intergenic |  | |  | Y |
| HCC-A2 | somaticSNP | 14 | 23733725 | C | T | intron | C14orf164 |  |  | Y |
| HCC-A2 | somaticSNP | 14 | 23869882 | G | A | intron | MYH6 |  |  | Y |
| HCC-A2 | somaticINDEL | 14 | 30095782 | G | +A | intron | PRKD1,MIR548AI |  |  | Y |
| HCC-A2 | somaticSNP | 14 | 37147839 | T | A | intron,3-prime-UTR | SLC25A21 | SLC25A21 |  | Y |
| HCC-A2 | somaticSNP | 14 | 61643154 | T | C | intergenic |  | |  | N |
| HCC-A2 | somaticSNP | 14 | 70245100 | A | C | missense | SLC10A1 | SLC10A1 |  | Y |
| HCC-A2 | somaticSNP | 14 | 70826226 | C | G | intron | SYNJ2BP-COX16,COX16 |  |  | Y |
| HCC-A2 | somaticSNP | 14 | 72926278 | G | A | intron | RGS6 |  |  | Y |
| HCC-A2 | somaticSNP | 14 | 86088238 | C | T | missense | FLRT2 | FLRT2 |  | Y |
| HCC-A2 | somaticSNP | 14 | 104199580 | A | C | 3-prime-UTR | ZFYVE21 | ZFYVE21 |  | Y |
| HCC-A2 | somaticSNP | 15 | 25449634 | A | T | upstream-gene |  | |  | Y |
| HCC-A2 | somaticSNP | 15 | 28375351 | T | C | missense | HERC2 | HERC2 |  | Y |
| HCC-A2 | somaticSNP | 15 | 33893830 | T | A | intron | RYR3 |  |  | Y |
| HCC-A2 | somaticSNP | 15 | 33952558 | A | T | missense | RYR3 | RYR3 |  | N |
| HCC-A2 | somaticSNP | 15 | 40846225 | G | A | intron,missense | C15orf57 | C15orf57 |  | Y |
| HCC-A2 | somaticINDEL | 15 | 41308142 | C | -A | intron | INO80 |  |  | N |
| HCC-A2 | somaticSNP | 15 | 43889786 | T | C | intron | RNU6-28P,CKMT1B |  |  | Y |
| HCC-A2 | somaticSNP | 15 | 48818257 | T | A | intron | FBN1 |  |  | Y |
| HCC-A2 | somaticSNP | 15 | 79585665 | C | A | missense | ANKRD34C | ANKRD34C |  | Y |
| HCC-A2 | somaticSNP | 15 | 81572064 | A | T | missense | IL16 | IL16 |  | Y |
| HCC-A2 | somaticINDEL | 15 | 83241984 | A | -T | intron | CPEB1 |  |  | Y |
| HCC-A2 | somaticSNP | 16 | 332316 | G | A | synonymous | ARHGDIG | ARHGDIG |  | Y |
| HCC-A2 | somaticSNP | 16 | 842643 | A | T | intron | CHTF18 |  |  | Y |
| HCC-A2 | somaticSNP | 16 | 2821211 | T | C | 3-prime-UTR | SRRM2 | SRRM2 |  | Y |
| HCC-A2 | somaticSNP | 16 | 11556121 | A | G | synonymous | LOC400499 |  |  | N |
| HCC-A2 | somaticSNP | 16 | 15808975 | C | T | intron | NDE1,MYH11 |  | MYH11 | Y |
| HCC-A2 | somaticSNP | 16 | 30380445 | A | T | intron | TBC1D10B |  |  | Y |
| HCC-A2 | somaticSNP | 16 | 30941687 | A | G | synonymous,non-coding-exon | FBXL19 | FBXL19 |  | Y |
| HCC-A2 | somaticSNP | 16 | 31374294 | C | T | synonymous | ITGAX | ITGAX |  | Y |
| HCC-A2 | somaticSNP | 16 | 46540233 | A | G | intron | ANKRD26P1 |  |  | Y |
| HCC-A2 | somaticSNP | 16 | 55617059 | T | A | 3-prime-UTR | LPCAT2 | LPCAT2 |  | Y |
| HCC-A2 | somaticSNP | 16 | 57168812 | C | A | intron | CPNE2 |  |  | Y |
| HCC-A2 | somaticSNP | 16 | 71969447 | T | C | intron | PKD1L3 |  |  | Y |
| HCC-A2 | somaticSNP | 16 | 74382863 | T | C | non-coding-exon | LOC283922 | LOC283922 |  | Y |
| HCC-A2 | somaticSNP | 16 | 86601254 | C | T | missense | FOXC2 | FOXC2 |  | Y |
| HCC-A2 | somaticSNP | 17 | 4719892 | G | T | intron | PLD2 |  |  | Y |
| HCC-A2 | somaticSNP | 17 | 8076698 | G | C | 3-prime-UTR | TMEM107 | TMEM107 |  | Y |
| HCC-A2 | somaticINDEL | 17 | 9142981 | C | -G | nonsynonymous,del,frameshift | NTN1 | NTN1 |  | Y |
| HCC-A2 | somaticINDEL | 17 | 10550845 | T | -TTTTG | intron | MYH3 |  |  | N |
| HCC-A2 | somaticSNP | 17 | 11671943 | G | T | missense-near-splice | DNAH9 | DNAH9 |  | Y |
| HCC-A2 | somaticSNP | 17 | 12852477 | C | T | synonymous | ARHGAP44 | ARHGAP44 |  | Y |
| HCC-A2 | somaticSNP | 17 | 16040527 | T | C | intron | NCOR1 |  |  | Y |
| HCC-A2 | somaticSNP | 17 | 27822745 | G | T | missense-near-splice | TAOK1 | TAOK1 |  | Y |
| HCC-A2 | somaticSNP | 17 | 29226536 | G | T | missense | TEFM | TEFM |  | Y |
| HCC-A2 | somaticSNP | 17 | 38910804 | T | C | intron | KRT25 |  |  | Y |
| HCC-A2 | somaticSNP | 17 | 56429045 | C | A | intron | SUPT4H1,BZRAP1-AS1 |  |  | Y |
| HCC-A2 | somaticINDEL | 17 | 56693799 | C | +T | intron | TEX14 |  |  | N |
| HCC-A2 | somaticSNP | 17 | 57228448 | C | T | intron | SKA2 |  |  | N |
| HCC-A2 | somaticSNP | 17 | 71223299 | C | A | intron-near-splice | FAM104A |  |  | Y |
| HCC-A2 | somaticSNP | 17 | 74079838 | A | G | intron-near-splice | EXOC7 |  |  | N |
| HCC-A2 | somaticSNP | 17 | 75834846 | C | T | intergenic |  | |  | Y |
| HCC-A2 | somaticSNP | 17 | 80129776 | T | C | intron | CCDC57 |  |  | Y |
| HCC-A2 | somaticSNP | 18 | 12067950 | C | T | intergenic |  | |  | Y |
| HCC-A2 | somaticSNP | 18 | 12103292 | T | G | intron | ANKRD62 |  |  | Y |
| HCC-A2 | somaticSNP | 18 | 74620187 | A | T | intron | ZNF236 |  |  | Y |
| HCC-A2 | somaticSNP | 19 | 9007560 | A | G | intron-near-splice | MUC16 |  |  | Y |
| HCC-A2 | somaticSNP | 19 | 15276007 | C | A | intron | NOTCH3 |  |  | N |
| HCC-A2 | somaticSNP | 19 | 33359482 | C | T | 5-prime-UTR,intron | SLC7A9 | SLC7A9 |  | Y |
| HCC-A2 | somaticSNP | 19 | 34663663 | C | T | missense | LSM14A | LSM14A |  | Y |
| HCC-A2 | somaticSNP | 19 | 34858156 | A | T | intron | GPI |  |  | Y |
| HCC-A2 | somaticSNP | 19 | 38692623 | C | T | synonymous | SIPA1L3 | SIPA1L3 |  | Y |
| HCC-A2 | somaticSNP | 19 | 39689787 | C | A | intron | NCCRP1 |  |  | Y |
| HCC-A2 | somaticSNP | 19 | 41916759 | G | A | intron | BCKDHA |  |  | Y |
| HCC-A2 | somaticSNP | 19 | 42814990 | G | A | upstream-gene |  | |  | Y |
| HCC-A2 | somaticSNP | 19 | 45147340 | G | T | 5-prime-UTR | PVR | PVR |  | N |
| HCC-A2 | somaticSNP | 19 | 47763712 | G | C | intron | CCDC9 |  |  | Y |
| HCC-A2 | somaticSNP | 19 | 48244218 | G | A | synonymous | EHD2 | EHD2 |  | Y |
| HCC-A2 | somaticSNP | 19 | 49303120 | C | A | intron | BCAT2 |  |  | Y |
| HCC-A2 | somaticSNP | 19 | 50250504 | C | A | 5-prime-UTR,intron | TSKS |  |  | Y |
| HCC-A2 | somaticSNP | 19 | 51919279 | A | G | intron,synonymous,missense | SIGLEC10,LOC100129083 | SIGLEC10,LOC100129083 |  | Y |
| HCC-A2 | somaticSNP | 19 | 51919302 | T | C | intron,synonymous,missense | SIGLEC10,LOC100129083 | SIGLEC10,LOC100129083 |  | N |
| HCC-A2 | somaticSNP | 19 | 53576684 | C | A | intron,missense | ZNF160 |  |  | Y |
| HCC-A2 | somaticSNP | 19 | 56436029 | A | C | intron-near-splice | NLRP13 |  |  | Y |
| HCC-A2 | somaticSNP | 20 | 3219894 | A | G | intron | SLC4A11 |  |  | N |
| HCC-A2 | somaticSNP | 20 | 31887621 | A | T | intron | BPIFB1 |  |  | Y |
| HCC-A2 | somaticSNP | 20 | 32868834 | T | A | 3-prime-UTR | AHCY | AHCY |  | Y |
| HCC-A2 | somaticSNP | 20 | 34204981 | C | T | intron | SPAG4 |  |  | Y |
| HCC-A2 | somaticSNP | 20 | 37529300 | G | A | missense | PPP1R16B | PPP1R16B |  | Y |
| HCC-A2 | somaticINDEL | 20 | 43249004 | A | -TCT | nonsynonymous,del,frameshift | ADA | ADA |  | Y |
| HCC-A2 | somaticINDEL | 20 | 46281139 | A | -T | intron | NCOA3 |  |  | N |
| HCC-A2 | somaticSNP | 20 | 46295240 | C | T | intron-near-splice | SULF2 |  |  | Y |
| HCC-A2 | somaticSNP | 20 | 49202537 | G | C | downstream-gene |  | |  | Y |
| HCC-A2 | somaticSNP | 20 | 49225000 | C | A | synonymous | FAM65C | FAM65C |  | Y |
| HCC-A2 | somaticSNP | 20 | 55803351 | C | T | missense | BMP7 | BMP7 |  | Y |
| HCC-A2 | somaticSNP | 20 | 55908207 | T | C | intron | SPO11 |  |  | Y |
| HCC-A2 | somaticSNP | 20 | 58425592 | T | C | downstream-gene |  | |  | Y |
| HCC-A2 | somaticSNP | 21 | 16340367 | C | A | missense | NRIP1 | NRIP1 |  | Y |
| HCC-A2 | somaticSNP | 21 | 19642346 | G | A | synonymous | TMPRSS15 | TMPRSS15 |  | Y |
| HCC-A2 | somaticINDEL | 21 | 35186127 | C | -T | intron | ITSN1 |  |  | Y |
| HCC-A2 | somaticSNP | 21 | 41719719 | G | T | missense,non-coding-exon | DSCAM | DSCAM |  | Y |
| HCC-A2 | somaticSNP | 21 | 43541206 | A | C | missense | UMODL1 | UMODL1 |  | Y |
| HCC-A2 | somaticSNP | 21 | 45706413 | A | T | intron | AIRE |  |  | Y |
| HCC-A2 | somaticSNP | 22 | 23041026 | G | A | intergenic |  | |  | Y |
| HCC-A2 | somaticSNP | 22 | 25603127 | G | A | missense | CRYBB3 | CRYBB3 |  | Y |
| HCC-A2 | somaticSNP | 22 | 31335601 | C | A | intron-near-splice | MORC2 |  |  | Y |
| HCC-A2 | somaticSNP | 22 | 36690459 | T | C | intron | MYH9 |  | MYH9 | Y |
| HCC-A2 | somaticSNP | 22 | 42415519 | A | G | intron | WBP2NL |  |  | N |
| HCC-A2 | somaticSNP | 22 | 44594723 | T | C | intron | PARVG |  |  | Y |
| HCC-A2 | somaticSNP | 3 | 403347 | A | G | intron | CHL1 |  |  | Y |
| HCC-A2 | somaticSNP | X | 49851018 | C | T | missense,non-coding-exon | CLCN5 | CLCN5 |  | Y |
| HCC-A2 | somaticSNP | X | 112065959 | A | G | intron,synonymous | AMOT | AMOT |  | Y |
| HCC-A2 | somaticSNP | X | 117815120 | A | G | missense | DOCK11 | DOCK11 |  | Y |
| HCC-A2 | somaticSNP | X | 125686072 | G | T | missense | DCAF12L1 | DCAF12L1 |  | Y |
| HCC-A2 | somaticSNP | X | 153236100 | C | T | synonymous-near-splice | HCFC1 | HCFC1 |  | Y |
| HCC-A2 | somaticSNP | Y | 14532150 | G | A | non-coding-exon | GYG2P1 | GYG2P1 |  | Y |
| HCC-A2 | somaticSNP | 11 | 9075256 | T | C | intron,missense | SCUBE2 | SCUBE2 |  | Y |
| HCC-A2 | somaticSNP | 1 | 228547462 | C | T | intron,missense | OBSCN | OBSCN |  | Y |
| HCC-A2 | somaticSNP | 11 | 123847509 | A | G | missense | OR10S1 | OR10S1 |  | Y |
| HCC-A2 | somaticSNP | 12 | 80732939 | A | T | missense | OTOGL | OTOGL |  | Y |
| HCC-A2 | somaticSNP | 20 | 6060118 | T | C | missense | FERMT1 | FERMT1 |  | Y |
| HCC-A2 | somaticSNP | 9 | 112172661 | G | C | missense,non-coding-exon | PTPN3 | PTPN3 |  | Y |
| HCC-A2 | somaticSNP | 1 | 21012616 | G | A | missense | KIF17 | KIF17 |  | Y |
| HCC-A2 | somaticSNP | 1 | 34180246 | C | T | missense | CSMD2 | CSMD2 |  | Y |
| HCC-A2 | somaticSNP | 12 | 52882317 | C | T | missense | KRT6A | KRT6A |  | Y |
| HCC-A2 | somaticSNP | 12 | 64488721 | C | A | missense | SRGAP1 | SRGAP1 |  | Y |
| HCC-A2 | somaticSNP | 13 | 26117437 | C | T | missense | ATP8A2 | ATP8A2 |  | Y |
| HCC-A2 | somaticSNP | 13 | 37619503 | C | A | missense | SUPT20H | SUPT20H |  | Y |
| HCC-A2 | somaticSNP | 14 | 105613850 | T | A | missense | JAG2 | JAG2 |  | Y |
| HCC-A2 | somaticSNP | 15 | 23891689 | C | T | missense | MAGEL2 | MAGEL2 |  | Y |
| HCC-A2 | somaticSNP | 15 | 43528016 | C | G | missense | TGM5 | TGM5 |  | Y |
| HCC-A2 | somaticSNP | 16 | 347179 | G | T | stop-gained | AXIN1 | AXIN1 | AXIN1 | Y |
| HCC-A2 | somaticSNP | 16 | 58568227 | C | T | missense,non-coding-exon | CNOT1 | CNOT1 |  | Y |
| HCC-A2 | somaticSNP | 17 | 29161991 | G | A | missense | ATAD5 | ATAD5 |  | Y |
| HCC-A2 | somaticSNP | 17 | 57886199 | C | A | missense | VMP1 | VMP1 |  | Y |
| HCC-A2 | somaticSNP | 19 | 37368933 | A | T | stop-gained,intron | ZNF345 | ZNF345 |  | Y |
| HCC-A2 | somaticSNP | 19 | 50266498 | T | A | missense | TSKS | TSKS |  | Y |
| HCC-A2 | somaticSNP | 2 | 29379290 | A | C | missense | CLIP4 | CLIP4 |  | Y |
| HCC-A2 | somaticSNP | 2 | 228846479 | T | G | missense | SPHKAP | SPHKAP |  | Y |
| HCC-A2 | somaticSNP | 21 | 31798199 | G | C | missense | KRTAP13-3 | KRTAP13-3 |  | Y |
| HCC-A2 | somaticSNP | 3 | 49311526 | T | A | missense | C3orf62 | C3orf62 |  | Y |
| HCC-A2 | somaticSNP | 3 | 195517843 | G | A | intron,missense | MUC4 | MUC4 |  | Y |
| HCC-A2 | somaticSNP | 4 | 73164017 | T | A | missense | ADAMTS3 | ADAMTS3 |  | Y |
| HCC-A2 | somaticSNP | 4 | 126240096 | G | A | missense | FAT4 | FAT4 |  | Y |
| HCC-A2 | somaticSNP | 4 | 155507484 | T | A | missense | FGA | FGA |  | Y |
| HCC-A2 | somaticSNP | 5 | 16701535 | T | G | missense | MYO10 | MYO10 |  | Y |
| HCC-A2 | somaticSNP | 6 | 11104238 | C | A | stop-gained,intron | SMIM13,ERVFRD-1 | ERVFRD-1 |  | Y |
| HCC-A2 | somaticSNP | 6 | 33653560 | C | A | missense | ITPR3 | ITPR3 |  | Y |
| HCC-A2 | somaticSNP | 7 | 4304941 | G | T | intron,missense | SDK1 | SDK1 |  | Y |
| HCC-A2 | somaticSNP | 8 | 105393433 | T | A | missense | DPYS | DPYS |  | N |
| HCC-A2 | somaticSNP | 9 | 130860849 | C | T | intron,missense,non-coding-exon | SLC25A25 | SLC25A25 |  | Y |
| HCC-A2 | somaticSNP | X | 50053087 | T | A | intron,missense | CCNB3 | CCNB3 |  | Y |
| HCC-A2 | somaticSNP | X | 53250052 | G | A | 5-prime-UTR,intron,missense | KDM5C | KDM5C | KDM5C | Y |
| HCC-A2 | somaticSNP | 1 | 67559220 | T | C | 3-prime-UTR,missense,non-coding-exon | C1orf141 | C1orf141 |  | Y |
| HCC-A3 | somaticSNP | 1 | 111217382 | C | T | missense | KCNA3 | KCNA3 |  | Y |
| HCC-A3 | somaticSNP | 12 | 80732939 | A | T | missense | OTOGL | OTOGL |  | Y |
| HCC-A3 | somaticSNP | 20 | 6060118 | T | C | missense | FERMT1 | FERMT1 |  | Y |
| HCC-A3 | somaticSNP | 5 | 172110938 | G | A | intron,missense | NEURL1B | NEURL1B |  | Y |
| HCC-A3 | somaticSNP | 6 | 71233675 | A | G | missense | FAM135A | FAM135A |  | Y |
| HCC-A3 | somaticSNP | 7 | 151970859 | C | T | missense | KMT2C | KMT2C | KMT2C | N |
| HCC-A3 | somaticSNP | X | 49851018 | C | T | missense,non-coding-exon | CLCN5 | CLCN5 |  | Y |
| HCC-A3 | somaticSNP | 1 | 15855461 | T | G | intron | DNAJC16 |  |  | Y |
| HCC-A3 | somaticSNP | 1 | 17023483 | C | T | intron | ESPNP |  |  | N |
| HCC-A3 | somaticSNP | 1 | 17267496 | C | T | intron | CROCC |  |  | N |
| HCC-A3 | somaticINDEL | 1 | 24294450 | C | +A | intron | SRSF10 |  |  | N |
| HCC-A3 | somaticINDEL | 1 | 25780889 | A | +TG | intron | TMEM57 |  |  | N |
| HCC-A3 | somaticSNP | 1 | 26738094 | T | G | intron | LIN28A |  |  | Y |
| HCC-A3 | somaticSNP | 1 | 26873700 | C | T | 5-prime-UTR,synonymous | RPS6KA1 | RPS6KA1 |  | N |
| HCC-A3 | somaticSNP | 1 | 34180246 | C | T | missense | CSMD2 | CSMD2 |  | Y |
| HCC-A3 | somaticSNP | 1 | 36939412 | T | G | synonymous | CSF3R | CSF3R | CSF3R | Y |
| HCC-A3 | somaticSNP | 1 | 41327871 | C | A | 5-prime-UTR | CITED4 | CITED4 |  | N |
| HCC-A3 | somaticSNP | 1 | 53279594 | A | G | intron | ZYG11B |  |  | Y |
| HCC-A3 | somaticINDEL | 1 | 62350123 | G | -TTTTT | intron | INADL |  |  | N |
| HCC-A3 | somaticSNP | 1 | 62503632 | A | G | intron | INADL |  |  | Y |
| HCC-A3 | somaticSNP | 1 | 78267110 | G | A | missense | FAM73A | FAM73A |  | Y |
| HCC-A3 | somaticINDEL | 1 | 93159821 | C | -A | intron | EVI5 |  |  | Y |
| HCC-A3 | somaticSNP | 1 | 103385782 | T | C | intron | COL11A1 |  |  | Y |
| HCC-A3 | somaticSNP | 1 | 145281824 | T | G | intron | NBPF20,NBPF9,NOTCH2NL |  |  | Y |
| HCC-A3 | somaticSNP | 1 | 153642826 | C | T | intron | ILF2 |  |  | Y |
| HCC-A3 | somaticSNP | 1 | 156845864 | C | A | intron-near-splice,synonymous-near-splice | NTRK1 |  | NTRK1 | Y |
| HCC-A3 | somaticSNP | 1 | 158435706 | G | T | missense | OR10K1 | OR10K1 |  | Y |
| HCC-A3 | somaticSNP | 1 | 158687102 | A | G | synonymous | OR6K3 | OR6K3 |  | Y |
| HCC-A3 | somaticSNP | 1 | 165180036 | T | C | intron | LMX1A |  |  | Y |
| HCC-A3 | somaticSNP | 1 | 166959161 | T | C | intron | MAEL |  |  | Y |
| HCC-A3 | somaticSNP | 1 | 168250404 | G | A | missense | TBX19 | TBX19 |  | Y |
| HCC-A3 | somaticSNP | 1 | 181732615 | G | A | missense | CACNA1E | CACNA1E |  | Y |
| HCC-A3 | somaticSNP | 1 | 186122844 | A | G | intron | HMCN1,MIR548F1 |  |  | Y |
| HCC-A3 | somaticSNP | 1 | 205034434 | G | T | intron | CNTN2 |  |  | Y |
| HCC-A3 | somaticSNP | 1 | 227227740 | A | C | intron | CDC42BPA |  |  | Y |
| HCC-A3 | somaticSNP | 1 | 227922773 | G | C | 5-prime-UTR,missense | SNAP47,JMJD4 | SNAP47,JMJD4 |  | Y |
| HCC-A3 | somaticSNP | 1 | 228509727 | T | A | missense | OBSCN | OBSCN |  | Y |
| HCC-A3 | somaticSNP | 1 | 228547462 | C | T | intron,missense | OBSCN | OBSCN |  | Y |
| HCC-A3 | somaticSNP | 1 | 237773958 | A | T | intron | RYR2 |  |  | Y |
| HCC-A3 | somaticSNP | 1 | 247921652 | G | A | intron,synonymous | OR1C1 | OR1C1 |  | Y |
| HCC-A3 | somaticSNP | 10 | 1405432 | G | C | missense | ADARB2 | ADARB2 |  | Y |
| HCC-A3 | somaticSNP | 10 | 16948243 | C | T | missense | CUBN | CUBN |  | Y |
| HCC-A3 | somaticSNP | 10 | 17085892 | T | C | missense | CUBN | CUBN |  | Y |
| HCC-A3 | somaticSNP | 10 | 27381387 | C | T | missense | ANKRD26 | ANKRD26 |  | Y |
| HCC-A3 | somaticSNP | 10 | 28023428 | A | T | synonymous | MKX | MKX |  | Y |
| HCC-A3 | somaticSNP | 10 | 29581055 | T | C | intron | LYZL1 |  |  | N |
| HCC-A3 | somaticSNP | 10 | 32326541 | A | T | intron-near-splice | KIF5B |  | KIF5B | Y |
| HCC-A3 | somaticSNP | 10 | 75585233 | T | C | intron | CAMK2G |  |  | Y |
| HCC-A3 | somaticSNP | 10 | 82331347 | A | G | intron | SH2D4B |  |  | N |
| HCC-A3 | somaticINDEL | 10 | 93776240 | C | +T | intron | BTAF1 |  |  | Y |
| HCC-A3 | somaticINDEL | 10 | 101978688 | T | -AAAG | intron | CHUK |  |  | Y |
| HCC-A3 | somaticSNP | 10 | 102744480 | C | T | 3-prime-UTR,intron,missense | MRPL43,SEMA4G | SEMA4G |  | Y |
| HCC-A3 | somaticSNP | 10 | 116621277 | A | G | intron,missense | FAM160B1 | FAM160B1 |  | Y |
| HCC-A3 | somaticSNP | 10 | 121287023 | C | T | intron | RGS10 |  |  | Y |
| HCC-A3 | somaticSNP | 10 | 125425914 | G | A | 5-prime-UTR | GPR26 | GPR26 |  | Y |
| HCC-A3 | somaticSNP | 10 | 127265198 | A | T | intron,3-prime-UTR | TEX36-AS1 |  |  | Y |
| HCC-A3 | somaticSNP | 11 | 6412119 | A | G | synonymous | SMPD1 | SMPD1 |  | Y |
| HCC-A3 | somaticSNP | 11 | 8642032 | T | C | synonymous | TRIM66 | TRIM66 |  | Y |
| HCC-A3 | somaticSNP | 11 | 9075256 | T | C | intron,missense | SCUBE2 | SCUBE2 |  | Y |
| HCC-A3 | somaticSNP | 11 | 26563468 | A | T | intron | ANO3 |  |  | Y |
| HCC-A3 | somaticSNP | 11 | 57100642 | T | A | intron | SSRP1 |  |  | Y |
| HCC-A3 | somaticSNP | 11 | 61318960 | T | C | intron | SYT7 |  |  | Y |
| HCC-A3 | somaticSNP | 11 | 61912567 | A | C | intron-near-splice | INCENP |  |  | Y |
| HCC-A3 | somaticSNP | 11 | 64939850 | T | C | intron | SPDYC |  |  | Y |
| HCC-A3 | somaticSNP | 11 | 77825342 | G | T | synonymous | ALG8,RNU6-83P | ALG8 |  | Y |
| HCC-A3 | somaticSNP | 11 | 85685824 | G | A | intron,missense | PICALM | PICALM | PICALM | Y |
| HCC-A3 | somaticSNP | 11 | 89429907 | A | G | intron | FOLH1B |  |  | Y |
| HCC-A3 | somaticSNP | 11 | 101375549 | T | C | intron | TRPC6 |  |  | N |
| HCC-A3 | somaticSNP | 11 | 106807392 | C | A | intron,missense | GUCY1A2 |  |  | Y |
| HCC-A3 | somaticSNP | 11 | 113239077 | A | T | intron-near-splice,intron,3-prime-UTR | TTC12 |  |  | Y |
| HCC-A3 | somaticSNP | 11 | 118499010 | C | T | missense | PHLDB1 | PHLDB1 |  | Y |
| HCC-A3 | somaticSNP | 11 | 123847509 | A | G | missense | OR10S1 | OR10S1 |  | Y |
| HCC-A3 | somaticSNP | 11 | 125503061 | A | T | missense,non-coding-exon | CHEK1 | CHEK1 |  | Y |
| HCC-A3 | somaticSNP | 11 | 130079615 | G | T | missense | ST14 | ST14 |  | Y |
| HCC-A3 | somaticSNP | 11 | 130731699 | T | A | intergenic |  | |  | Y |
| HCC-A3 | somaticSNP | 12 | 8758100 | C | T | intron | AICDA |  |  | Y |
| HCC-A3 | somaticSNP | 12 | 10150721 | A | T | intron | CLEC1B |  |  | Y |
| HCC-A3 | somaticSNP | 12 | 21695584 | A | G | intron | GYS2 |  |  | N |
| HCC-A3 | somaticSNP | 12 | 25101801 | T | C | intron | BCAT1 |  |  | Y |
| HCC-A3 | somaticSNP | 12 | 31255227 | A | G | synonymous | DDX11 | DDX11 |  | N |
| HCC-A3 | somaticSNP | 12 | 48370448 | C | T | intron | COL2A1 |  |  | Y |
| HCC-A3 | somaticSNP | 12 | 53238112 | C | T | intron | KRT78 |  |  | N |
| HCC-A3 | somaticSNP | 12 | 72893286 | G | T | missense | TRHDE | TRHDE |  | Y |
| HCC-A3 | somaticSNP | 12 | 93287162 | C | T | intron | EEA1 |  |  | Y |
| HCC-A3 | somaticSNP | 12 | 104025383 | C | A | missense | STAB2 | STAB2 |  | Y |
| HCC-A3 | somaticSNP | 13 | 26117437 | C | T | missense | ATP8A2 | ATP8A2 |  | Y |
| HCC-A3 | somaticSNP | 13 | 26133834 | A | C | intron | ATP8A2 |  |  | Y |
| HCC-A3 | somaticSNP | 13 | 36424921 | A | G | intron,intron-near-splice | MIR548F5,DCLK1 |  |  | Y |
| HCC-A3 | somaticSNP | 13 | 46104056 | G | A | intron-near-splice,intron | COG3 |  |  | Y |
| HCC-A3 | somaticSNP | 14 | 22508913 | A | G | intergenic |  | |  | Y |
| HCC-A3 | somaticSNP | 14 | 22749767 | T | C | intergenic |  | |  | Y |
| HCC-A3 | somaticSNP | 14 | 23733725 | C | T | intron | C14orf164 |  |  | Y |
| HCC-A3 | somaticSNP | 14 | 23869882 | G | A | intron | MYH6 |  |  | N |
| HCC-A3 | somaticINDEL | 14 | 30095782 | G | +A | intron | PRKD1,MIR548AI |  |  | Y |
| HCC-A3 | somaticSNP | 14 | 37147839 | T | A | intron,3-prime-UTR | SLC25A21 | SLC25A21 |  | N |
| HCC-A3 | somaticSNP | 14 | 61643154 | T | C | intergenic |  | |  | Y |
| HCC-A3 | somaticSNP | 14 | 70826226 | C | G | intron | SYNJ2BP-COX16,COX16 |  |  | Y |
| HCC-A3 | somaticSNP | 14 | 72926278 | G | A | intron | RGS6 |  |  | Y |
| HCC-A3 | somaticSNP | 14 | 86088238 | C | T | missense | FLRT2 | FLRT2 |  | Y |
| HCC-A3 | somaticSNP | 14 | 104199580 | A | C | 3-prime-UTR | ZFYVE21 | ZFYVE21 |  | Y |
| HCC-A3 | somaticSNP | 15 | 25449634 | A | T | upstream-gene |  | |  | Y |
| HCC-A3 | somaticSNP | 15 | 28375351 | T | C | missense | HERC2 | HERC2 |  | Y |
| HCC-A3 | somaticSNP | 15 | 33893830 | T | A | intron | RYR3 |  |  | Y |
| HCC-A3 | somaticSNP | 15 | 33952558 | A | T | missense | RYR3 | RYR3 |  | Y |
| HCC-A3 | somaticSNP | 15 | 40846225 | G | A | intron,missense | C15orf57 | C15orf57 |  | Y |
| HCC-A3 | somaticSNP | 15 | 43528016 | C | G | missense | TGM5 | TGM5 |  | Y |
| HCC-A3 | somaticSNP | 15 | 43889786 | T | C | intron | RNU6-28P,CKMT1B |  |  | Y |
| HCC-A3 | somaticSNP | 15 | 48818257 | T | A | intron | FBN1 |  |  | Y |
| HCC-A3 | somaticINDEL | 15 | 83241984 | A | -T | intron | CPEB1 |  |  | N |
| HCC-A3 | somaticSNP | 16 | 332316 | G | A | synonymous | ARHGDIG | ARHGDIG |  | Y |
| HCC-A3 | somaticSNP | 16 | 842643 | A | T | intron | CHTF18 |  |  | Y |
| HCC-A3 | somaticSNP | 16 | 2821211 | T | C | 3-prime-UTR | SRRM2 | SRRM2 |  | Y |
| HCC-A3 | somaticSNP | 16 | 11556121 | A | G | synonymous | LOC400499 |  |  | Y |
| HCC-A3 | somaticSNP | 16 | 15808975 | C | T | intron | NDE1,MYH11 |  | MYH11 | Y |
| HCC-A3 | somaticSNP | 16 | 30380445 | A | T | intron | TBC1D10B |  |  | Y |
| HCC-A3 | somaticSNP | 16 | 30941687 | A | G | synonymous,non-coding-exon | FBXL19 | FBXL19 |  | Y |
| HCC-A3 | somaticSNP | 16 | 31374294 | C | T | synonymous | ITGAX | ITGAX |  | Y |
| HCC-A3 | somaticSNP | 16 | 46540233 | A | G | intron | ANKRD26P1 |  |  | Y |
| HCC-A3 | somaticSNP | 16 | 55617059 | T | A | 3-prime-UTR | LPCAT2 | LPCAT2 |  | Y |
| HCC-A3 | somaticSNP | 16 | 57168812 | C | A | intron | CPNE2 |  |  | Y |
| HCC-A3 | somaticSNP | 16 | 71969447 | T | C | intron | PKD1L3 |  |  | Y |
| HCC-A3 | somaticSNP | 16 | 74382863 | T | C | non-coding-exon | LOC283922 | LOC283922 |  | Y |
| HCC-A3 | somaticSNP | 16 | 86601254 | C | T | missense | FOXC2 | FOXC2 |  | Y |
| HCC-A3 | somaticSNP | 17 | 4719892 | G | T | intron | PLD2 |  |  | Y |
| HCC-A3 | somaticSNP | 17 | 8076698 | G | C | 3-prime-UTR | TMEM107 | TMEM107 |  | Y |
| HCC-A3 | somaticINDEL | 17 | 9142981 | C | -G | nonsynonymous,del,frameshift | NTN1 | NTN1 |  | Y |
| HCC-A3 | somaticSNP | 17 | 12852477 | C | T | synonymous | ARHGAP44 | ARHGAP44 |  | Y |
| HCC-A3 | somaticSNP | 17 | 16040527 | T | C | intron | NCOR1 |  |  | Y |
| HCC-A3 | somaticSNP | 17 | 27822745 | G | T | missense-near-splice | TAOK1 | TAOK1 |  | Y |
| HCC-A3 | somaticSNP | 17 | 38910804 | T | C | intron | KRT25 |  |  | Y |
| HCC-A3 | somaticSNP | 17 | 56429045 | C | A | intron | SUPT4H1,BZRAP1-AS1 |  |  | Y |
| HCC-A3 | somaticINDEL | 17 | 56693799 | C | +T | intron | TEX14 |  |  | Y |
| HCC-A3 | somaticSNP | 17 | 57228448 | C | T | intron | SKA2 |  |  | Y |
| HCC-A3 | somaticSNP | 17 | 57886199 | C | A | missense | VMP1 | VMP1 |  | Y |
| HCC-A3 | somaticSNP | 17 | 58326937 | C | T | intron | USP32 |  |  | Y |
| HCC-A3 | somaticSNP | 17 | 71223299 | C | A | intron-near-splice | FAM104A |  |  | Y |
| HCC-A3 | somaticSNP | 17 | 74079838 | A | G | intron-near-splice | EXOC7 |  |  | Y |
| HCC-A3 | somaticSNP | 17 | 75834846 | C | T | intergenic |  | |  | Y |
| HCC-A3 | somaticSNP | 17 | 80129776 | T | C | intron | CCDC57 |  |  | Y |
| HCC-A3 | somaticSNP | 18 | 12067950 | C | T | intergenic |  | |  | Y |
| HCC-A3 | somaticSNP | 18 | 12103292 | T | G | intron | ANKRD62 |  |  | Y |
| HCC-A3 | somaticSNP | 18 | 74620187 | A | T | intron | ZNF236 |  |  | Y |
| HCC-A3 | somaticSNP | 19 | 9007560 | A | G | intron-near-splice | MUC16 |  |  | Y |
| HCC-A3 | somaticSNP | 19 | 10266472 | G | T | intron | DNMT1 |  |  | N |
| HCC-A3 | somaticSNP | 19 | 15276007 | C | A | intron | NOTCH3 |  |  | Y |
| HCC-A3 | somaticSNP | 19 | 33359482 | C | T | 5-prime-UTR,intron | SLC7A9 | SLC7A9 |  | Y |
| HCC-A3 | somaticSNP | 19 | 34663663 | C | T | missense | LSM14A | LSM14A |  | Y |
| HCC-A3 | somaticSNP | 19 | 34858156 | A | T | intron | GPI |  |  | Y |
| HCC-A3 | somaticSNP | 19 | 37368933 | A | T | stop-gained,intron | ZNF345 | ZNF345 |  | Y |
| HCC-A3 | somaticSNP | 19 | 38692623 | C | T | synonymous | SIPA1L3 | SIPA1L3 |  | Y |
| HCC-A3 | somaticSNP | 19 | 39689787 | C | A | intron | NCCRP1 |  |  | Y |
| HCC-A3 | somaticSNP | 19 | 41916759 | G | A | intron | BCKDHA |  |  | Y |
| HCC-A3 | somaticSNP | 19 | 42814990 | G | A | upstream-gene |  | |  | Y |
| HCC-A3 | somaticSNP | 19 | 45147340 | G | T | 5-prime-UTR | PVR | PVR |  | N |
| HCC-A3 | somaticSNP | 19 | 47763712 | G | C | intron | CCDC9 |  |  | Y |
| HCC-A3 | somaticSNP | 19 | 48244218 | G | A | synonymous | EHD2 | EHD2 |  | Y |
| HCC-A3 | somaticSNP | 19 | 49303120 | C | A | intron | BCAT2 |  |  | Y |
| HCC-A3 | somaticSNP | 19 | 50250504 | C | A | 5-prime-UTR,intron | TSKS |  |  | Y |
| HCC-A3 | somaticSNP | 19 | 51919279 | A | G | intron,synonymous,missense | SIGLEC10,LOC100129083 | SIGLEC10,LOC100129083 |  | N |
| HCC-A3 | somaticSNP | 19 | 51919302 | T | C | intron,synonymous,missense | SIGLEC10,LOC100129083 | SIGLEC10,LOC100129083 |  | N |
| HCC-A3 | somaticSNP | 19 | 56436029 | A | C | intron-near-splice | NLRP13 |  |  | Y |
| HCC-A3 | somaticSNP | 2 | 29379290 | A | C | missense | CLIP4 | CLIP4 |  | Y |
| HCC-A3 | somaticSNP | 2 | 31412096 | T | C | intron | CAPN14 |  |  | N |
| HCC-A3 | somaticSNP | 2 | 54891911 | A | T | intron | SPTBN1 |  |  | Y |
| HCC-A3 | somaticSNP | 2 | 55433542 | T | C | intron | CLHC1 |  |  | Y |
| HCC-A3 | somaticSNP | 2 | 61020681 | A | T | intron | PAPOLG |  |  | Y |
| HCC-A3 | somaticSNP | 2 | 71635939 | C | G | intron | ZNF638 |  |  | Y |
| HCC-A3 | somaticSNP | 2 | 75116536 | A | T | missense | HK2 | HK2 |  | Y |
| HCC-A3 | somaticSNP | 2 | 98833151 | T | G | intron | VWA3B |  |  | Y |
| HCC-A3 | somaticSNP | 2 | 116447348 | A | G | intron | DPP10 |  |  | Y |
| HCC-A3 | somaticSNP | 2 | 118701541 | T | C | intron | CCDC93 |  |  | Y |
| HCC-A3 | somaticSNP | 2 | 128522167 | G | A | intron,synonymous | WDR33 | WDR33 |  | Y |
| HCC-A3 | somaticSNP | 2 | 133014556 | C | T | intron,non-coding-exon | MIR663B,ANKRD30BL | MIR663B |  | N |
| HCC-A3 | somaticSNP | 2 | 151331371 | T | C | intron | RND3 |  |  | Y |
| HCC-A3 | somaticSNP | 2 | 153551068 | C | T | synonymous | PRPF40A | PRPF40A |  | Y |
| HCC-A3 | somaticSNP | 2 | 177034400 | G | A | intron | HOXD3 |  |  | Y |
| HCC-A3 | somaticSNP | 2 | 197712586 | G | T | intron | PGAP1 |  |  | Y |
| HCC-A3 | somaticSNP | 2 | 201756675 | A | G | intron,synonymous | NIF3L1 | NIF3L1 |  | Y |
| HCC-A3 | somaticSNP | 2 | 210877639 | A | T | intron | RPE |  |  | Y |
| HCC-A3 | somaticSNP | 2 | 219558631 | A | G | missense | STK36 | STK36 |  | Y |
| HCC-A3 | somaticSNP | 2 | 220358383 | G | T | upstream-gene |  | |  | Y |
| HCC-A3 | somaticSNP | 2 | 228846479 | T | G | missense | SPHKAP | SPHKAP |  | Y |
| HCC-A3 | somaticSNP | 2 | 236708207 | A | G | intron | AGAP1 |  |  | Y |
| HCC-A3 | somaticSNP | 2 | 242800900 | G | A | intron | PDCD1 |  |  | Y |
| HCC-A3 | somaticSNP | 20 | 31887621 | A | T | intron | BPIFB1 |  |  | Y |
| HCC-A3 | somaticSNP | 20 | 32868834 | T | A | 3-prime-UTR | AHCY | AHCY |  | Y |
| HCC-A3 | somaticSNP | 20 | 34204981 | C | T | intron | SPAG4 |  |  | Y |
| HCC-A3 | somaticINDEL | 20 | 43249004 | A | -TCT | nonsynonymous,del,frameshift | ADA | ADA |  | Y |
| HCC-A3 | somaticINDEL | 20 | 46281139 | A | -T | intron | NCOA3 |  |  | N |
| HCC-A3 | somaticSNP | 20 | 46295240 | C | T | intron-near-splice | SULF2 |  |  | Y |
| HCC-A3 | somaticSNP | 20 | 49202537 | G | C | downstream-gene |  | |  | Y |
| HCC-A3 | somaticSNP | 20 | 49225000 | C | A | synonymous | FAM65C | FAM65C |  | Y |
| HCC-A3 | somaticSNP | 20 | 55803351 | C | T | missense | BMP7 | BMP7 |  | Y |
| HCC-A3 | somaticSNP | 20 | 55908207 | T | C | intron | SPO11 |  |  | Y |
| HCC-A3 | somaticSNP | 20 | 58425592 | T | C | downstream-gene |  | |  | Y |
| HCC-A3 | somaticINDEL | 21 | 19628809 | C | -T | intron | CHODL |  |  | N |
| HCC-A3 | somaticSNP | 21 | 19642346 | G | A | synonymous | TMPRSS15 | TMPRSS15 |  | Y |
| HCC-A3 | somaticSNP | 21 | 31798199 | G | C | missense | KRTAP13-3 | KRTAP13-3 |  | Y |
| HCC-A3 | somaticINDEL | 21 | 35186127 | C | -T | intron | ITSN1 |  |  | N |
| HCC-A3 | somaticSNP | 21 | 41719719 | G | T | missense,non-coding-exon | DSCAM | DSCAM |  | Y |
| HCC-A3 | somaticSNP | 21 | 43541206 | A | C | missense | UMODL1 | UMODL1 |  | Y |
| HCC-A3 | somaticSNP | 21 | 45706413 | A | T | intron | AIRE |  |  | Y |
| HCC-A3 | somaticSNP | 22 | 23041026 | G | A | intergenic |  | |  | Y |
| HCC-A3 | somaticSNP | 22 | 25603127 | G | A | missense | CRYBB3 | CRYBB3 |  | Y |
| HCC-A3 | somaticSNP | 22 | 31335601 | C | A | intron-near-splice | MORC2 |  |  | Y |
| HCC-A3 | somaticSNP | 22 | 36690459 | T | C | intron | MYH9 |  | MYH9 | N |
| HCC-A3 | somaticSNP | 22 | 38622957 | C | A | intron | TMEM184B |  |  | Y |
| HCC-A3 | somaticSNP | 22 | 44594723 | T | C | intron | PARVG |  |  | Y |
| HCC-A3 | somaticSNP | 3 | 403347 | A | G | intron | CHL1 |  |  | Y |
| HCC-A3 | somaticINDEL | 3 | 19389266 | C | +A | nonsynonymous,ins,frameshift | KCNH8 | KCNH8 |  | Y |
| HCC-A3 | somaticSNP | 3 | 38051009 | C | T | intron | PLCD1 |  |  | Y |
| HCC-A3 | somaticSNP | 3 | 39448229 | A | G | 5-prime-UTR | RPSA | RPSA |  | Y |
| HCC-A3 | somaticSNP | 3 | 44283610 | T | A | missense | TOPAZ1 | TOPAZ1 |  | Y |
| HCC-A3 | somaticSNP | 3 | 48594104 | C | G | intron | PFKFB4 |  |  | Y |
| HCC-A3 | somaticSNP | 3 | 49311526 | T | A | missense | C3orf62 | C3orf62 |  | Y |
| HCC-A3 | somaticINDEL | 3 | 57108374 | T | -C | nonsynonymous,del,frameshift | SPATA12,ARHGEF3 | SPATA12 |  | Y |
| HCC-A3 | somaticSNP | 3 | 69112049 | C | T | intron | UBA3 |  |  | N |
| HCC-A3 | somaticSNP | 3 | 97464291 | T | A | intron | EPHA6 |  |  | Y |
| HCC-A3 | somaticSNP | 3 | 100084408 | T | C | synonymous | TOMM70A | TOMM70A |  | Y |
| HCC-A3 | somaticSNP | 3 | 121132268 | T | A | intron | STXBP5L |  |  | Y |
| HCC-A3 | somaticSNP | 3 | 124449461 | G | A | 5-prime-UTR,missense,non-coding-exon | UMPS | UMPS |  | Y |
| HCC-A3 | somaticSNP | 3 | 124689398 | G | A | 3-prime-UTR | HEG1 | HEG1 |  | Y |
| HCC-A3 | somaticSNP | 3 | 133877257 | C | T | intron | RYK |  |  | Y |
| HCC-A3 | somaticSNP | 3 | 142840069 | T | A | synonymous | CHST2 | CHST2 |  | Y |
| HCC-A3 | somaticSNP | 3 | 171965580 | C | T | intron | FNDC3B |  |  | Y |
| HCC-A3 | somaticSNP | 3 | 184299095 | A | C | synonymous | EPHB3 | EPHB3 |  | Y |
| HCC-A3 | somaticSNP | 3 | 184552484 | G | A | missense | VPS8 | VPS8 |  | Y |
| HCC-A3 | somaticSNP | 3 | 185879581 | T | A | intron | DGKG |  |  | Y |
| HCC-A3 | somaticSNP | 3 | 190995829 | A | T | intron | UTS2B |  |  | Y |
| HCC-A3 | somaticSNP | 3 | 193855537 | G | T | stop-gained | HES1 | HES1 |  | Y |
| HCC-A3 | somaticSNP | 3 | 194118705 | G | A | synonymous | GP5 | GP5 |  | Y |
| HCC-A3 | somaticSNP | 3 | 195517843 | G | A | intron,missense | MUC4 | MUC4 |  | Y |
| HCC-A3 | somaticSNP | 4 | 2900240 | G | A | synonymous | ADD1 | ADD1 |  | Y |
| HCC-A3 | somaticSNP | 4 | 10020564 | G | A | intron | SLC2A9 |  |  | Y |
| HCC-A3 | somaticINDEL | 4 | 74281947 | A | -TT | intron | ALB |  |  | Y |
| HCC-A3 | somaticSNP | 4 | 103556316 | T | C | intron | MANBA |  |  | N |
| HCC-A3 | somaticINDEL | 4 | 106317370 | T | +ATAAA | intron | PPA2 |  |  | N |
| HCC-A3 | somaticSNP | 4 | 122068167 | T | C | intron | TNIP3 |  |  | Y |
| HCC-A3 | somaticSNP | 4 | 125631753 | T | C | 5-prime-UTR,intron | ANKRD50 | ANKRD50 |  | Y |
| HCC-A3 | somaticSNP | 5 | 7414718 | A | G | synonymous | ADCY2 | ADCY2 |  | Y |
| HCC-A3 | somaticSNP | 5 | 16701535 | T | G | missense | MYO10 | MYO10 |  | Y |
| HCC-A3 | somaticSNP | 5 | 74931692 | A | G | missense | ANKDD1B | ANKDD1B |  | Y |
| HCC-A3 | somaticSNP | 5 | 76714070 | T | A | stop-gained | PDE8B | PDE8B |  | Y |
| HCC-A3 | somaticINDEL | 5 | 78416973 | C | -A | intron | BHMT |  |  | Y |
| HCC-A3 | somaticSNP | 5 | 135396468 | T | A | intron | TGFBI |  |  | Y |
| HCC-A3 | somaticSNP | 5 | 145531578 | G | A | intron | LARS |  |  | Y |
| HCC-A3 | somaticSNP | 5 | 149112300 | T | G | intron | PPARGC1B |  |  | Y |
| HCC-A3 | somaticSNP | 5 | 169309494 | T | A | intron | DOCK2,FAM196B |  |  | Y |
| HCC-A3 | somaticSNP | 5 | 178506468 | G | T | missense | ZNF354C | ZNF354C |  | Y |
| HCC-A3 | somaticSNP | 6 | 5000779 | G | A | intron | RPP40 |  |  | N |
| HCC-A3 | somaticSNP | 6 | 11104238 | C | A | stop-gained,intron | SMIM13,ERVFRD-1 | ERVFRD-1 |  | Y |
| HCC-A3 | somaticSNP | 6 | 28197605 | C | T | intron | ZSCAN9 |  |  | N |
| HCC-A3 | somaticSNP | 6 | 34029881 | C | T | intron | GRM4 |  |  | Y |
| HCC-A3 | somaticSNP | 6 | 75801024 | G | T | intron | COL12A1 |  |  | Y |
| HCC-A3 | somaticSNP | 6 | 76720848 | T | A | intron | IMPG1 |  |  | Y |
| HCC-A3 | somaticSNP | 6 | 99848727 | T | C | missense | PNISR | PNISR |  | Y |
| HCC-A3 | somaticSNP | 6 | 108831414 | G | A | splice-acceptor | LACE1 | LACE1 |  | Y |
| HCC-A3 | somaticSNP | 6 | 111329423 | G | T | intron | RPF2 |  |  | Y |
| HCC-A3 | somaticSNP | 6 | 129799721 | T | C | intron | LAMA2 |  |  | N |
| HCC-A3 | somaticSNP | 6 | 144508629 | C | A | 3-prime-UTR | STX11 | STX11 |  | Y |
| HCC-A3 | somaticSNP | 6 | 167426949 | A | G | intron | FGFR1OP |  | FGFR1OP | Y |
| HCC-A3 | somaticSNP | 6 | 167446139 | A | G | intron | FGFR1OP |  | FGFR1OP | Y |
| HCC-A3 | somaticSNP | 6 | 170034675 | A | T | intron | WDR27 |  |  | Y |
| HCC-A3 | somaticSNP | 6 | 170049362 | C | A | missense | WDR27 | WDR27 |  | Y |
| HCC-A3 | somaticSNP | 7 | 330562 | G | A | intron | WI2-2373I1.2 |  |  | Y |
| HCC-A3 | somaticSNP | 7 | 1914350 | A | G | intron | MAD1L1 |  |  | N |
| HCC-A3 | somaticSNP | 7 | 12409830 | T | C | missense | VWDE | VWDE |  | Y |
| HCC-A3 | somaticSNP | 7 | 12610475 | C | T | synonymous | SCIN | SCIN |  | Y |
| HCC-A3 | somaticSNP | 7 | 19156335 | G | T | 3-prime-UTR | TWIST1 | TWIST1 |  | Y |
| HCC-A3 | somaticINDEL | 7 | 23296415 | G | +A | intron | GPNMB |  |  | Y |
| HCC-A3 | somaticSNP | 7 | 26404329 | T | C | intron | SNX10 |  |  | Y |
| HCC-A3 | somaticSNP | 7 | 44619235 | G | A | intron-near-splice | TMED4 |  |  | Y |
| HCC-A3 | somaticSNP | 7 | 63726690 | C | A | missense | ZNF679 | ZNF679 |  | Y |
| HCC-A3 | somaticSNP | 7 | 80374631 | A | G | intron-near-splice | SEMA3C |  |  | Y |
| HCC-A3 | somaticSNP | 7 | 83035341 | T | G | missense | SEMA3E | SEMA3E |  | Y |
| HCC-A3 | somaticSNP | 7 | 99578370 | T | G | upstream-gene |  | |  | N |
| HCC-A3 | somaticSNP | 7 | 99578381 | T | C | upstream-gene |  | |  | N |
| HCC-A3 | somaticSNP | 7 | 99578411 | T | C | non-coding-exon | AZGP1P1 | AZGP1P1 |  | Y |
| HCC-A3 | somaticSNP | 7 | 99691647 | T | C | intron,non-coding-exon | MIR106B,MCM7 | MIR106B |  | Y |
| HCC-A3 | somaticINDEL | 7 | 116409675 | C | -T | intron | MET |  | MET | N |
| HCC-A3 | somaticSNP | 7 | 131982688 | C | A | intron | PLXNA4 |  |  | N |
| HCC-A3 | somaticSNP | 7 | 142045484 | C | A | intergenic |  | |  | Y |
| HCC-A3 | somaticSNP | 7 | 142561350 | G | T | intron | EPHB6 |  |  | Y |
| HCC-A3 | somaticSNP | 7 | 143095395 | C | T | intron | EPHA1 |  |  | Y |
| HCC-A3 | somaticSNP | 7 | 143573132 | C | T | intron,synonymous | FAM115A | FAM115A |  | Y |
| HCC-A3 | somaticSNP | 8 | 35579196 | T | C | intron | UNC5D |  |  | Y |
| HCC-A3 | somaticSNP | 8 | 39103668 | C | G | missense | ADAM32 | ADAM32 |  | Y |
| HCC-A3 | somaticSNP | 8 | 39862909 | T | C | intron | IDO2 |  |  | Y |
| HCC-A3 | somaticSNP | 8 | 69013781 | T | A | intron | PREX2 |  |  | Y |
| HCC-A3 | somaticSNP | 8 | 69351923 | A | C | intron | C8orf34 |  |  | Y |
| HCC-A3 | somaticSNP | 8 | 73150337 | A | T | non-coding-exon | LOC392232 | LOC392232 |  | Y |
| HCC-A3 | somaticSNP | 8 | 73479912 | T | A | 5-prime-UTR | KCNB2 | KCNB2 |  | Y |
| HCC-A3 | somaticSNP | 8 | 104897740 | A | G | missense | RIMS2 | RIMS2 |  | Y |
| HCC-A3 | somaticSNP | 8 | 105393433 | T | A | missense | DPYS | DPYS |  | N |
| HCC-A3 | somaticSNP | 8 | 113418760 | A | T | synonymous | CSMD3 | CSMD3 |  | Y |
| HCC-A3 | somaticSNP | 8 | 125047646 | C | T | intron,synonymous | FER1L6,FER1L6-AS1 | FER1L6 |  | Y |
| HCC-A3 | somaticSNP | 8 | 131964000 | C | A | intron | ADCY8 |  |  | Y |
| HCC-A3 | somaticSNP | 8 | 141712834 | T | C | intron | PTK2 |  |  | Y |
| HCC-A3 | somaticSNP | 8 | 143614849 | G | T | intron | BAI1 |  |  | Y |
| HCC-A3 | somaticSNP | 8 | 146220171 | T | C | intron | ZNF252P |  |  | Y |
| HCC-A3 | somaticSNP | 9 | 14784688 | A | G | intron | FREM1 |  |  | Y |
| HCC-A3 | somaticSNP | 9 | 84202674 | C | G | synonymous | TLE1 | TLE1 |  | Y |
| HCC-A3 | somaticSNP | 9 | 91360763 | G | A | non-coding-exon | MIR4289 | MIR4289 |  | Y |
| HCC-A3 | somaticSNP | 9 | 98766997 | G | T | intron | ERCC6L2 |  |  | Y |
| HCC-A3 | somaticSNP | 9 | 99314276 | G | C | intron | CDC14B |  |  | Y |
| HCC-A3 | somaticSNP | 9 | 105763940 | T | C | intron | CYLC2 |  |  | Y |
| HCC-A3 | somaticSNP | 9 | 111693233 | C | A | intron | IKBKAP |  |  | Y |
| HCC-A3 | somaticSNP | 9 | 112172661 | G | C | missense,non-coding-exon | PTPN3 | PTPN3 |  | Y |
| HCC-A3 | somaticSNP | 9 | 113341468 | G | T | missense | SVEP1 | SVEP1 |  | Y |
| HCC-A3 | somaticSNP | 9 | 133554034 | G | C | intron-near-splice | PRDM12 |  |  | Y |
| HCC-A3 | somaticSNP | 9 | 139396088 | C | A | intron | NOTCH1 |  | NOTCH1 | Y |
| HCC-A3 | somaticSNP | 9 | 139904603 | T | A | intron | ABCA2 |  |  | Y |
| HCC-A3 | somaticSNP | X | 50053087 | T | A | intron,missense | CCNB3 | CCNB3 |  | Y |
| HCC-A3 | somaticSNP | X | 53250052 | G | A | 5-prime-UTR,intron,missense | KDM5C | KDM5C | KDM5C | Y |
| HCC-A3 | somaticSNP | X | 112065959 | A | G | intron,synonymous | AMOT | AMOT |  | Y |
| HCC-A3 | somaticSNP | X | 125686072 | G | T | missense | DCAF12L1 | DCAF12L1 |  | Y |
| HCC-A3 | somaticSNP | X | 153236100 | C | T | synonymous-near-splice | HCFC1 | HCFC1 |  | Y |
| HCC-A3 | somaticSNP | Y | 14532150 | G | A | non-coding-exon | GYG2P1 | GYG2P1 |  | Y |
| HCC-A3 | somaticSNP | 2 | 166908325 | G | T | missense | SCN1A | SCN1A |  | Y |
| HCC-A3 | somaticSNP | 9 | 112141909 | T | C | missense,non-coding-exon | PTPN3 | PTPN3 |  | Y |
| HCC-A3 | somaticSNP | 1 | 19062153 | G | A | missense | PAX7 | PAX7 | PAX7 | Y |
| HCC-A3 | somaticSNP | 1 | 21012616 | G | A | missense | KIF17 | KIF17 |  | Y |
| HCC-A3 | somaticSNP | 1 | 22158261 | T | C | missense | HSPG2 | HSPG2 |  | Y |
| HCC-A3 | somaticSNP | 1 | 120306816 | T | C | missense | HMGCS2 | HMGCS2 |  | Y |
| HCC-A3 | somaticSNP | 1 | 157514235 | C | G | missense | FCRL5 | FCRL5 |  | Y |
| HCC-A3 | somaticSNP | 11 | 35747593 | C | T | missense | TRIM44 | TRIM44 |  | Y |
| HCC-A3 | somaticSNP | 11 | 64876028 | G | A | missense,non-coding-exon | VPS51 | VPS51 |  | N |
| HCC-A3 | somaticSNP | 11 | 86666063 | C | T | missense | FZD4 | FZD4 |  | N |
| HCC-A3 | somaticSNP | 12 | 52882317 | C | T | missense | KRT6A | KRT6A |  | Y |
| HCC-A3 | somaticSNP | 12 | 64488721 | C | A | missense | SRGAP1 | SRGAP1 |  | Y |
| HCC-A3 | somaticSNP | 12 | 96932190 | T | A | missense | LOC101928871 | LOC101928871 |  | Y |
| HCC-A3 | somaticSNP | 12 | 132502115 | A | C | missense | EP400 | EP400 |  | Y |
| HCC-A3 | somaticSNP | 13 | 37619503 | C | A | missense | SUPT20H | SUPT20H |  | Y |
| HCC-A3 | somaticSNP | 14 | 70245100 | A | C | missense | SLC10A1 | SLC10A1 |  | Y |
| HCC-A3 | somaticSNP | 14 | 105613850 | T | A | missense | JAG2 | JAG2 |  | Y |
| HCC-A3 | somaticSNP | 15 | 23891689 | C | T | missense | MAGEL2 | MAGEL2 |  | Y |
| HCC-A3 | somaticSNP | 15 | 79585665 | C | A | missense | ANKRD34C | ANKRD34C |  | Y |
| HCC-A3 | somaticSNP | 15 | 81572064 | A | T | missense | IL16 | IL16 |  | Y |
| HCC-A3 | somaticSNP | 16 | 347179 | G | T | stop-gained | AXIN1 | AXIN1 | AXIN1 | Y |
| HCC-A3 | somaticSNP | 16 | 58568227 | C | T | missense,non-coding-exon | CNOT1 | CNOT1 |  | Y |
| HCC-A3 | somaticSNP | 17 | 11671943 | G | T | missense-near-splice | DNAH9 | DNAH9 |  | Y |
| HCC-A3 | somaticSNP | 17 | 29161991 | G | A | missense | ATAD5 | ATAD5 |  | Y |
| HCC-A3 | somaticSNP | 17 | 29226536 | G | T | missense | TEFM | TEFM |  | Y |
| HCC-A3 | somaticSNP | 19 | 50266498 | T | A | missense | TSKS | TSKS |  | Y |
| HCC-A3 | somaticSNP | 19 | 53576684 | C | A | intron,missense | ZNF160 |  |  | Y |
| HCC-A3 | somaticSNP | 2 | 21239443 | G | T | missense | APOB | APOB |  | Y |
| HCC-A3 | somaticSNP | 2 | 153574008 | C | A | 5-prime-UTR,missense | PRPF40A | PRPF40A |  | Y |
| HCC-A3 | somaticSNP | 20 | 37529300 | G | A | missense | PPP1R16B | PPP1R16B |  | Y |
| HCC-A3 | somaticSNP | 21 | 16340367 | C | A | missense | NRIP1 | NRIP1 |  | Y |
| HCC-A3 | somaticSNP | 4 | 73164017 | T | A | missense | ADAMTS3 | ADAMTS3 |  | Y |
| HCC-A3 | somaticSNP | 4 | 90034354 | G | C | missense | TIGD2 | TIGD2 |  | Y |
| HCC-A3 | somaticSNP | 4 | 126240096 | G | A | missense | FAT4 | FAT4 |  | Y |
| HCC-A3 | somaticSNP | 4 | 155507484 | T | A | missense | FGA | FGA |  | Y |
| HCC-A3 | somaticSNP | 4 | 155533047 | A | G | missense | FGG | FGG |  | Y |
| HCC-A3 | somaticSNP | 5 | 169412889 | C | T | missense | DOCK2 | DOCK2 |  | Y |
| HCC-A3 | somaticSNP | 6 | 33653560 | C | A | missense | ITPR3 | ITPR3 |  | Y |
| HCC-A3 | somaticSNP | 6 | 132874297 | T | A | missense | TAAR8 | TAAR8 |  | Y |
| HCC-A3 | somaticSNP | 6 | 149771895 | T | A | missense | ZC3H12D | ZC3H12D |  | Y |
| HCC-A3 | somaticSNP | 7 | 4304941 | G | T | intron,missense | SDK1 | SDK1 |  | Y |
| HCC-A3 | somaticSNP | 7 | 48146967 | G | A | missense | UPP1 | UPP1 |  | Y |
| HCC-A3 | somaticSNP | 7 | 107332509 | G | A | intron,missense | SLC26A4 |  |  | Y |
| HCC-A3 | somaticSNP | 9 | 130860849 | C | T | intron,missense,non-coding-exon | SLC25A25 | SLC25A25 |  | Y |
| HCC-A3 | somaticSNP | X | 117815120 | A | G | missense | DOCK11 | DOCK11 |  | Y |
| HCC-A3 | somaticSNP | 1 | 67559220 | T | C | 3-prime-UTR,missense,non-coding-exon | C1orf141 | C1orf141 |  | Y |
| HCC-A3 | somaticSNP | 10 | 106039197 | G | A | intron,missense | GSTO2 | GSTO2 |  | Y |
| HCC-B1 | somaticSNP | 11 | 56086317 | T | A | missense | OR8K3 | OR8K3 |  | N |
| HCC-B1 | somaticSNP | 15 | 79304864 | G | T | intron,missense | RASGRF1 | RASGRF1 |  | N |
| HCC-B1 | somaticSNP | 6 | 46672392 | C | T | missense | PLA2G7 | PLA2G7 |  | N |
| HCC-B1 | somaticSNP | 9 | 96285612 | C | T | intron,missense | FAM120A |  |  | N |
| HCC-B1 | somaticSNP | 1 | 17023483 | C | T | intron | ESPNP |  |  | Y |
| HCC-B1 | somaticINDEL | 1 | 24294450 | C | +A | intron | SRSF10 |  |  | N |
| HCC-B1 | somaticINDEL | 1 | 25780889 | A | +TG | intron | TMEM57 |  |  | N |
| HCC-B1 | somaticSNP | 1 | 26873700 | C | T | 5-prime-UTR,synonymous | RPS6KA1 | RPS6KA1 |  | N |
| HCC-B1 | somaticSNP | 1 | 29611123 | C | T | intron | PTPRU |  |  | N |
| HCC-B1 | somaticSNP | 1 | 36028719 | C | T | intron | NCDN |  |  | N |
| HCC-B1 | somaticSNP | 1 | 41327871 | C | A | 5-prime-UTR | CITED4 | CITED4 |  | Y |
| HCC-B1 | somaticSNP | 1 | 44363924 | A | G | intron,missense,non-coding-exon | ST3GAL3 | ST3GAL3 |  | Y |
| HCC-B1 | somaticINDEL | 1 | 62350123 | G | -TTTTT | intron | INADL |  |  | N |
| HCC-B1 | somaticSNP | 1 | 67861900 | C | A | 3-prime-UTR,non-coding-exon | IL12RB2 | IL12RB2 |  | Y |
| HCC-B1 | somaticSNP | 1 | 85431403 | G | C | intron | MCOLN2 |  |  | N |
| HCC-B1 | somaticSNP | 1 | 86430684 | T | A | intron-near-splice | COL24A1 |  |  | Y |
| HCC-B1 | somaticINDEL | 1 | 93159821 | C | -A | intron | EVI5 |  |  | N |
| HCC-B1 | somaticSNP | 1 | 109813860 | C | T | synonymous | CELSR2 | CELSR2 |  | N |
| HCC-B1 | somaticSNP | 1 | 157716473 | C | T | 3-prime-UTR | FCRL2 | FCRL2 |  | Y |
| HCC-B1 | somaticSNP | 1 | 158609897 | G | C | intron | SPTA1 |  |  | Y |
| HCC-B1 | somaticSNP | 1 | 158746420 | A | T | downstream-gene |  | |  | Y |
| HCC-B1 | somaticSNP | 1 | 159824488 | A | G | 3-prime-UTR,intron | VSIG8,C1orf204 | VSIG8 |  | Y |
| HCC-B1 | somaticSNP | 1 | 167742442 | A | G | intron | MPZL1 |  |  | Y |
| HCC-B1 | somaticSNP | 1 | 181700435 | C | A | intron | CACNA1E |  |  | Y |
| HCC-B1 | somaticSNP | 1 | 182443101 | A | T | missense | RGSL1 | RGSL1 |  | Y |
| HCC-B1 | somaticINDEL | 1 | 193202032 | C | +A | intron | CDC73 |  | CDC73 | Y |
| HCC-B1 | somaticSNP | 1 | 205034434 | G | T | intron | CNTN2 |  |  | Y |
| HCC-B1 | somaticSNP | 1 | 227227740 | A | C | intron | CDC42BPA |  |  | N |
| HCC-B1 | somaticSNP | 1 | 245703873 | T | C | intron | KIF26B |  |  | Y |
| HCC-B1 | somaticSNP | 1 | 247051591 | C | A | intron | AHCTF1 |  |  | Y |
| HCC-B1 | somaticSNP | 10 | 29581055 | T | C | intron | LYZL1 |  |  | N |
| HCC-B1 | somaticINDEL | 10 | 60160134 | T | -AAA | intergenic |  | |  | N |
| HCC-B1 | somaticSNP | 10 | 75090988 | T | A | missense | TTC18 | TTC18 |  | Y |
| HCC-B1 | somaticSNP | 10 | 82331347 | A | G | intron | SH2D4B |  |  | N |
| HCC-B1 | somaticINDEL | 10 | 93776240 | C | +T | intron | BTAF1 |  |  | Y |
| HCC-B1 | somaticINDEL | 10 | 95454768 | T | -A | intron | FRA10AC1 |  |  | N |
| HCC-B1 | somaticSNP | 10 | 118671072 | G | C | intron | KIAA1598 |  |  | Y |
| HCC-B1 | somaticSNP | 10 | 134188508 | C | T | intron | LRRC27 |  |  | Y |
| HCC-B1 | somaticSNP | 11 | 18750380 | G | C | 3-prime-UTR | PTPN5 | PTPN5 |  | N |
| HCC-B1 | somaticSNP | 11 | 20940973 | C | A | intron | NELL1 |  |  | N |
| HCC-B1 | somaticSNP | 11 | 65988562 | A | T | intron | PACS1 |  |  | N |
| HCC-B1 | somaticSNP | 11 | 68213996 | G | A | synonymous | LRP5 | LRP5 |  | Y |
| HCC-B1 | somaticSNP | 11 | 73536698 | A | C | intron | MRPL48 |  |  | N |
| HCC-B1 | somaticSNP | 11 | 101375549 | T | C | intron | TRPC6 |  |  | Y |
| HCC-B1 | somaticSNP | 11 | 122647728 | A | T | intron-near-splice | UBASH3B |  |  | Y |
| HCC-B1 | somaticSNP | 12 | 21695584 | A | G | intron | GYS2 |  |  | N |
| HCC-B1 | somaticSNP | 12 | 31255227 | A | G | synonymous | DDX11 | DDX11 |  | N |
| HCC-B1 | somaticSNP | 12 | 40258589 | G | C | missense | SLC2A13 | SLC2A13 |  | N |
| HCC-B1 | somaticSNP | 12 | 51451772 | A | G | intron | LETMD1 |  |  | N |
| HCC-B1 | somaticSNP | 12 | 53238112 | C | T | intron | KRT78 |  |  | Y |
| HCC-B1 | somaticSNP | 12 | 95915034 | C | G | intron | USP44 |  |  | Y |
| HCC-B1 | somaticINDEL | 12 | 97311376 | C | -T | intron | NEDD1 |  |  | N |
| HCC-B1 | somaticSNP | 13 | 20277413 | T | C | missense | PSPC1 | PSPC1 |  | N |
| HCC-B1 | somaticSNP | 13 | 43788226 | A | G | missense | ENOX1 | ENOX1 |  | Y |
| HCC-B1 | somaticSNP | 13 | 47470954 | C | A | 5-prime-UTR,missense | HTR2A | HTR2A |  | Y |
| HCC-B1 | somaticSNP | 13 | 114309228 | A | T | missense | ATP4B | ATP4B |  | N |
| HCC-B1 | somaticSNP | 14 | 23733725 | C | T | intron | C14orf164 |  |  | N |
| HCC-B1 | somaticSNP | 14 | 45639980 | T | C | intron | FANCM |  |  | Y |
| HCC-B1 | somaticSNP | 14 | 56079145 | G | T | stop-gained,non-coding-exon | KTN1 | KTN1 | KTN1 | Y |
| HCC-B1 | somaticSNP | 14 | 58583074 | C | T | intron | C14orf37 |  |  | Y |
| HCC-B1 | somaticSNP | 14 | 104199580 | A | C | 3-prime-UTR | ZFYVE21 | ZFYVE21 |  | Y |
| HCC-B1 | somaticINDEL | 15 | 41308142 | C | -A | intron | INO80 |  |  | N |
| HCC-B1 | somaticSNP | 15 | 42154041 | G | A | synonymous | SPTBN5 | SPTBN5 |  | N |
| HCC-B1 | somaticSNP | 15 | 63908503 | C | A | intron | HERC1 |  |  | N |
| HCC-B1 | somaticSNP | 15 | 64791958 | G | A | missense | ZNF609 | ZNF609 |  | Y |
| HCC-B1 | somaticINDEL | 15 | 83241984 | A | -T | intron | CPEB1 |  |  | Y |
| HCC-B1 | somaticSNP | 16 | 1259343 | C | T | synonymous | CACNA1H | CACNA1H |  | Y |
| HCC-B1 | somaticSNP | 16 | 2255888 | G | T | intron | MLST8 |  |  | N |
| HCC-B1 | somaticSNP | 16 | 2891338 | G | T | non-coding-exon | PRSS30P | PRSS30P |  | Y |
| HCC-B1 | somaticSNP | 16 | 7629905 | C | T | intron,missense | RBFOX1 | RBFOX1 |  | Y |
| HCC-B1 | somaticSNP | 16 | 69788684 | G | A | intron | NOB1 |  |  | Y |
| HCC-B1 | somaticSNP | 16 | 85120713 | A | C | missense | KIAA0513 | KIAA0513 |  | Y |
| HCC-B1 | somaticSNP | 17 | 6596268 | A | G | intron | SLC13A5 |  |  | Y |
| HCC-B1 | somaticSNP | 17 | 10411706 | A | G | missense | MYH1 | MYH1 |  | Y |
| HCC-B1 | somaticSNP | 17 | 21207939 | G | T | intron | MAP2K3 |  |  | Y |
| HCC-B1 | somaticSNP | 17 | 37824920 | C | T | intron,synonymous | PNMT | PNMT |  | Y |
| HCC-B1 | somaticSNP | 17 | 38078771 | T | C | 3-prime-UTR | ORMDL3 | ORMDL3 |  | N |
| HCC-B1 | somaticSNP | 17 | 38634492 | G | A | intron | TNS4 |  |  | Y |
| HCC-B1 | somaticSNP | 17 | 39534271 | A | T | 3-prime-UTR | KRT34 | KRT34 |  | N |
| HCC-B1 | somaticSNP | 17 | 40937100 | C | T | intron | WNK4 |  |  | Y |
| HCC-B1 | somaticINDEL | 17 | 56693799 | C | +T | intron | TEX14 |  |  | N |
| HCC-B1 | somaticSNP | 17 | 62777779 | C | T | non-coding-exon | LOC146880 | LOC146880 |  | Y |
| HCC-B1 | somaticSNP | 17 | 67170973 | G | T | intron | ABCA10 |  |  | N |
| HCC-B1 | somaticSNP | 17 | 72578342 | C | A | intron | CD300LD |  |  | Y |
| HCC-B1 | somaticSNP | 17 | 74079838 | A | G | intron-near-splice | EXOC7 |  |  | Y |
| HCC-B1 | somaticSNP | 17 | 74740318 | G | T | intron | MFSD11 |  |  | Y |
| HCC-B1 | somaticSNP | 17 | 78318589 | C | G | missense | RNF213 | RNF213 |  | Y |
| HCC-B1 | somaticSNP | 18 | 2567069 | T | C | intron,synonymous | METTL4 | METTL4 |  | Y |
| HCC-B1 | somaticSNP | 18 | 10857072 | C | A | missense | PIEZO2 | PIEZO2 |  | Y |
| HCC-B1 | somaticSNP | 18 | 63489374 | T | A | missense | CDH7 | CDH7 |  | Y |
| HCC-B1 | somaticSNP | 19 | 5746056 | T | C | synonymous | CATSPERD | CATSPERD |  | Y |
| HCC-B1 | somaticSNP | 19 | 7808968 | A | G | intron | CD209 |  |  | Y |
| HCC-B1 | somaticSNP | 19 | 9586051 | A | G | intron-near-splice | ZNF560 |  |  | N |
| HCC-B1 | somaticSNP | 19 | 10741732 | G | A | splice-acceptor | SLC44A2 | SLC44A2 |  | Y |
| HCC-B1 | somaticSNP | 19 | 15276007 | C | A | intron | NOTCH3 |  |  | N |
| HCC-B1 | somaticSNP | 19 | 16496069 | C | A | intron | EPS15L1 |  |  | N |
| HCC-B1 | somaticSNP | 19 | 38958392 | G | A | synonymous | RYR1 | RYR1 |  | N |
| HCC-B1 | somaticSNP | 19 | 40433693 | T | C | synonymous | FCGBP | FCGBP |  | Y |
| HCC-B1 | somaticSNP | 19 | 45147340 | G | T | 5-prime-UTR | PVR | PVR |  | N |
| HCC-B1 | somaticSNP | 19 | 47259006 | G | A | 5-prime-UTR,missense | FKRP | FKRP |  | Y |
| HCC-B1 | somaticSNP | 19 | 51919279 | A | G | intron,synonymous,missense | SIGLEC10,LOC100129083 | SIGLEC10,LOC100129083 |  | N |
| HCC-B1 | somaticSNP | 19 | 51919302 | T | C | intron,synonymous,missense | SIGLEC10,LOC100129083 | SIGLEC10,LOC100129083 |  | N |
| HCC-B1 | somaticSNP | 19 | 54485450 | G | A | missense | CACNG8 | CACNG8 |  | Y |
| HCC-B1 | somaticSNP | 2 | 21361803 | A | G | intergenic |  | |  | N |
| HCC-B1 | somaticSNP | 2 | 31412096 | T | C | intron | CAPN14 |  |  | Y |
| HCC-B1 | somaticSNP | 2 | 42511974 | G | A | intron | EML4 |  | EML4 | N |
| HCC-B1 | somaticSNP | 2 | 61697887 | C | G | upstream-gene |  | |  | Y |
| HCC-B1 | somaticSNP | 2 | 72960159 | T | C | intron | EXOC6B |  |  | N |
| HCC-B1 | somaticSNP | 2 | 175436749 | C | A | missense | WIPF1 | WIPF1 |  | N |
| HCC-B1 | somaticSNP | 2 | 178096766 | A | C | intron,intron-near-splice | NFE2L2 |  | NFE2L2 | Y |
| HCC-B1 | somaticSNP | 2 | 186667287 | T | A | synonymous | FSIP2 | FSIP2 |  | N |
| HCC-B1 | somaticINDEL | 2 | 201778717 | T | +A | intron | ORC2 |  |  | N |
| HCC-B1 | somaticSNP | 2 | 208773309 | C | A | intron-near-splice | PLEKHM3 |  |  | Y |
| HCC-B1 | somaticSNP | 2 | 242800900 | G | A | intron | PDCD1 |  |  | N |
| HCC-B1 | somaticSNP | 20 | 3219894 | A | G | intron | SLC4A11 |  |  | N |
| HCC-B1 | somaticSNP | 20 | 9346228 | G | A | intron | PLCB4 |  |  | Y |
| HCC-B1 | somaticSNP | 20 | 9449200 | C | T | intron | PLCB4 |  |  | N |
| HCC-B1 | somaticSNP | 20 | 14306910 | C | G | intron,missense | FLRT3,MACROD2 | FLRT3 |  | Y |
| HCC-B1 | somaticSNP | 20 | 40125973 | T | C | intron | CHD6 |  |  | Y |
| HCC-B1 | somaticSNP | 20 | 44486101 | G | A | upstream-gene |  | |  | N |
| HCC-B1 | somaticSNP | 22 | 23041026 | G | A | intergenic |  | |  | N |
| HCC-B1 | somaticSNP | 22 | 30050611 | A | G | intron | NF2 |  | NF2 | Y |
| HCC-B1 | somaticSNP | 3 | 443305 | T | G | intron-near-splice | CHL1 |  |  | Y |
| HCC-B1 | somaticSNP | 3 | 19322831 | G | A | intron | KCNH8 |  |  | Y |
| HCC-B1 | somaticSNP | 3 | 47807173 | G | T | intron | SMARCC1 |  |  | Y |
| HCC-B1 | somaticSNP | 3 | 52584712 | A | G | intron | PBRM1 |  | PBRM1 | N |
| HCC-B1 | somaticSNP | 3 | 69112049 | C | T | intron | UBA3 |  |  | Y |
| HCC-B1 | somaticSNP | 3 | 121132268 | T | A | intron | STXBP5L |  |  | N |
| HCC-B1 | somaticSNP | 3 | 142211929 | C | T | intron | ATR |  |  | Y |
| HCC-B1 | somaticINDEL | 3 | 150282061 | C | -T | intron | EIF2A |  |  | Y |
| HCC-B1 | somaticINDEL | 3 | 171065010 | G | +A | intron | TNIK |  |  | N |
| HCC-B1 | somaticSNP | 4 | 1244834 | G | A | non-coding-exon | CTBP1-AS2 | CTBP1-AS2 |  | Y |
| HCC-B1 | somaticSNP | 4 | 1818471 | G | A | synonymous | LETM1 | LETM1 |  | N |
| HCC-B1 | somaticSNP | 4 | 10020564 | G | A | intron | SLC2A9 |  |  | Y |
| HCC-B1 | somaticINDEL | 4 | 26241366 | G | +GGAGA | intron | RBPJ |  |  | N |
| HCC-B1 | somaticSNP | 4 | 103556316 | T | C | intron | MANBA |  |  | Y |
| HCC-B1 | somaticINDEL | 4 | 106317370 | T | +ATAAA | intron | PPA2 |  |  | N |
| HCC-B1 | somaticSNP | 5 | 7832063 | C | T | missense | C5orf49 | C5orf49 |  | Y |
| HCC-B1 | somaticSNP | 5 | 10382131 | A | G | intron | MARCH6 |  |  | Y |
| HCC-B1 | somaticINDEL | 5 | 78416973 | C | -A | intron | BHMT |  |  | N |
| HCC-B1 | somaticSNP | 5 | 118480380 | T | A | intron | DMXL1 |  |  | Y |
| HCC-B1 | somaticSNP | 5 | 128440932 | A | G | missense | ISOC1 | ISOC1 |  | Y |
| HCC-B1 | somaticSNP | 5 | 135396468 | T | A | intron | TGFBI |  |  | N |
| HCC-B1 | somaticSNP | 5 | 141005368 | G | A | intron | HDAC3 |  |  | Y |
| HCC-B1 | somaticSNP | 5 | 149112300 | T | G | intron | PPARGC1B |  |  | Y |
| HCC-B1 | somaticSNP | 6 | 5000779 | G | A | intron | RPP40 |  |  | Y |
| HCC-B1 | somaticSNP | 6 | 13228075 | T | G | synonymous | PHACTR1 | PHACTR1 |  | Y |
| HCC-B1 | somaticINDEL | 6 | 24651352 | T | -A | intron | TDP2 |  |  | Y |
| HCC-B1 | somaticSNP | 6 | 28197605 | C | T | intron | ZSCAN9 |  |  | Y |
| HCC-B1 | somaticSNP | 6 | 33659550 | C | T | intron | ITPR3 |  |  | N |
| HCC-B1 | somaticSNP | 6 | 43483097 | A | G | intron | YIPF3 |  |  | Y |
| HCC-B1 | somaticSNP | 6 | 54212153 | T | C | intron | TINAG |  |  | Y |
| HCC-B1 | somaticSNP | 6 | 73001594 | T | A | intron | RIMS1 |  |  | Y |
| HCC-B1 | somaticSNP | 6 | 75860786 | A | C | intron | COL12A1 |  |  | N |
| HCC-B1 | somaticSNP | 6 | 110048404 | G | T | stop-gained,intron | FIG4 | FIG4 |  | N |
| HCC-B1 | somaticSNP | 6 | 130425541 | T | G | intron | L3MBTL3 |  |  | N |
| HCC-B1 | somaticSNP | 6 | 139100991 | G | C | missense | CCDC28A | CCDC28A |  | Y |
| HCC-B1 | somaticSNP | 7 | 1914350 | A | G | intron | MAD1L1 |  |  | Y |
| HCC-B1 | somaticSNP | 7 | 38295939 | A | T | downstream-gene |  | |  | Y |
| HCC-B1 | somaticINDEL | 7 | 48412112 | T | -A | intron | ABCA13 |  |  | Y |
| HCC-B1 | somaticSNP | 7 | 82785265 | T | A | missense | PCLO | PCLO |  | Y |
| HCC-B1 | somaticSNP | 7 | 87808196 | T | A | intron | ADAM22 |  |  | Y |
| HCC-B1 | somaticSNP | 7 | 94248183 | A | G | synonymous | SGCE | SGCE |  | N |
| HCC-B1 | somaticSNP | 7 | 99578370 | T | G | upstream-gene |  | |  | N |
| HCC-B1 | somaticSNP | 7 | 99578381 | T | C | upstream-gene |  | |  | N |
| HCC-B1 | somaticSNP | 7 | 99578411 | T | C | non-coding-exon | AZGP1P1 | AZGP1P1 |  | Y |
| HCC-B1 | somaticSNP | 7 | 103053467 | A | G | missense | SLC26A5 | SLC26A5 |  | Y |
| HCC-B1 | somaticSNP | 7 | 113519608 | A | T | missense | PPP1R3A | PPP1R3A |  | Y |
| HCC-B1 | somaticSNP | 7 | 113520039 | A | G | missense | PPP1R3A | PPP1R3A |  | Y |
| HCC-B1 | somaticSNP | 8 | 6500615 | C | T | 3-prime-UTR | MCPH1 | MCPH1 |  | N |
| HCC-B1 | somaticSNP | 8 | 8234211 | C | T | missense | SGK223 | SGK223 |  | Y |
| HCC-B1 | somaticSNP | 8 | 52336073 | T | G | intron | PXDNL |  |  | Y |
| HCC-B1 | somaticSNP | 8 | 52384961 | T | A | intron | PXDNL |  |  | Y |
| HCC-B1 | somaticSNP | 8 | 95894920 | T | C | intron | CCNE2 |  |  | N |
| HCC-B1 | somaticSNP | 8 | 116426272 | T | C | synonymous | TRPS1 | TRPS1 |  | Y |
| HCC-B1 | somaticSNP | 8 | 133769672 | A | T | intron | TMEM71 |  |  | Y |
| HCC-B1 | somaticSNP | 9 | 2651521 | C | A | intron | VLDLR |  |  | Y |
| HCC-B1 | somaticSNP | 9 | 19087295 | T | C | intron | HAUS6 |  |  | Y |
| HCC-B1 | somaticSNP | 9 | 21409818 | C | G | 3-prime-UTR | IFNA8 | IFNA8 |  | Y |
| HCC-B1 | somaticSNP | 9 | 88207477 | G | T | missense | AGTPBP1 | AGTPBP1 |  | N |
| HCC-B1 | somaticSNP | 9 | 125909298 | G | A | synonymous,non-coding-exon | STRBP | STRBP |  | Y |
| HCC-B1 | somaticSNP | X | 48855744 | A | C | intron | GRIPAP1 |  |  | Y |
| HCC-B1 | somaticSNP | X | 69669167 | C | T | intron | DLG3 |  |  | Y |
| HCC-B1 | somaticSNP | X | 101858685 | T | G | stop-gained,intron | ARMCX5,ARMCX5-GPRASP2 | ARMCX5 |  | Y |
| HCC-B1 | somaticSNP | X | 140996400 | G | C | synonymous | MAGEC1 | MAGEC1 |  | Y |
| HCC-B1 | somaticSNP | 10 | 26462949 | G | T | missense | MYO3A | MYO3A |  | N |
| HCC-B1 | somaticSNP | 1 | 201009442 | G | T | missense | CACNA1S | CACNA1S |  | Y |
| HCC-B1 | somaticSNP | 1 | 211751845 | A | C | missense | SLC30A1 | SLC30A1 |  | Y |
| HCC-B1 | somaticSNP | 11 | 4661742 | T | A | missense | OR51D1 | OR51D1 |  | N |
| HCC-B1 | somaticSNP | 11 | 71806007 | T | C | 5-prime-UTR,intron,missense,non-coding-exon | LRTOMT | LRTOMT |  | N |
| HCC-B1 | somaticSNP | 12 | 123276650 | G | A | missense,non-coding-exon | CCDC62 | CCDC62 |  | N |
| HCC-B1 | somaticSNP | 15 | 42458777 | C | A | missense | VPS39 | VPS39 |  | N |
| HCC-B1 | somaticSNP | 17 | 76989903 | T | C | missense | CANT1 | CANT1 | CANT1 | N |
| HCC-B1 | somaticSNP | 19 | 9000446 | C | T | missense | MUC16 | MUC16 |  | N |
| HCC-B1 | somaticSNP | 19 | 10476488 | C | A | missense | TYK2 | TYK2 |  | N |
| HCC-B1 | somaticSNP | 21 | 19167551 | T | C | intron,missense | C21orf91 | C21orf91 |  | N |
| HCC-B1 | somaticSNP | 4 | 126412781 | A | C | missense | FAT4 | FAT4 |  | N |
| HCC-B1 | somaticSNP | 5 | 33649003 | G | A | missense | ADAMTS12 | ADAMTS12 |  | Y |
| HCC-B1 | somaticSNP | 7 | 30830809 | G | A | missense,non-coding-exon | INMT-FAM188B,FAM188B | INMT-FAM188B,FAM188B |  | N |
| HCC-B1 | somaticSNP | 9 | 1056448 | C | A | stop-gained,3-prime-UTR | DMRT2 | DMRT2 |  | N |
| HCC-B2 | somaticSNP | 15 | 79304864 | G | T | intron,missense | RASGRF1 | RASGRF1 |  | N |
| HCC-B2 | somaticSNP | 17 | 78318589 | C | G | missense | RNF213 | RNF213 |  | N |
| HCC-B2 | somaticSNP | 1 | 17023483 | C | T | intron | ESPNP |  |  | Y |
| HCC-B2 | somaticSNP | 1 | 17267496 | C | T | intron | CROCC |  |  | N |
| HCC-B2 | somaticSNP | 1 | 26873700 | C | T | 5-prime-UTR,synonymous | RPS6KA1 | RPS6KA1 |  | N |
| HCC-B2 | somaticSNP | 1 | 36028719 | C | T | intron | NCDN |  |  | Y |
| HCC-B2 | somaticSNP | 1 | 41327871 | C | A | 5-prime-UTR | CITED4 | CITED4 |  | N |
| HCC-B2 | somaticINDEL | 1 | 62350123 | G | -TTTTT | intron | INADL |  |  | N |
| HCC-B2 | somaticSNP | 1 | 67861900 | C | A | 3-prime-UTR,non-coding-exon | IL12RB2 | IL12RB2 |  | Y |
| HCC-B2 | somaticSNP | 1 | 85431403 | G | C | intron | MCOLN2 |  |  | N |
| HCC-B2 | somaticSNP | 1 | 86430684 | T | A | intron-near-splice | COL24A1 |  |  | Y |
| HCC-B2 | somaticINDEL | 1 | 100680593 | C | +T | intron | DBT |  |  | N |
| HCC-B2 | somaticSNP | 1 | 109813860 | C | T | synonymous | CELSR2 | CELSR2 |  | N |
| HCC-B2 | somaticSNP | 1 | 157716473 | C | T | 3-prime-UTR | FCRL2 | FCRL2 |  | Y |
| HCC-B2 | somaticSNP | 1 | 158609897 | G | C | intron | SPTA1 |  |  | Y |
| HCC-B2 | somaticSNP | 1 | 158746420 | A | T | downstream-gene |  | |  | Y |
| HCC-B2 | somaticSNP | 1 | 159824488 | A | G | 3-prime-UTR,intron | VSIG8,C1orf204 | VSIG8 |  | Y |
| HCC-B2 | somaticSNP | 1 | 167742442 | A | G | intron | MPZL1 |  |  | Y |
| HCC-B2 | somaticSNP | 1 | 181700435 | C | A | intron | CACNA1E |  |  | Y |
| HCC-B2 | somaticSNP | 1 | 182443101 | A | T | missense | RGSL1 | RGSL1 |  | Y |
| HCC-B2 | somaticSNP | 1 | 205034434 | G | T | intron | CNTN2 |  |  | Y |
| HCC-B2 | somaticSNP | 1 | 227227740 | A | C | intron | CDC42BPA |  |  | Y |
| HCC-B2 | somaticSNP | 1 | 245703873 | T | C | intron | KIF26B |  |  | Y |
| HCC-B2 | somaticSNP | 1 | 247051591 | C | A | intron | AHCTF1 |  |  | Y |
| HCC-B2 | somaticSNP | 10 | 29581055 | T | C | intron | LYZL1 |  |  | N |
| HCC-B2 | somaticINDEL | 10 | 60160134 | T | -AAA | intergenic |  | |  | N |
| HCC-B2 | somaticSNP | 10 | 75090988 | T | A | missense | TTC18 | TTC18 |  | N |
| HCC-B2 | somaticSNP | 10 | 82331347 | A | G | intron | SH2D4B |  |  | Y |
| HCC-B2 | somaticINDEL | 10 | 93776240 | C | +T | intron | BTAF1 |  |  | N |
| HCC-B2 | somaticINDEL | 10 | 95454768 | T | -A | intron | FRA10AC1 |  |  | Y |
| HCC-B2 | somaticSNP | 10 | 118671072 | G | C | intron | KIAA1598 |  |  | N |
| HCC-B2 | somaticSNP | 10 | 134188508 | C | T | intron | LRRC27 |  |  | N |
| HCC-B2 | somaticSNP | 11 | 4661742 | T | A | missense | OR51D1 | OR51D1 |  | Y |
| HCC-B2 | somaticSNP | 11 | 18750380 | G | C | 3-prime-UTR | PTPN5 | PTPN5 |  | Y |
| HCC-B2 | somaticSNP | 11 | 20940973 | C | A | intron | NELL1 |  |  | N |
| HCC-B2 | somaticSNP | 11 | 56086317 | T | A | missense | OR8K3 | OR8K3 |  | Y |
| HCC-B2 | somaticSNP | 11 | 65988562 | A | T | intron | PACS1 |  |  | N |
| HCC-B2 | somaticSNP | 11 | 68213996 | G | A | synonymous | LRP5 | LRP5 |  | N |
| HCC-B2 | somaticSNP | 11 | 71806007 | T | C | 5-prime-UTR,intron,missense,non-coding-exon | LRTOMT | LRTOMT |  | Y |
| HCC-B2 | somaticSNP | 11 | 73536698 | A | C | intron | MRPL48 |  |  | N |
| HCC-B2 | somaticSNP | 11 | 101375549 | T | C | intron | TRPC6 |  |  | Y |
| HCC-B2 | somaticSNP | 11 | 122647728 | A | T | intron-near-splice | UBASH3B |  |  | Y |
| HCC-B2 | somaticSNP | 12 | 21695584 | A | G | intron | GYS2 |  |  | Y |
| HCC-B2 | somaticSNP | 12 | 31255227 | A | G | synonymous | DDX11 | DDX11 |  | Y |
| HCC-B2 | somaticSNP | 12 | 51451772 | A | G | intron | LETMD1 |  |  | N |
| HCC-B2 | somaticSNP | 12 | 53238112 | C | T | intron | KRT78 |  |  | N |
| HCC-B2 | somaticSNP | 12 | 95915034 | C | G | intron | USP44 |  |  | N |
| HCC-B2 | somaticINDEL | 12 | 97311376 | C | -T | intron | NEDD1 |  |  | N |
| HCC-B2 | somaticSNP | 12 | 123276650 | G | A | missense,non-coding-exon | CCDC62 | CCDC62 |  | Y |
| HCC-B2 | somaticSNP | 13 | 43788226 | A | G | missense | ENOX1 | ENOX1 |  | Y |
| HCC-B2 | somaticSNP | 13 | 47470954 | C | A | 5-prime-UTR,missense | HTR2A | HTR2A |  | N |
| HCC-B2 | somaticSNP | 13 | 114309228 | A | T | missense | ATP4B | ATP4B |  | Y |
| HCC-B2 | somaticSNP | 14 | 23733725 | C | T | intron | C14orf164 |  |  | Y |
| HCC-B2 | somaticSNP | 14 | 45639980 | T | C | intron | FANCM |  |  | Y |
| HCC-B2 | somaticSNP | 14 | 56079145 | G | T | stop-gained,non-coding-exon | KTN1 | KTN1 | KTN1 | N |
| HCC-B2 | somaticSNP | 14 | 58583074 | C | T | intron | C14orf37 |  |  | N |
| HCC-B2 | somaticINDEL | 15 | 41308142 | C | -A | intron | INO80 |  |  | N |
| HCC-B2 | somaticSNP | 15 | 42154041 | G | A | synonymous | SPTBN5 | SPTBN5 |  | Y |
| HCC-B2 | somaticSNP | 15 | 42458777 | C | A | missense | VPS39 | VPS39 |  | N |
| HCC-B2 | somaticSNP | 15 | 64791958 | G | A | missense | ZNF609 | ZNF609 |  | Y |
| HCC-B2 | somaticINDEL | 15 | 83241984 | A | -T | intron | CPEB1 |  |  | N |
| HCC-B2 | somaticSNP | 16 | 1259343 | C | T | synonymous | CACNA1H | CACNA1H |  | N |
| HCC-B2 | somaticSNP | 16 | 2255888 | G | T | intron | MLST8 |  |  | Y |
| HCC-B2 | somaticSNP | 16 | 2891338 | G | T | non-coding-exon | PRSS30P | PRSS30P |  | Y |
| HCC-B2 | somaticSNP | 16 | 11556121 | A | G | synonymous | LOC400499 |  |  | Y |
| HCC-B2 | somaticSNP | 16 | 69788684 | G | A | intron | NOB1 |  |  | Y |
| HCC-B2 | somaticSNP | 17 | 6596268 | A | G | intron | SLC13A5 |  |  | N |
| HCC-B2 | somaticSNP | 17 | 10411706 | A | G | missense | MYH1 | MYH1 |  | N |
| HCC-B2 | somaticSNP | 17 | 21207939 | G | T | intron | MAP2K3 |  |  | Y |
| HCC-B2 | somaticSNP | 17 | 37824920 | C | T | intron,synonymous | PNMT | PNMT |  | Y |
| HCC-B2 | somaticSNP | 17 | 38078771 | T | C | 3-prime-UTR | ORMDL3 | ORMDL3 |  | Y |
| HCC-B2 | somaticSNP | 17 | 38634492 | G | A | intron | TNS4 |  |  | N |
| HCC-B2 | somaticSNP | 17 | 39534271 | A | T | 3-prime-UTR | KRT34 | KRT34 |  | N |
| HCC-B2 | somaticSNP | 17 | 40937100 | C | T | intron | WNK4 |  |  | Y |
| HCC-B2 | somaticSNP | 17 | 62777779 | C | T | non-coding-exon | LOC146880 | LOC146880 |  | Y |
| HCC-B2 | somaticSNP | 17 | 67170973 | G | T | intron | ABCA10 |  |  | Y |
| HCC-B2 | somaticSNP | 17 | 72578342 | C | A | intron | CD300LD |  |  | Y |
| HCC-B2 | somaticSNP | 17 | 74079838 | A | G | intron-near-splice | EXOC7 |  |  | N |
| HCC-B2 | somaticSNP | 17 | 74740318 | G | T | intron | MFSD11 |  |  | Y |
| HCC-B2 | somaticSNP | 18 | 2567069 | T | C | intron,synonymous | METTL4 | METTL4 |  | Y |
| HCC-B2 | somaticSNP | 18 | 63489374 | T | A | missense | CDH7 | CDH7 |  | N |
| HCC-B2 | somaticSNP | 19 | 5746056 | T | C | synonymous | CATSPERD | CATSPERD |  | N |
| HCC-B2 | somaticSNP | 19 | 7808968 | A | G | intron | CD209 |  |  | N |
| HCC-B2 | somaticSNP | 19 | 9000446 | C | T | missense | MUC16 | MUC16 |  | Y |
| HCC-B2 | somaticSNP | 19 | 9586051 | A | G | intron-near-splice | ZNF560 |  |  | N |
| HCC-B2 | somaticSNP | 19 | 10476488 | C | A | missense | TYK2 | TYK2 |  | Y |
| HCC-B2 | somaticSNP | 19 | 10741732 | G | A | splice-acceptor | SLC44A2 | SLC44A2 |  | N |
| HCC-B2 | somaticSNP | 19 | 15276007 | C | A | intron | NOTCH3 |  |  | N |
| HCC-B2 | somaticSNP | 19 | 16496069 | C | A | intron | EPS15L1 |  |  | N |
| HCC-B2 | somaticSNP | 19 | 38958392 | G | A | synonymous | RYR1 | RYR1 |  | Y |
| HCC-B2 | somaticSNP | 19 | 40433693 | T | C | synonymous | FCGBP | FCGBP |  | N |
| HCC-B2 | somaticSNP | 19 | 47259006 | G | A | 5-prime-UTR,missense | FKRP | FKRP |  | Y |
| HCC-B2 | somaticSNP | 19 | 50463727 | G | A | intron | SIGLEC11 |  |  | Y |
| HCC-B2 | somaticSNP | 19 | 51919279 | A | G | intron,synonymous,missense | SIGLEC10,LOC100129083 | SIGLEC10,LOC100129083 |  | Y |
| HCC-B2 | somaticSNP | 19 | 51919302 | T | C | intron,synonymous,missense | SIGLEC10,LOC100129083 | SIGLEC10,LOC100129083 |  | N |
| HCC-B2 | somaticSNP | 2 | 21361803 | A | G | intergenic |  | |  | N |
| HCC-B2 | somaticSNP | 2 | 31412096 | T | C | intron | CAPN14 |  |  | Y |
| HCC-B2 | somaticSNP | 2 | 42511974 | G | A | intron | EML4 |  | EML4 | N |
| HCC-B2 | somaticSNP | 2 | 61697887 | C | G | upstream-gene |  | |  | Y |
| HCC-B2 | somaticSNP | 2 | 72960159 | T | C | intron | EXOC6B |  |  | Y |
| HCC-B2 | somaticSNP | 2 | 175436749 | C | A | missense | WIPF1 | WIPF1 |  | Y |
| HCC-B2 | somaticSNP | 2 | 178096766 | A | C | intron,intron-near-splice | NFE2L2 |  | NFE2L2 | Y |
| HCC-B2 | somaticSNP | 2 | 186667287 | T | A | synonymous | FSIP2 | FSIP2 |  | N |
| HCC-B2 | somaticSNP | 2 | 208773309 | C | A | intron-near-splice | PLEKHM3 |  |  | Y |
| HCC-B2 | somaticSNP | 2 | 242800900 | G | A | intron | PDCD1 |  |  | N |
| HCC-B2 | somaticSNP | 20 | 3219894 | A | G | intron | SLC4A11 |  |  | N |
| HCC-B2 | somaticSNP | 20 | 9346228 | G | A | intron | PLCB4 |  |  | Y |
| HCC-B2 | somaticSNP | 20 | 9449200 | C | T | intron | PLCB4 |  |  | N |
| HCC-B2 | somaticSNP | 20 | 40125973 | T | C | intron | CHD6 |  |  | Y |
| HCC-B2 | somaticSNP | 20 | 44486101 | G | A | upstream-gene |  | |  | Y |
| HCC-B2 | somaticSNP | 21 | 19167551 | T | C | intron,missense | C21orf91 | C21orf91 |  | Y |
| HCC-B2 | somaticSNP | 22 | 23041026 | G | A | intergenic |  | |  | N |
| HCC-B2 | somaticSNP | 22 | 30050611 | A | G | intron | NF2 |  | NF2 | Y |
| HCC-B2 | somaticSNP | 3 | 443305 | T | G | intron-near-splice | CHL1 |  |  | Y |
| HCC-B2 | somaticSNP | 3 | 19322831 | G | A | intron | KCNH8 |  |  | N |
| HCC-B2 | somaticSNP | 3 | 47807173 | G | T | intron | SMARCC1 |  |  | N |
| HCC-B2 | somaticSNP | 3 | 52584712 | A | G | intron | PBRM1 |  | PBRM1 | Y |
| HCC-B2 | somaticSNP | 3 | 69112049 | C | T | intron | UBA3 |  |  | N |
| HCC-B2 | somaticSNP | 3 | 121132268 | T | A | intron | STXBP5L |  |  | N |
| HCC-B2 | somaticSNP | 3 | 124807132 | C | T | intron | SLC12A8 |  |  | N |
| HCC-B2 | somaticINDEL | 3 | 171065010 | G | +A | intron | TNIK |  |  | N |
| HCC-B2 | somaticSNP | 4 | 1244834 | G | A | non-coding-exon | CTBP1-AS2 | CTBP1-AS2 |  | N |
| HCC-B2 | somaticSNP | 4 | 1818471 | G | A | synonymous | LETM1 | LETM1 |  | Y |
| HCC-B2 | somaticSNP | 4 | 10020564 | G | A | intron | SLC2A9 |  |  | N |
| HCC-B2 | somaticINDEL | 4 | 26241366 | G | +GGAGA | intron | RBPJ |  |  | N |
| HCC-B2 | somaticSNP | 4 | 103556316 | T | C | intron | MANBA |  |  | N |
| HCC-B2 | somaticINDEL | 4 | 106317370 | T | +ATAAA | intron | PPA2 |  |  | Y |
| HCC-B2 | somaticSNP | 5 | 7832063 | C | T | missense | C5orf49 | C5orf49 |  | Y |
| HCC-B2 | somaticSNP | 5 | 10382131 | A | G | intron | MARCH6 |  |  | N |
| HCC-B2 | somaticINDEL | 5 | 78416973 | C | -A | intron | BHMT |  |  | N |
| HCC-B2 | somaticSNP | 5 | 118480380 | T | A | intron | DMXL1 |  |  | Y |
| HCC-B2 | somaticSNP | 5 | 135396468 | T | A | intron | TGFBI |  |  | Y |
| HCC-B2 | somaticSNP | 5 | 141005368 | G | A | intron | HDAC3 |  |  | Y |
| HCC-B2 | somaticSNP | 5 | 149112300 | T | G | intron | PPARGC1B |  |  | Y |
| HCC-B2 | somaticSNP | 6 | 13228075 | T | G | synonymous | PHACTR1 | PHACTR1 |  | Y |
| HCC-B2 | somaticSNP | 6 | 28197605 | C | T | intron | ZSCAN9 |  |  | Y |
| HCC-B2 | somaticSNP | 6 | 33659550 | C | T | intron | ITPR3 |  |  | Y |
| HCC-B2 | somaticSNP | 6 | 43483097 | A | G | intron | YIPF3 |  |  | N |
| HCC-B2 | somaticSNP | 6 | 46672392 | C | T | missense | PLA2G7 | PLA2G7 |  | Y |
| HCC-B2 | somaticSNP | 6 | 54212153 | T | C | intron | TINAG |  |  | N |
| HCC-B2 | somaticSNP | 6 | 73001594 | T | A | intron | RIMS1 |  |  | Y |
| HCC-B2 | somaticSNP | 6 | 75860786 | A | C | intron | COL12A1 |  |  | N |
| HCC-B2 | somaticSNP | 6 | 130425541 | T | G | intron | L3MBTL3 |  |  | N |
| HCC-B2 | somaticSNP | 6 | 139100991 | G | C | missense | CCDC28A | CCDC28A |  | N |
| HCC-B2 | somaticINDEL | 7 | 22330862 | T | +A | intron | RAPGEF5 |  |  | N |
| HCC-B2 | somaticSNP | 7 | 30830809 | G | A | missense,non-coding-exon | INMT-FAM188B,FAM188B | INMT-FAM188B,FAM188B |  | Y |
| HCC-B2 | somaticSNP | 7 | 38295939 | A | T | downstream-gene |  | |  | Y |
| HCC-B2 | somaticINDEL | 7 | 48412112 | T | -A | intron | ABCA13 |  |  | N |
| HCC-B2 | somaticSNP | 7 | 94248183 | A | G | synonymous | SGCE | SGCE |  | Y |
| HCC-B2 | somaticSNP | 7 | 99578411 | T | C | non-coding-exon | AZGP1P1 | AZGP1P1 |  | Y |
| HCC-B2 | somaticSNP | 7 | 113519608 | A | T | missense | PPP1R3A | PPP1R3A |  | Y |
| HCC-B2 | somaticSNP | 7 | 113520039 | A | G | missense | PPP1R3A | PPP1R3A |  | Y |
| HCC-B2 | somaticSNP | 8 | 6500615 | C | T | 3-prime-UTR | MCPH1 | MCPH1 |  | Y |
| HCC-B2 | somaticSNP | 8 | 8234211 | C | T | missense | SGK223 | SGK223 |  | Y |
| HCC-B2 | somaticSNP | 8 | 52336073 | T | G | intron | PXDNL |  |  | Y |
| HCC-B2 | somaticSNP | 8 | 95894920 | T | C | intron | CCNE2 |  |  | N |
| HCC-B2 | somaticSNP | 8 | 116426272 | T | C | synonymous | TRPS1 | TRPS1 |  | N |
| HCC-B2 | somaticSNP | 8 | 133769672 | A | T | intron | TMEM71 |  |  | Y |
| HCC-B2 | somaticSNP | 8 | 146220171 | T | C | intron | ZNF252P |  |  | N |
| HCC-B2 | somaticSNP | 9 | 1056448 | C | A | stop-gained,3-prime-UTR | DMRT2 | DMRT2 |  | Y |
| HCC-B2 | somaticSNP | 9 | 2651521 | C | A | intron | VLDLR |  |  | Y |
| HCC-B2 | somaticSNP | 9 | 19087295 | T | C | intron | HAUS6 |  |  | N |
| HCC-B2 | somaticSNP | 9 | 21409818 | C | G | 3-prime-UTR | IFNA8 | IFNA8 |  | N |
| HCC-B2 | somaticSNP | 9 | 88207477 | G | T | missense | AGTPBP1 | AGTPBP1 |  | N |
| HCC-B2 | somaticSNP | 9 | 96285612 | C | T | intron,missense | FAM120A |  |  | Y |
| HCC-B2 | somaticSNP | 9 | 125909298 | G | A | synonymous,non-coding-exon | STRBP | STRBP |  | N |
| HCC-B2 | somaticSNP | X | 48855744 | A | C | intron | GRIPAP1 |  |  | Y |
| HCC-B2 | somaticSNP | X | 69669167 | C | T | intron | DLG3 |  |  | Y |
| HCC-B2 | somaticSNP | X | 101858685 | T | G | stop-gained,intron | ARMCX5,ARMCX5-GPRASP2 | ARMCX5 |  | Y |
| HCC-B2 | somaticSNP | X | 140996400 | G | C | synonymous | MAGEC1 | MAGEC1 |  | Y |
| HCC-B2 | somaticSNP | 18 | 10857072 | C | A | missense | PIEZO2 | PIEZO2 |  | N |
| HCC-B2 | somaticSNP | 10 | 26462949 | G | T | missense | MYO3A | MYO3A |  | N |
| HCC-B2 | somaticSNP | 20 | 14306910 | C | G | intron,missense | FLRT3,MACROD2 | FLRT3 |  | Y |
| HCC-B2 | somaticSNP | 1 | 44363924 | A | G | intron,missense,non-coding-exon | ST3GAL3 | ST3GAL3 |  | N |
| HCC-B2 | somaticSNP | 1 | 201009442 | G | T | missense | CACNA1S | CACNA1S |  | Y |
| HCC-B2 | somaticSNP | 1 | 211751845 | A | C | missense | SLC30A1 | SLC30A1 |  | Y |
| HCC-B2 | somaticSNP | 12 | 40258589 | G | C | missense | SLC2A13 | SLC2A13 |  | N |
| HCC-B2 | somaticSNP | 16 | 7629905 | C | T | intron,missense | RBFOX1 | RBFOX1 |  | N |
| HCC-B2 | somaticSNP | 16 | 85120713 | A | C | missense | KIAA0513 | KIAA0513 |  | N |
| HCC-B2 | somaticSNP | 17 | 76989903 | T | C | missense | CANT1 | CANT1 | CANT1 | N |
| HCC-B2 | somaticSNP | 19 | 54485450 | G | A | missense | CACNG8 | CACNG8 |  | N |
| HCC-B2 | somaticSNP | 4 | 126412781 | A | C | missense | FAT4 | FAT4 |  | N |
| HCC-B2 | somaticSNP | 5 | 33649003 | G | A | missense | ADAMTS12 | ADAMTS12 |  | N |
| HCC-B2 | somaticSNP | 5 | 128440932 | A | G | missense | ISOC1 | ISOC1 |  | Y |
| HCC-B2 | somaticSNP | 6 | 110048404 | G | T | stop-gained,intron | FIG4 | FIG4 |  | N |
| HCC-B2 | somaticSNP | 7 | 82785265 | T | A | missense | PCLO | PCLO |  | N |
| HCC-B2 | somaticSNP | 7 | 103053467 | A | G | missense | SLC26A5 | SLC26A5 |  | N |
| HCC-B3 | somaticSNP | 17 | 78318589 | C | G | missense | RNF213 | RNF213 |  | N |
| HCC-B3 | somaticSNP | 7 | 151970859 | C | T | missense | KMT2C | KMT2C | KMT2C | Y |
| HCC-B3 | somaticSNP | 1 | 17023483 | C | T | intron | ESPNP |  |  | N |
| HCC-B3 | somaticSNP | 1 | 17267496 | C | T | intron | CROCC |  |  | N |
| HCC-B3 | somaticINDEL | 1 | 25780889 | A | +TG | intron | TMEM57 |  |  | N |
| HCC-B3 | somaticSNP | 1 | 26873700 | C | T | 5-prime-UTR,synonymous | RPS6KA1 | RPS6KA1 |  | Y |
| HCC-B3 | somaticSNP | 1 | 29611123 | C | T | intron | PTPRU |  |  | N |
| HCC-B3 | somaticSNP | 1 | 36028719 | C | T | intron | NCDN |  |  | Y |
| HCC-B3 | somaticSNP | 1 | 41327871 | C | A | 5-prime-UTR | CITED4 | CITED4 |  | Y |
| HCC-B3 | somaticSNP | 1 | 44363924 | A | G | intron,missense,non-coding-exon | ST3GAL3 | ST3GAL3 |  | Y |
| HCC-B3 | somaticINDEL | 1 | 62350123 | G | -TTTTT | intron | INADL |  |  | Y |
| HCC-B3 | somaticSNP | 1 | 67861900 | C | A | 3-prime-UTR,non-coding-exon | IL12RB2 | IL12RB2 |  | Y |
| HCC-B3 | somaticSNP | 1 | 85431403 | G | C | intron | MCOLN2 |  |  | Y |
| HCC-B3 | somaticSNP | 1 | 86430684 | T | A | intron-near-splice | COL24A1 |  |  | Y |
| HCC-B3 | somaticINDEL | 1 | 93159821 | C | -A | intron | EVI5 |  |  | N |
| HCC-B3 | somaticINDEL | 1 | 100680593 | C | +T | intron | DBT |  |  | N |
| HCC-B3 | somaticSNP | 1 | 109813860 | C | T | synonymous | CELSR2 | CELSR2 |  | Y |
| HCC-B3 | somaticSNP | 1 | 157716473 | C | T | 3-prime-UTR | FCRL2 | FCRL2 |  | Y |
| HCC-B3 | somaticSNP | 1 | 158609897 | G | C | intron | SPTA1 |  |  | Y |
| HCC-B3 | somaticSNP | 1 | 158746420 | A | T | downstream-gene |  | |  | Y |
| HCC-B3 | somaticSNP | 1 | 159824488 | A | G | 3-prime-UTR,intron | VSIG8,C1orf204 | VSIG8 |  | Y |
| HCC-B3 | somaticSNP | 1 | 167742442 | A | G | intron | MPZL1 |  |  | Y |
| HCC-B3 | somaticSNP | 1 | 181700435 | C | A | intron | CACNA1E |  |  | Y |
| HCC-B3 | somaticINDEL | 1 | 193202032 | C | +A | intron | CDC73 |  | CDC73 | N |
| HCC-B3 | somaticSNP | 1 | 201009442 | G | T | missense | CACNA1S | CACNA1S |  | Y |
| HCC-B3 | somaticSNP | 1 | 205034434 | G | T | intron | CNTN2 |  |  | N |
| HCC-B3 | somaticSNP | 1 | 211751845 | A | C | missense | SLC30A1 | SLC30A1 |  | Y |
| HCC-B3 | somaticSNP | 1 | 227227740 | A | C | intron | CDC42BPA |  |  | Y |
| HCC-B3 | somaticSNP | 1 | 245703873 | T | C | intron | KIF26B |  |  | Y |
| HCC-B3 | somaticSNP | 1 | 247051591 | C | A | intron | AHCTF1 |  |  | Y |
| HCC-B3 | somaticSNP | 10 | 26462949 | G | T | missense | MYO3A | MYO3A |  | Y |
| HCC-B3 | somaticSNP | 10 | 29581055 | T | C | intron | LYZL1 |  |  | N |
| HCC-B3 | somaticINDEL | 10 | 60160134 | T | -AAA | intergenic |  | |  | N |
| HCC-B3 | somaticSNP | 10 | 75090988 | T | A | missense | TTC18 | TTC18 |  | N |
| HCC-B3 | somaticSNP | 10 | 82331347 | A | G | intron | SH2D4B |  |  | Y |
| HCC-B3 | somaticINDEL | 10 | 93776240 | C | +T | intron | BTAF1 |  |  | Y |
| HCC-B3 | somaticINDEL | 10 | 95454768 | T | -A | intron | FRA10AC1 |  |  | Y |
| HCC-B3 | somaticSNP | 10 | 100004119 | G | T | 3-prime-UTR | R3HCC1L | R3HCC1L |  | Y |
| HCC-B3 | somaticSNP | 10 | 118671072 | G | C | intron | KIAA1598 |  |  | N |
| HCC-B3 | somaticSNP | 10 | 134188508 | C | T | intron | LRRC27 |  |  | Y |
| HCC-B3 | somaticSNP | 11 | 4661742 | T | A | missense | OR51D1 | OR51D1 |  | Y |
| HCC-B3 | somaticSNP | 11 | 18750380 | G | C | 3-prime-UTR | PTPN5 | PTPN5 |  | Y |
| HCC-B3 | somaticSNP | 11 | 20940973 | C | A | intron | NELL1 |  |  | Y |
| HCC-B3 | somaticSNP | 11 | 56086317 | T | A | missense | OR8K3 | OR8K3 |  | Y |
| HCC-B3 | somaticSNP | 11 | 65988562 | A | T | intron | PACS1 |  |  | Y |
| HCC-B3 | somaticSNP | 11 | 68213996 | G | A | synonymous | LRP5 | LRP5 |  | Y |
| HCC-B3 | somaticSNP | 11 | 71806007 | T | C | 5-prime-UTR,intron,missense,non-coding-exon | LRTOMT | LRTOMT |  | Y |
| HCC-B3 | somaticSNP | 11 | 73536698 | A | C | intron | MRPL48 |  |  | Y |
| HCC-B3 | somaticSNP | 11 | 101375549 | T | C | intron | TRPC6 |  |  | N |
| HCC-B3 | somaticSNP | 11 | 122647728 | A | T | intron-near-splice | UBASH3B |  |  | Y |
| HCC-B3 | somaticSNP | 12 | 21695584 | A | G | intron | GYS2 |  |  | Y |
| HCC-B3 | somaticSNP | 12 | 40258589 | G | C | missense | SLC2A13 | SLC2A13 |  | Y |
| HCC-B3 | somaticSNP | 12 | 51451772 | A | G | intron | LETMD1 |  |  | Y |
| HCC-B3 | somaticSNP | 12 | 53238112 | C | T | intron | KRT78 |  |  | N |
| HCC-B3 | somaticSNP | 12 | 95915034 | C | G | intron | USP44 |  |  | N |
| HCC-B3 | somaticINDEL | 12 | 97311376 | C | -T | intron | NEDD1 |  |  | N |
| HCC-B3 | somaticSNP | 12 | 123276650 | G | A | missense,non-coding-exon | CCDC62 | CCDC62 |  | Y |
| HCC-B3 | somaticSNP | 13 | 20277413 | T | C | missense | PSPC1 | PSPC1 |  | N |
| HCC-B3 | somaticSNP | 13 | 47470954 | C | A | 5-prime-UTR,missense | HTR2A | HTR2A |  | Y |
| HCC-B3 | somaticSNP | 13 | 99540395 | A | G | intron | DOCK9 |  |  | N |
| HCC-B3 | somaticSNP | 14 | 23733725 | C | T | intron | C14orf164 |  |  | Y |
| HCC-B3 | somaticSNP | 14 | 45639980 | T | C | intron | FANCM |  |  | Y |
| HCC-B3 | somaticSNP | 14 | 58583074 | C | T | intron | C14orf37 |  |  | Y |
| HCC-B3 | somaticSNP | 14 | 104199580 | A | C | 3-prime-UTR | ZFYVE21 | ZFYVE21 |  | Y |
| HCC-B3 | somaticINDEL | 15 | 41308142 | C | -A | intron | INO80 |  |  | N |
| HCC-B3 | somaticSNP | 15 | 42154041 | G | A | synonymous | SPTBN5 | SPTBN5 |  | N |
| HCC-B3 | somaticSNP | 15 | 42458777 | C | A | missense | VPS39 | VPS39 |  | Y |
| HCC-B3 | somaticSNP | 15 | 63908503 | C | A | intron | HERC1 |  |  | Y |
| HCC-B3 | somaticSNP | 15 | 64791958 | G | A | missense | ZNF609 | ZNF609 |  | Y |
| HCC-B3 | somaticSNP | 15 | 79304864 | G | T | intron,missense | RASGRF1 | RASGRF1 |  | Y |
| HCC-B3 | somaticINDEL | 15 | 83241984 | A | -T | intron | CPEB1 |  |  | N |
| HCC-B3 | somaticSNP | 16 | 1259343 | C | T | synonymous | CACNA1H | CACNA1H |  | Y |
| HCC-B3 | somaticSNP | 16 | 2255888 | G | T | intron | MLST8 |  |  | N |
| HCC-B3 | somaticSNP | 16 | 2891338 | G | T | non-coding-exon | PRSS30P | PRSS30P |  | Y |
| HCC-B3 | somaticSNP | 16 | 7629905 | C | T | intron,missense | RBFOX1 | RBFOX1 |  | Y |
| HCC-B3 | somaticSNP | 16 | 11556121 | A | G | synonymous | LOC400499 |  |  | Y |
| HCC-B3 | somaticSNP | 16 | 69788684 | G | A | intron | NOB1 |  |  | Y |
| HCC-B3 | somaticSNP | 16 | 85120713 | A | C | missense | KIAA0513 | KIAA0513 |  | Y |
| HCC-B3 | somaticSNP | 17 | 6596268 | A | G | intron | SLC13A5 |  |  | N |
| HCC-B3 | somaticSNP | 17 | 10411706 | A | G | missense | MYH1 | MYH1 |  | N |
| HCC-B3 | somaticINDEL | 17 | 10550845 | T | -TTTTG | intron | MYH3 |  |  | N |
| HCC-B3 | somaticSNP | 17 | 37824920 | C | T | intron,synonymous | PNMT | PNMT |  | Y |
| HCC-B3 | somaticSNP | 17 | 38078771 | T | C | 3-prime-UTR | ORMDL3 | ORMDL3 |  | Y |
| HCC-B3 | somaticSNP | 17 | 38634492 | G | A | intron | TNS4 |  |  | N |
| HCC-B3 | somaticSNP | 17 | 39534271 | A | T | 3-prime-UTR | KRT34 | KRT34 |  | Y |
| HCC-B3 | somaticSNP | 17 | 40937100 | C | T | intron | WNK4 |  |  | Y |
| HCC-B3 | somaticINDEL | 17 | 56693799 | C | +T | intron | TEX14 |  |  | N |
| HCC-B3 | somaticSNP | 17 | 62777779 | C | T | non-coding-exon | LOC146880 | LOC146880 |  | Y |
| HCC-B3 | somaticSNP | 17 | 67170973 | G | T | intron | ABCA10 |  |  | N |
| HCC-B3 | somaticSNP | 17 | 72578342 | C | A | intron | CD300LD |  |  | N |
| HCC-B3 | somaticSNP | 17 | 74079838 | A | G | intron-near-splice | EXOC7 |  |  | N |
| HCC-B3 | somaticSNP | 17 | 74740318 | G | T | intron | MFSD11 |  |  | Y |
| HCC-B3 | somaticSNP | 17 | 76989903 | T | C | missense | CANT1 | CANT1 | CANT1 | Y |
| HCC-B3 | somaticSNP | 18 | 2567069 | T | C | intron,synonymous | METTL4 | METTL4 |  | N |
| HCC-B3 | somaticSNP | 18 | 63489374 | T | A | missense | CDH7 | CDH7 |  | Y |
| HCC-B3 | somaticSNP | 19 | 5746056 | T | C | synonymous | CATSPERD | CATSPERD |  | Y |
| HCC-B3 | somaticSNP | 19 | 7808968 | A | G | intron | CD209 |  |  | Y |
| HCC-B3 | somaticSNP | 19 | 9000446 | C | T | missense | MUC16 | MUC16 |  | Y |
| HCC-B3 | somaticSNP | 19 | 9586051 | A | G | intron-near-splice | ZNF560 |  |  | Y |
| HCC-B3 | somaticSNP | 19 | 10741732 | G | A | splice-acceptor | SLC44A2 | SLC44A2 |  | Y |
| HCC-B3 | somaticSNP | 19 | 16496069 | C | A | intron | EPS15L1 |  |  | Y |
| HCC-B3 | somaticSNP | 19 | 18248192 | A | G | intron | MAST3 |  |  | Y |
| HCC-B3 | somaticSNP | 19 | 38958392 | G | A | synonymous | RYR1 | RYR1 |  | N |
| HCC-B3 | somaticSNP | 19 | 40433693 | T | C | synonymous | FCGBP | FCGBP |  | Y |
| HCC-B3 | somaticSNP | 19 | 45147340 | G | T | 5-prime-UTR | PVR | PVR |  | Y |
| HCC-B3 | somaticSNP | 19 | 47259006 | G | A | 5-prime-UTR,missense | FKRP | FKRP |  | Y |
| HCC-B3 | somaticSNP | 19 | 50463727 | G | A | intron | SIGLEC11 |  |  | N |
| HCC-B3 | somaticSNP | 19 | 51919279 | A | G | intron,synonymous,missense | SIGLEC10,LOC100129083 | SIGLEC10,LOC100129083 |  | N |
| HCC-B3 | somaticSNP | 19 | 51919302 | T | C | intron,synonymous,missense | SIGLEC10,LOC100129083 | SIGLEC10,LOC100129083 |  | N |
| HCC-B3 | somaticSNP | 2 | 21361803 | A | G | intergenic |  | |  | Y |
| HCC-B3 | somaticSNP | 2 | 31412096 | T | C | intron | CAPN14 |  |  | Y |
| HCC-B3 | somaticSNP | 2 | 42511974 | G | A | intron | EML4 |  | EML4 | Y |
| HCC-B3 | somaticSNP | 2 | 61697887 | C | G | upstream-gene |  | |  | Y |
| HCC-B3 | somaticSNP | 2 | 72960159 | T | C | intron | EXOC6B |  |  | Y |
| HCC-B3 | somaticSNP | 2 | 133014556 | C | T | intron,non-coding-exon | MIR663B,ANKRD30BL | MIR663B |  | N |
| HCC-B3 | somaticINDEL | 2 | 170677918 | C | +T | intron | METTL5 |  |  | Y |
| HCC-B3 | somaticSNP | 2 | 175436749 | C | A | missense | WIPF1 | WIPF1 |  | N |
| HCC-B3 | somaticSNP | 2 | 178096766 | A | C | intron,intron-near-splice | NFE2L2 |  | NFE2L2 | N |
| HCC-B3 | somaticSNP | 2 | 186667287 | T | A | synonymous | FSIP2 | FSIP2 |  | Y |
| HCC-B3 | somaticSNP | 2 | 242800900 | G | A | intron | PDCD1 |  |  | N |
| HCC-B3 | somaticSNP | 20 | 3219894 | A | G | intron | SLC4A11 |  |  | N |
| HCC-B3 | somaticSNP | 20 | 9346228 | G | A | intron | PLCB4 |  |  | Y |
| HCC-B3 | somaticSNP | 20 | 9449200 | C | T | intron | PLCB4 |  |  | Y |
| HCC-B3 | somaticSNP | 20 | 40125973 | T | C | intron | CHD6 |  |  | Y |
| HCC-B3 | somaticSNP | 20 | 44486101 | G | A | upstream-gene |  | |  | Y |
| HCC-B3 | somaticSNP | 21 | 19167551 | T | C | intron,missense | C21orf91 | C21orf91 |  | Y |
| HCC-B3 | somaticINDEL | 21 | 35186127 | C | -T | intron | ITSN1 |  |  | Y |
| HCC-B3 | somaticSNP | 22 | 23041026 | G | A | intergenic |  | |  | N |
| HCC-B3 | somaticSNP | 22 | 30050611 | A | G | intron | NF2 |  | NF2 | Y |
| HCC-B3 | somaticSNP | 3 | 443305 | T | G | intron-near-splice | CHL1 |  |  | Y |
| HCC-B3 | somaticSNP | 3 | 19322831 | G | A | intron | KCNH8 |  |  | N |
| HCC-B3 | somaticSNP | 3 | 47807173 | G | T | intron | SMARCC1 |  |  | Y |
| HCC-B3 | somaticSNP | 3 | 52584712 | A | G | intron | PBRM1 |  | PBRM1 | Y |
| HCC-B3 | somaticSNP | 3 | 69112049 | C | T | intron | UBA3 |  |  | N |
| HCC-B3 | somaticSNP | 3 | 121132268 | T | A | intron | STXBP5L |  |  | N |
| HCC-B3 | somaticSNP | 3 | 124807132 | C | T | intron | SLC12A8 |  |  | Y |
| HCC-B3 | somaticSNP | 3 | 142211929 | C | T | intron | ATR |  |  | Y |
| HCC-B3 | somaticINDEL | 3 | 171065010 | G | +A | intron | TNIK |  |  | N |
| HCC-B3 | somaticSNP | 4 | 1244834 | G | A | non-coding-exon | CTBP1-AS2 | CTBP1-AS2 |  | Y |
| HCC-B3 | somaticSNP | 4 | 1818471 | G | A | synonymous | LETM1 | LETM1 |  | N |
| HCC-B3 | somaticSNP | 4 | 10020564 | G | A | intron | SLC2A9 |  |  | N |
| HCC-B3 | somaticINDEL | 4 | 26241366 | G | +GGAGA | intron | RBPJ |  |  | N |
| HCC-B3 | somaticSNP | 4 | 103556316 | T | C | intron | MANBA |  |  | Y |
| HCC-B3 | somaticINDEL | 4 | 106317370 | T | +ATAAA | intron | PPA2 |  |  | N |
| HCC-B3 | somaticSNP | 5 | 7832063 | C | T | missense | C5orf49 | C5orf49 |  | Y |
| HCC-B3 | somaticSNP | 5 | 10382131 | A | G | intron | MARCH6 |  |  | Y |
| HCC-B3 | somaticSNP | 5 | 33649003 | G | A | missense | ADAMTS12 | ADAMTS12 |  | Y |
| HCC-B3 | somaticINDEL | 5 | 78416973 | C | -A | intron | BHMT |  |  | Y |
| HCC-B3 | somaticSNP | 5 | 118480380 | T | A | intron | DMXL1 |  |  | Y |
| HCC-B3 | somaticSNP | 5 | 135396468 | T | A | intron | TGFBI |  |  | N |
| HCC-B3 | somaticSNP | 5 | 141005368 | G | A | intron | HDAC3 |  |  | Y |
| HCC-B3 | somaticSNP | 5 | 149112300 | T | G | intron | PPARGC1B |  |  | Y |
| HCC-B3 | somaticSNP | 6 | 5000779 | G | A | intron | RPP40 |  |  | N |
| HCC-B3 | somaticSNP | 6 | 13228075 | T | G | synonymous | PHACTR1 | PHACTR1 |  | N |
| HCC-B3 | somaticSNP | 6 | 28197605 | C | T | intron | ZSCAN9 |  |  | Y |
| HCC-B3 | somaticSNP | 6 | 54212153 | T | C | intron | TINAG |  |  | N |
| HCC-B3 | somaticSNP | 6 | 73001594 | T | A | intron | RIMS1 |  |  | N |
| HCC-B3 | somaticSNP | 6 | 75860786 | A | C | intron | COL12A1 |  |  | Y |
| HCC-B3 | somaticSNP | 6 | 110048404 | G | T | stop-gained,intron | FIG4 | FIG4 |  | Y |
| HCC-B3 | somaticSNP | 6 | 130425541 | T | G | intron | L3MBTL3 |  |  | Y |
| HCC-B3 | somaticSNP | 6 | 139100991 | G | C | missense | CCDC28A | CCDC28A |  | Y |
| HCC-B3 | somaticSNP | 7 | 1914350 | A | G | intron | MAD1L1 |  |  | Y |
| HCC-B3 | somaticINDEL | 7 | 22330862 | T | +A | intron | RAPGEF5 |  |  | N |
| HCC-B3 | somaticSNP | 7 | 38295939 | A | T | downstream-gene |  | |  | Y |
| HCC-B3 | somaticSNP | 7 | 94248183 | A | G | synonymous | SGCE | SGCE |  | Y |
| HCC-B3 | somaticSNP | 7 | 99578370 | T | G | upstream-gene |  | |  | Y |
| HCC-B3 | somaticSNP | 7 | 99578381 | T | C | upstream-gene |  | |  | Y |
| HCC-B3 | somaticSNP | 7 | 99578411 | T | C | non-coding-exon | AZGP1P1 | AZGP1P1 |  | Y |
| HCC-B3 | somaticSNP | 7 | 103053467 | A | G | missense | SLC26A5 | SLC26A5 |  | N |
| HCC-B3 | somaticSNP | 7 | 113519608 | A | T | missense | PPP1R3A | PPP1R3A |  | N |
| HCC-B3 | somaticSNP | 8 | 6500615 | C | T | 3-prime-UTR | MCPH1 | MCPH1 |  | N |
| HCC-B3 | somaticSNP | 8 | 8234211 | C | T | missense | SGK223 | SGK223 |  | Y |
| HCC-B3 | somaticSNP | 8 | 52336073 | T | G | intron | PXDNL |  |  | Y |
| HCC-B3 | somaticSNP | 8 | 52384961 | T | A | intron | PXDNL |  |  | N |
| HCC-B3 | somaticSNP | 8 | 95894920 | T | C | intron | CCNE2 |  |  | Y |
| HCC-B3 | somaticSNP | 8 | 116426272 | T | C | synonymous | TRPS1 | TRPS1 |  | Y |
| HCC-B3 | somaticSNP | 8 | 133769672 | A | T | intron | TMEM71 |  |  | Y |
| HCC-B3 | somaticSNP | 9 | 2651521 | C | A | intron | VLDLR |  |  | N |
| HCC-B3 | somaticSNP | 9 | 19087295 | T | C | intron | HAUS6 |  |  | Y |
| HCC-B3 | somaticSNP | 9 | 21409818 | C | G | 3-prime-UTR | IFNA8 | IFNA8 |  | Y |
| HCC-B3 | somaticSNP | 9 | 88207477 | G | T | missense | AGTPBP1 | AGTPBP1 |  | Y |
| HCC-B3 | somaticSNP | 9 | 96285612 | C | T | intron,missense | FAM120A |  |  | Y |
| HCC-B3 | somaticSNP | 9 | 125909298 | G | A | synonymous,non-coding-exon | STRBP | STRBP |  | N |
| HCC-B3 | somaticSNP | X | 48855744 | A | C | intron | GRIPAP1 |  |  | Y |
| HCC-B3 | somaticSNP | X | 69669167 | C | T | intron | DLG3 |  |  | Y |
| HCC-B3 | somaticSNP | X | 101858685 | T | G | stop-gained,intron | ARMCX5,ARMCX5-GPRASP2 | ARMCX5 |  | Y |
| HCC-B3 | somaticSNP | X | 140996400 | G | C | synonymous | MAGEC1 | MAGEC1 |  | Y |
| HCC-B3 | somaticSNP | 18 | 10857072 | C | A | missense | PIEZO2 | PIEZO2 |  | Y |
| HCC-B3 | somaticSNP | 20 | 14306910 | C | G | intron,missense | FLRT3,MACROD2 | FLRT3 |  | Y |
| HCC-B3 | somaticSNP | 7 | 113520039 | A | G | missense | PPP1R3A | PPP1R3A |  | N |
| HCC-B3 | somaticSNP | 1 | 182443101 | A | T | missense | RGSL1 | RGSL1 |  | Y |
| HCC-B3 | somaticSNP | 13 | 43788226 | A | G | missense | ENOX1 | ENOX1 |  | N |
| HCC-B3 | somaticSNP | 13 | 114309228 | A | T | missense | ATP4B | ATP4B |  | N |
| HCC-B3 | somaticSNP | 14 | 56079145 | G | T | stop-gained,non-coding-exon | KTN1 | KTN1 | KTN1 | N |
| HCC-B3 | somaticSNP | 19 | 2247967 | G | T | missense | SF3A2 | SF3A2 |  | Y |
| HCC-B3 | somaticSNP | 19 | 10476488 | C | A | missense | TYK2 | TYK2 |  | N |
| HCC-B3 | somaticSNP | 19 | 54485450 | G | A | missense | CACNG8 | CACNG8 |  | N |
| HCC-B3 | somaticSNP | 4 | 126412781 | A | C | missense | FAT4 | FAT4 |  | Y |
| HCC-B3 | somaticSNP | 5 | 128440932 | A | G | missense | ISOC1 | ISOC1 |  | Y |
| HCC-B3 | somaticSNP | 7 | 30830809 | G | A | missense,non-coding-exon | INMT-FAM188B,FAM188B | INMT-FAM188B,FAM188B |  | N |
| HCC-B3 | somaticSNP | 7 | 82785265 | T | A | missense | PCLO | PCLO |  | N |
| HCC-B3 | somaticSNP | 9 | 1056448 | C | A | stop-gained,3-prime-UTR | DMRT2 | DMRT2 |  | N |
| ICC-C1 | somaticSNP | 7 | 151970859 | C | T | missense | KMT2C | KMT2C | KMT2C | Y |
| ICC-C1 | somaticSNP | 1 | 16458553 | A | C | intron-near-splice | EPHA2 |  |  | Y |
| ICC-C1 | somaticSNP | 1 | 17023483 | C | T | intron | ESPNP |  |  | Y |
| ICC-C1 | somaticSNP | 1 | 17267496 | C | T | intron | CROCC |  |  | N |
| ICC-C1 | somaticSNP | 1 | 26873700 | C | T | 5-prime-UTR,synonymous | RPS6KA1 | RPS6KA1 |  | N |
| ICC-C1 | somaticSNP | 1 | 29611123 | C | T | intron | PTPRU |  |  | Y |
| ICC-C1 | somaticSNP | 1 | 41327871 | C | A | 5-prime-UTR | CITED4 | CITED4 |  | Y |
| ICC-C1 | somaticSNP | 1 | 76771484 | A | T | intron | ST6GALNAC3 |  |  | Y |
| ICC-C1 | somaticSNP | 1 | 86249967 | C | T | missense | COL24A1 | COL24A1 |  | Y |
| ICC-C1 | somaticINDEL | 1 | 93159821 | C | -A | intron | EVI5 |  |  | N |
| ICC-C1 | somaticINDEL | 1 | 103471734 | A | +AAATAAATAAAT | intron | COL11A1 |  |  | Y |
| ICC-C1 | somaticSNP | 1 | 205034434 | G | T | intron | CNTN2 |  |  | Y |
| ICC-C1 | somaticSNP | 1 | 227227740 | A | C | intron | CDC42BPA |  |  | N |
| ICC-C1 | somaticSNP | 1 | 247902511 | G | A | intron | TRIM58 |  |  | Y |
| ICC-C1 | somaticINDEL | 10 | 60160134 | T | -AAA | intergenic |  | |  | N |
| ICC-C1 | somaticSNP | 10 | 82331347 | A | G | intron | SH2D4B |  |  | N |
| ICC-C1 | somaticINDEL | 10 | 93776240 | C | +T | intron | BTAF1 |  |  | Y |
| ICC-C1 | somaticSNP | 10 | 94822347 | A | G | intron | CYP26C1 |  |  | Y |
| ICC-C1 | somaticINDEL | 10 | 95454768 | T | -A | intron | FRA10AC1 |  |  | N |
| ICC-C1 | somaticSNP | 11 | 30353024 | A | G | 5-prime-UTR,intron | ARL14EP |  |  | Y |
| ICC-C1 | somaticSNP | 11 | 73536698 | A | C | intron | MRPL48 |  |  | N |
| ICC-C1 | somaticSNP | 11 | 101375549 | T | C | intron | TRPC6 |  |  | N |
| ICC-C1 | somaticSNP | 12 | 11286184 | G | A | synonymous | PRH1-PRR4,TAS2R30,PRH1 | TAS2R30 |  | N |
| ICC-C1 | somaticSNP | 12 | 21695584 | A | G | intron | GYS2 |  |  | N |
| ICC-C1 | somaticINDEL | 12 | 97311376 | C | -T | intron | NEDD1 |  |  | Y |
| ICC-C1 | somaticSNP | 13 | 99540395 | A | G | intron | DOCK9 |  |  | N |
| ICC-C1 | somaticSNP | 14 | 23733725 | C | T | intron | C14orf164 |  |  | N |
| ICC-C1 | somaticSNP | 14 | 104199580 | A | C | 3-prime-UTR | ZFYVE21 | ZFYVE21 |  | N |
| ICC-C1 | somaticINDEL | 15 | 83241984 | A | -T | intron | CPEB1 |  |  | N |
| ICC-C1 | somaticSNP | 16 | 11556121 | A | G | synonymous | LOC400499 |  |  | Y |
| ICC-C1 | somaticSNP | 16 | 23695237 | C | T | missense | PLK1 | PLK1 |  | Y |
| ICC-C1 | somaticINDEL | 17 | 10550845 | T | -TTTTG | intron | MYH3 |  |  | N |
| ICC-C1 | somaticINDEL | 17 | 56693799 | C | +T | intron | TEX14 |  |  | N |
| ICC-C1 | somaticSNP | 17 | 74079838 | A | G | intron-near-splice | EXOC7 |  |  | N |
| ICC-C1 | somaticSNP | 19 | 15276007 | C | A | intron | NOTCH3 |  |  | N |
| ICC-C1 | somaticSNP | 19 | 45147340 | G | T | 5-prime-UTR | PVR | PVR |  | N |
| ICC-C1 | somaticSNP | 19 | 50463727 | G | A | intron | SIGLEC11 |  |  | Y |
| ICC-C1 | somaticSNP | 2 | 31412096 | T | C | intron | CAPN14 |  |  | N |
| ICC-C1 | somaticSNP | 2 | 62067147 | T | C | missense,non-coding-exon | FAM161A | FAM161A |  | Y |
| ICC-C1 | somaticSNP | 2 | 89197249 | A | T | intergenic |  | |  | Y |
| ICC-C1 | somaticINDEL | 2 | 201778717 | T | +A | intron | ORC2 |  |  | Y |
| ICC-C1 | somaticSNP | 2 | 203424500 | G | A | missense | BMPR2 | BMPR2 |  | Y |
| ICC-C1 | somaticSNP | 2 | 242800900 | G | A | intron | PDCD1 |  |  | Y |
| ICC-C1 | somaticSNP | 20 | 3219894 | A | G | intron | SLC4A11 |  |  | Y |
| ICC-C1 | somaticSNP | 20 | 9449200 | C | T | intron | PLCB4 |  |  | N |
| ICC-C1 | somaticSNP | 20 | 47731276 | T | C | 3-prime-UTR | STAU1 | STAU1 |  | Y |
| ICC-C1 | somaticINDEL | 21 | 19628809 | C | -T | intron | CHODL |  |  | Y |
| ICC-C1 | somaticINDEL | 21 | 35186127 | C | -T | intron | ITSN1 |  |  | Y |
| ICC-C1 | somaticSNP | 21 | 45757631 | G | A | intron | C21orf2 |  |  | Y |
| ICC-C1 | somaticSNP | 22 | 23041026 | G | A | intergenic |  | |  | N |
| ICC-C1 | somaticSNP | 3 | 69112049 | C | T | intron | UBA3 |  |  | N |
| ICC-C1 | somaticSNP | 3 | 121132268 | T | A | intron | STXBP5L |  |  | N |
| ICC-C1 | somaticINDEL | 3 | 150282061 | C | -T | intron | EIF2A |  |  | N |
| ICC-C1 | somaticINDEL | 4 | 26241366 | G | +GGAGA | intron | RBPJ |  |  | N |
| ICC-C1 | somaticSNP | 4 | 103556316 | T | C | intron | MANBA |  |  | N |
| ICC-C1 | somaticINDEL | 4 | 106317370 | T | +ATAAA | intron | PPA2 |  |  | N |
| ICC-C1 | somaticSNP | 5 | 102489511 | T | G | intron | PPIP5K2 |  |  | Y |
| ICC-C1 | somaticSNP | 5 | 135396468 | T | A | intron | TGFBI |  |  | N |
| ICC-C1 | somaticSNP | 5 | 149112300 | T | G | intron | PPARGC1B |  |  | Y |
| ICC-C1 | somaticSNP | 6 | 5000779 | G | A | intron | RPP40 |  |  | Y |
| ICC-C1 | somaticINDEL | 6 | 24651352 | T | -A | intron | TDP2 |  |  | Y |
| ICC-C1 | somaticSNP | 6 | 28197605 | C | T | intron | ZSCAN9 |  |  | Y |
| ICC-C1 | somaticSNP | 7 | 1914350 | A | G | intron | MAD1L1 |  |  | Y |
| ICC-C1 | somaticINDEL | 7 | 22330862 | T | +A | intron | RAPGEF5 |  |  | Y |
| ICC-C1 | somaticSNP | 7 | 99578370 | T | G | upstream-gene |  | |  | Y |
| ICC-C1 | somaticSNP | 7 | 99578381 | T | C | upstream-gene |  | |  | Y |
| ICC-C1 | somaticSNP | 7 | 99578411 | T | C | non-coding-exon | AZGP1P1 | AZGP1P1 |  | Y |
| ICC-C1 | somaticSNP | 7 | 100696345 | C | T | synonymous | MUC17 | MUC17 |  | Y |
| ICC-C1 | somaticSNP | 9 | 136509373 | T | G | missense | DBH | DBH |  | Y |
| ICC-C1 | somaticSNP | 9 | 137968821 | G | A | intron | OLFM1 |  |  | Y |
| ICC-C1 | somaticSNP | X | 129535976 | A | G | 5-prime-UTR | RBMX2 | RBMX2 |  | Y |
| ICC-C1 | somaticSNP | X | 135496361 | A | T | missense | GPR112 | GPR112 |  | Y |
| ICC-C1 | somaticSNP | X | 135579972 | G | T | missense | HTATSF1 | HTATSF1 |  | Y |
| ICC-C1 | somaticSNP | 1 | 115258747 | C | T | missense | NRAS | NRAS | NRAS | Y |
| ICC-C2 | somaticSNP | 1 | 86249967 | C | T | missense | COL24A1 | COL24A1 |  | N |
| ICC-C2 | somaticSNP | 1 | 16458553 | A | C | intron-near-splice | EPHA2 |  |  | N |
| ICC-C2 | somaticSNP | 1 | 17023483 | C | T | intron | ESPNP |  |  | N |
| ICC-C2 | somaticINDEL | 1 | 24294450 | C | +A | intron | SRSF10 |  |  | N |
| ICC-C2 | somaticINDEL | 1 | 25780889 | A | +TG | intron | TMEM57 |  |  | Y |
| ICC-C2 | somaticSNP | 1 | 26873700 | C | T | 5-prime-UTR,synonymous | RPS6KA1 | RPS6KA1 |  | N |
| ICC-C2 | somaticSNP | 1 | 29611123 | C | T | intron | PTPRU |  |  | N |
| ICC-C2 | somaticSNP | 1 | 41327871 | C | A | 5-prime-UTR | CITED4 | CITED4 |  | Y |
| ICC-C2 | somaticINDEL | 1 | 62350123 | G | -TTTTT | intron | INADL |  |  | Y |
| ICC-C2 | somaticINDEL | 1 | 93159821 | C | -A | intron | EVI5 |  |  | N |
| ICC-C2 | somaticSNP | 1 | 205034434 | G | T | intron | CNTN2 |  |  | Y |
| ICC-C2 | somaticSNP | 1 | 227227740 | A | C | intron | CDC42BPA |  |  | N |
| ICC-C2 | somaticSNP | 10 | 82331347 | A | G | intron | SH2D4B |  |  | N |
| ICC-C2 | somaticINDEL | 10 | 95454768 | T | -A | intron | FRA10AC1 |  |  | N |
| ICC-C2 | somaticSNP | 11 | 73536698 | A | C | intron | MRPL48 |  |  | N |
| ICC-C2 | somaticSNP | 12 | 21695584 | A | G | intron | GYS2 |  |  | N |
| ICC-C2 | somaticSNP | 12 | 53238112 | C | T | intron | KRT78 |  |  | N |
| ICC-C2 | somaticINDEL | 12 | 97311376 | C | -T | intron | NEDD1 |  |  | N |
| ICC-C2 | somaticSNP | 13 | 20277413 | T | C | missense | PSPC1 | PSPC1 |  | N |
| ICC-C2 | somaticSNP | 13 | 99540395 | A | G | intron | DOCK9 |  |  | N |
| ICC-C2 | somaticSNP | 14 | 23733725 | C | T | intron | C14orf164 |  |  | Y |
| ICC-C2 | somaticSNP | 14 | 104199580 | A | C | 3-prime-UTR | ZFYVE21 | ZFYVE21 |  | Y |
| ICC-C2 | somaticINDEL | 15 | 41308142 | C | -A | intron | INO80 |  |  | Y |
| ICC-C2 | somaticINDEL | 15 | 83241984 | A | -T | intron | CPEB1 |  |  | N |
| ICC-C2 | somaticSNP | 16 | 11556121 | A | G | synonymous | LOC400499 |  |  | Y |
| ICC-C2 | somaticSNP | 16 | 23695237 | C | T | missense | PLK1 | PLK1 |  | N |
| ICC-C2 | somaticINDEL | 17 | 56693799 | C | +T | intron | TEX14 |  |  | Y |
| ICC-C2 | somaticSNP | 17 | 74079838 | A | G | intron-near-splice | EXOC7 |  |  | Y |
| ICC-C2 | somaticSNP | 19 | 15276007 | C | A | intron | NOTCH3 |  |  | N |
| ICC-C2 | somaticSNP | 19 | 45147340 | G | T | 5-prime-UTR | PVR | PVR |  | N |
| ICC-C2 | somaticSNP | 19 | 50463727 | G | A | intron | SIGLEC11 |  |  | Y |
| ICC-C2 | somaticSNP | 19 | 51919279 | A | G | intron,synonymous,missense | SIGLEC10,LOC100129083 | SIGLEC10,LOC100129083 |  | Y |
| ICC-C2 | somaticSNP | 19 | 51919302 | T | C | intron,synonymous,missense | SIGLEC10,LOC100129083 | SIGLEC10,LOC100129083 |  | Y |
| ICC-C2 | somaticSNP | 2 | 31412096 | T | C | intron | CAPN14 |  |  | Y |
| ICC-C2 | somaticSNP | 2 | 61697887 | C | G | upstream-gene |  | |  | N |
| ICC-C2 | somaticSNP | 2 | 62067147 | T | C | missense,non-coding-exon | FAM161A | FAM161A |  | N |
| ICC-C2 | somaticSNP | 2 | 89197249 | A | T | intergenic |  | |  | N |
| ICC-C2 | somaticINDEL | 2 | 170677918 | C | +T | intron | METTL5 |  |  | N |
| ICC-C2 | somaticINDEL | 2 | 201778717 | T | +A | intron | ORC2 |  |  | Y |
| ICC-C2 | somaticSNP | 2 | 242800900 | G | A | intron | PDCD1 |  |  | Y |
| ICC-C2 | somaticSNP | 20 | 3219894 | A | G | intron | SLC4A11 |  |  | Y |
| ICC-C2 | somaticSNP | 20 | 9449200 | C | T | intron | PLCB4 |  |  | N |
| ICC-C2 | somaticINDEL | 21 | 19628809 | C | -T | intron | CHODL |  |  | N |
| ICC-C2 | somaticINDEL | 21 | 35186127 | C | -T | intron | ITSN1 |  |  | N |
| ICC-C2 | somaticSNP | 22 | 23041026 | G | A | intergenic |  | |  | Y |
| ICC-C2 | somaticSNP | 3 | 69112049 | C | T | intron | UBA3 |  |  | Y |
| ICC-C2 | somaticSNP | 3 | 121132268 | T | A | intron | STXBP5L |  |  | N |
| ICC-C2 | somaticSNP | 4 | 10020564 | G | A | intron | SLC2A9 |  |  | Y |
| ICC-C2 | somaticINDEL | 4 | 26241366 | G | +GGAGA | intron | RBPJ |  |  | N |
| ICC-C2 | somaticSNP | 4 | 103556316 | T | C | intron | MANBA |  |  | Y |
| ICC-C2 | somaticINDEL | 4 | 106317370 | T | +ATAAA | intron | PPA2 |  |  | N |
| ICC-C2 | somaticINDEL | 5 | 78416973 | C | -A | intron | BHMT |  |  | N |
| ICC-C2 | somaticSNP | 5 | 135396468 | T | A | intron | TGFBI |  |  | N |
| ICC-C2 | somaticSNP | 5 | 149112300 | T | G | intron | PPARGC1B |  |  | Y |
| ICC-C2 | somaticSNP | 6 | 5000779 | G | A | intron | RPP40 |  |  | Y |
| ICC-C2 | somaticSNP | 6 | 28197605 | C | T | intron | ZSCAN9 |  |  | Y |
| ICC-C2 | somaticSNP | 7 | 1914350 | A | G | intron | MAD1L1 |  |  | Y |
| ICC-C2 | somaticINDEL | 7 | 22330862 | T | +A | intron | RAPGEF5 |  |  | Y |
| ICC-C2 | somaticSNP | 7 | 99578370 | T | G | upstream-gene |  | |  | N |
| ICC-C2 | somaticSNP | 7 | 99578381 | T | C | upstream-gene |  | |  | Y |
| ICC-C2 | somaticSNP | 7 | 99578411 | T | C | non-coding-exon | AZGP1P1 | AZGP1P1 |  | Y |
| ICC-C2 | somaticINDEL | 7 | 116409675 | C | -T | intron | MET |  | MET | N |
| ICC-C2 | somaticSNP | X | 129535976 | A | G | 5-prime-UTR | RBMX2 | RBMX2 |  | N |
| ICC-C2 | somaticSNP | X | 135579972 | G | T | missense | HTATSF1 | HTATSF1 |  | N |
| ICC-C3 | somaticSNP | 1 | 16458553 | A | C | intron-near-splice | EPHA2 |  |  | N |
| ICC-C3 | somaticSNP | 1 | 17023483 | C | T | intron | ESPNP |  |  | N |
| ICC-C3 | somaticSNP | 1 | 17267496 | C | T | intron | CROCC |  |  | N |
| ICC-C3 | somaticINDEL | 1 | 24294450 | C | +A | intron | SRSF10 |  |  | Y |
| ICC-C3 | somaticSNP | 1 | 26873700 | C | T | 5-prime-UTR,synonymous | RPS6KA1 | RPS6KA1 |  | N |
| ICC-C3 | somaticSNP | 1 | 29611123 | C | T | intron | PTPRU |  |  | N |
| ICC-C3 | somaticSNP | 1 | 41327871 | C | A | 5-prime-UTR | CITED4 | CITED4 |  | N |
| ICC-C3 | somaticSNP | 1 | 86249967 | C | T | missense | COL24A1 | COL24A1 |  | Y |
| ICC-C3 | somaticINDEL | 1 | 103471734 | A | +AAATAAATAAAT | intron | COL11A1 |  |  | N |
| ICC-C3 | somaticSNP | 1 | 145281824 | T | G | intron | NBPF20,NBPF9,NOTCH2NL |  |  | N |
| ICC-C3 | somaticSNP | 1 | 205034434 | G | T | intron | CNTN2 |  |  | Y |
| ICC-C3 | somaticSNP | 1 | 227227740 | A | C | intron | CDC42BPA |  |  | Y |
| ICC-C3 | somaticSNP | 10 | 29581055 | T | C | intron | LYZL1 |  |  | N |
| ICC-C3 | somaticINDEL | 10 | 60160134 | T | -AAA | intergenic |  | |  | Y |
| ICC-C3 | somaticSNP | 10 | 82331347 | A | G | intron | SH2D4B |  |  | Y |
| ICC-C3 | somaticINDEL | 10 | 93776240 | C | +T | intron | BTAF1 |  |  | N |
| ICC-C3 | somaticINDEL | 10 | 95454768 | T | -A | intron | FRA10AC1 |  |  | Y |
| ICC-C3 | somaticSNP | 11 | 73536698 | A | C | intron | MRPL48 |  |  | N |
| ICC-C3 | somaticSNP | 11 | 101375549 | T | C | intron | TRPC6 |  |  | N |
| ICC-C3 | somaticSNP | 12 | 21695584 | A | G | intron | GYS2 |  |  | Y |
| ICC-C3 | somaticSNP | 12 | 31255227 | A | G | synonymous | DDX11 | DDX11 |  | Y |
| ICC-C3 | somaticINDEL | 12 | 97311376 | C | -T | intron | NEDD1 |  |  | N |
| ICC-C3 | somaticSNP | 13 | 20277413 | T | C | missense | PSPC1 | PSPC1 |  | N |
| ICC-C3 | somaticSNP | 13 | 99540395 | A | G | intron | DOCK9 |  |  | N |
| ICC-C3 | somaticSNP | 14 | 23733725 | C | T | intron | C14orf164 |  |  | Y |
| ICC-C3 | somaticSNP | 14 | 104199580 | A | C | 3-prime-UTR | ZFYVE21 | ZFYVE21 |  | Y |
| ICC-C3 | somaticSNP | 16 | 11556121 | A | G | synonymous | LOC400499 |  |  | N |
| ICC-C3 | somaticINDEL | 17 | 10550845 | T | -TTTTG | intron | MYH3 |  |  | N |
| ICC-C3 | somaticSNP | 17 | 21207939 | G | T | intron | MAP2K3 |  |  | Y |
| ICC-C3 | somaticSNP | 17 | 74079838 | A | G | intron-near-splice | EXOC7 |  |  | N |
| ICC-C3 | somaticSNP | 19 | 15276007 | C | A | intron | NOTCH3 |  |  | N |
| ICC-C3 | somaticSNP | 2 | 31412096 | T | C | intron | CAPN14 |  |  | N |
| ICC-C3 | somaticSNP | 2 | 62067147 | T | C | missense,non-coding-exon | FAM161A | FAM161A |  | N |
| ICC-C3 | somaticSNP | 2 | 89197249 | A | T | intergenic |  | |  | Y |
| ICC-C3 | somaticSNP | 2 | 133014556 | C | T | intron,non-coding-exon | MIR663B,ANKRD30BL | MIR663B |  | Y |
| ICC-C3 | somaticINDEL | 2 | 170677918 | C | +T | intron | METTL5 |  |  | Y |
| ICC-C3 | somaticINDEL | 2 | 201778717 | T | +A | intron | ORC2 |  |  | Y |
| ICC-C3 | somaticSNP | 2 | 242800900 | G | A | intron | PDCD1 |  |  | Y |
| ICC-C3 | somaticSNP | 20 | 3219894 | A | G | intron | SLC4A11 |  |  | N |
| ICC-C3 | somaticSNP | 20 | 9449200 | C | T | intron | PLCB4 |  |  | N |
| ICC-C3 | somaticINDEL | 21 | 35186127 | C | -T | intron | ITSN1 |  |  | N |
| ICC-C3 | somaticSNP | 22 | 23041026 | G | A | intergenic |  | |  | Y |
| ICC-C3 | somaticSNP | 3 | 69112049 | C | T | intron | UBA3 |  |  | N |
| ICC-C3 | somaticSNP | 3 | 121132268 | T | A | intron | STXBP5L |  |  | Y |
| ICC-C3 | somaticINDEL | 3 | 171065010 | G | +A | intron | TNIK |  |  | N |
| ICC-C3 | somaticSNP | 4 | 10020564 | G | A | intron | SLC2A9 |  |  | N |
| ICC-C3 | somaticINDEL | 4 | 26241366 | G | +GGAGA | intron | RBPJ |  |  | N |
| ICC-C3 | somaticSNP | 4 | 103556316 | T | C | intron | MANBA |  |  | Y |
| ICC-C3 | somaticINDEL | 4 | 106317370 | T | +ATAAA | intron | PPA2 |  |  | N |
| ICC-C3 | somaticINDEL | 5 | 78416973 | C | -A | intron | BHMT |  |  | N |
| ICC-C3 | somaticSNP | 5 | 135396468 | T | A | intron | TGFBI |  |  | N |
| ICC-C3 | somaticSNP | 5 | 149112300 | T | G | intron | PPARGC1B |  |  | Y |
| ICC-C3 | somaticSNP | 6 | 5000779 | G | A | intron | RPP40 |  |  | N |
| ICC-C3 | somaticSNP | 6 | 28197605 | C | T | intron | ZSCAN9 |  |  | Y |
| ICC-C3 | somaticSNP | 7 | 1914350 | A | G | intron | MAD1L1 |  |  | Y |
| ICC-C3 | somaticINDEL | 7 | 22330862 | T | +A | intron | RAPGEF5 |  |  | N |
| ICC-C3 | somaticINDEL | 7 | 48412112 | T | -A | intron | ABCA13 |  |  | N |
| ICC-C3 | somaticSNP | 7 | 99578370 | T | G | upstream-gene |  | |  | N |
| ICC-C3 | somaticSNP | 7 | 99578381 | T | C | upstream-gene |  | |  | Y |
| ICC-C3 | somaticSNP | 7 | 99578411 | T | C | non-coding-exon | AZGP1P1 | AZGP1P1 |  | Y |
| ICC-C3 | somaticSNP | 9 | 136509373 | T | G | missense | DBH | DBH |  | N |
| ICC-C3 | somaticSNP | 9 | 137968821 | G | A | intron | OLFM1 |  |  | N |
| ICC-C3 | somaticSNP | X | 129535976 | A | G | 5-prime-UTR | RBMX2 | RBMX2 |  | N |
| ICC-C3 | somaticSNP | X | 135579972 | G | T | missense | HTATSF1 | HTATSF1 |  | Y |
| IM1 | somaticSNP | 1 | 15855461 | T | G | intron | DNAJC16 |  |  | Y |
| IM1 | somaticSNP | 1 | 17023483 | C | T | intron | ESPNP |  |  | Y |
| IM1 | somaticSNP | 1 | 17267496 | C | T | intron | CROCC |  |  | N |
| IM1 | somaticSNP | 1 | 19062153 | G | A | missense | PAX7 | PAX7 | PAX7 | Y |
| IM1 | somaticSNP | 1 | 21012616 | G | A | missense | KIF17 | KIF17 |  | Y |
| IM1 | somaticINDEL | 1 | 24294450 | C | +A | intron | SRSF10 |  |  | N |
| IM1 | somaticINDEL | 1 | 25780889 | A | +TG | intron | TMEM57 |  |  | N |
| IM1 | somaticSNP | 1 | 26738094 | T | G | intron | LIN28A |  |  | Y |
| IM1 | somaticSNP | 1 | 26873700 | C | T | 5-prime-UTR,synonymous | RPS6KA1 | RPS6KA1 |  | Y |
| IM1 | somaticSNP | 1 | 29611123 | C | T | intron | PTPRU |  |  | Y |
| IM1 | somaticSNP | 1 | 32150075 | T | G | intron | COL16A1 |  |  | Y |
| IM1 | somaticSNP | 1 | 34180246 | C | T | missense | CSMD2 | CSMD2 |  | Y |
| IM1 | somaticSNP | 1 | 36939412 | T | G | synonymous | CSF3R | CSF3R | CSF3R | Y |
| IM1 | somaticSNP | 1 | 53279594 | A | G | intron | ZYG11B |  |  | Y |
| IM1 | somaticINDEL | 1 | 62350123 | G | -TTTTT | intron | INADL |  |  | N |
| IM1 | somaticSNP | 1 | 62503632 | A | G | intron | INADL |  |  | Y |
| IM1 | somaticSNP | 1 | 67559220 | T | C | 3-prime-UTR,missense,non-coding-exon | C1orf141 | C1orf141 |  | Y |
| IM1 | somaticSNP | 1 | 78267110 | G | A | missense | FAM73A | FAM73A |  | Y |
| IM1 | somaticSNP | 1 | 103385782 | T | C | intron | COL11A1 |  |  | Y |
| IM1 | somaticSNP | 1 | 110129627 | G | T | intron | GNAI3 |  |  | N |
| IM1 | somaticSNP | 1 | 118165691 | C | T | synonymous | FAM46C | FAM46C | FAM46C | Y |
| IM1 | somaticSNP | 1 | 120306816 | T | C | missense | HMGCS2 | HMGCS2 |  | Y |
| IM1 | somaticSNP | 1 | 121116733 | A | G | intergenic | SRGAP2D,SRGAP2-AS1 | SRGAP2D |  | Y |
| IM1 | somaticSNP | 1 | 145281824 | T | G | intron | NBPF20,NBPF9,NOTCH2NL |  |  | Y |
| IM1 | somaticSNP | 1 | 153642826 | C | T | intron | ILF2 |  |  | Y |
| IM1 | somaticSNP | 1 | 156845864 | C | A | intron-near-splice,synonymous-near-splice | NTRK1 |  | NTRK1 | N |
| IM1 | somaticSNP | 1 | 157514235 | C | G | missense | FCRL5 | FCRL5 |  | Y |
| IM1 | somaticSNP | 1 | 158435706 | G | T | missense | OR10K1 | OR10K1 |  | Y |
| IM1 | somaticSNP | 1 | 158687102 | A | G | synonymous | OR6K3 | OR6K3 |  | N |
| IM1 | somaticSNP | 1 | 165180036 | T | C | intron | LMX1A |  |  | Y |
| IM1 | somaticSNP | 1 | 166959161 | T | C | intron | MAEL |  |  | Y |
| IM1 | somaticSNP | 1 | 168250404 | G | A | missense | TBX19 | TBX19 |  | Y |
| IM1 | somaticSNP | 1 | 175372731 | T | G | missense | TNR | TNR |  | N |
| IM1 | somaticSNP | 1 | 186122844 | A | G | intron | HMCN1,MIR548F1 |  |  | Y |
| IM1 | somaticSNP | 1 | 202113710 | C | A | 5-prime-UTR | ARL8A | ARL8A |  | Y |
| IM1 | somaticSNP | 1 | 205034434 | G | T | intron | CNTN2 |  |  | Y |
| IM1 | somaticSNP | 1 | 227227740 | A | C | intron | CDC42BPA |  |  | Y |
| IM1 | somaticSNP | 1 | 227922773 | G | C | 5-prime-UTR,missense | SNAP47,JMJD4 | SNAP47,JMJD4 |  | Y |
| IM1 | somaticSNP | 1 | 228509727 | T | A | missense | OBSCN | OBSCN |  | N |
| IM1 | somaticSNP | 1 | 228547462 | C | T | intron,missense | OBSCN | OBSCN |  | Y |
| IM1 | somaticSNP | 1 | 237773958 | A | T | intron | RYR2 |  |  | Y |
| IM1 | somaticSNP | 1 | 247921652 | G | A | intron,synonymous | OR1C1 | OR1C1 |  | Y |
| IM1 | somaticSNP | 10 | 1405432 | G | C | missense | ADARB2 | ADARB2 |  | Y |
| IM1 | somaticSNP | 10 | 15889816 | C | G | intron | FAM188A |  |  | N |
| IM1 | somaticSNP | 10 | 17085892 | T | C | missense | CUBN | CUBN |  | Y |
| IM1 | somaticSNP | 10 | 27381387 | C | T | missense | ANKRD26 | ANKRD26 |  | Y |
| IM1 | somaticSNP | 10 | 28023428 | A | T | synonymous | MKX | MKX |  | Y |
| IM1 | somaticSNP | 10 | 29581055 | T | C | intron | LYZL1 |  |  | N |
| IM1 | somaticSNP | 10 | 32326541 | A | T | intron-near-splice | KIF5B |  | KIF5B | Y |
| IM1 | somaticINDEL | 10 | 60160134 | T | -AAA | intergenic |  | |  | N |
| IM1 | somaticSNP | 10 | 75585233 | T | C | intron | CAMK2G |  |  | Y |
| IM1 | somaticSNP | 10 | 82331347 | A | G | intron | SH2D4B |  |  | Y |
| IM1 | somaticINDEL | 10 | 95454768 | T | -A | intron | FRA10AC1 |  |  | N |
| IM1 | somaticINDEL | 10 | 101978688 | T | -AAAG | intron | CHUK |  |  | Y |
| IM1 | somaticSNP | 10 | 102744480 | C | T | 3-prime-UTR,intron,missense | MRPL43,SEMA4G | SEMA4G |  | Y |
| IM1 | somaticSNP | 10 | 106039197 | G | A | intron,missense | GSTO2 | GSTO2 |  | Y |
| IM1 | somaticSNP | 10 | 116621277 | A | G | intron,missense | FAM160B1 | FAM160B1 |  | Y |
| IM1 | somaticSNP | 10 | 121287023 | C | T | intron | RGS10 |  |  | Y |
| IM1 | somaticSNP | 11 | 6412119 | A | G | synonymous | SMPD1 | SMPD1 |  | Y |
| IM1 | somaticSNP | 11 | 8642032 | T | C | synonymous | TRIM66 | TRIM66 |  | Y |
| IM1 | somaticSNP | 11 | 9075256 | T | C | intron,missense | SCUBE2 | SCUBE2 |  | Y |
| IM1 | somaticSNP | 11 | 35747593 | C | T | missense | TRIM44 | TRIM44 |  | Y |
| IM1 | somaticSNP | 11 | 57100642 | T | A | intron | SSRP1 |  |  | Y |
| IM1 | somaticSNP | 11 | 61318960 | T | C | intron | SYT7 |  |  | Y |
| IM1 | somaticSNP | 11 | 61912567 | A | C | intron-near-splice | INCENP |  |  | N |
| IM1 | somaticSNP | 11 | 64876028 | G | A | missense,non-coding-exon | VPS51 | VPS51 |  | N |
| IM1 | somaticSNP | 11 | 64939850 | T | C | intron | SPDYC |  |  | Y |
| IM1 | somaticSNP | 11 | 73536698 | A | C | intron | MRPL48 |  |  | Y |
| IM1 | somaticSNP | 11 | 77825342 | G | T | synonymous | ALG8,RNU6-83P | ALG8 |  | Y |
| IM1 | somaticSNP | 11 | 101375549 | T | C | intron | TRPC6 |  |  | Y |
| IM1 | somaticSNP | 11 | 106807392 | C | A | intron,missense | GUCY1A2 |  |  | Y |
| IM1 | somaticSNP | 11 | 113239077 | A | T | intron-near-splice,intron,3-prime-UTR | TTC12 |  |  | Y |
| IM1 | somaticSNP | 11 | 130079615 | G | T | missense | ST14 | ST14 |  | Y |
| IM1 | somaticSNP | 12 | 8758100 | C | T | intron | AICDA |  |  | Y |
| IM1 | somaticSNP | 12 | 10150721 | A | T | intron | CLEC1B |  |  | Y |
| IM1 | somaticSNP | 12 | 11286184 | G | A | synonymous | PRH1-PRR4,TAS2R30,PRH1 | TAS2R30 |  | Y |
| IM1 | somaticSNP | 12 | 21695584 | A | G | intron | GYS2 |  |  | N |
| IM1 | somaticSNP | 12 | 31255227 | A | G | synonymous | DDX11 | DDX11 |  | Y |
| IM1 | somaticSNP | 12 | 52882317 | C | T | missense | KRT6A | KRT6A |  | Y |
| IM1 | somaticSNP | 12 | 53238112 | C | T | intron | KRT78 |  |  | Y |
| IM1 | somaticSNP | 12 | 64488721 | C | A | missense | SRGAP1 | SRGAP1 |  | Y |
| IM1 | somaticSNP | 12 | 72893286 | G | T | missense | TRHDE | TRHDE |  | Y |
| IM1 | somaticSNP | 12 | 80732939 | A | T | missense | OTOGL | OTOGL |  | Y |
| IM1 | somaticSNP | 12 | 96932190 | T | A | missense | LOC101928871 | LOC101928871 |  | Y |
| IM1 | somaticSNP | 12 | 104025383 | C | A | missense | STAB2 | STAB2 |  | Y |
| IM1 | somaticSNP | 12 | 132502115 | A | C | missense | EP400 | EP400 |  | Y |
| IM1 | somaticSNP | 12 | 133808015 | T | C | intron | ANHX |  |  | Y |
| IM1 | somaticSNP | 13 | 20277413 | T | C | missense | PSPC1 | PSPC1 |  | Y |
| IM1 | somaticSNP | 13 | 26117437 | C | T | missense | ATP8A2 | ATP8A2 |  | Y |
| IM1 | somaticSNP | 13 | 26133834 | A | C | intron | ATP8A2 |  |  | Y |
| IM1 | somaticSNP | 13 | 36424921 | A | G | intron,intron-near-splice | MIR548F5,DCLK1 |  |  | Y |
| IM1 | somaticSNP | 13 | 37619503 | C | A | missense | SUPT20H | SUPT20H |  | Y |
| IM1 | somaticSNP | 13 | 46104056 | G | A | intron-near-splice,intron | COG3 |  |  | Y |
| IM1 | somaticSNP | 13 | 99540395 | A | G | intron | DOCK9 |  |  | N |
| IM1 | somaticSNP | 13 | 114240233 | T | G | intron | TFDP1 |  |  | Y |
| IM1 | somaticSNP | 14 | 22508913 | A | G | intergenic |  | |  | Y |
| IM1 | somaticSNP | 14 | 23733725 | C | T | intron | C14orf164 |  |  | Y |
| IM1 | somaticSNP | 14 | 23869882 | G | A | intron | MYH6 |  |  | Y |
| IM1 | somaticSNP | 14 | 37147839 | T | A | intron,3-prime-UTR | SLC25A21 | SLC25A21 |  | N |
| IM1 | somaticSNP | 14 | 70245100 | A | C | missense | SLC10A1 | SLC10A1 |  | Y |
| IM1 | somaticSNP | 14 | 70826226 | C | G | intron | SYNJ2BP-COX16,COX16 |  |  | Y |
| IM1 | somaticSNP | 14 | 72926278 | G | A | intron | RGS6 |  |  | Y |
| IM1 | somaticSNP | 14 | 104199580 | A | C | 3-prime-UTR | ZFYVE21 | ZFYVE21 |  | Y |
| IM1 | somaticSNP | 14 | 105613850 | T | A | missense | JAG2 | JAG2 |  | Y |
| IM1 | somaticSNP | 15 | 23891689 | C | T | missense | MAGEL2 | MAGEL2 |  | Y |
| IM1 | somaticSNP | 15 | 25449634 | A | T | upstream-gene |  | |  | Y |
| IM1 | somaticSNP | 15 | 28375351 | T | C | missense | HERC2 | HERC2 |  | Y |
| IM1 | somaticSNP | 15 | 33893830 | T | A | intron | RYR3 |  |  | Y |
| IM1 | somaticSNP | 15 | 40846225 | G | A | intron,missense | C15orf57 | C15orf57 |  | Y |
| IM1 | somaticSNP | 15 | 43528016 | C | G | missense | TGM5 | TGM5 |  | Y |
| IM1 | somaticSNP | 15 | 43889786 | T | C | intron | RNU6-28P,CKMT1B |  |  | Y |
| IM1 | somaticSNP | 15 | 48818257 | T | A | intron | FBN1 |  |  | Y |
| IM1 | somaticSNP | 15 | 75971091 | C | G | intron | CSPG4 |  |  | Y |
| IM1 | somaticSNP | 15 | 79585665 | C | A | missense | ANKRD34C | ANKRD34C |  | Y |
| IM1 | somaticSNP | 15 | 90029474 | C | T | intron | RHCG |  |  | Y |
| IM1 | somaticSNP | 16 | 332316 | G | A | synonymous | ARHGDIG | ARHGDIG |  | Y |
| IM1 | somaticSNP | 16 | 842643 | A | T | intron | CHTF18 |  |  | Y |
| IM1 | somaticSNP | 16 | 2821211 | T | C | 3-prime-UTR | SRRM2 | SRRM2 |  | Y |
| IM1 | somaticSNP | 16 | 11556121 | A | G | synonymous | LOC400499 |  |  | Y |
| IM1 | somaticSNP | 16 | 15808975 | C | T | intron | NDE1,MYH11 |  | MYH11 | Y |
| IM1 | somaticINDEL | 16 | 23593886 | A | +TGTT | intron | NDUFAB1 |  |  | Y |
| IM1 | somaticSNP | 16 | 30380445 | A | T | intron | TBC1D10B |  |  | Y |
| IM1 | somaticSNP | 16 | 31374294 | C | T | synonymous | ITGAX | ITGAX |  | Y |
| IM1 | somaticSNP | 16 | 55617059 | T | A | 3-prime-UTR | LPCAT2 | LPCAT2 |  | Y |
| IM1 | somaticSNP | 16 | 57168812 | C | A | intron | CPNE2 |  |  | Y |
| IM1 | somaticSNP | 16 | 58568227 | C | T | missense,non-coding-exon | CNOT1 | CNOT1 |  | Y |
| IM1 | somaticSNP | 16 | 71969447 | T | C | intron | PKD1L3 |  |  | Y |
| IM1 | somaticSNP | 16 | 74382863 | T | C | non-coding-exon | LOC283922 | LOC283922 |  | Y |
| IM1 | somaticSNP | 17 | 4719892 | G | T | intron | PLD2 |  |  | Y |
| IM1 | somaticSNP | 17 | 8076698 | G | C | 3-prime-UTR | TMEM107 | TMEM107 |  | Y |
| IM1 | somaticINDEL | 17 | 10550845 | T | -TTTTG | intron | MYH3 |  |  | Y |
| IM1 | somaticSNP | 17 | 11671943 | G | T | missense-near-splice | DNAH9 | DNAH9 |  | Y |
| IM1 | somaticSNP | 17 | 12852477 | C | T | synonymous | ARHGAP44 | ARHGAP44 |  | Y |
| IM1 | somaticSNP | 17 | 16040527 | T | C | intron | NCOR1 |  |  | N |
| IM1 | somaticSNP | 17 | 21207939 | G | T | intron | MAP2K3 |  |  | Y |
| IM1 | somaticSNP | 17 | 27822745 | G | T | missense-near-splice | TAOK1 | TAOK1 |  | Y |
| IM1 | somaticSNP | 17 | 28616359 | A | G | intron | BLMH |  |  | Y |
| IM1 | somaticSNP | 17 | 29161991 | G | A | missense | ATAD5 | ATAD5 |  | Y |
| IM1 | somaticSNP | 17 | 29226536 | G | T | missense | TEFM | TEFM |  | Y |
| IM1 | somaticSNP | 17 | 38910804 | T | C | intron | KRT25 |  |  | Y |
| IM1 | somaticSNP | 17 | 42335782 | T | C | synonymous-near-splice | SLC4A1 | SLC4A1 |  | Y |
| IM1 | somaticSNP | 17 | 56429045 | C | A | intron | SUPT4H1,BZRAP1-AS1 |  |  | N |
| IM1 | somaticINDEL | 17 | 56693799 | C | +T | intron | TEX14 |  |  | N |
| IM1 | somaticSNP | 17 | 57886199 | C | A | missense | VMP1 | VMP1 |  | Y |
| IM1 | somaticSNP | 17 | 58326937 | C | T | intron | USP32 |  |  | N |
| IM1 | somaticSNP | 17 | 71223299 | C | A | intron-near-splice | FAM104A |  |  | Y |
| IM1 | somaticSNP | 17 | 74079838 | A | G | intron-near-splice | EXOC7 |  |  | N |
| IM1 | somaticSNP | 17 | 75834846 | C | T | intergenic |  | |  | Y |
| IM1 | somaticSNP | 17 | 80129776 | T | C | intron | CCDC57 |  |  | Y |
| IM1 | somaticSNP | 18 | 12067950 | C | T | intergenic |  | |  | Y |
| IM1 | somaticSNP | 18 | 12103292 | T | G | intron | ANKRD62 |  |  | Y |
| IM1 | somaticSNP | 18 | 74620187 | A | T | intron | ZNF236 |  |  | Y |
| IM1 | somaticSNP | 19 | 9007560 | A | G | intron-near-splice | MUC16 |  |  | Y |
| IM1 | somaticSNP | 19 | 15276007 | C | A | intron | NOTCH3 |  |  | Y |
| IM1 | somaticSNP | 19 | 33359482 | C | T | 5-prime-UTR,intron | SLC7A9 | SLC7A9 |  | Y |
| IM1 | somaticSNP | 19 | 34663663 | C | T | missense | LSM14A | LSM14A |  | Y |
| IM1 | somaticINDEL | 19 | 35832910 | G | -TCCT | intron | CD22 |  |  | N |
| IM1 | somaticSNP | 19 | 37368933 | A | T | stop-gained,intron | ZNF345 | ZNF345 |  | Y |
| IM1 | somaticSNP | 19 | 38692623 | C | T | synonymous | SIPA1L3 | SIPA1L3 |  | Y |
| IM1 | somaticSNP | 19 | 41916759 | G | A | intron | BCKDHA |  |  | Y |
| IM1 | somaticSNP | 19 | 42814990 | G | A | upstream-gene |  | |  | N |
| IM1 | somaticSNP | 19 | 45147340 | G | T | 5-prime-UTR | PVR | PVR |  | Y |
| IM1 | somaticSNP | 19 | 46811328 | T | C | intron | HIF3A |  |  | Y |
| IM1 | somaticSNP | 19 | 47763712 | G | C | intron | CCDC9 |  |  | Y |
| IM1 | somaticSNP | 19 | 49303120 | C | A | intron | BCAT2 |  |  | Y |
| IM1 | somaticSNP | 19 | 50250504 | C | A | 5-prime-UTR,intron | TSKS |  |  | Y |
| IM1 | somaticSNP | 19 | 50266498 | T | A | missense | TSKS | TSKS |  | Y |
| IM1 | somaticSNP | 19 | 50463727 | G | A | intron | SIGLEC11 |  |  | Y |
| IM1 | somaticSNP | 2 | 26204388 | G | A | synonymous | KIF3C | KIF3C |  | Y |
| IM1 | somaticSNP | 2 | 29379290 | A | C | missense | CLIP4 | CLIP4 |  | Y |
| IM1 | somaticSNP | 2 | 31412096 | T | C | intron | CAPN14 |  |  | Y |
| IM1 | somaticSNP | 2 | 31483585 | T | C | missense | EHD3 | EHD3 |  | Y |
| IM1 | somaticSNP | 2 | 54891911 | A | T | intron | SPTBN1 |  |  | Y |
| IM1 | somaticSNP | 2 | 55433542 | T | C | intron | CLHC1 |  |  | Y |
| IM1 | somaticSNP | 2 | 86422665 | A | G | 5-prime-UTR | IMMT | IMMT |  | N |
| IM1 | somaticSNP | 2 | 98833151 | T | G | intron | VWA3B |  |  | Y |
| IM1 | somaticSNP | 2 | 116447348 | A | G | intron | DPP10 |  |  | N |
| IM1 | somaticSNP | 2 | 118701541 | T | C | intron | CCDC93 |  |  | Y |
| IM1 | somaticSNP | 2 | 128522167 | G | A | intron,synonymous | WDR33 | WDR33 |  | Y |
| IM1 | somaticSNP | 2 | 151331371 | T | C | intron | RND3 |  |  | Y |
| IM1 | somaticSNP | 2 | 153551068 | C | T | synonymous | PRPF40A | PRPF40A |  | Y |
| IM1 | somaticSNP | 2 | 153574008 | C | A | 5-prime-UTR,missense | PRPF40A | PRPF40A |  | Y |
| IM1 | somaticSNP | 2 | 166908325 | G | T | missense | SCN1A | SCN1A |  | Y |
| IM1 | somaticSNP | 2 | 176857148 | T | A | splice-3 | KIAA1715 |  |  | N |
| IM1 | somaticSNP | 2 | 177034400 | G | A | intron | HOXD3 |  |  | Y |
| IM1 | somaticSNP | 2 | 179442805 | C | G | missense | MIR548N,TTN-AS1,TTN | TTN |  | Y |
| IM1 | somaticINDEL | 2 | 197643854 | C | -T | intron | GTF3C3 |  |  | Y |
| IM1 | somaticSNP | 2 | 201756675 | A | G | intron,synonymous | NIF3L1 | NIF3L1 |  | Y |
| IM1 | somaticINDEL | 2 | 201778717 | T | +A | intron | ORC2 |  |  | N |
| IM1 | somaticSNP | 2 | 210877639 | A | T | intron | RPE |  |  | Y |
| IM1 | somaticSNP | 2 | 220358383 | G | T | upstream-gene |  | |  | Y |
| IM1 | somaticSNP | 2 | 228846479 | T | G | missense | SPHKAP | SPHKAP |  | Y |
| IM1 | somaticSNP | 2 | 231152571 | A | T | intron | SP140 |  |  | Y |
| IM1 | somaticSNP | 2 | 236708207 | A | G | intron | AGAP1 |  |  | Y |
| IM1 | somaticSNP | 2 | 242800900 | G | A | intron | PDCD1 |  |  | Y |
| IM1 | somaticSNP | 20 | 3219894 | A | G | intron | SLC4A11 |  |  | N |
| IM1 | somaticSNP | 20 | 6060118 | T | C | missense | FERMT1 | FERMT1 |  | N |
| IM1 | somaticSNP | 20 | 31887621 | A | T | intron | BPIFB1 |  |  | N |
| IM1 | somaticSNP | 20 | 32868834 | T | A | 3-prime-UTR | AHCY | AHCY |  | Y |
| IM1 | somaticSNP | 20 | 34204981 | C | T | intron | SPAG4 |  |  | Y |
| IM1 | somaticSNP | 20 | 37529300 | G | A | missense | PPP1R16B | PPP1R16B |  | Y |
| IM1 | somaticINDEL | 20 | 43249004 | A | -TCT | nonsynonymous,del,frameshift | ADA | ADA |  | Y |
| IM1 | somaticINDEL | 20 | 46281139 | A | -T | intron | NCOA3 |  |  | N |
| IM1 | somaticSNP | 20 | 46295240 | C | T | intron-near-splice | SULF2 |  |  | Y |
| IM1 | somaticSNP | 20 | 49202537 | G | C | downstream-gene |  | |  | Y |
| IM1 | somaticSNP | 20 | 49225000 | C | A | synonymous | FAM65C | FAM65C |  | Y |
| IM1 | somaticSNP | 20 | 55803351 | C | T | missense | BMP7 | BMP7 |  | Y |
| IM1 | somaticSNP | 20 | 55908207 | T | C | intron | SPO11 |  |  | Y |
| IM1 | somaticSNP | 20 | 60503171 | C | T | intron | CDH4 |  |  | Y |
| IM1 | somaticSNP | 21 | 16340367 | C | A | missense | NRIP1 | NRIP1 |  | Y |
| IM1 | somaticSNP | 21 | 19642346 | G | A | synonymous | TMPRSS15 | TMPRSS15 |  | Y |
| IM1 | somaticSNP | 21 | 31798199 | G | C | missense | KRTAP13-3 | KRTAP13-3 |  | Y |
| IM1 | somaticSNP | 21 | 33039493 | A | C | intron | SOD1 |  |  | Y |
| IM1 | somaticINDEL | 21 | 35186127 | C | -T | intron | ITSN1 |  |  | N |
| IM1 | somaticSNP | 21 | 41719719 | G | T | missense,non-coding-exon | DSCAM | DSCAM |  | Y |
| IM1 | somaticSNP | 21 | 43541206 | A | C | missense | UMODL1 | UMODL1 |  | Y |
| IM1 | somaticSNP | 21 | 44478157 | C | G | intron | CBS |  |  | Y |
| IM1 | somaticSNP | 22 | 23041026 | G | A | intergenic |  | |  | Y |
| IM1 | somaticSNP | 22 | 25603127 | G | A | missense | CRYBB3 | CRYBB3 |  | Y |
| IM1 | somaticSNP | 22 | 31335601 | C | A | intron-near-splice | MORC2 |  |  | Y |
| IM1 | somaticSNP | 22 | 36690459 | T | C | intron | MYH9 |  | MYH9 | N |
| IM1 | somaticSNP | 22 | 38622957 | C | A | intron | TMEM184B |  |  | Y |
| IM1 | somaticSNP | 22 | 40661645 | G | T | intron,missense,missense | TNRC6B | TNRC6B |  | Y |
| IM1 | somaticINDEL | 22 | 42289405 | C | +GTTT | intron | SREBF2 |  |  | N |
| IM1 | somaticSNP | 22 | 42415519 | A | G | intron | WBP2NL |  |  | Y |
| IM1 | somaticSNP | 22 | 44594723 | T | C | intron | PARVG |  |  | Y |
| IM1 | somaticSNP | 22 | 50469385 | G | A | intron | TTLL8 |  |  | Y |
| IM1 | somaticSNP | 3 | 403347 | A | G | intron | CHL1 |  |  | Y |
| IM1 | somaticINDEL | 3 | 19389266 | C | +A | nonsynonymous,ins,frameshift | KCNH8 | KCNH8 |  | N |
| IM1 | somaticSNP | 3 | 38051009 | C | T | intron | PLCD1 |  |  | N |
| IM1 | somaticSNP | 3 | 39448229 | A | G | 5-prime-UTR | RPSA | RPSA |  | Y |
| IM1 | somaticSNP | 3 | 44283610 | T | A | missense | TOPAZ1 | TOPAZ1 |  | Y |
| IM1 | somaticSNP | 3 | 49311526 | T | A | missense | C3orf62 | C3orf62 |  | Y |
| IM1 | somaticINDEL | 3 | 57108374 | T | -C | nonsynonymous,del,frameshift | SPATA12,ARHGEF3 | SPATA12 |  | N |
| IM1 | somaticSNP | 3 | 57447331 | C | A | missense | DNAH12 | DNAH12 |  | Y |
| IM1 | somaticSNP | 3 | 69112049 | C | T | intron | UBA3 |  |  | N |
| IM1 | somaticSNP | 3 | 97464291 | T | A | intron | EPHA6 |  |  | Y |
| IM1 | somaticSNP | 3 | 100084408 | T | C | synonymous | TOMM70A | TOMM70A |  | Y |
| IM1 | somaticSNP | 3 | 121132268 | T | A | intron | STXBP5L |  |  | Y |
| IM1 | somaticSNP | 3 | 124449461 | G | A | 5-prime-UTR,missense,non-coding-exon | UMPS | UMPS |  | Y |
| IM1 | somaticSNP | 3 | 133877257 | C | T | intron | RYK |  |  | Y |
| IM1 | somaticSNP | 3 | 142840069 | T | A | synonymous | CHST2 | CHST2 |  | Y |
| IM1 | somaticSNP | 3 | 167413491 | T | G | synonymous | PDCD10 | PDCD10 |  | Y |
| IM1 | somaticSNP | 3 | 184299095 | A | C | synonymous | EPHB3 | EPHB3 |  | Y |
| IM1 | somaticSNP | 3 | 185213018 | T | G | missense | TMEM41A | TMEM41A |  | Y |
| IM1 | somaticSNP | 3 | 185879581 | T | A | intron | DGKG |  |  | Y |
| IM1 | somaticSNP | 3 | 190995829 | A | T | intron | UTS2B |  |  | N |
| IM1 | somaticSNP | 3 | 194118705 | G | A | synonymous | GP5 | GP5 |  | Y |
| IM1 | somaticSNP | 3 | 195517843 | G | A | intron,missense | MUC4 | MUC4 |  | Y |
| IM1 | somaticSNP | 4 | 2900240 | G | A | synonymous | ADD1 | ADD1 |  | Y |
| IM1 | somaticSNP | 4 | 10020564 | G | A | intron | SLC2A9 |  |  | Y |
| IM1 | somaticINDEL | 4 | 26241366 | G | +GGAGA | intron | RBPJ |  |  | N |
| IM1 | somaticSNP | 4 | 48546969 | T | G | intron | FRYL |  |  | Y |
| IM1 | somaticSNP | 4 | 73164017 | T | A | missense | ADAMTS3 | ADAMTS3 |  | Y |
| IM1 | somaticSNP | 4 | 90034354 | G | C | missense | TIGD2 | TIGD2 |  | Y |
| IM1 | somaticSNP | 4 | 103556316 | T | C | intron | MANBA |  |  | Y |
| IM1 | somaticSNP | 4 | 122068167 | T | C | intron | TNIP3 |  |  | N |
| IM1 | somaticSNP | 4 | 125631753 | T | C | 5-prime-UTR,intron | ANKRD50 | ANKRD50 |  | Y |
| IM1 | somaticSNP | 4 | 126240096 | G | A | missense | FAT4 | FAT4 |  | Y |
| IM1 | somaticSNP | 4 | 155507484 | T | A | missense | FGA | FGA |  | Y |
| IM1 | somaticSNP | 4 | 155533047 | A | G | missense | FGG | FGG |  | Y |
| IM1 | somaticSNP | 5 | 7414718 | A | G | synonymous | ADCY2 | ADCY2 |  | Y |
| IM1 | somaticSNP | 5 | 16701535 | T | G | missense | MYO10 | MYO10 |  | Y |
| IM1 | somaticSNP | 5 | 37168957 | A | G | missense | C5orf42 | C5orf42 |  | Y |
| IM1 | somaticINDEL | 5 | 78416973 | C | -A | intron | BHMT |  |  | N |
| IM1 | somaticSNP | 5 | 101813531 | G | C | missense | SLCO6A1 | SLCO6A1 |  | Y |
| IM1 | somaticSNP | 5 | 132052648 | T | C | synonymous | KIF3A | KIF3A |  | Y |
| IM1 | somaticSNP | 5 | 135396468 | T | A | intron | TGFBI |  |  | N |
| IM1 | somaticSNP | 5 | 141059904 | G | T | missense | ARAP3 | ARAP3 |  | Y |
| IM1 | somaticSNP | 5 | 145531578 | G | A | intron | LARS |  |  | Y |
| IM1 | somaticSNP | 5 | 149112300 | T | G | intron | PPARGC1B |  |  | Y |
| IM1 | somaticSNP | 5 | 154396804 | T | A | missense | KIF4B | KIF4B |  | Y |
| IM1 | somaticSNP | 5 | 169309494 | T | A | intron | DOCK2,FAM196B |  |  | Y |
| IM1 | somaticSNP | 5 | 169412889 | C | T | missense | DOCK2 | DOCK2 |  | Y |
| IM1 | somaticSNP | 5 | 172110938 | G | A | intron,missense | NEURL1B | NEURL1B |  | Y |
| IM1 | somaticSNP | 6 | 5000779 | G | A | intron | RPP40 |  |  | N |
| IM1 | somaticSNP | 6 | 11104238 | C | A | stop-gained,intron | SMIM13,ERVFRD-1 | ERVFRD-1 |  | Y |
| IM1 | somaticSNP | 6 | 28197605 | C | T | intron | ZSCAN9 |  |  | N |
| IM1 | somaticSNP | 6 | 33653560 | C | A | missense | ITPR3 | ITPR3 |  | N |
| IM1 | somaticSNP | 6 | 34029881 | C | T | intron | GRM4 |  |  | N |
| IM1 | somaticSNP | 6 | 71233675 | A | G | missense | FAM135A | FAM135A |  | Y |
| IM1 | somaticSNP | 6 | 75801024 | G | T | intron | COL12A1 |  |  | Y |
| IM1 | somaticSNP | 6 | 99848727 | T | C | missense | PNISR | PNISR |  | Y |
| IM1 | somaticSNP | 6 | 108831414 | G | A | splice-acceptor | LACE1 | LACE1 |  | Y |
| IM1 | somaticSNP | 6 | 116441709 | C | G | missense,intron | COL10A1,NT5DC1 | COL10A1 |  | Y |
| IM1 | somaticSNP | 6 | 129799721 | T | C | intron | LAMA2 |  |  | Y |
| IM1 | somaticSNP | 6 | 132874297 | T | A | missense | TAAR8 | TAAR8 |  | Y |
| IM1 | somaticSNP | 6 | 144508629 | C | A | 3-prime-UTR | STX11 | STX11 |  | Y |
| IM1 | somaticSNP | 6 | 149771895 | T | A | missense | ZC3H12D | ZC3H12D |  | Y |
| IM1 | somaticSNP | 6 | 167426949 | A | G | intron | FGFR1OP |  | FGFR1OP | Y |
| IM1 | somaticSNP | 6 | 167446139 | A | G | intron | FGFR1OP |  | FGFR1OP | Y |
| IM1 | somaticSNP | 6 | 170034675 | A | T | intron | WDR27 |  |  | Y |
| IM1 | somaticSNP | 6 | 170049362 | C | A | missense | WDR27 | WDR27 |  | Y |
| IM1 | somaticSNP | 7 | 1914350 | A | G | intron | MAD1L1 |  |  | Y |
| IM1 | somaticSNP | 7 | 4304941 | G | T | intron,missense | SDK1 | SDK1 |  | Y |
| IM1 | somaticSNP | 7 | 12409830 | T | C | missense | VWDE | VWDE |  | N |
| IM1 | somaticSNP | 7 | 12610475 | C | T | synonymous | SCIN | SCIN |  | Y |
| IM1 | somaticSNP | 7 | 19156335 | G | T | 3-prime-UTR | TWIST1 | TWIST1 |  | Y |
| IM1 | somaticINDEL | 7 | 22330862 | T | +A | intron | RAPGEF5 |  |  | N |
| IM1 | somaticSNP | 7 | 26404329 | T | C | intron | SNX10 |  |  | Y |
| IM1 | somaticSNP | 7 | 44619235 | G | A | intron-near-splice | TMED4 |  |  | Y |
| IM1 | somaticSNP | 7 | 48146967 | G | A | missense | UPP1 | UPP1 |  | Y |
| IM1 | somaticSNP | 7 | 63726690 | C | A | missense | ZNF679 | ZNF679 |  | Y |
| IM1 | somaticSNP | 7 | 81631020 | T | A | intron | CACNA2D1 |  |  | Y |
| IM1 | somaticSNP | 7 | 99578411 | T | C | non-coding-exon | AZGP1P1 | AZGP1P1 |  | Y |
| IM1 | somaticSNP | 7 | 107332509 | G | A | intron,missense | SLC26A4 |  |  | Y |
| IM1 | somaticSNP | 7 | 107599765 | G | A | synonymous | LAMB1 | LAMB1 |  | Y |
| IM1 | somaticINDEL | 7 | 134930169 | A | +GAG | intron | STRA8 |  |  | Y |
| IM1 | somaticSNP | 7 | 142045484 | C | A | intergenic |  | |  | Y |
| IM1 | somaticSNP | 7 | 142561350 | G | T | intron | EPHB6 |  |  | Y |
| IM1 | somaticSNP | 7 | 143095395 | C | T | intron | EPHA1 |  |  | Y |
| IM1 | somaticSNP | 7 | 143573132 | C | T | intron,synonymous | FAM115A | FAM115A |  | Y |
| IM1 | somaticSNP | 7 | 149525600 | G | T | intron | SSPO |  |  | Y |
| IM1 | somaticSNP | 8 | 28821353 | T | C | 5-prime-UTR | HMBOX1 | HMBOX1 |  | Y |
| IM1 | somaticSNP | 8 | 39103668 | C | G | missense | ADAM32 | ADAM32 |  | Y |
| IM1 | somaticSNP | 8 | 69351923 | A | C | intron | C8orf34 |  |  | Y |
| IM1 | somaticSNP | 8 | 73150337 | A | T | non-coding-exon | LOC392232 | LOC392232 |  | Y |
| IM1 | somaticSNP | 8 | 73479912 | T | A | 5-prime-UTR | KCNB2 | KCNB2 |  | Y |
| IM1 | somaticSNP | 8 | 113418760 | A | T | synonymous | CSMD3 | CSMD3 |  | Y |
| IM1 | somaticSNP | 8 | 125047646 | C | T | intron,synonymous | FER1L6,FER1L6-AS1 | FER1L6 |  | N |
| IM1 | somaticSNP | 8 | 131964000 | C | A | intron | ADCY8 |  |  | Y |
| IM1 | somaticSNP | 8 | 141712834 | T | C | intron | PTK2 |  |  | N |
| IM1 | somaticSNP | 8 | 143614849 | G | T | intron | BAI1 |  |  | Y |
| IM1 | somaticSNP | 8 | 146220171 | T | C | intron | ZNF252P |  |  | Y |
| IM1 | somaticSNP | 9 | 84202674 | C | G | synonymous | TLE1 | TLE1 |  | Y |
| IM1 | somaticSNP | 9 | 86895009 | G | T | intron | SLC28A3 |  |  | Y |
| IM1 | somaticSNP | 9 | 91360763 | G | A | non-coding-exon | MIR4289 | MIR4289 |  | Y |
| IM1 | somaticSNP | 9 | 98766997 | G | T | intron | ERCC6L2 |  |  | N |
| IM1 | somaticSNP | 9 | 99314276 | G | C | intron | CDC14B |  |  | Y |
| IM1 | somaticSNP | 9 | 105763940 | T | C | intron | CYLC2 |  |  | Y |
| IM1 | somaticSNP | 9 | 111693233 | C | A | intron | IKBKAP |  |  | Y |
| IM1 | somaticSNP | 9 | 112172661 | G | C | missense,non-coding-exon | PTPN3 | PTPN3 |  | Y |
| IM1 | somaticSNP | 9 | 113341468 | G | T | missense | SVEP1 | SVEP1 |  | Y |
| IM1 | somaticSNP | 9 | 130860849 | C | T | intron,missense,non-coding-exon | SLC25A25 | SLC25A25 |  | Y |
| IM1 | somaticSNP | 9 | 131223392 | C | A | intron | ODF2 |  |  | Y |
| IM1 | somaticSNP | 9 | 133554034 | G | C | intron-near-splice | PRDM12 |  |  | Y |
| IM1 | somaticSNP | 9 | 139396088 | C | A | intron | NOTCH1 |  | NOTCH1 | Y |
| IM1 | somaticSNP | 9 | 139904603 | T | A | intron | ABCA2 |  |  | Y |
| IM1 | somaticSNP | X | 35985729 | A | G | intron | CXorf22 |  |  | Y |
| IM1 | somaticSNP | X | 50053087 | T | A | intron,missense | CCNB3 | CCNB3 |  | Y |
| IM1 | somaticSNP | X | 53250052 | G | A | 5-prime-UTR,intron,missense | KDM5C | KDM5C | KDM5C | Y |
| IM1 | somaticSNP | X | 117815120 | A | G | missense | DOCK11 | DOCK11 |  | Y |
| IM1 | somaticSNP | X | 125686072 | G | T | missense | DCAF12L1 | DCAF12L1 |  | Y |
| IM1 | somaticSNP | X | 153236100 | C | T | synonymous-near-splice | HCFC1 | HCFC1 |  | Y |
| IM2 | somaticSNP | 1 | 15855461 | T | G | intron | DNAJC16 |  |  | Y |
| IM2 | somaticSNP | 1 | 17023483 | C | T | intron | ESPNP |  |  | Y |
| IM2 | somaticSNP | 1 | 17267496 | C | T | intron | CROCC |  |  | Y |
| IM2 | somaticSNP | 1 | 19062153 | G | A | missense | PAX7 | PAX7 | PAX7 | N |
| IM2 | somaticSNP | 1 | 21012616 | G | A | missense | KIF17 | KIF17 |  | Y |
| IM2 | somaticINDEL | 1 | 25780889 | A | +TG | intron | TMEM57 |  |  | N |
| IM2 | somaticSNP | 1 | 26738094 | T | G | intron | LIN28A |  |  | Y |
| IM2 | somaticSNP | 1 | 26873700 | C | T | 5-prime-UTR,synonymous | RPS6KA1 | RPS6KA1 |  | Y |
| IM2 | somaticSNP | 1 | 29611123 | C | T | intron | PTPRU |  |  | N |
| IM2 | somaticSNP | 1 | 32150075 | T | G | intron | COL16A1 |  |  | Y |
| IM2 | somaticSNP | 1 | 34180246 | C | T | missense | CSMD2 | CSMD2 |  | Y |
| IM2 | somaticSNP | 1 | 36939412 | T | G | synonymous | CSF3R | CSF3R | CSF3R | Y |
| IM2 | somaticSNP | 1 | 41327871 | C | A | 5-prime-UTR | CITED4 | CITED4 |  | Y |
| IM2 | somaticSNP | 1 | 53279594 | A | G | intron | ZYG11B |  |  | Y |
| IM2 | somaticSNP | 1 | 62503632 | A | G | intron | INADL |  |  | Y |
| IM2 | somaticSNP | 1 | 67559220 | T | C | 3-prime-UTR,missense,non-coding-exon | C1orf141 | C1orf141 |  | Y |
| IM2 | somaticSNP | 1 | 78267110 | G | A | missense | FAM73A | FAM73A |  | N |
| IM2 | somaticINDEL | 1 | 93159821 | C | -A | intron | EVI5 |  |  | N |
| IM2 | somaticSNP | 1 | 103385782 | T | C | intron | COL11A1 |  |  | Y |
| IM2 | somaticSNP | 1 | 110129627 | G | T | intron | GNAI3 |  |  | N |
| IM2 | somaticSNP | 1 | 111217382 | C | T | missense | KCNA3 | KCNA3 |  | N |
| IM2 | somaticSNP | 1 | 121116733 | A | G | intergenic | SRGAP2D,SRGAP2-AS1 | SRGAP2D |  | Y |
| IM2 | somaticSNP | 1 | 145281824 | T | G | intron | NBPF20,NBPF9,NOTCH2NL |  |  | Y |
| IM2 | somaticSNP | 1 | 153642826 | C | T | intron | ILF2 |  |  | Y |
| IM2 | somaticSNP | 1 | 156845864 | C | A | intron-near-splice,synonymous-near-splice | NTRK1 |  | NTRK1 | Y |
| IM2 | somaticSNP | 1 | 157514235 | C | G | missense | FCRL5 | FCRL5 |  | Y |
| IM2 | somaticSNP | 1 | 158435706 | G | T | missense | OR10K1 | OR10K1 |  | Y |
| IM2 | somaticSNP | 1 | 158687102 | A | G | synonymous | OR6K3 | OR6K3 |  | Y |
| IM2 | somaticSNP | 1 | 165180036 | T | C | intron | LMX1A |  |  | Y |
| IM2 | somaticSNP | 1 | 166959161 | T | C | intron | MAEL |  |  | Y |
| IM2 | somaticSNP | 1 | 168250404 | G | A | missense | TBX19 | TBX19 |  | N |
| IM2 | somaticSNP | 1 | 175372731 | T | G | missense | TNR | TNR |  | Y |
| IM2 | somaticSNP | 1 | 186122844 | A | G | intron | HMCN1,MIR548F1 |  |  | Y |
| IM2 | somaticSNP | 1 | 202113710 | C | A | 5-prime-UTR | ARL8A | ARL8A |  | Y |
| IM2 | somaticSNP | 1 | 205034434 | G | T | intron | CNTN2 |  |  | Y |
| IM2 | somaticSNP | 1 | 227227740 | A | C | intron | CDC42BPA |  |  | N |
| IM2 | somaticSNP | 1 | 227922773 | G | C | 5-prime-UTR,missense | SNAP47,JMJD4 | SNAP47,JMJD4 |  | N |
| IM2 | somaticSNP | 1 | 228509727 | T | A | missense | OBSCN | OBSCN |  | Y |
| IM2 | somaticSNP | 1 | 228547462 | C | T | intron,missense | OBSCN | OBSCN |  | Y |
| IM2 | somaticSNP | 1 | 237773958 | A | T | intron | RYR2 |  |  | N |
| IM2 | somaticSNP | 1 | 247921652 | G | A | intron,synonymous | OR1C1 | OR1C1 |  | Y |
| IM2 | somaticSNP | 10 | 1405432 | G | C | missense | ADARB2 | ADARB2 |  | Y |
| IM2 | somaticSNP | 10 | 15889816 | C | G | intron | FAM188A |  |  | Y |
| IM2 | somaticSNP | 10 | 17085892 | T | C | missense | CUBN | CUBN |  | Y |
| IM2 | somaticSNP | 10 | 27381387 | C | T | missense | ANKRD26 | ANKRD26 |  | Y |
| IM2 | somaticSNP | 10 | 28023428 | A | T | synonymous | MKX | MKX |  | Y |
| IM2 | somaticSNP | 10 | 29581055 | T | C | intron | LYZL1 |  |  | Y |
| IM2 | somaticSNP | 10 | 32326541 | A | T | intron-near-splice | KIF5B |  | KIF5B | Y |
| IM2 | somaticSNP | 10 | 75585233 | T | C | intron | CAMK2G |  |  | N |
| IM2 | somaticSNP | 10 | 82331347 | A | G | intron | SH2D4B |  |  | Y |
| IM2 | somaticINDEL | 10 | 93776240 | C | +T | intron | BTAF1 |  |  | N |
| IM2 | somaticINDEL | 10 | 95454768 | T | -A | intron | FRA10AC1 |  |  | Y |
| IM2 | somaticINDEL | 10 | 101978688 | T | -AAAG | intron | CHUK |  |  | N |
| IM2 | somaticSNP | 10 | 102744480 | C | T | 3-prime-UTR,intron,missense | MRPL43,SEMA4G | SEMA4G |  | Y |
| IM2 | somaticSNP | 10 | 106039197 | G | A | intron,missense | GSTO2 | GSTO2 |  | Y |
| IM2 | somaticSNP | 10 | 116621277 | A | G | intron,missense | FAM160B1 | FAM160B1 |  | Y |
| IM2 | somaticSNP | 10 | 121287023 | C | T | intron | RGS10 |  |  | Y |
| IM2 | somaticSNP | 10 | 125425914 | G | A | 5-prime-UTR | GPR26 | GPR26 |  | Y |
| IM2 | somaticSNP | 11 | 6412119 | A | G | synonymous | SMPD1 | SMPD1 |  | Y |
| IM2 | somaticSNP | 11 | 8642032 | T | C | synonymous | TRIM66 | TRIM66 |  | Y |
| IM2 | somaticSNP | 11 | 9075256 | T | C | intron,missense | SCUBE2 | SCUBE2 |  | Y |
| IM2 | somaticSNP | 11 | 35747593 | C | T | missense | TRIM44 | TRIM44 |  | Y |
| IM2 | somaticSNP | 11 | 57100642 | T | A | intron | SSRP1 |  |  | N |
| IM2 | somaticSNP | 11 | 61318960 | T | C | intron | SYT7 |  |  | Y |
| IM2 | somaticSNP | 11 | 61912567 | A | C | intron-near-splice | INCENP |  |  | N |
| IM2 | somaticSNP | 11 | 64876028 | G | A | missense,non-coding-exon | VPS51 | VPS51 |  | Y |
| IM2 | somaticSNP | 11 | 64939850 | T | C | intron | SPDYC |  |  | Y |
| IM2 | somaticSNP | 11 | 73536698 | A | C | intron | MRPL48 |  |  | Y |
| IM2 | somaticSNP | 11 | 77825342 | G | T | synonymous | ALG8,RNU6-83P | ALG8 |  | Y |
| IM2 | somaticSNP | 11 | 101375549 | T | C | intron | TRPC6 |  |  | Y |
| IM2 | somaticSNP | 11 | 106807392 | C | A | intron,missense | GUCY1A2 |  |  | Y |
| IM2 | somaticSNP | 11 | 113239077 | A | T | intron-near-splice,intron,3-prime-UTR | TTC12 |  |  | Y |
| IM2 | somaticSNP | 11 | 130079615 | G | T | missense | ST14 | ST14 |  | N |
| IM2 | somaticSNP | 12 | 8758100 | C | T | intron | AICDA |  |  | Y |
| IM2 | somaticSNP | 12 | 10150721 | A | T | intron | CLEC1B |  |  | Y |
| IM2 | somaticSNP | 12 | 11286184 | G | A | synonymous | PRH1-PRR4,TAS2R30,PRH1 | TAS2R30 |  | Y |
| IM2 | somaticSNP | 12 | 21695584 | A | G | intron | GYS2 |  |  | Y |
| IM2 | somaticSNP | 12 | 31255227 | A | G | synonymous | DDX11 | DDX11 |  | Y |
| IM2 | somaticSNP | 12 | 48370448 | C | T | intron | COL2A1 |  |  | Y |
| IM2 | somaticSNP | 12 | 52882317 | C | T | missense | KRT6A | KRT6A |  | Y |
| IM2 | somaticSNP | 12 | 53238112 | C | T | intron | KRT78 |  |  | Y |
| IM2 | somaticSNP | 12 | 64488721 | C | A | missense | SRGAP1 | SRGAP1 |  | N |
| IM2 | somaticSNP | 12 | 72893286 | G | T | missense | TRHDE | TRHDE |  | Y |
| IM2 | somaticSNP | 12 | 80732939 | A | T | missense | OTOGL | OTOGL |  | Y |
| IM2 | somaticSNP | 12 | 96932190 | T | A | missense | LOC101928871 | LOC101928871 |  | Y |
| IM2 | somaticSNP | 12 | 104025383 | C | A | missense | STAB2 | STAB2 |  | Y |
| IM2 | somaticSNP | 12 | 104698758 | T | A | 3-prime-UTR,intron,intron,intron,intron,intron,intron,intron | EID3,TXNRD1 | EID3 |  | Y |
| IM2 | somaticSNP | 12 | 132502115 | A | C | missense | EP400 | EP400 |  | Y |
| IM2 | somaticSNP | 12 | 133808015 | T | C | intron | ANHX |  |  | Y |
| IM2 | somaticSNP | 13 | 20277413 | T | C | missense | PSPC1 | PSPC1 |  | N |
| IM2 | somaticSNP | 13 | 26117437 | C | T | missense | ATP8A2 | ATP8A2 |  | Y |
| IM2 | somaticSNP | 13 | 26133834 | A | C | intron | ATP8A2 |  |  | Y |
| IM2 | somaticSNP | 13 | 36424921 | A | G | intron,intron-near-splice | MIR548F5,DCLK1 |  |  | Y |
| IM2 | somaticSNP | 13 | 37619503 | C | A | missense | SUPT20H | SUPT20H |  | Y |
| IM2 | somaticSNP | 13 | 46104056 | G | A | intron-near-splice,intron | COG3 |  |  | Y |
| IM2 | somaticSNP | 13 | 99540395 | A | G | intron | DOCK9 |  |  | Y |
| IM2 | somaticSNP | 13 | 114240233 | T | G | intron | TFDP1 |  |  | Y |
| IM2 | somaticSNP | 14 | 22508913 | A | G | intergenic |  | |  | Y |
| IM2 | somaticSNP | 14 | 23733725 | C | T | intron | C14orf164 |  |  | Y |
| IM2 | somaticSNP | 14 | 23869882 | G | A | intron | MYH6 |  |  | Y |
| IM2 | somaticINDEL | 14 | 30095782 | G | +A | intron | PRKD1,MIR548AI |  |  | N |
| IM2 | somaticSNP | 14 | 37147839 | T | A | intron,3-prime-UTR | SLC25A21 | SLC25A21 |  | Y |
| IM2 | somaticSNP | 14 | 70245100 | A | C | missense | SLC10A1 | SLC10A1 |  | Y |
| IM2 | somaticSNP | 14 | 70826226 | C | G | intron | SYNJ2BP-COX16,COX16 |  |  | Y |
| IM2 | somaticSNP | 14 | 72926278 | G | A | intron | RGS6 |  |  | Y |
| IM2 | somaticSNP | 14 | 104199580 | A | C | 3-prime-UTR | ZFYVE21 | ZFYVE21 |  | Y |
| IM2 | somaticSNP | 14 | 105613850 | T | A | missense | JAG2 | JAG2 |  | Y |
| IM2 | somaticSNP | 15 | 23891689 | C | T | missense | MAGEL2 | MAGEL2 |  | Y |
| IM2 | somaticSNP | 15 | 25449634 | A | T | upstream-gene |  | |  | N |
| IM2 | somaticSNP | 15 | 33893830 | T | A | intron | RYR3 |  |  | Y |
| IM2 | somaticSNP | 15 | 40846225 | G | A | intron,missense | C15orf57 | C15orf57 |  | Y |
| IM2 | somaticSNP | 15 | 43528016 | C | G | missense | TGM5 | TGM5 |  | Y |
| IM2 | somaticSNP | 15 | 43889786 | T | C | intron | RNU6-28P,CKMT1B |  |  | Y |
| IM2 | somaticSNP | 15 | 48818257 | T | A | intron | FBN1 |  |  | Y |
| IM2 | somaticSNP | 15 | 79585665 | C | A | missense | ANKRD34C | ANKRD34C |  | Y |
| IM2 | somaticINDEL | 15 | 83241984 | A | -T | intron | CPEB1 |  |  | Y |
| IM2 | somaticSNP | 15 | 90029474 | C | T | intron | RHCG |  |  | N |
| IM2 | somaticSNP | 16 | 332316 | G | A | synonymous | ARHGDIG | ARHGDIG |  | N |
| IM2 | somaticSNP | 16 | 842643 | A | T | intron | CHTF18 |  |  | Y |
| IM2 | somaticSNP | 16 | 2821211 | T | C | 3-prime-UTR | SRRM2 | SRRM2 |  | Y |
| IM2 | somaticSNP | 16 | 11556121 | A | G | synonymous | LOC400499 |  |  | N |
| IM2 | somaticSNP | 16 | 15808975 | C | T | intron | NDE1,MYH11 |  | MYH11 | Y |
| IM2 | somaticINDEL | 16 | 23593886 | A | +TGTT | intron | NDUFAB1 |  |  | N |
| IM2 | somaticSNP | 16 | 30380445 | A | T | intron | TBC1D10B |  |  | Y |
| IM2 | somaticSNP | 16 | 31374294 | C | T | synonymous | ITGAX | ITGAX |  | Y |
| IM2 | somaticSNP | 16 | 46540233 | A | G | intron | ANKRD26P1 |  |  | N |
| IM2 | somaticSNP | 16 | 55617059 | T | A | 3-prime-UTR | LPCAT2 | LPCAT2 |  | Y |
| IM2 | somaticSNP | 16 | 57168812 | C | A | intron | CPNE2 |  |  | Y |
| IM2 | somaticSNP | 16 | 58568227 | C | T | missense,non-coding-exon | CNOT1 | CNOT1 |  | Y |
| IM2 | somaticSNP | 16 | 71969447 | T | C | intron | PKD1L3 |  |  | Y |
| IM2 | somaticSNP | 16 | 74382863 | T | C | non-coding-exon | LOC283922 | LOC283922 |  | Y |
| IM2 | somaticSNP | 17 | 4719892 | G | T | intron | PLD2 |  |  | N |
| IM2 | somaticSNP | 17 | 8076698 | G | C | 3-prime-UTR | TMEM107 | TMEM107 |  | Y |
| IM2 | somaticINDEL | 17 | 9142981 | C | -G | nonsynonymous,del,frameshift | NTN1 | NTN1 |  | N |
| IM2 | somaticSNP | 17 | 11671943 | G | T | missense-near-splice | DNAH9 | DNAH9 |  | Y |
| IM2 | somaticSNP | 17 | 12852477 | C | T | synonymous | ARHGAP44 | ARHGAP44 |  | N |
| IM2 | somaticSNP | 17 | 16040527 | T | C | intron | NCOR1 |  |  | N |
| IM2 | somaticSNP | 17 | 21207939 | G | T | intron | MAP2K3 |  |  | Y |
| IM2 | somaticSNP | 17 | 27822745 | G | T | missense-near-splice | TAOK1 | TAOK1 |  | Y |
| IM2 | somaticSNP | 17 | 28616359 | A | G | intron | BLMH |  |  | Y |
| IM2 | somaticSNP | 17 | 29161991 | G | A | missense | ATAD5 | ATAD5 |  | Y |
| IM2 | somaticSNP | 17 | 29226536 | G | T | missense | TEFM | TEFM |  | N |
| IM2 | somaticSNP | 17 | 38910804 | T | C | intron | KRT25 |  |  | Y |
| IM2 | somaticSNP | 17 | 42335782 | T | C | synonymous-near-splice | SLC4A1 | SLC4A1 |  | N |
| IM2 | somaticSNP | 17 | 56429045 | C | A | intron | SUPT4H1,BZRAP1-AS1 |  |  | N |
| IM2 | somaticINDEL | 17 | 56693799 | C | +T | intron | TEX14 |  |  | Y |
| IM2 | somaticSNP | 17 | 57228448 | C | T | intron | SKA2 |  |  | N |
| IM2 | somaticSNP | 17 | 57886199 | C | A | missense | VMP1 | VMP1 |  | Y |
| IM2 | somaticSNP | 17 | 58326937 | C | T | intron | USP32 |  |  | N |
| IM2 | somaticSNP | 17 | 71223299 | C | A | intron-near-splice | FAM104A |  |  | Y |
| IM2 | somaticSNP | 17 | 74079838 | A | G | intron-near-splice | EXOC7 |  |  | Y |
| IM2 | somaticSNP | 17 | 75834846 | C | T | intergenic |  | |  | Y |
| IM2 | somaticSNP | 17 | 80129776 | T | C | intron | CCDC57 |  |  | Y |
| IM2 | somaticSNP | 18 | 12067950 | C | T | intergenic |  | |  | Y |
| IM2 | somaticSNP | 18 | 12103292 | T | G | intron | ANKRD62 |  |  | N |
| IM2 | somaticSNP | 18 | 74620187 | A | T | intron | ZNF236 |  |  | Y |
| IM2 | somaticSNP | 19 | 7706661 | G | A | missense | STXBP2 | STXBP2 |  | Y |
| IM2 | somaticSNP | 19 | 9007560 | A | G | intron-near-splice | MUC16 |  |  | Y |
| IM2 | somaticSNP | 19 | 10266472 | G | T | intron | DNMT1 |  |  | N |
| IM2 | somaticSNP | 19 | 15276007 | C | A | intron | NOTCH3 |  |  | Y |
| IM2 | somaticSNP | 19 | 33359482 | C | T | 5-prime-UTR,intron | SLC7A9 | SLC7A9 |  | N |
| IM2 | somaticSNP | 19 | 34663663 | C | T | missense | LSM14A | LSM14A |  | Y |
| IM2 | somaticINDEL | 19 | 35832910 | G | -TCCT | intron | CD22 |  |  | Y |
| IM2 | somaticSNP | 19 | 37368933 | A | T | stop-gained,intron | ZNF345 | ZNF345 |  | Y |
| IM2 | somaticSNP | 19 | 38692623 | C | T | synonymous | SIPA1L3 | SIPA1L3 |  | Y |
| IM2 | somaticSNP | 19 | 41916759 | G | A | intron | BCKDHA |  |  | Y |
| IM2 | somaticSNP | 19 | 42814990 | G | A | upstream-gene |  | |  | Y |
| IM2 | somaticSNP | 19 | 45147340 | G | T | 5-prime-UTR | PVR | PVR |  | N |
| IM2 | somaticSNP | 19 | 46811328 | T | C | intron | HIF3A |  |  | Y |
| IM2 | somaticSNP | 19 | 47763712 | G | C | intron | CCDC9 |  |  | Y |
| IM2 | somaticSNP | 19 | 49303120 | C | A | intron | BCAT2 |  |  | Y |
| IM2 | somaticSNP | 19 | 50250504 | C | A | 5-prime-UTR,intron | TSKS |  |  | Y |
| IM2 | somaticSNP | 19 | 50266498 | T | A | missense | TSKS | TSKS |  | Y |
| IM2 | somaticSNP | 19 | 50463727 | G | A | intron | SIGLEC11 |  |  | Y |
| IM2 | somaticSNP | 2 | 26204388 | G | A | synonymous | KIF3C | KIF3C |  | Y |
| IM2 | somaticSNP | 2 | 29379290 | A | C | missense | CLIP4 | CLIP4 |  | Y |
| IM2 | somaticSNP | 2 | 31412096 | T | C | intron | CAPN14 |  |  | Y |
| IM2 | somaticSNP | 2 | 31483585 | T | C | missense | EHD3 | EHD3 |  | Y |
| IM2 | somaticSNP | 2 | 54891911 | A | T | intron | SPTBN1 |  |  | N |
| IM2 | somaticSNP | 2 | 55433542 | T | C | intron | CLHC1 |  |  | N |
| IM2 | somaticSNP | 2 | 86422665 | A | G | 5-prime-UTR | IMMT | IMMT |  | N |
| IM2 | somaticSNP | 2 | 98833151 | T | G | intron | VWA3B |  |  | Y |
| IM2 | somaticSNP | 2 | 116447348 | A | G | intron | DPP10 |  |  | Y |
| IM2 | somaticSNP | 2 | 118701541 | T | C | intron | CCDC93 |  |  | Y |
| IM2 | somaticSNP | 2 | 128522167 | G | A | intron,synonymous | WDR33 | WDR33 |  | Y |
| IM2 | somaticSNP | 2 | 151331371 | T | C | intron | RND3 |  |  | Y |
| IM2 | somaticSNP | 2 | 153551068 | C | T | synonymous | PRPF40A | PRPF40A |  | Y |
| IM2 | somaticSNP | 2 | 153574008 | C | A | 5-prime-UTR,missense | PRPF40A | PRPF40A |  | Y |
| IM2 | somaticSNP | 2 | 166908325 | G | T | missense | SCN1A | SCN1A |  | Y |
| IM2 | somaticSNP | 2 | 176857148 | T | A | splice-3 | KIAA1715 |  |  | Y |
| IM2 | somaticSNP | 2 | 177034400 | G | A | intron | HOXD3 |  |  | Y |
| IM2 | somaticINDEL | 2 | 197643854 | C | -T | intron | GTF3C3 |  |  | N |
| IM2 | somaticSNP | 2 | 201756675 | A | G | intron,synonymous | NIF3L1 | NIF3L1 |  | Y |
| IM2 | somaticSNP | 2 | 210877639 | A | T | intron | RPE |  |  | Y |
| IM2 | somaticSNP | 2 | 220358383 | G | T | upstream-gene |  | |  | Y |
| IM2 | somaticSNP | 2 | 228846479 | T | G | missense | SPHKAP | SPHKAP |  | Y |
| IM2 | somaticSNP | 2 | 231152571 | A | T | intron | SP140 |  |  | Y |
| IM2 | somaticSNP | 2 | 236708207 | A | G | intron | AGAP1 |  |  | Y |
| IM2 | somaticSNP | 2 | 242800900 | G | A | intron | PDCD1 |  |  | Y |
| IM2 | somaticSNP | 20 | 3219894 | A | G | intron | SLC4A11 |  |  | N |
| IM2 | somaticSNP | 20 | 6060118 | T | C | missense | FERMT1 | FERMT1 |  | N |
| IM2 | somaticSNP | 20 | 31887621 | A | T | intron | BPIFB1 |  |  | Y |
| IM2 | somaticSNP | 20 | 32868834 | T | A | 3-prime-UTR | AHCY | AHCY |  | Y |
| IM2 | somaticSNP | 20 | 34204981 | C | T | intron | SPAG4 |  |  | Y |
| IM2 | somaticSNP | 20 | 37529300 | G | A | missense | PPP1R16B | PPP1R16B |  | Y |
| IM2 | somaticINDEL | 20 | 43249004 | A | -TCT | nonsynonymous,del,frameshift | ADA | ADA |  | N |
| IM2 | somaticINDEL | 20 | 46281139 | A | -T | intron | NCOA3 |  |  | N |
| IM2 | somaticSNP | 20 | 49202537 | G | C | downstream-gene |  | |  | N |
| IM2 | somaticSNP | 20 | 49225000 | C | A | synonymous | FAM65C | FAM65C |  | Y |
| IM2 | somaticSNP | 20 | 55803351 | C | T | missense | BMP7 | BMP7 |  | Y |
| IM2 | somaticSNP | 20 | 55908207 | T | C | intron | SPO11 |  |  | Y |
| IM2 | somaticSNP | 20 | 58425592 | T | C | downstream-gene |  | |  | Y |
| IM2 | somaticSNP | 21 | 16340367 | C | A | missense | NRIP1 | NRIP1 |  | Y |
| IM2 | somaticSNP | 21 | 19642346 | G | A | synonymous | TMPRSS15 | TMPRSS15 |  | Y |
| IM2 | somaticSNP | 21 | 31798199 | G | C | missense | KRTAP13-3 | KRTAP13-3 |  | Y |
| IM2 | somaticSNP | 21 | 33039493 | A | C | intron | SOD1 |  |  | Y |
| IM2 | somaticINDEL | 21 | 35186127 | C | -T | intron | ITSN1 |  |  | N |
| IM2 | somaticSNP | 21 | 41719719 | G | T | missense,non-coding-exon | DSCAM | DSCAM |  | Y |
| IM2 | somaticSNP | 21 | 43541206 | A | C | missense | UMODL1 | UMODL1 |  | Y |
| IM2 | somaticSNP | 21 | 44478157 | C | G | intron | CBS |  |  | Y |
| IM2 | somaticSNP | 22 | 23041026 | G | A | intergenic |  | |  | Y |
| IM2 | somaticSNP | 22 | 25603127 | G | A | missense | CRYBB3 | CRYBB3 |  | Y |
| IM2 | somaticSNP | 22 | 31335601 | C | A | intron-near-splice | MORC2 |  |  | Y |
| IM2 | somaticSNP | 22 | 36690459 | T | C | intron | MYH9 |  | MYH9 | N |
| IM2 | somaticSNP | 22 | 38622957 | C | A | intron | TMEM184B |  |  | N |
| IM2 | somaticSNP | 22 | 40661645 | G | T | intron,missense,missense | TNRC6B | TNRC6B |  | Y |
| IM2 | somaticINDEL | 22 | 42289405 | C | +GTTT | intron | SREBF2 |  |  | Y |
| IM2 | somaticSNP | 22 | 42415519 | A | G | intron | WBP2NL |  |  | Y |
| IM2 | somaticSNP | 22 | 44594723 | T | C | intron | PARVG |  |  | Y |
| IM2 | somaticSNP | 22 | 50469385 | G | A | intron | TTLL8 |  |  | N |
| IM2 | somaticSNP | 3 | 403347 | A | G | intron | CHL1 |  |  | Y |
| IM2 | somaticINDEL | 3 | 19389266 | C | +A | nonsynonymous,ins,frameshift | KCNH8 | KCNH8 |  | N |
| IM2 | somaticSNP | 3 | 38051009 | C | T | intron | PLCD1 |  |  | Y |
| IM2 | somaticSNP | 3 | 39448229 | A | G | 5-prime-UTR | RPSA | RPSA |  | N |
| IM2 | somaticSNP | 3 | 44283610 | T | A | missense | TOPAZ1 | TOPAZ1 |  | Y |
| IM2 | somaticSNP | 3 | 49311526 | T | A | missense | C3orf62 | C3orf62 |  | N |
| IM2 | somaticINDEL | 3 | 57108374 | T | -C | nonsynonymous,del,frameshift | SPATA12,ARHGEF3 | SPATA12 |  | N |
| IM2 | somaticSNP | 3 | 57447331 | C | A | missense | DNAH12 | DNAH12 |  | Y |
| IM2 | somaticSNP | 3 | 69112049 | C | T | intron | UBA3 |  |  | N |
| IM2 | somaticSNP | 3 | 97464291 | T | A | intron | EPHA6 |  |  | Y |
| IM2 | somaticSNP | 3 | 100084408 | T | C | synonymous | TOMM70A | TOMM70A |  | Y |
| IM2 | somaticSNP | 3 | 121132268 | T | A | intron | STXBP5L |  |  | Y |
| IM2 | somaticSNP | 3 | 124449461 | G | A | 5-prime-UTR,missense,non-coding-exon | UMPS | UMPS |  | Y |
| IM2 | somaticSNP | 3 | 133877257 | C | T | intron | RYK |  |  | Y |
| IM2 | somaticSNP | 3 | 142840069 | T | A | synonymous | CHST2 | CHST2 |  | N |
| IM2 | somaticSNP | 3 | 160149944 | C | T | intron | SMC4 |  |  | Y |
| IM2 | somaticSNP | 3 | 167413491 | T | G | synonymous | PDCD10 | PDCD10 |  | Y |
| IM2 | somaticINDEL | 3 | 171065010 | G | +A | intron | TNIK |  |  | Y |
| IM2 | somaticSNP | 3 | 184299095 | A | C | synonymous | EPHB3 | EPHB3 |  | Y |
| IM2 | somaticSNP | 3 | 185213018 | T | G | missense | TMEM41A | TMEM41A |  | Y |
| IM2 | somaticSNP | 3 | 185879581 | T | A | intron | DGKG |  |  | Y |
| IM2 | somaticSNP | 3 | 194118705 | G | A | synonymous | GP5 | GP5 |  | Y |
| IM2 | somaticSNP | 3 | 195517843 | G | A | intron,missense | MUC4 | MUC4 |  | Y |
| IM2 | somaticSNP | 4 | 2900240 | G | A | synonymous | ADD1 | ADD1 |  | Y |
| IM2 | somaticSNP | 4 | 10020564 | G | A | intron | SLC2A9 |  |  | N |
| IM2 | somaticINDEL | 4 | 26241366 | G | +GGAGA | intron | RBPJ |  |  | N |
| IM2 | somaticSNP | 4 | 48546969 | T | G | intron | FRYL |  |  | Y |
| IM2 | somaticSNP | 4 | 73164017 | T | A | missense | ADAMTS3 | ADAMTS3 |  | N |
| IM2 | somaticSNP | 4 | 90034354 | G | C | missense | TIGD2 | TIGD2 |  | Y |
| IM2 | somaticSNP | 4 | 103556316 | T | C | intron | MANBA |  |  | Y |
| IM2 | somaticINDEL | 4 | 106317370 | T | +ATAAA | intron | PPA2 |  |  | N |
| IM2 | somaticSNP | 4 | 122068167 | T | C | intron | TNIP3 |  |  | Y |
| IM2 | somaticSNP | 4 | 125631753 | T | C | 5-prime-UTR,intron | ANKRD50 | ANKRD50 |  | Y |
| IM2 | somaticSNP | 4 | 126240096 | G | A | missense | FAT4 | FAT4 |  | Y |
| IM2 | somaticSNP | 4 | 155507484 | T | A | missense | FGA | FGA |  | Y |
| IM2 | somaticSNP | 4 | 155533047 | A | G | missense | FGG | FGG |  | N |
| IM2 | somaticSNP | 5 | 7414718 | A | G | synonymous | ADCY2 | ADCY2 |  | Y |
| IM2 | somaticSNP | 5 | 16701535 | T | G | missense | MYO10 | MYO10 |  | Y |
| IM2 | somaticSNP | 5 | 37168957 | A | G | missense | C5orf42 | C5orf42 |  | Y |
| IM2 | somaticINDEL | 5 | 78416973 | C | -A | intron | BHMT |  |  | N |
| IM2 | somaticSNP | 5 | 101813531 | G | C | missense | SLCO6A1 | SLCO6A1 |  | Y |
| IM2 | somaticSNP | 5 | 132052648 | T | C | synonymous | KIF3A | KIF3A |  | Y |
| IM2 | somaticSNP | 5 | 135396468 | T | A | intron | TGFBI |  |  | Y |
| IM2 | somaticSNP | 5 | 141059904 | G | T | missense | ARAP3 | ARAP3 |  | Y |
| IM2 | somaticSNP | 5 | 145531578 | G | A | intron | LARS |  |  | Y |
| IM2 | somaticSNP | 5 | 149112300 | T | G | intron | PPARGC1B |  |  | Y |
| IM2 | somaticSNP | 5 | 154396804 | T | A | missense | KIF4B | KIF4B |  | Y |
| IM2 | somaticSNP | 5 | 169309494 | T | A | intron | DOCK2,FAM196B |  |  | Y |
| IM2 | somaticSNP | 5 | 169412889 | C | T | missense | DOCK2 | DOCK2 |  | Y |
| IM2 | somaticSNP | 5 | 172110938 | G | A | intron,missense | NEURL1B | NEURL1B |  | Y |
| IM2 | somaticSNP | 6 | 5000779 | G | A | intron | RPP40 |  |  | Y |
| IM2 | somaticSNP | 6 | 11104238 | C | A | stop-gained,intron | SMIM13,ERVFRD-1 | ERVFRD-1 |  | Y |
| IM2 | somaticSNP | 6 | 33653560 | C | A | missense | ITPR3 | ITPR3 |  | Y |
| IM2 | somaticSNP | 6 | 34029881 | C | T | intron | GRM4 |  |  | Y |
| IM2 | somaticSNP | 6 | 71233675 | A | G | missense | FAM135A | FAM135A |  | Y |
| IM2 | somaticSNP | 6 | 75801024 | G | T | intron | COL12A1 |  |  | Y |
| IM2 | somaticSNP | 6 | 99848727 | T | C | missense | PNISR | PNISR |  | Y |
| IM2 | somaticSNP | 6 | 108831414 | G | A | splice-acceptor | LACE1 | LACE1 |  | N |
| IM2 | somaticSNP | 6 | 116441709 | C | G | missense,intron | COL10A1,NT5DC1 | COL10A1 |  | Y |
| IM2 | somaticSNP | 6 | 132874297 | T | A | missense | TAAR8 | TAAR8 |  | Y |
| IM2 | somaticSNP | 6 | 144508629 | C | A | 3-prime-UTR | STX11 | STX11 |  | Y |
| IM2 | somaticSNP | 6 | 149771895 | T | A | missense | ZC3H12D | ZC3H12D |  | Y |
| IM2 | somaticSNP | 6 | 167426949 | A | G | intron | FGFR1OP |  | FGFR1OP | Y |
| IM2 | somaticSNP | 6 | 167446139 | A | G | intron | FGFR1OP |  | FGFR1OP | Y |
| IM2 | somaticSNP | 6 | 170034675 | A | T | intron | WDR27 |  |  | Y |
| IM2 | somaticSNP | 6 | 170049362 | C | A | missense | WDR27 | WDR27 |  | Y |
| IM2 | somaticSNP | 7 | 1914350 | A | G | intron | MAD1L1 |  |  | Y |
| IM2 | somaticSNP | 7 | 4304941 | G | T | intron,missense | SDK1 | SDK1 |  | Y |
| IM2 | somaticSNP | 7 | 12409830 | T | C | missense | VWDE | VWDE |  | Y |
| IM2 | somaticSNP | 7 | 12610475 | C | T | synonymous | SCIN | SCIN |  | Y |
| IM2 | somaticSNP | 7 | 16729336 | C | T | intron | BZW2 |  |  | Y |
| IM2 | somaticSNP | 7 | 19156335 | G | T | 3-prime-UTR | TWIST1 | TWIST1 |  | Y |
| IM2 | somaticINDEL | 7 | 22330862 | T | +A | intron | RAPGEF5 |  |  | N |
| IM2 | somaticINDEL | 7 | 23296415 | G | +A | intron | GPNMB |  |  | N |
| IM2 | somaticSNP | 7 | 44619235 | G | A | intron-near-splice | TMED4 |  |  | Y |
| IM2 | somaticSNP | 7 | 48146967 | G | A | missense | UPP1 | UPP1 |  | Y |
| IM2 | somaticSNP | 7 | 63726690 | C | A | missense | ZNF679 | ZNF679 |  | Y |
| IM2 | somaticSNP | 7 | 81631020 | T | A | intron | CACNA2D1 |  |  | Y |
| IM2 | somaticSNP | 7 | 99578411 | T | C | non-coding-exon | AZGP1P1 | AZGP1P1 |  | N |
| IM2 | somaticSNP | 7 | 107332509 | G | A | intron,missense | SLC26A4 |  |  | Y |
| IM2 | somaticSNP | 7 | 107599765 | G | A | synonymous | LAMB1 | LAMB1 |  | Y |
| IM2 | somaticINDEL | 7 | 116409675 | C | -T | intron | MET |  | MET | Y |
| IM2 | somaticSNP | 7 | 131982688 | C | A | intron | PLXNA4 |  |  | N |
| IM2 | somaticINDEL | 7 | 134930169 | A | +GAG | intron | STRA8 |  |  | N |
| IM2 | somaticSNP | 7 | 142045484 | C | A | intergenic |  | |  | N |
| IM2 | somaticSNP | 7 | 142561350 | G | T | intron | EPHB6 |  |  | Y |
| IM2 | somaticSNP | 7 | 143095395 | C | T | intron | EPHA1 |  |  | Y |
| IM2 | somaticSNP | 7 | 143573132 | C | T | intron,synonymous | FAM115A | FAM115A |  | Y |
| IM2 | somaticSNP | 7 | 149525600 | G | T | intron | SSPO |  |  | N |
| IM2 | somaticSNP | 8 | 28821353 | T | C | 5-prime-UTR | HMBOX1 | HMBOX1 |  | Y |
| IM2 | somaticSNP | 8 | 35579196 | T | C | intron | UNC5D |  |  | Y |
| IM2 | somaticSNP | 8 | 39103668 | C | G | missense | ADAM32 | ADAM32 |  | Y |
| IM2 | somaticSNP | 8 | 69351923 | A | C | intron | C8orf34 |  |  | Y |
| IM2 | somaticSNP | 8 | 73150337 | A | T | non-coding-exon | LOC392232 | LOC392232 |  | N |
| IM2 | somaticSNP | 8 | 73479912 | T | A | 5-prime-UTR | KCNB2 | KCNB2 |  | N |
| IM2 | somaticSNP | 8 | 113418760 | A | T | synonymous | CSMD3 | CSMD3 |  | Y |
| IM2 | somaticSNP | 8 | 125047646 | C | T | intron,synonymous | FER1L6,FER1L6-AS1 | FER1L6 |  | N |
| IM2 | somaticSNP | 8 | 131964000 | C | A | intron | ADCY8 |  |  | Y |
| IM2 | somaticSNP | 8 | 141712834 | T | C | intron | PTK2 |  |  | Y |
| IM2 | somaticSNP | 8 | 143614849 | G | T | intron | BAI1 |  |  | Y |
| IM2 | somaticSNP | 8 | 146220171 | T | C | intron | ZNF252P |  |  | Y |
| IM2 | somaticSNP | 9 | 84202674 | C | G | synonymous | TLE1 | TLE1 |  | Y |
| IM2 | somaticSNP | 9 | 86895009 | G | T | intron | SLC28A3 |  |  | Y |
| IM2 | somaticSNP | 9 | 98766997 | G | T | intron | ERCC6L2 |  |  | Y |
| IM2 | somaticSNP | 9 | 99314276 | G | C | intron | CDC14B |  |  | Y |
| IM2 | somaticSNP | 9 | 105763940 | T | C | intron | CYLC2 |  |  | Y |
| IM2 | somaticSNP | 9 | 111693233 | C | A | intron | IKBKAP |  |  | Y |
| IM2 | somaticSNP | 9 | 112172661 | G | C | missense,non-coding-exon | PTPN3 | PTPN3 |  | Y |
| IM2 | somaticSNP | 9 | 113341468 | G | T | missense | SVEP1 | SVEP1 |  | Y |
| IM2 | somaticSNP | 9 | 130860849 | C | T | intron,missense,non-coding-exon | SLC25A25 | SLC25A25 |  | Y |
| IM2 | somaticSNP | 9 | 131223392 | C | A | intron | ODF2 |  |  | Y |
| IM2 | somaticSNP | 9 | 133554034 | G | C | intron-near-splice | PRDM12 |  |  | Y |
| IM2 | somaticSNP | 9 | 139396088 | C | A | intron | NOTCH1 |  | NOTCH1 | N |
| IM2 | somaticSNP | 9 | 139904603 | T | A | intron | ABCA2 |  |  | Y |
| IM2 | somaticSNP | X | 35985729 | A | G | intron | CXorf22 |  |  | Y |
| IM2 | somaticSNP | X | 50053087 | T | A | intron,missense | CCNB3 | CCNB3 |  | Y |
| IM2 | somaticSNP | X | 53250052 | G | A | 5-prime-UTR,intron,missense | KDM5C | KDM5C | KDM5C | Y |
| IM2 | somaticSNP | X | 117815120 | A | G | missense | DOCK11 | DOCK11 |  | Y |
| IM2 | somaticSNP | X | 125686072 | G | T | missense | DCAF12L1 | DCAF12L1 |  | Y |
| IM2 | somaticSNP | X | 153236100 | C | T | synonymous-near-splice | HCFC1 | HCFC1 |  | Y |
| TIS | somaticSNP | 7 | 151970859 | C | T | missense | KMT2C | KMT2C | KMT2C | Y |
| TIS | somaticSNP | 1 | 17023483 | C | T | intron | ESPNP |  |  | N |
| TIS | somaticSNP | 1 | 17267496 | C | T | intron | CROCC |  |  | N |
| TIS | somaticINDEL | 1 | 24294450 | C | +A | intron | SRSF10 |  |  | N |
| TIS | somaticSNP | 1 | 26873700 | C | T | 5-prime-UTR,synonymous | RPS6KA1 | RPS6KA1 |  | N |
| TIS | somaticSNP | 1 | 41327871 | C | A | 5-prime-UTR | CITED4 | CITED4 |  | N |
| TIS | somaticINDEL | 1 | 93159821 | C | -A | intron | EVI5 |  |  | N |
| TIS | somaticINDEL | 1 | 103471734 | A | +AAATAAATAAAT | intron | COL11A1 |  |  | N |
| TIS | somaticSNP | 1 | 145281824 | T | G | intron | NBPF20,NBPF9,NOTCH2NL |  |  | N |
| TIS | somaticSNP | 1 | 205034434 | G | T | intron | CNTN2 |  |  | Y |
| TIS | somaticSNP | 1 | 227227740 | A | C | intron | CDC42BPA |  |  | N |
| TIS | somaticSNP | 10 | 82331347 | A | G | intron | SH2D4B |  |  | Y |
| TIS | somaticINDEL | 10 | 95454768 | T | -A | intron | FRA10AC1 |  |  | N |
| TIS | somaticSNP | 11 | 101375549 | T | C | intron | TRPC6 |  |  | Y |
| TIS | somaticSNP | 12 | 21695584 | A | G | intron | GYS2 |  |  | N |
| TIS | somaticSNP | 12 | 29936449 | C | A | intron,missense | TMTC1 | TMTC1 |  | Y |
| TIS | somaticSNP | 12 | 53238112 | C | T | intron | KRT78 |  |  | N |
| TIS | somaticSNP | 13 | 99540395 | A | G | intron | DOCK9 |  |  | N |
| TIS | somaticSNP | 14 | 23733725 | C | T | intron | C14orf164 |  |  | Y |
| TIS | somaticSNP | 14 | 104199580 | A | C | 3-prime-UTR | ZFYVE21 | ZFYVE21 |  | Y |
| TIS | somaticSNP | 16 | 11556121 | A | G | synonymous | LOC400499 |  |  | N |
| TIS | somaticINDEL | 17 | 10550845 | T | -TTTTG | intron | MYH3 |  |  | N |
| TIS | somaticINDEL | 17 | 56693799 | C | +T | intron | TEX14 |  |  | N |
| TIS | somaticSNP | 17 | 74079838 | A | G | intron-near-splice | EXOC7 |  |  | Y |
| TIS | somaticSNP | 19 | 15276007 | C | A | intron | NOTCH3 |  |  | Y |
| TIS | somaticSNP | 19 | 45147340 | G | T | 5-prime-UTR | PVR | PVR |  | N |
| TIS | somaticSNP | 19 | 50463727 | G | A | intron | SIGLEC11 |  |  | N |
| TIS | somaticSNP | 2 | 31412096 | T | C | intron | CAPN14 |  |  | N |
| TIS | somaticSNP | 2 | 242800900 | G | A | intron | PDCD1 |  |  | Y |
| TIS | somaticSNP | 20 | 3219894 | A | G | intron | SLC4A11 |  |  | N |
| TIS | somaticINDEL | 21 | 19628809 | C | -T | intron | CHODL |  |  | N |
| TIS | somaticINDEL | 21 | 35186127 | C | -T | intron | ITSN1 |  |  | N |
| TIS | somaticSNP | 22 | 23041026 | G | A | intergenic |  | |  | Y |
| TIS | somaticSNP | 3 | 69112049 | C | T | intron | UBA3 |  |  | N |
| TIS | somaticSNP | 3 | 121132268 | T | A | intron | STXBP5L |  |  | N |
| TIS | somaticINDEL | 3 | 150282061 | C | -T | intron | EIF2A |  |  | N |
| TIS | somaticINDEL | 3 | 171065010 | G | +A | intron | TNIK |  |  | N |
| TIS | somaticSNP | 4 | 10020564 | G | A | intron | SLC2A9 |  |  | N |
| TIS | somaticINDEL | 4 | 26241366 | G | +GGAGA | intron | RBPJ |  |  | N |
| TIS | somaticSNP | 4 | 103556316 | T | C | intron | MANBA |  |  | Y |
| TIS | somaticINDEL | 4 | 106317370 | T | +ATAAA | intron | PPA2 |  |  | N |
| TIS | somaticSNP | 5 | 149112300 | T | G | intron | PPARGC1B |  |  | Y |
| TIS | somaticSNP | 6 | 5000779 | G | A | intron | RPP40 |  |  | Y |
| TIS | somaticSNP | 7 | 1914350 | A | G | intron | MAD1L1 |  |  | Y |
| TIS | somaticINDEL | 7 | 22330862 | T | +A | intron | RAPGEF5 |  |  | N |
| TIS | somaticSNP | 7 | 99578370 | T | G | upstream-gene |  | |  | N |
| TIS | somaticSNP | 7 | 99578381 | T | C | upstream-gene |  | |  | N |
| TIS | somaticINDEL | 7 | 116409675 | C | -T | intron | MET |  | MET | N |
| TIS | somaticSNP | 7 | 99578411 | T | C | non-coding-exon | AZGP1P1 | AZGP1P1 |  | Y |
| TIS | somaticSNP | X | 119760799 | C | T | missense | C1GALT1C1 | C1GALT1C1 |  | Y |
|  |  |  |  |  |  |  |  |  |  |  |
| Validation value=Y: the mutation was Sanger validated; value=N: the mutation was not Sanger validated; value=NA: not selected for Sanger validation. | | | | | | | | | | |
| GATK value=Y: the SNP/indel was detected by GATK; Otherwise, it was detected by the more sensitive method as described in the Method. | | | | | | | | | | |
|  |  |  |  |  |  |  |  |  |  |  |
