# Supplementary material for: Inferring the progression of multifocal liver cancer from spatial and temporal genomic heterogeneity
Source: Oncotarget. 2015 Dec 11;7(3):2867–77. doi: 10.18632/oncotarget.6558 (PMC4823077; doi:10.18632/oncotarget.6558)
Supplement: Supplementary file 5 [file oncotarget-07-2867-s005.docx]

| **Supplementary Table 4. Regions with copy number variations.** | | | | |  |
| --- | --- | --- | --- | --- | --- |
| **Sample** | **Chromosome** | **Start** | **End** | **Copy Number Allele1** | **Copy Number Allele2** |
| HCC-A1 | 1 | 879317 | 142540223 | 1 | 1 |
| HCC-A1 | 1 | 144013853 | 145530254 | 1 | 2 |
| HCC-A1 | 1 | 145535611 | 223954080 | 1 | 3 |
| HCC-A1 | 1 | 224318151 | 249150116 | 1 | 2 |
| HCC-A1 | 2 | 672976 | 242839363 | 1 | 1 |
| HCC-A1 | 3 | 239555 | 197566254 | 1 | 1 |
| HCC-A1 | 4 | 67846 | 190903950 | 1 | 1 |
| HCC-A1 | 5 | 163205 | 180632059 | 1 | 2 |
| HCC-A1 | 6 | 335251 | 170892848 | 1 | 1 |
| HCC-A1 | 7 | 590148 | 158827326 | 1 | 1 |
| HCC-A1 | 8 | 182949 | 43147940 | 0 | 1 |
| HCC-A1 | 8 | 48585975 | 146157339 | 1 | 4 |
| HCC-A1 | 9 | 214706 | 140938183 | 1 | 1 |
| HCC-A1 | 10 | 285481 | 135381592 | 1 | 2 |
| HCC-A1 | 11 | 193112 | 134226278 | 1 | 1 |
| HCC-A1 | 12 | 235143 | 133683020 | 1 | 1 |
| HCC-A1 | 13 | 19756002 | 115091399 | 1 | 1 |
| HCC-A1 | 14 | 20201972 | 39512122 | 1 | 2 |
| HCC-A1 | 14 | 39517925 | 107282988 | 0 | 2 |
| HCC-A1 | 15 | 20453896 | 102192014 | 1 | 1 |
| HCC-A1 | 16 | 97610 | 11544741 | 0 | 1 |
| HCC-A1 | 16 | 11553883 | 33965644 | 1 | 1 |
| HCC-A1 | 16 | 34490107 | 90095620 | 0 | 1 |
| HCC-A1 | 17 | 6115 | 81006387 | 1 | 1 |
| HCC-A1 | 18 | 480801 | 77921406 | 1 | 1 |
| HCC-A1 | 19 | 311825 | 59059493 | 1 | 2 |
| HCC-A1 | 20 | 76962 | 31074372 | 1 | 1 |
| HCC-A1 | 20 | 31196471 | 62854566 | 1 | 2 |
| HCC-A1 | 21 | 9825966 | 48078611 | 1 | 1 |
| HCC-A1 | 22 | 17449076 | 51219006 | 1 | 1 |
| HCC-A2 | 1 | 879317 | 144013853 | 1 | 1 |
| HCC-A2 | 1 | 144013865 | 145282093 | 1 | 2 |
| HCC-A2 | 1 | 145498665 | 223936864 | 1 | 3 |
| HCC-A2 | 1 | 223951841 | 249150116 | 1 | 2 |
| HCC-A2 | 2 | 672976 | 242839363 | 1 | 1 |
| HCC-A2 | 3 | 239555 | 197566254 | 1 | 1 |
| HCC-A2 | 4 | 67846 | 190903950 | 1 | 1 |
| HCC-A2 | 5 | 163205 | 180632059 | 1 | 2 |
| HCC-A2 | 6 | 335251 | 170892848 | 1 | 1 |
| HCC-A2 | 7 | 590148 | 158827326 | 1 | 1 |
| HCC-A2 | 8 | 182949 | 43147940 | 0 | 1 |
| HCC-A2 | 8 | 48585975 | 146157339 | 1 | 4 |
| HCC-A2 | 9 | 214706 | 140938183 | 1 | 1 |
| HCC-A2 | 10 | 285481 | 135381592 | 1 | 2 |
| HCC-A2 | 11 | 193112 | 48367474 | 1 | 1 |
| HCC-A2 | 11 | 48373748 | 56468452 | 1 | 1 |
| HCC-A2 | 11 | 56468493 | 134226278 | 1 | 1 |
| HCC-A2 | 12 | 235143 | 133683020 | 1 | 1 |
| HCC-A2 | 13 | 19756002 | 115091399 | 1 | 1 |
| HCC-A2 | 14 | 20201972 | 38276433 | 1 | 2 |
| HCC-A2 | 14 | 39512122 | 107282988 | 0 | 2 |
| HCC-A2 | 15 | 20453896 | 102192014 | 1 | 1 |
| HCC-A2 | 16 | 97610 | 90095620 | 1 | 1 |
| HCC-A2 | 17 | 6115 | 81006387 | 1 | 1 |
| HCC-A2 | 18 | 480801 | 77921406 | 1 | 1 |
| HCC-A2 | 19 | 311825 | 59059493 | 1 | 2 |
| HCC-A2 | 20 | 76962 | 31074372 | 1 | 1 |
| HCC-A2 | 20 | 31196471 | 62854566 | 1 | 2 |
| HCC-A2 | 21 | 9825966 | 48078611 | 1 | 1 |
| HCC-A2 | 22 | 17449076 | 51219006 | 1 | 1 |
| HCC-A3 | 1 | 879317 | 142540223 | 1 | 1 |
| HCC-A3 | 1 | 144013853 | 145282093 | 1 | 2 |
| HCC-A3 | 1 | 145498665 | 223954080 | 1 | 3 |
| HCC-A3 | 1 | 224318151 | 249150116 | 1 | 2 |
| HCC-A3 | 2 | 672976 | 242839363 | 1 | 1 |
| HCC-A3 | 3 | 239555 | 111853895 | 1 | 1 |
| HCC-A3 | 3 | 112066562 | 112299732 | 0 | 1 |
| HCC-A3 | 3 | 112642568 | 197566254 | 1 | 1 |
| HCC-A3 | 4 | 67846 | 190903950 | 1 | 1 |
| HCC-A3 | 5 | 163205 | 180632059 | 1 | 2 |
| HCC-A3 | 6 | 335251 | 170892848 | 1 | 1 |
| HCC-A3 | 7 | 590148 | 100549593 | 1 | 1 |
| HCC-A3 | 7 | 100549650 | 100635205 | 1 | 1 |
| HCC-A3 | 7 | 100647875 | 158827326 | 1 | 1 |
| HCC-A3 | 8 | 182949 | 43147940 | 0 | 1 |
| HCC-A3 | 8 | 48585975 | 146157339 | 1 | 4 |
| HCC-A3 | 9 | 214706 | 140938183 | 1 | 1 |
| HCC-A3 | 10 | 285481 | 135381592 | 1 | 2 |
| HCC-A3 | 11 | 193112 | 134226278 | 1 | 1 |
| HCC-A3 | 12 | 235143 | 133683020 | 1 | 1 |
| HCC-A3 | 13 | 19756002 | 115091399 | 1 | 1 |
| HCC-A3 | 14 | 20201972 | 39517925 | 1 | 2 |
| HCC-A3 | 14 | 39532492 | 107170005 | 0 | 2 |
| HCC-A3 | 14 | 107282859 | 107282988 | 1 | 1 |
| HCC-A3 | 15 | 20453896 | 102192014 | 1 | 1 |
| HCC-A3 | 16 | 97610 | 90095620 | 1 | 1 |
| HCC-A3 | 17 | 6115 | 81006387 | 1 | 1 |
| HCC-A3 | 18 | 480801 | 77921406 | 1 | 1 |
| HCC-A3 | 19 | 311825 | 59059493 | 1 | 2 |
| HCC-A3 | 20 | 76962 | 30527065 | 1 | 1 |
| HCC-A3 | 20 | 31074372 | 62854566 | 1 | 2 |
| HCC-A3 | 21 | 9825966 | 48078611 | 1 | 1 |
| HCC-A3 | 22 | 17449076 | 51219006 | 1 | 1 |
| HCC-B1 | 1 | 879317 | 142540223 | 1 | 1 |
| HCC-B1 | 1 | 144013853 | 145282093 | 1 | 1 |
| HCC-B1 | 1 | 145498665 | 249150116 | 1 | 2 |
| HCC-B1 | 2 | 672976 | 242839363 | 1 | 1 |
| HCC-B1 | 3 | 239555 | 197566254 | 1 | 1 |
| HCC-B1 | 4 | 67846 | 190903950 | 1 | 1 |
| HCC-B1 | 5 | 163205 | 180632059 | 1 | 1 |
| HCC-B1 | 6 | 335251 | 170892848 | 1 | 1 |
| HCC-B1 | 7 | 590148 | 158827326 | 1 | 1 |
| HCC-B1 | 8 | 182949 | 146157339 | 1 | 1 |
| HCC-B1 | 9 | 214706 | 140938183 | 1 | 1 |
| HCC-B1 | 10 | 285481 | 135381592 | 1 | 1 |
| HCC-B1 | 11 | 193112 | 103780610 | 1 | 1 |
| HCC-B1 | 11 | 104866461 | 134226278 | 1 | 1 |
| HCC-B1 | 12 | 235143 | 133683020 | 1 | 1 |
| HCC-B1 | 13 | 19756002 | 115091399 | 1 | 1 |
| HCC-B1 | 14 | 20201972 | 65250939 | 1 | 1 |
| HCC-B1 | 14 | 65419293 | 107282988 | 1 | 1 |
| HCC-B1 | 15 | 20453896 | 102192014 | 1 | 1 |
| HCC-B1 | 16 | 97610 | 33630336 | 1 | 1 |
| HCC-B1 | 16 | 33630533 | 90095620 | 1 | 1 |
| HCC-B1 | 17 | 6115 | 21320114 | 1 | 1 |
| HCC-B1 | 17 | 21730653 | 81006387 | 1 | 1 |
| HCC-B1 | 18 | 480801 | 77921406 | 1 | 1 |
| HCC-B1 | 19 | 311825 | 59059493 | 1 | 1 |
| HCC-B1 | 20 | 76962 | 62854566 | 1 | 1 |
| HCC-B1 | 21 | 9825966 | 48078611 | 1 | 1 |
| HCC-B1 | 22 | 17449076 | 51219006 | 1 | 1 |
| HCC-B2 | 1 | 879317 | 145498665 | 1 | 1 |
| HCC-B2 | 1 | 145507826 | 249150116 | 1 | 2 |
| HCC-B2 | 2 | 672976 | 242839363 | 1 | 1 |
| HCC-B2 | 3 | 239555 | 197566254 | 1 | 1 |
| HCC-B2 | 4 | 67846 | 190903950 | 1 | 1 |
| HCC-B2 | 5 | 163205 | 180632059 | 1 | 1 |
| HCC-B2 | 6 | 335251 | 170892848 | 1 | 1 |
| HCC-B2 | 7 | 590148 | 100488658 | 1 | 1 |
| HCC-B2 | 7 | 100549505 | 100675976 | 1 | 1 |
| HCC-B2 | 7 | 100731925 | 158827326 | 1 | 1 |
| HCC-B2 | 8 | 182949 | 98943446 | 1 | 1 |
| HCC-B2 | 8 | 98954108 | 146157339 | 1 | 1 |
| HCC-B2 | 9 | 214706 | 140938183 | 1 | 1 |
| HCC-B2 | 10 | 285481 | 135381592 | 1 | 1 |
| HCC-B2 | 11 | 193112 | 117077034 | 1 | 1 |
| HCC-B2 | 11 | 117094020 | 134226278 | 1 | 1 |
| HCC-B2 | 12 | 235143 | 133683020 | 1 | 1 |
| HCC-B2 | 13 | 19756002 | 115091399 | 1 | 1 |
| HCC-B2 | 14 | 20201972 | 63269317 | 1 | 1 |
| HCC-B2 | 14 | 63453773 | 107282988 | 1 | 1 |
| HCC-B2 | 15 | 20453896 | 102192014 | 1 | 1 |
| HCC-B2 | 16 | 97610 | 33630336 | 1 | 1 |
| HCC-B2 | 16 | 33630533 | 90095620 | 1 | 1 |
| HCC-B2 | 17 | 6115 | 21319860 | 1 | 1 |
| HCC-B2 | 17 | 21319868 | 81006387 | 1 | 1 |
| HCC-B2 | 18 | 480801 | 77921406 | 1 | 1 |
| HCC-B2 | 19 | 311825 | 59059493 | 1 | 1 |
| HCC-B2 | 20 | 76962 | 62854566 | 1 | 1 |
| HCC-B2 | 21 | 9825966 | 48078611 | 1 | 1 |
| HCC-B2 | 22 | 17449076 | 51219006 | 1 | 1 |
| HCC-B3 | 1 | 879317 | 145282093 | 1 | 1 |
| HCC-B3 | 1 | 145498665 | 249150116 | 1 | 2 |
| HCC-B3 | 2 | 672976 | 242839363 | 1 | 1 |
| HCC-B3 | 3 | 239555 | 197566254 | 1 | 1 |
| HCC-B3 | 4 | 67846 | 190903950 | 1 | 1 |
| HCC-B3 | 5 | 163205 | 180632059 | 1 | 1 |
| HCC-B3 | 6 | 335251 | 170892848 | 1 | 1 |
| HCC-B3 | 7 | 590148 | 158827326 | 1 | 1 |
| HCC-B3 | 8 | 182949 | 87517700 | 1 | 1 |
| HCC-B3 | 8 | 87520894 | 146157339 | 1 | 1 |
| HCC-B3 | 9 | 214706 | 140938183 | 1 | 1 |
| HCC-B3 | 10 | 285481 | 135381592 | 1 | 1 |
| HCC-B3 | 11 | 193112 | 103123999 | 1 | 1 |
| HCC-B3 | 11 | 103126296 | 134226278 | 1 | 1 |
| HCC-B3 | 12 | 235143 | 133683020 | 1 | 1 |
| HCC-B3 | 13 | 19756002 | 115091399 | 1 | 1 |
| HCC-B3 | 14 | 20201972 | 62213920 | 1 | 1 |
| HCC-B3 | 14 | 62463214 | 107282988 | 1 | 1 |
| HCC-B3 | 15 | 20453896 | 102192014 | 1 | 1 |
| HCC-B3 | 16 | 97610 | 52785194 | 1 | 1 |
| HCC-B3 | 16 | 53076131 | 90095620 | 1 | 1 |
| HCC-B3 | 17 | 6115 | 21319786 | 1 | 1 |
| HCC-B3 | 17 | 21319845 | 81006387 | 1 | 1 |
| HCC-B3 | 18 | 480801 | 77921406 | 1 | 1 |
| HCC-B3 | 19 | 311825 | 59059493 | 1 | 1 |
| HCC-B3 | 20 | 76962 | 29630695 | 1 | 1 |
| HCC-B3 | 20 | 29633927 | 29652294 | 1 | 1 |
| HCC-B3 | 20 | 29652303 | 62854566 | 1 | 1 |
| HCC-B3 | 21 | 9825966 | 48078611 | 1 | 1 |
| HCC-B3 | 22 | 17449076 | 51219006 | 1 | 1 |
| ICC-C1 | 1 | 879317 | 31215364 | 1 | 1 |
| ICC-C1 | 1 | 31346057 | 249150116 | 1 | 1 |
| ICC-C1 | 2 | 672976 | 242839363 | 1 | 1 |
| ICC-C1 | 3 | 239555 | 101136402 | 1 | 1 |
| ICC-C1 | 3 | 101219889 | 197566254 | 1 | 1 |
| ICC-C1 | 4 | 67846 | 190903950 | 1 | 1 |
| ICC-C1 | 5 | 163205 | 180632059 | 1 | 1 |
| ICC-C1 | 6 | 335251 | 75968720 | 1 | 1 |
| ICC-C1 | 6 | 76357593 | 170892848 | 1 | 1 |
| ICC-C1 | 7 | 590148 | 158827326 | 1 | 1 |
| ICC-C1 | 8 | 182949 | 71071695 | 1 | 1 |
| ICC-C1 | 8 | 71486568 | 146157339 | 1 | 1 |
| ICC-C1 | 9 | 214706 | 140938183 | 1 | 1 |
| ICC-C1 | 10 | 285481 | 135381592 | 1 | 1 |
| ICC-C1 | 11 | 193112 | 70281359 | 1 | 1 |
| ICC-C1 | 11 | 70281665 | 134226278 | 1 | 1 |
| ICC-C1 | 12 | 235143 | 93100366 | 1 | 1 |
| ICC-C1 | 12 | 93131203 | 133683020 | 1 | 1 |
| ICC-C1 | 13 | 19756002 | 115091399 | 1 | 1 |
| ICC-C1 | 14 | 20201972 | 107282988 | 1 | 1 |
| ICC-C1 | 15 | 20453896 | 102192014 | 1 | 1 |
| ICC-C1 | 16 | 97610 | 90095620 | 1 | 1 |
| ICC-C1 | 17 | 6115 | 81006387 | 1 | 1 |
| ICC-C1 | 18 | 480801 | 77921406 | 1 | 1 |
| ICC-C1 | 19 | 311825 | 59059493 | 1 | 1 |
| ICC-C1 | 20 | 76962 | 62854566 | 1 | 1 |
| ICC-C1 | 21 | 9825966 | 48078611 | 1 | 1 |
| ICC-C1 | 22 | 17449076 | 51219006 | 1 | 1 |
| ICC-C2 | 1 | 879317 | 28555579 | 1 | 1 |
| ICC-C2 | 1 | 28559574 | 249150116 | 1 | 1 |
| ICC-C2 | 2 | 672976 | 242839363 | 1 | 1 |
| ICC-C2 | 3 | 239555 | 75786910 | 1 | 1 |
| ICC-C2 | 3 | 75986717 | 197566254 | 1 | 1 |
| ICC-C2 | 4 | 67846 | 190903950 | 1 | 1 |
| ICC-C2 | 5 | 163205 | 180632059 | 1 | 1 |
| ICC-C2 | 6 | 335251 | 170892848 | 1 | 1 |
| ICC-C2 | 7 | 590148 | 158827326 | 1 | 1 |
| ICC-C2 | 8 | 182949 | 146157339 | 1 | 1 |
| ICC-C2 | 9 | 214706 | 140938183 | 1 | 1 |
| ICC-C2 | 10 | 285481 | 135381592 | 1 | 1 |
| ICC-C2 | 11 | 193112 | 71169547 | 1 | 1 |
| ICC-C2 | 11 | 71174452 | 134226278 | 1 | 1 |
| ICC-C2 | 12 | 235143 | 133683020 | 1 | 1 |
| ICC-C2 | 13 | 19756002 | 115091399 | 1 | 1 |
| ICC-C2 | 14 | 20201972 | 107282988 | 1 | 1 |
| ICC-C2 | 15 | 20453896 | 102192014 | 1 | 1 |
| ICC-C2 | 16 | 97610 | 90095620 | 1 | 1 |
| ICC-C2 | 17 | 6115 | 81006387 | 1 | 1 |
| ICC-C2 | 18 | 480801 | 77921406 | 1 | 1 |
| ICC-C2 | 19 | 311825 | 59059493 | 1 | 1 |
| ICC-C2 | 20 | 76962 | 62854566 | 1 | 1 |
| ICC-C2 | 21 | 9825966 | 48078611 | 1 | 1 |
| ICC-C2 | 22 | 17449076 | 51219006 | 1 | 1 |
| ICC-C3 | 1 | 879317 | 31973215 | 1 | 1 |
| ICC-C3 | 1 | 31973348 | 249150116 | 1 | 1 |
| ICC-C3 | 2 | 672976 | 242839363 | 1 | 1 |
| ICC-C3 | 3 | 239555 | 100368546 | 1 | 1 |
| ICC-C3 | 3 | 100374073 | 197566254 | 1 | 1 |
| ICC-C3 | 4 | 67846 | 190903950 | 1 | 1 |
| ICC-C3 | 5 | 163205 | 180632059 | 1 | 1 |
| ICC-C3 | 6 | 335251 | 170892848 | 1 | 1 |
| ICC-C3 | 7 | 590148 | 158827326 | 1 | 1 |
| ICC-C3 | 8 | 182949 | 146157339 | 1 | 1 |
| ICC-C3 | 9 | 214706 | 140938183 | 1 | 1 |
| ICC-C3 | 10 | 285481 | 135381592 | 1 | 1 |
| ICC-C3 | 11 | 193112 | 134226278 | 1 | 1 |
| ICC-C3 | 12 | 235143 | 133683020 | 1 | 1 |
| ICC-C3 | 13 | 19756002 | 115091399 | 1 | 1 |
| ICC-C3 | 14 | 20201972 | 107282988 | 1 | 1 |
| ICC-C3 | 15 | 20453896 | 102192014 | 1 | 1 |
| ICC-C3 | 16 | 97610 | 90095620 | 1 | 1 |
| ICC-C3 | 17 | 6115 | 81006387 | 1 | 1 |
| ICC-C3 | 18 | 480801 | 77921406 | 1 | 1 |
| ICC-C3 | 19 | 311825 | 59059493 | 1 | 1 |
| ICC-C3 | 20 | 76962 | 62854566 | 1 | 1 |
| ICC-C3 | 21 | 9825966 | 48078611 | 1 | 1 |
| ICC-C3 | 22 | 17449076 | 51219006 | 1 | 1 |
| IM1 | 1 | 879317 | 120484421 | 1 | 1 |
| IM1 | 1 | 120539213 | 152185500 | 1 | 2 |
| IM1 | 1 | 152185750 | 223954080 | 1 | 3 |
| IM1 | 1 | 224318151 | 249150116 | 1 | 2 |
| IM1 | 2 | 672976 | 242839363 | 1 | 1 |
| IM1 | 3 | 239555 | 197566254 | 1 | 1 |
| IM1 | 4 | 67846 | 190903950 | 1 | 1 |
| IM1 | 5 | 163205 | 180632059 | 1 | 2 |
| IM1 | 6 | 335251 | 57255032 | 1 | 1 |
| IM1 | 6 | 57372361 | 57457704 | 0 | 1 |
| IM1 | 6 | 57457737 | 170892848 | 1 | 1 |
| IM1 | 7 | 590148 | 100488658 | 1 | 1 |
| IM1 | 7 | 100549505 | 100549593 | 0 | 1 |
| IM1 | 7 | 100549650 | 100549788 | 1 | 1 |
| IM1 | 7 | 100549873 | 100549942 | 0 | 1 |
| IM1 | 7 | 100549979 | 100552675 | 1 | 1 |
| IM1 | 7 | 100552711 | 100552739 | 0 | 1 |
| IM1 | 7 | 100552788 | 142045613 | 1 | 1 |
| IM1 | 7 | 142045678 | 142045693 | 0 | 1 |
| IM1 | 7 | 142045802 | 142144111 | 0 | 1 |
| IM1 | 7 | 142180704 | 142180770 | 4 | 4 |
| IM1 | 7 | 142197897 | 142459065 | 1 | 1 |
| IM1 | 7 | 142470574 | 142470612 | 2 | 4 |
| IM1 | 7 | 142498673 | 142498708 | 0 | 1 |
| IM1 | 7 | 142498735 | 158827326 | 1 | 1 |
| IM1 | 8 | 182949 | 43147940 | 0 | 1 |
| IM1 | 8 | 48585975 | 146157339 | 1 | 4 |
| IM1 | 9 | 214706 | 140938183 | 1 | 1 |
| IM1 | 10 | 285481 | 127585040 | 1 | 2 |
| IM1 | 10 | 127585090 | 127585237 | 0 | 1 |
| IM1 | 10 | 127585260 | 135381592 | 1 | 2 |
| IM1 | 11 | 193112 | 1011490 | 1 | 1 |
| IM1 | 11 | 1016910 | 1016933 | 4 | 4 |
| IM1 | 11 | 1016959 | 1093600 | 1 | 2 |
| IM1 | 11 | 1093622 | 1213264 | 0 | 2 |
| IM1 | 11 | 1213272 | 48367097 | 1 | 1 |
| IM1 | 11 | 48367133 | 48367170 | 0 | 1 |
| IM1 | 11 | 48367190 | 55339768 | 0 | 1 |
| IM1 | 11 | 56143528 | 56143564 | 0 | 1 |
| IM1 | 11 | 56143570 | 56143699 | 0 | 1 |
| IM1 | 11 | 56143716 | 56143786 | 0 | 1 |
| IM1 | 11 | 56230352 | 134226278 | 1 | 1 |
| IM1 | 12 | 235143 | 133683020 | 1 | 1 |
| IM1 | 13 | 19756002 | 115091399 | 1 | 1 |
| IM1 | 14 | 20201972 | 38266074 | 1 | 2 |
| IM1 | 14 | 38276433 | 107282988 | 0 | 2 |
| IM1 | 15 | 20453896 | 28259962 | 1 | 1 |
| IM1 | 15 | 28517258 | 28517310 | 0 | 1 |
| IM1 | 15 | 28517436 | 102192014 | 1 | 1 |
| IM1 | 16 | 97610 | 90095620 | 1 | 1 |
| IM1 | 17 | 6115 | 81006387 | 1 | 1 |
| IM1 | 18 | 480801 | 77921406 | 1 | 1 |
| IM1 | 19 | 311825 | 10659737 | 1 | 2 |
| IM1 | 19 | 10665924 | 10665945 | 0 | 0 |
| IM1 | 19 | 10668383 | 59059493 | 1 | 2 |
| IM1 | 20 | 76962 | 29652010 | 1 | 1 |
| IM1 | 20 | 29652036 | 31074372 | 0 | 1 |
| IM1 | 20 | 31196471 | 62854566 | 1 | 2 |
| IM1 | 21 | 9825966 | 48078611 | 1 | 1 |
| IM1 | 22 | 17449076 | 51219006 | 1 | 1 |
| IM2 | 1 | 879317 | 1647686 | 1 | 2 |
| IM2 | 1 | 1647689 | 1647730 | 0 | 2 |
| IM2 | 1 | 1647745 | 120484421 | 1 | 1 |
| IM2 | 1 | 120539213 | 145115820 | 1 | 2 |
| IM2 | 1 | 145282093 | 223954080 | 1 | 3 |
| IM2 | 1 | 224318151 | 249150116 | 1 | 2 |
| IM2 | 2 | 672976 | 242839363 | 1 | 1 |
| IM2 | 3 | 239555 | 197566254 | 1 | 1 |
| IM2 | 4 | 67846 | 1087239 | 1 | 1 |
| IM2 | 4 | 1087324 | 1087327 | 0 | 0 |
| IM2 | 4 | 1161190 | 190903950 | 1 | 1 |
| IM2 | 5 | 163205 | 180632059 | 1 | 2 |
| IM2 | 6 | 335251 | 57372361 | 1 | 1 |
| IM2 | 6 | 57372436 | 57457704 | 0 | 1 |
| IM2 | 6 | 57457737 | 170892848 | 1 | 1 |
| IM2 | 7 | 590148 | 100488658 | 1 | 1 |
| IM2 | 7 | 100549505 | 100549548 | 0 | 1 |
| IM2 | 7 | 100549573 | 100549873 | 0 | 1 |
| IM2 | 7 | 100549884 | 100549942 | 0 | 1 |
| IM2 | 7 | 100549979 | 100552675 | 1 | 1 |
| IM2 | 7 | 100552711 | 100552739 | 0 | 1 |
| IM2 | 7 | 100552788 | 142045613 | 1 | 1 |
| IM2 | 7 | 142045678 | 142045693 | 0 | 1 |
| IM2 | 7 | 142045802 | 142144111 | 1 | 1 |
| IM2 | 7 | 142180704 | 142180737 | 4 | 4 |
| IM2 | 7 | 142180770 | 142458987 | 1 | 1 |
| IM2 | 7 | 142459042 | 142470612 | 2 | 3 |
| IM2 | 7 | 142498673 | 142498708 | 0 | 1 |
| IM2 | 7 | 142498735 | 158827326 | 1 | 1 |
| IM2 | 8 | 182949 | 43147940 | 0 | 1 |
| IM2 | 8 | 48585975 | 146157339 | 1 | 4 |
| IM2 | 9 | 214706 | 140938183 | 1 | 1 |
| IM2 | 10 | 285481 | 127585040 | 1 | 2 |
| IM2 | 10 | 127585090 | 127585150 | 0 | 1 |
| IM2 | 10 | 127585173 | 135053123 | 1 | 3 |
| IM2 | 10 | 135077113 | 135103311 | 0 | 0 |
| IM2 | 10 | 135126477 | 135381592 | 1 | 2 |
| IM2 | 11 | 193112 | 1011490 | 1 | 1 |
| IM2 | 11 | 1016910 | 1016933 | 4 | 4 |
| IM2 | 11 | 1016959 | 1213245 | 0 | 2 |
| IM2 | 11 | 1213257 | 1213264 | 0 | 1 |
| IM2 | 11 | 1213272 | 48367097 | 1 | 1 |
| IM2 | 11 | 48367133 | 48367170 | 0 | 1 |
| IM2 | 11 | 48367190 | 55339768 | 0 | 1 |
| IM2 | 11 | 56143528 | 56143570 | 0 | 1 |
| IM2 | 11 | 56143592 | 56143699 | 0 | 1 |
| IM2 | 11 | 56143716 | 56143786 | 0 | 1 |
| IM2 | 11 | 56230352 | 134226278 | 1 | 1 |
| IM2 | 12 | 235143 | 133683020 | 1 | 1 |
| IM2 | 13 | 19756002 | 115091399 | 1 | 1 |
| IM2 | 14 | 20201972 | 38276433 | 1 | 2 |
| IM2 | 14 | 39512122 | 107282988 | 0 | 2 |
| IM2 | 15 | 20453896 | 28259962 | 1 | 1 |
| IM2 | 15 | 28517258 | 28517310 | 0 | 1 |
| IM2 | 15 | 28517436 | 102192014 | 1 | 1 |
| IM2 | 16 | 97610 | 90095620 | 1 | 1 |
| IM2 | 17 | 6115 | 81006387 | 1 | 1 |
| IM2 | 18 | 480801 | 77921406 | 1 | 1 |
| IM2 | 19 | 311825 | 59059493 | 1 | 2 |
| IM2 | 20 | 76962 | 29630695 | 1 | 1 |
| IM2 | 20 | 29633916 | 29652294 | 0 | 1 |
| IM2 | 20 | 29652303 | 62854566 | 1 | 2 |
| IM2 | 21 | 9825966 | 48078611 | 1 | 1 |
| IM2 | 22 | 17449076 | 51219006 | 1 | 1 |
| TIS | 1 | 879317 | 249150116 | 1 | 1 |
| TIS | 2 | 672976 | 242839363 | 1 | 1 |
| TIS | 3 | 239555 | 197566254 | 1 | 1 |
| TIS | 4 | 67846 | 190903950 | 1 | 1 |
| TIS | 5 | 163205 | 180632059 | 1 | 1 |
| TIS | 6 | 335251 | 170892848 | 1 | 1 |
| TIS | 7 | 590148 | 100549593 | 1 | 1 |
| TIS | 7 | 100549650 | 100675976 | 1 | 1 |
| TIS | 7 | 100731925 | 158827326 | 1 | 1 |
| TIS | 8 | 182949 | 146157339 | 1 | 1 |
| TIS | 9 | 214706 | 140938183 | 1 | 1 |
| TIS | 10 | 285481 | 135381592 | 1 | 1 |
| TIS | 11 | 193112 | 134226278 | 1 | 1 |
| TIS | 12 | 235143 | 133683020 | 1 | 1 |
| TIS | 13 | 19756002 | 115091399 | 1 | 1 |
| TIS | 14 | 20201972 | 107282988 | 1 | 1 |
| TIS | 15 | 20453896 | 102192014 | 1 | 1 |
| TIS | 16 | 97610 | 90095620 | 1 | 1 |
| TIS | 17 | 6115 | 81006387 | 1 | 1 |
| TIS | 18 | 480801 | 77921406 | 1 | 1 |
| TIS | 19 | 311825 | 59059493 | 1 | 1 |
| TIS | 20 | 76962 | 62854566 | 1 | 1 |
| TIS | 21 | 9825966 | 48078611 | 1 | 1 |
| TIS | 22 | 17449076 | 51219006 | 1 | 1 |
|  |  |  |  |  |  |
|  |  |  |  |  |  |
