# Supplementary material for: Inferring the progression of multifocal liver cancer from spatial and temporal genomic heterogeneity
Source: Oncotarget. 2015 Dec 11;7(3):2867–77. doi: 10.18632/oncotarget.6558 (PMC4823077; doi:10.18632/oncotarget.6558)
Supplement: Supplementary file 6 [file oncotarget-07-2867-s006.docx]

| **Supplementary Table 5. Pathway enrichment of the genes with nonsynonymous somatic alterarions in coding regions of tumor exomes.** | | | | | | | | | | | |  |
| --- | --- | --- | --- | --- | --- | --- | --- | --- | --- | --- | --- | --- |
| **Sample** | **Category** | **Term** | **Count** | **P Value** | **Genes** | **List Total** | **Pop Hits** | **Pop Total** | **Fold Enrichment** | **Bonferroni** | **Benjamini** | **FDR** |
| HCC-A | SP_PIR_KEYWORDS | **glycoprotein** | 21 | 1.65E-05 | FLRT2, SCN1A, CUBN, SDK1, HSPG2, STAB2, FCRL5, CSMD2, SLC10A1, MUC4, FGG, APOB, FGA, VWDE, FAT4, RYR3, SEMA3E, KCNH8, CACNA1E, OR10K1, DSCAM | 37 | 4318 | 19235 | 2.52829 | 0.00206 | 0.002058 | 0.01906 |
|  | SP_PIR_KEYWORDS | **egf-like domain** | 6 | 6.52E-05 | CUBN, **FAT4**, VWDE, HSPG2, STAB2, MUC4 | 37 | 230 | 19235 | 13.5617 | 0.00812 | 0.004067 | 0.07538 |
|  | SP_PIR_KEYWORDS | disulfide bond | 16 | 1.19E-04 | OBSCN, CUBN, HSPG2, SDK1, STAB2, FCRL5, CSMD2, MUC4, FGG, APOB, FGA, VWDE, FAT4, SEMA3E, CACNA1E, DSCAM | 37 | 2924 | 19235 | 2.84468 | 0.01476 | 0.004944 | 0.13745 |
|  | SP_PIR_KEYWORDS | Immunoglobulin domain | 6 | 1.72E-03 | OBSCN, SEMA3E, SDK1, HSPG2, FCRL5, DSCAM | 37 | 470 | 19235 | 6.63657 | 0.19364 | 0.052385 | 1.97144 |
|  | SP_PIR_KEYWORDS | polymorphism | 31 | 2.44E-03 | DNAH9, SCN1A, IL16, CNOT1, FCRL5, ZNF679, CSMD2, SPHKAP, APOB, DOCK2, FGG, FGA, FAT4, VWDE, DSCAM, OBSCN, FLRT2, CUBN, KIF17, SDK1, HSPG2, STAB2, ITPR3, MUC4, SLC10A1, CCNB3, MYO10, RYR3, CACNA1E, KCNH8, EP400 | 37 | 11550 | 19235 | 1.39531 | 0.26351 | 0.059337 | 2.78989 |
|  | SP_PIR_KEYWORDS | ionic channel | 5 | 2.85E-03 | SCN1A, RYR3, CACNA1E, KCNH8, ITPR3 | 37 | 318 | 19235 | 8.17398 | 0.29967 | 0.05764 | 3.24166 |
|  | SP_PIR_KEYWORDS | calcium | 7 | 3.46E-03 | FGG, APOB, CUBN, FAT4, RYR3, CACNA1E, ITPR3 | 37 | 803 | 19235 | 4.53182 | 0.35193 | 0.060085 | 3.93339 |
|  | SP_PIR_KEYWORDS | ion transport | 6 | 4.20E-03 | SCN1A, RYR3, CACNA1E, KCNH8, ITPR3, SLC10A1 | 37 | 578 | 19235 | 5.39652 | 0.4092 | 0.063667 | 4.7521 |
|  | SP_PIR_KEYWORDS | signal | 14 | 4.36E-03 | FLRT2, CUBN, HSPG2, SDK1, STAB2, FCRL5, MUC4, FGG, APOB, FGA, FAT4, VWDE, SEMA3E, DSCAM | 37 | 3250 | 19235 | 2.23942 | 0.42086 | 0.058885 | 4.92763 |
|  | SP_PIR_KEYWORDS | liver | 3 | 5.45E-03 | FGG, APOB, FGA | 37 | 59 | 19235 | 26.4338 | 0.49493 | 0.066025 | 6.12359 |
|  | SP_PIR_KEYWORDS | alternative splicing | 23 | 5.78E-03 | OBSCN, PHLDB1, DNAH9, SCN1A, IL16, KIF17, SDK1, CNOT1, RIMS2, FCRL5, CSMD2, MUC4, SPHKAP, FGG, CCNB3, DOCK2, FGA, FAT4, RYR3, CACNA1E, EP400, DSCAM, AXIN1 | 37 | 7488 | 19235 | 1.59681 | 0.51553 | 0.063758 | 6.48456 |
|  | SP_PIR_KEYWORDS | calcium channel | 3 | 5.81E-03 | RYR3, CACNA1E, ITPR3 | 37 | 61 | 19235 | 25.5671 | 0.5176 | 0.05894 | 6.52163 |
|  | SP_PIR_KEYWORDS | cell adhesion | 5 | 7.73E-03 | FLRT2, FAT4, SDK1, DSCAM, MUC4 | 37 | 422 | 19235 | 6.15954 | 0.62073 | 0.071864 | 8.57865 |
|  | SP_PIR_KEYWORDS | transport | 9 | 1.06E-02 | SCN1A, APOB, CUBN, KIF17, RYR3, CACNA1E, KCNH8, ITPR3, SLC10A1 | 37 | 1670 | 19235 | 2.80167 | 0.73694 | 0.090977 | 11.6214 |
|  | SP_PIR_KEYWORDS | calcium transport | 3 | 1.10E-02 | RYR3, CACNA1E, ITPR3 | 37 | 85 | 19235 | 18.3482 | 0.75002 | 0.088282 | 12.0373 |
|  | SP_PIR_KEYWORDS | Secreted | 9 | 1.13E-02 | FGG, APOB, FGA, IL16, VWDE, SEMA3E, HSPG2, DSCAM, MUC4 | 37 | 1689 | 19235 | 2.77015 | 0.75963 | 0.085244 | 12.3557 |
|  | SP_PIR_KEYWORDS | plasma | 3 | 1.31E-02 | FGG, APOB, FGA | 37 | 93 | 19235 | 16.7698 | 0.80748 | 0.092368 | 14.1374 |
|  | SP_PIR_KEYWORDS | membrane | 19 | 2.20E-02 | FLRT2, SCN1A, CUBN, SDK1, STAB2, ITPR3, RIMS2, FCRL5, CSMD2, SLC10A1, MUC4, PPP1R16B, DOCK2, FAT4, RYR3, KCNH8, CACNA1E, OR10K1, DSCAM | 37 | 6256 | 19235 | 1.57887 | 0.93772 | 0.142925 | 22.6499 |
|  | SP_PIR_KEYWORDS | motor protein | 3 | 2.53E-02 | MYO10, DNAH9, KIF17 | 37 | 132 | 19235 | 11.8151 | 0.95931 | 0.155079 | 25.6362 |
|  | SP_PIR_KEYWORDS | voltage-gated channel | 3 | 3.20E-02 | SCN1A, CACNA1E, KCNH8 | 37 | 150 | 19235 | 10.3973 | 0.98287 | 0.183993 | 31.3547 |
|  | SP_PIR_KEYWORDS | egf-like domain | 6 | 2.61E-05 | CUBN, FAT4, VWDE, TNR, STAB2, MUC4 | 31 | 230 | 19235 | 16.1865 | 0.00284 | 0.002844 | 0.02947 |
| HCC-B | SP_PIR_KEYWORDS | **phosphoprotein** | 10 | 3.74E-03 | FLRT3, MYO3A, MYH1, FAT4, SLC30A1, ZNF609, PCLO, RNF213, MUC16, ENOX1 | 12 | 7263 | 19235 | 2.20696 | 0.1954 | 0.1954 | 3.66076 |
|  | SP_PIR_KEYWORDS | **cell adhesion** | 3 | 2.32E-02 | FLRT3, CDH7, **FAT4** | 12 | 422 | 19235 | 11.3951 | 0.74323 | 0.493275 | 20.8022 |
|  | SP_PIR_KEYWORDS | myosin | 2 | 2.88E-02 | MYO3A, MYH1 | 12 | 51 | 19235 | 62.8595 | 0.81627 | 0.431506 | 25.2215 |
|  | SP_PIR_KEYWORDS | cell membrane | 5 | 2.88E-02 | CDH7, SLC30A1, OR8K3, MUC16, ENOX1 | 12 | 2194 | 19235 | 3.65296 | 0.8165 | 0.345503 | 25.2377 |
| IM | SP_PIR_KEYWORDS | **egf-like domain** | 6 | 2.61E-05 | CUBN, **FAT4,** VWDE, TNR, STAB2, MUC4 | 31 | 230 | 19235 | 16.1865 | 0.00284 | 0.002844 | 0.02947 |
|  | SP_PIR_KEYWORDS | polymorphism | 29 | 4.78E-05 | KIF4B, DNAH9, SCN1A, DNAH12, CNOT1, TTN, ZNF679, FCRL5, CSMD2, SPHKAP, DOCK2, FGG, FGA, FAT4, VWDE, TNR, DSCAM, OBSCN, CUBN, KIF17, SDK1, STAB2, ITPR3, SLC10A1, MUC4, CCNB3, MYO10, KCNH8, EP400 | 31 | 11550 | 19235 | 1.55792 | 0.0052 | 0.002603 | 0.05393 |
|  | SP_PIR_KEYWORDS | motor protein | 5 | 5.06E-05 | KIF4B, MYO10, DNAH9, DNAH12, KIF17 | 31 | 132 | 19235 | 23.5032 | 0.0055 | 0.001835 | 0.05702 |
|  | SP_PIR_KEYWORDS | disulfide bond | 14 | 2.10E-04 | OBSCN, CUBN, SDK1, STAB2, TTN, FCRL5, CSMD2, MUC4, FGG, FGA, FAT4, VWDE, TNR, DSCAM | 31 | 2924 | 19235 | 2.97085 | 0.02265 | 0.005711 | 0.2368 |
|  | SP_PIR_KEYWORDS | glycoprotein | 16 | 8.50E-04 | SCN1A, CUBN, SDK1, STAB2, FCRL5, CSMD2, SLC10A1, MUC4, FGG, FGA, VWDE, FAT4, TNR, KCNH8, OR10K1, DSCAM | 31 | 4318 | 19235 | 2.29915 | 0.0885 | 0.018363 | 0.95433 |
|  | SP_PIR_KEYWORDS | alternative splicing | 21 | 1.93E-03 | OBSCN, KIF4B, SCN1A, DNAH9, DNAH12, KIF17, SDK1, CNOT1, TTN, FCRL5, CSMD2, MUC4, SPHKAP, FGG, CCNB3, DOCK2, FGA, FAT4, TNR, EP400, DSCAM | 31 | 7488 | 19235 | 1.74014 | 0.18997 | 0.034505 | 2.15659 |
|  | SP_PIR_KEYWORDS | coiled coil | 10 | 2.80E-03 | KIF4B, FGG, PPP1R16B, MYO10, DNAH9, DNAH12, FGA, KIF17, TNR, TTN | 31 | 2019 | 19235 | 3.07322 | 0.26342 | 0.042737 | 3.11427 |
|  | SP_PIR_KEYWORDS | atp-binding | 8 | 3.64E-03 | KIF4B, OBSCN, MYO10, DNAH9, DNAH12, KIF17, TTN, EP400 | 31 | 1326 | 19235 | 3.74349 | 0.32765 | 0.048411 | 4.02461 |
|  | SP_PIR_KEYWORDS | cell adhesion | 5 | 3.98E-03 | FAT4, TNR, SDK1, DSCAM, MUC4 | 31 | 422 | 19235 | 7.3517 | 0.35285 | 0.047203 | 4.4033 |
|  | SP_PIR_KEYWORDS | microtubule | 4 | 5.46E-03 | KIF4B, DNAH9, DNAH12, KIF17 | 31 | 231 | 19235 | 10.7443 | 0.44968 | 0.057977 | 5.9932 |
|  | SP_PIR_KEYWORDS | Immunoglobulin domain | 5 | 5.83E-03 | OBSCN, SDK1, TTN, FCRL5, DSCAM | 31 | 470 | 19235 | 6.60089 | 0.47128 | 0.05629 | 6.38196 |
|  | SP_PIR_KEYWORDS | nucleotide-binding | 8 | 1.32E-02 | KIF4B, OBSCN, MYO10, DNAH9, DNAH12, KIF17, TTN, EP400 | 31 | 1686 | 19235 | 2.94417 | 0.76618 | 0.114055 | 13.9615 |
|  | SP_PIR_KEYWORDS | signal | 11 | 2.15E-02 | FGG, CUBN, FGA, FAT4, VWDE, TNR, SDK1, STAB2, FCRL5, DSCAM, MUC4 | 31 | 3250 | 19235 | 2.1001 | 0.90607 | 0.166349 | 21.7101 |
|  | SP_PIR_KEYWORDS | calcium | 5 | 3.49E-02 | FGG, CUBN, FAT4, ITPR3, TTN | 31 | 803 | 19235 | 3.86354 | 0.97916 | 0.241555 | 33.0045 |
|  | SP_PIR_KEYWORDS | dynein | 2 | 4.88E-02 | DNAH9, DNAH12 | 31 | 32 | 19235 | 38.7802 | 0.9957 | 0.304586 | 43.0974 |
|  |  |  |  |  |  |  |  |  |  |  |  |  |
|  |  |  |  |  |  |  |  |  |  |  |  |  |
