# Supplementary material for: Inferring the progression of multifocal liver cancer from spatial and temporal genomic heterogeneity
Source: Oncotarget. 2015 Dec 11;7(3):2867–77. doi: 10.18632/oncotarget.6558 (PMC4823077; doi:10.18632/oncotarget.6558)
Supplement: Supplementary file 7 [file oncotarget-07-2867-s007.docx]

| **Supplementary Table 6. Somatic mutations of FAT4 in HCC samples and cell lines.** | | | | | | |  |  |
| --- | --- | --- | --- | --- | --- | --- | --- | --- |
| **Exon** | **Nucleotide change** | **Amino acid change** | **Mutation type** | **COSMIC** | **Polyphen-2 prediction** | **Zygosity** | **Domain** |  |
| exon-1 | c.G451A | p.G151R | Missense |  | Probably damaging | Heterozygous | Cadherin 1 |  |
| exon-1 | c.G863T | p.R288L | Missense |  | Benign | Heterozygous | Cadherin 3 |  |
| exon-1 | c.G1333A | p.G445R | Missense |  | Probably damaging | Heterozygous | Cadherin 4 |  |
| exon-1 | c.A1358T | p.Q453L | Missense | COSM3760369 | Benign | Heterozygous | Cadherin 4 |  |
| exon-1 | c.A2167C | p.T723P | Missense |  | Probably damaging | Heterozygous | Cadherin 7 |  |
| exon-1 | c.C2420T | p.A807V | Missense | COSM3760371 | Probably damaging | **Homozygous** | Cadherin 8 |  |
| exon-1 | c.G2530A | p.V844I | Missense |  | Possibly damaging | Heterozygous | Cadherin 8 |  |
| exon-1 | c.C3769G | p.Q1257E | Missense | COSM3760373 | Benign | Heterozygous | Cadherin 12 |  |
| exon-5 | c.G4129A | p.V1407I | Missense |  | Benign | Heterozygous | Cadherin 13 |  |
| exon-5 | c.C5946A | p.S1982R | Missense |  | Benign | Heterozygous | Cadherin 19 |  |
| exon-5 | c.G5993A | p.G1998D | Missense |  | Probably damaging | Heterozygous | Cadherin 19 |  |
| exon-5 | c.T6773C | p.V2258A | Missense |  | Benign | Heterozygous | Cadherin 21 |  |
| exon-9 | c.A10810C | p.I3602L | Missense |  | Possibly damaging | Heterozygous | Cadherin 34 |  |
| exon-16 | c.G12817T | p.V4273L | Missense |  | Benign | Heterozygous | Laminin G-like 2 |  |
| exon-17 | c.G13828T | p.A4608S | Missense |  | Probably damaging | Heterozygous | Cytoplasmic |  |
| exon-17 | c.C13927T | p.Q4641* | Nonsense |  | / | Heterozygous | Cytoplasmic |  |
| exon-17 | c.G14184T | p.R4726S | Missense |  | Probably damaging | Heterozygous | Cytoplasmic |  |
| exon-17 | c.G14362A | p.G4786R | Missense |  | Probably damaging | Heterozygous | Cytoplasmic |  |
| exon-17 | c.A14804C | p.N4935T | Missense |  | Possibly damaging | Heterozygous | Cytoplasmic |  |
| exon-17 | c.C14920T | p.P4972S | Missense |  | Benign | **Homozygous** | Cytoplasmic |  |
| exon-17 | c.G14935A | p.A4977T | Missense |  | Benign | Heterozygous | Cytoplasmic |  |
|  |  |  |  |  |  |  |  |  |
|  |  |  |  |  |  |  |  |  |
