# Supplementary material for: Inferring the progression of multifocal liver cancer from spatial and temporal genomic heterogeneity
Source: Oncotarget. 2015 Dec 11;7(3):2867–77. doi: 10.18632/oncotarget.6558 (PMC4823077; doi:10.18632/oncotarget.6558)
Supplement: Supplementary file 8 [file oncotarget-07-2867-s008.docx]

| **Supplementary Table 7. HCC cell lines used in this study.** | | |  |
| --- | --- | --- | --- |
| **Cell lines** | **FAT4 mutation** | **FAT4 expression** |  |
| BEL-7404 | N | 0.12 |  |
| Hep3B | N | 0.17 |  |
| HepG2 | Y | 0.13 |  |
| HLE | N | 0.95 |  |
| HLF | Y | 0.37 |  |
| Huh-1 | N | 0.09 |  |
| Huh-6 | Y | 0.11 |  |
| Huh7 | N | 0.13 |  |
| JHH-1 | N | 0.15 |  |
| JHH-4 | Y | 0.35 |  |
| JHH-5 | Y | 0.18 |  |
| JHH-6 | Y | 0.50 |  |
| JHH-7 | N | 0.08 |  |
| MHCC97L | Y (3)* | 0.07 |  |
| MHCC97H | Y (3) | 0.02 |  |
| HCCLM3 | Y (3) | 0.02 |  |
| PLC/PRE/5 | Y (2) | 0.12 |  |
| SK-HEP1 | Y | 2.12 |  |
| SMMC7721 | N | 0.16 |  |
| SNU-354 | Y (2) | 0.22 |  |
| SNU-398 | N | 1.12 |  |
| SNU-423 | Y | 1.24 |  |
| SNU-449 | N | 0.86 |  |
| SNU-739 | N | 0.47 |  |
| SNU-886 | Y | 0.32 |  |
|  | | |  |
| Y, yes. N, no. |  | |  |
| Numbers in the bracket indicate the number of mutations. | | |  |
|  |  |  |  |
