# Supplementary material for: Inferring the progression of multifocal liver cancer from spatial and temporal genomic heterogeneity
Source: Oncotarget. 2015 Dec 11;7(3):2867–77. doi: 10.18632/oncotarget.6558 (PMC4823077; doi:10.18632/oncotarget.6558)
Supplement: Supplementary file 9 [file oncotarget-07-2867-s009.docx]

| **Supplementary Table 8. Clinical characteristics of 60 HBV related-HCC patients for mutation prevelance screen.** | | | |
| --- | --- | --- | --- |
| **Characteristics** | ***FAT4* mutation** | | |
|  | **No** | **Yes** | ***P*** |
| Age, years |  | | |
| ≤51 | 17 | 9 | 0.252 |
| > 51 | 27 | 7 |  |
| Gender |  | | |
| Female | 7 | 3 | 1.00 |
| Male | 37 | 13 |  |
| Hepatitis history |  | | |
| No | 8 | 1 | 0.442 |
| Yes | 36 | 15 |  |
| α-Fetoprotein (ng/ml) |  | | |
| ≤20 | 25 | 7 | 0.272 |
| > 20 | 19 | 9 |  |
| γ-Glutamyl transferase (U/l) |  | | |
| ≤54 | 20 | 6 | 0.769 |
| >54 | 24 | 10 |  |
| Liver cirrhosis |  | | |
| No | 9 | 3 | 1.00 |
| Yes | 35 | 13 |  |
| Tumor size (cm) |  | | |
| ≤5 | 30 | 11 | 1.00 |
| > 5 | 14 | 5 |  |
| Tumor encapsulation |  | | |
| Complete | 27 | 10 | 0.825 |
| None | 17 | 6 |  |
| Tumor number |  | | |
| Single | 28 | 9 | 0.765 |
| Multiple | 16 | 7 |  |
| Vascular invasion |  | | |
| No | 33 | 7 | **0.032** |
| Yes | 11 | 9 |  |
| Tumor differentiation |  | | |
| I-II | 37 | 10 | **0.088** |
| III-IV | 7 | 6 |  |
| TNM stage |  | | |
| I | 21 | 5 | 0.426 |
| II | 13 | 5 |  |
| III | 10 | 6 |  |
| BCLC stage |  | | |
| 0-A | 24 | 8 | 0.767 |
| B-C | 20 | 8 |  |
|  | | | |
| P values were calculated by the Fisher's exact test. | | |  |
|  |  |  |  |
