# Supplementary material for: Inferring the progression of multifocal liver cancer from spatial and temporal genomic heterogeneity
Source: Oncotarget. 2015 Dec 11;7(3):2867–77. doi: 10.18632/oncotarget.6558 (PMC4823077; doi:10.18632/oncotarget.6558)
Supplement: Supplementary file 10 [file oncotarget-07-2867-s010.docx]

| **Supplementary Table 9. Clinical characteristics of 236 HBV related-HCC patients for immunostaining.** | | | |  |
| --- | --- | --- | --- | --- |
| **Characteristics** | ***FAT4* expression** | | |  |
|  | **High** | **Low** | ***P*** |  |
| Age, years |  | | |  |
| ≤51 | 68 | 53 | 0.896 |  |
| > 51 | 63 | 52 |  |  |
| Gender |  | | |  |
| Female | 19 | 11 | 0.433 |  |
| Male | 112 | 94 |  |  |
| Hepatitis history |  | | |  |
| No | 2 | 1 | 1.00 |  |
| Yes | 129 | 104 |  |  |
| α-Fetoprotein (ng/ml) |  |  | |  |
| ≤20 | 46 | 47 | 0.142 |  |
| > 20 | 85 | 58 |  |  |
| γ-Glutamyl transferase (U/l) |  | | |  |
| ≤54 | 20 | 26 | 0.072 |  |
| >54 | 111 | 79 |  |  |
| Liver cirrhosis |  | | |  |
| No | 21 | 7 | 0.041 |  |
| Yes | 110 | 98 |  |  |
| Tumor size (cm) |  | | |  |
| ≤5 | 70 | 40 | **0.025** |  |
| > 5 | 61 | 65 |  |  |
| Tumor encapsulation |  | | |  |
| Complete | 58 | 46 | 1.00 |  |
| None | 73 | 59 |  |  |
| Tumor number |  | | |  |
| Single | 105 | 89 | 0.395 |  |
| Multiple | 26 | 16 |  |  |
| Vascular invasion |  | | |  |
| No | 88 | 64 | 0.341 |  |
| Yes | 43 | 41 |  |  |
| Tumor differentiation |  | | |  |
| I-II | 90 | 73 | 1.00 |  |
| III-IV | 41 | 32 |  |  |
| TNM stage |  | | |  |
| I | 73 | 56 | 0.921 |  |
| II | 31 | 27 |  |  |
| III | 27 | 22 |  |  |
| BCLC stage |  | | |  |
| 0-A | 60 | 33 | **0.032** |  |
| B-C | 71 | 72 |  |  |
|  | | | |  |
| P values were calculated by the Fisher's exact test. | |  | |  |
|  |  |  |  |  |
