# Supplementary material for: Inferring the progression of multifocal liver cancer from spatial and temporal genomic heterogeneity
Source: Oncotarget. 2015 Dec 11;7(3):2867–77. doi: 10.18632/oncotarget.6558 (PMC4823077; doi:10.18632/oncotarget.6558)
Supplement: Supplementary file 11 [file oncotarget-07-2867-s011.docx]

| **Supplementary Table 10. Primer sets used for PCR validation of somatic mutations.** | | |
| --- | --- | --- |
| **Forward or reverse** | **Primers** | **Amplification Length** |
| ADAMTS12-F | CGTAGAATACCAAGGTGGCAAT | 442 |
| ADAMTS12-R | GGACAAAGAGTAAACCAGAGGG |  |
| ADAMTS3-F | CCACTACAGTAAATGCAAGACA | 414 |
| ADAMTS3-R | CTCAAGAAAATGATACCCGCTC |  |
| ADARB2-F | CAGCACCTGTTCTCCCAT | 600 |
| ADARB2-R | TTCCCCGACACGCTCTTC |  |
| ANKRD26-F | TGCAAAACACAGATCTAAAAAAAC | 450 |
| ANKRD26-R | GATGGGAAACAATACAATGAATAC |  |
| ANKRD34C-F | CTGCTTTTCATGTGGATGTTTT | 510 |
| ANKRD34C-R | ATCTTGGACTTGCTGATGCTTT |  |
| APOB-F | TCAGGTATGAAGTGGAAGAGGA | 470 |
| APOB-R | ATTTTTAGTAGAGACGGGGTTT |  |
| ATAD5-F | TAAATCCTAAACAAGGGACCAC | 594 |
| ATAD5-R | CAGAACTTCCAGCTTCCAAAAC |  |
| ATP4B-F | GGAAGTAAGTGGATGGAAAAAC | 582 |
| ATP4B-R | GGAACTAGGTAGGGAAGATGGT |  |
| ATP8A2-F | TGGTAACCTTTGTTGTCTGTGTTT | 512 |
| ATP8A2-R | TAGAATTGATTGTTCCCCTAATCC |  |
| AXIN1-F | GGCGTCACAGGCGACACT | 500 |
| AXIN1-R | CCAACCCCCAGACAGCAC |  |
| BMP7-F | AATTTGTTGTCGGTCATGGTTT | 408 |
| BMP7-R | TGTTGGTGTGTGTTCTCTTCCT |  |
| C1orf141-F | TTATCATCTACAGTTGTGTGGCC | 539 |
| C1orf141-R | TATTTCAAAAAACATTTAAGGCC |  |
| C21orf91-F | AAAAAAAGGAAAAATGTATTTCC | 425 |
| C21orf91-R | ACTTCTACTCCACACTTACGAGC |  |
| C3orf62-F | TTTTTAGTAGAGACGGGGTTT | 471 |
| C3orf62-R | GATGTAGCAGTTTCCAATTGT |  |
| CACNA1S-F | TGTTGGCCCTACCCTCTCTC | 596 |
| CACNA1S-R | TCTTATCCTTGCCCCTTCTT |  |
| CACNG8-F | GAGCCCTCTGGGTTGGA | 551 |
| CACNG8-R | GGTAGCGGAAGCGGTAA |  |
| CANT1-F | ACTCTGCCGCTGTCCTCCT | 587 |
| CANT1-R | TCCCTTTCCTTGGGTTCCT |  |
| CCDC62-F | GCTGAGGATGTGAGAGTTAGG | 529 |
| CCDC62-R | TTGAGGTTAGGAGTTCAAGAA |  |
| CCNB3-F | GGACTTTCAGGATATGATTGGT | 590 |
| CCNB3-R | ATTGTGTCTGTATGTTTGTGGC |  |
| CHEK1-F | TCTCAGGTTTAGAGTGCTCTTTTC | 592 |
| CHEK1-R | TTCCTTGGTTTATTTCATTTATTT |  |
| CLCN5-F | TGCTTTTGTGTCAGGAATTTT | 577 |
| CLCN5-R | TTGTGCTCATCCGAGTGTATT |  |
| CLIP4-F | TCCCTTAGGACCCATCTCAGA | 459 |
| CLIP4-R | AATTTTTCCCTTGCCAACTTT |  |
| CNOT1-F | AATAAATAAATAAATAAAACACCCC | 523 |
| CNOT1-R | GTTCTACATTAAACTTAAAACTGGA |  |
| COL24A1-F | CTTGAACACCATATTCTCCCT | 475 |
| COL24A1-R | CTTCCCTCTTCTTTCCACACA |  |
| CRYBB3-F | AGACCGTCCACATCTCAACCT | 441 |
| CRYBB3-R | CTCAGAACACTCAAGCCCCAG |  |
| CSMD2-F | TGCCCACTGTGGACCTAAT | 529 |
| CSMD2-R | TGTGCGTTCCCATCTTTTT |  |
| CUBN-F | AATAATTAGTTGTATGTGGTGATGT | 543 |
| CUBN-F | CGTTACACTTATTTCAGTTTGTTTC |  |
| CUBN-R | TTGATGTTTTAGTTACTGTTCCTGT | 409 |
| CUBN-R | TATATTTTTTCTTCCTCACTTCTTC |  |
| DCAF12L1-F | CTTGCGGTTACTGGGGTTG | 433 |
| DCAF12L1-R | TTTCGTGGTGGACGTGGAG |  |
| DMRT2-F | CTTCCCTCTACCTCCCCCA | 477 |
| DMRT2-R | CGTGCTCCCTTCAAGTCCC |  |
| DNAH9-F | CCTGACTGTCTTTGTTTTTGC | 457 |
| DNAH9-R | TCTTTCTCTGTGGCCTCTCTA |  |
| DOCK11-F | TCTAGATAAAGGATGGGGAGAG | 436 |
| DOCK11-R | GAACAAAAAATAATGCACAGGT |  |
| DOCK2-F | CATCCCATATTCATGTTCCACT | 578 |
| DOCK2-R | TTTCTAAGATCTCCTGTCCCAA |  |
| DPYS-F | CTCTGTATGTTATCAGCCTCCT | 524 |
| DPYS-R | ATTCTTTTTTTAAAGCTCTCGC |  |
| DSCAM-F | TTTTCTGAGTGCTCTGGGTTTG | 450 |
| DSCAM-R | TGTGGAGTATGATTGCGTGATG |  |
| ENOX1-F | CTCCTGCTTCCCCGTCG | 437 |
| ENOX1-R | AACTTGGCACTTTCCGC |  |
| EP400-F | CTCACTTTGCTCATCCCCCT | 538 |
| EP400-R | CCAACAGTAATTCCGCTTTC |  |
| ERVFRD-1-F | AAACCATATCCAGCCAGGTC | 551 |
| ERVFRD-1-R | CTCAAAGGAAATAGCCAACAA |  |
| FAM120A-F | GCTCCCTTTGATCTGGTATCC | 563 |
| FAM120A-R | ACTTCTTTCACAGCCTTGTTG |  |
| FAM135A-F | TACCAGTTTTTATCGCTGACCAA | 579 |
| FAM135A-R | ATCTCTGAAGATTCTGAATTCCC |  |
| FAM160B1-F | CTGTTAGTCAGAAAGCGGTTACT | 413 |
| FAM160B1-R | CAAGAATGTCCACTCAATAGGTT |  |
| FAM188B-F | GAGGTGGGAGCATTTGAGT | 493 |
| FAM188B-R | GCATCCTGTCCGTGTCTGT |  |
| FAT4-F1 | GAGAATGAGCCTGGAGGTAGC | 587 |
| FAT4-F2 | TCTAGAACAAGGAATCCAGCG |  |
| FAT4-R1 | TGAAGGAAATGGGGAGAGTTG | 569 |
| FAT4-R2 | TTTTTCAAATGTGACCCAACC |  |
| FCRL5-F | ACTGGTGACCCACGCTGATAT | 443 |
| FCRL5-R | GCTGCTTGAAGTGTGCTTTTC |  |
| FERMT1-F | ATGCTTGTGGTTTCTTAGGTC | 447 |
| FERMT1-R | TCATTCTTTTTTCACGCTGTC |  |
| FGA-F | GCCCCAGTCTGGGTTGTTA | 541 |
| FGA-R | CTTGGTTCCCGGAAATTTT |  |
| FGG-F | AAATGGGGAAAACACATTAAAAAT | 448 |
| FGG-R | ATCAAACCAAAGTAGACAAGGATC |  |
| FIG4-F | TTAAATAGGCATCTAAAAGCCA | 538 |
| FIG4-R | TCCTCAATAACAACCTCTCACC |  |
| FZD4-F | TTTGGCTTCCAGTCCCT | 569 |
| FZD4-R | GCATCACACTCCCGTCC |  |
| GSTO2-F | CGGAACCACAGAGAAAACCT | 598 |
| GSTO2-R | ACTTCAGCAGCCAAACACAA |  |
| GUCY1A2-F | GAAAAAGAAAATAGGGAGAGAGAAG | 471 |
| GUCY1A2-R | ACATGGAAAGAAGAAAGAAAAATAA |  |
| HERC2-F | TCTCGATCTCTTGACCTCGTGA | 442 |
| HERC2-R | ATGGATGGGAATGTAGCTTCTG |  |
| HMGCS2-F | GCAGCAGCTGTGTGAATATAT | 487 |
| HMGCS2-R | GGACCAAACTGACCTGGAGAA |  |
| HSPG2-F | GAAGTAGCAGTATGGGGTTGG | 557 |
| HSPG2-R | AATTCTGGCTCTTGTGGGAAG |  |
| IFT122-F | AAGGTTTGCATCTTTTTTTGA | 501 |
| IFT122-R | ACCATTGCTAGTTTTTGTCCG |  |
| IL16-F | TGTTGTAGCCCTCTCTCTTTCTT | 511 |
| IL16-R | CTCTCTGGGTATTTTTGTGTTTG |  |
| ISOC1-F | ATCATTCTTTTTCTTTTTTCCTC | 567 |
| ISOC1-R | TTCCTCATTATTACTGCCTACCT |  |
| ITPR3-F | GTCGACCCCACCACCAAAG | 439 |
| ITPR3-R | ACCAGCCACAAAGCAGCAG |  |
| JAG2-F | GTCAACACAGATGCCACCAT | 516 |
| JAG2-R | TCCTCTCCTTACAGCCAAGA |  |
| JMJD4-F | CCCGCCTGTATGTACTCTTTC | 585 |
| JMJD4-R | CGTGCTGAGCTGGATGGA |  |
| KCNA3-F | GGGGAACTGGCAAAGGGT | 556 |
| KCNA3-R | GAAGGAGGGAGGAGGCGT |  |
| KDM5C-F | TGGGCTATCAAATCACAAAT | 500 |
| KDM5C-R | TGAGGGGCTGCTAGACTCTT |  |
| KIAA0513-F | GAGAGTTGCTGGTGGCTAA | 451 |
| KIAA0513-R | TCCAAATGGAACGAGAGGT |  |
| KIF17-F | CTGGGGTGTTCAAATTGGG | 575 |
| KIF17-R | GAGTGACAGGCAGGGGAGG |  |
| KMT2C-F | AACCATGCAAACTTTATTTGAT | 428 |
| KMT2C-R | AGAGATTTCTCTCCTTTACCCC |  |
| KRT6A-F | TACCCACCTGCACTCCTCAC | 534 |
| KRT6A-R | GACAACACTCCAAACAAACC |  |
| KRTAP13-3-F | CCCCTATAGAGAGAGGAACCCA | 550 |
| KRTAP13-3-R | AAATTATTGCCCATAACAGCCA |  |
| KTN1-F | GTCCCCACCTTCTTCCCTATT | 559 |
| KTN1-R | TCTTCTGCCCTGGTTTCTTCT |  |
| LOC101928871-F | TGAACCTGCTGGGATGTGG | 539 |
| LOC101928871-R | CGTGAAAGGGAATGCGAAA |  |
| LRTOMT-F | AGTGTGATGCCCTGTATGTCT | 403 |
| LRTOMT-R | TTTCTCCTCCTCCTTTCCTTT |  |
| LYZL1-F | GGAAAAGACAAAAAACAAGAGGTAT | 543 |
| LYZL1-R | AAAAAAAAAAGAAAAGAAAAAAGAA |  |
| MACROD2-F | ACATACCAACACACTAAAGCAA | 576 |
| MACROD2-R | TGTAAGCACCATTCAGATAACC |  |
| MAGEL2-F | CAGTGGAGGTGGGGGTG | 597 |
| MAGEL2-R | CCGATGGCTTCTTGGGC |  |
| MIR205HG-F | GAGAAAAAGATCCTCAGACAATC | 395 |
| MIR205HG-R | TTTACAAGTTACAGAAAACGCAA |  |
| MUC16-F | GGCATCAGTGGTTGGGGTAT | 514 |
| MUC16-R | CGTGGTGTTGGCAAAGGTAG |  |
| MUC4-F | TCTGGATCAAATGTTACTAAGGC | 441 |
| MUC4-R | AGCAGGAACTGAAAGTTCTACCC |  |
| MYO10-F | GTTCATGTATGGGTCCTCCTCT | 580 |
| MYO10-R | AAATCTCCCTTCTGCTTCTGTC |  |
| MYO3A-F | CAGAAGTAAGCCCCAAACAGAA | 457 |
| MYO3A-R | TTGTAACCCCGGTATTTGCTCT |  |
| NEURL1B-F | TCTTCGTGGAGGTGGGC | 600 |
| NEURL1B-R | AGCCGCAACGTCAGCAT |  |
| NRAS-F | CCGACAAGTGAGAGACAGGAT | 441 |
| NRAS-R | GGGAGTAATAGGAAGGGGGAT |  |
| NRIP1-F | GGCTCTGCCTGATTTGTTG | 587 |
| NRIP1-R | GATTGCGATGGAAGGTGAT |  |
| OBSCN-F | GTGGTGGTCAGTGGGTCAGA | 410 |
| OBSCN-F | CCCACGTACCTCACCTCTTGT |  |
| OBSCN-R | GAAGTCAGTAGGCTGGGCAAT | 545 |
| OBSCN-R | ATGGTCTCCTCCACCTCCAC |  |
| OR10S1-F | TTTCCACCTGAATGATTTGA | 501 |
| OR10S1-R | ACTTCTTCTGCGACATACCC |  |
| OR51D1-F | ATCCAGGAGATCGAGTTCAA | 582 |
| OR51D1-R | TAGCAGCAAGTAGGTATTAGCC |  |
| OTOGL-F | TGAAAGGCAATGACGTTAGA | 494 |
| OTOGL-R | AAATGGCATAGCGTGAATAG |  |
| PAX7-F | AACACCGAAGACCAGCAGC | 460 |
| PAX7-R | AGGGCACCAGGCACTCACT |  |
| PCLO-F | CCCCTTACTATGTCTGCCTGTT | 420 |
| PCLO-R | TATGATGCCTGGCTTCCTC |  |
| PDE8B-F | TGGAGAATGAATTAAGCGTCTG | 432 |
| PDE8B-R | GGTGTCCTTGACCGTGAGC |  |
| PIEZO2-F | CATTGTTAATTGCAGCAGGTCA | 561 |
| PIEZO2-R | GTCATTTATTTAGGCTGAAGGAGAA |  |
| PLA2G7-F | TGAGTCCTTTGGGAAAATACAT | 422 |
| PLA2G7-R | CAGCTGATACAGAGCACATCGT |  |
| PNISR-F | CTTCCTGGAGACTCAGAACTGC | 435 |
| PNISR-R | CTCAAGGGATAGACGAACCAAT |  |
| PPP1R16B-F | TAGGGAGGTCTTCTGGGATAG | 596 |
| PPP1R16B-R | GTGGGTGACACGAATGAGAT |  |
| PPP1R3A-F | GACTTCTTTGCTGCCAGTATG | 544 |
| PPP1R3A-R | TAATCTCGTTCCCCAGTCATA |  |
| PTPN3-F | GCCCATTCCTTTCCCACAG | 425 |
| PTPN3-F | TCCAGCAGTTGGTGATAAGA |  |
| PTPN3-R | GACCGCCAGTTTCTCCCTC | 450 |
| PTPN3-R | AGGTTCCAGGGAAACATACA |  |
| RASGRF1-F | TAGCAAACTCCAACTGGTCCTT | 459 |
| RASGRF1-R | GCCTGGGTGACAGAGTGAAA |  |
| RBFOX1-F | CCTCTTCGGTTTCTTCTTGTT | 578 |
| RBFOX1-R | GAGTCACCCTCTGGATCATTT |  |
| RGSL1-F | GAAACGGTACTCAAGCAGGAA | 559 |
| RGSL1-R | ACCCAGCAAGTGACGAAAGAT |  |
| RNF213-F | GCAATGCCTTACTTAATGTGC | 415 |
| RNF213-R | CTTCGACAGAGCCTTCTTGAT |  |
| SCN1A-F | TGTGGGTACATGGTAGGTGTT | 581 |
| SCN1A-R | GTTGCTTCTCCACTAGCGTTG |  |
| SCUBE2-F | ATGTGGGAGCACGCAGAG | 489 |
| SCUBE2-R | CAGCCTGAGTGACAAGAGCA |  |
| SDK1-F | GCCACGGACTCTGACTACGA | 530 |
| SDK1-R | CCAAGCCATTGCACCAG |  |
| SF3A2-F | AAGGCGGAGGGCAAGTT | 528 |
| SF3A2-R | GGTGGACCCCAGAGGTTG |  |
| SLC10A1-F | GAGACAAAGCAGCCCAAAT | 485 |
| SLC10A1-R | TTCTTCACCGGCACAGG |  |
| SLC25A25-F | CAACCTGATGCCACCTTCT | 445 |
| SLC25A25-R | CCATTCACTCAACAAATACCG |  |
| SLC26A4-F | TGTGATGCTCCTTTCCTATTT | 588 |
| SLC26A4-R | ATGAAGCCATTTCTTTACTCG |  |
| SLC26A5-F | GGGGTTTCACTATGTTGGTC | 526 |
| SLC26A5-R | AAGATGATGTGCAGGACTGG |  |
| SLC2A13-F | CAGCAAGCTCCTACTCATCTC | 420 |
| SLC2A13-R | AACGTGCTAGTGAATAGTTCTGATA |  |
| SLC30A1-F | AGTCAGGAAGATGGCGTTCAC | 404 |
| SLC30A1-R | GGCCCCTCAGCGGTTT |  |
| SPEN-F | CTATACTGTGCCACGGGATG | 422 |
| SPEN-R | AAGCGGCGGGAAACTG |  |
| SPHKAP-F | TTGTCCAGCCCTTCTAATCC | 425 |
| SPHKAP-R | CTGTGCTCTGTGAGCCCTAA |  |
| SRGAP1-F | TTAACTTGGTTTGCTCCAGAA | 416 |
| SRGAP1-R | GGGATAGACGTGCAATTACCT |  |
| ST3GAL3-F | TGCCATCACCTCCTTTCTCA | 407 |
| ST3GAL3-R | ACTTACCTGCTCCACCCACC |  |
| STAB2-F | GGAGCCAAAGTTCAAACAAC | 400 |
| STAB2-R | TCCCTTTCCCTCCTCTTCTT |  |
| SUPT20H-F | TGTAAGTTATGCCTCTATAATTTTCC | 600 |
| SUPT20H-R | AGTTTTGTGAGCCTTCTCCATT |  |
| TAAR8-F | GTTGTGCAGCTTTGCTATGA | 571 |
| TAAR8-R | ACACCCAGCCTTGACTTACA |  |
| TBX19-F | CTTAGGCAAGAGCCAGGGTA | 490 |
| TBX19-R | AAGCCATTGGAGACAGTCGT |  |
| TEFM-F | GGGAGTCACCAATCATCAGTTC | 574 |
| TEFM-R | ACCGTATGCTCTATCCCAGAAA |  |
| TGM5-F | TGGGCACTCAGGTTCACTTT | 495 |
| TGM5-R | CTTGTGGTTGATACAGGAGGAA |  |
| TIGD2-F | CGGAATTGGTGAATCCACAG | 513 |
| TIGD2-R | CTGCTTGACCTACACCCAGA |  |
| TRIM44-F | TAACTCGGGACCAAATGAAG | 582 |
| TRIM44-R | GCCCAGGTCTATGATGACTTTA |  |
| TSKS-F | CTCTACCTCCCTGTGACTTGATG | 457 |
| TSKS-R | CAGTGGTTGGGTCCTTTGTG |  |
| TYK2-F | CACTGTCCCGGATGTAGCAG | 601 |
| TYK2-R | TGCGTCCCAGGTTCAAGAG |  |
| UPP1-F | TCAGTTACACTTCGCCCAGAG | 441 |
| UPP1-R | CAGCGGAAACAGAAACCAG |  |
| VMP1-F | CAGTGTCATCAGATTGGGAGAT | 433 |
| VMP1-R | CTAGGGAGGCTGAGACAGGA |  |
| VPS39-F | TTTGGAGGAATGAAATTGAGTG | 458 |
| VPS39--R | CTTGCTACGCCTGGAGAACA |  |
| VPS51-F | TGTGCTTTGCTTTCCTGCTC | 558 |
| VPS51-R | CCACTCGTTCCACGATCTCC |  |
| VWDE-F | TACATCCTCAGCATGGTCCTC | 433 |
| VWDE-R | ATCAGAAATTGCCTTGGGTT |  |
| WDR27-F | AATTGTTAAGGTGGTAAGAACAGAC | 565 |
| WDR27-R | AAAGCCAAGCATCAGGGT |  |
| ZC3H12D-F | CAGGCTTCCCTGACCATCTTT | 454 |
| ZC3H12D-R | CACGGCGACTTGCTTTCC |  |
| ZNF160-F | ACCAACATCTCCCTATTCCC | 598 |
| ZNF160-R | CATGATGGACAACACCCTGT |  |
| ZNF345-F | CAGCCCTTACTCGACATCAA | 572 |
| ZNF345-R | CTTCCCACAGTTCTTACATTCATA |  |
|  |  |  |
|  |  |  |
