# Supplementary material for: Inferring the progression of multifocal liver cancer from spatial and temporal genomic heterogeneity
Source: Oncotarget. 2015 Dec 11;7(3):2867–77. doi: 10.18632/oncotarget.6558 (PMC4823077; doi:10.18632/oncotarget.6558)
Supplement: Supplementary file 12 [file oncotarget-07-2867-s012.docx]

| **Supplementary Table 11. List of PCR primers used for target sequencing of *FAT4*.** | | | |
| --- | --- | --- | --- |
| **Exon** | **Forward or reverse** | **Primers** | **Amplification Length** |
| 1 | FAT4-1-1F | TCATTGCGTTTGCTTCACC | 1448 |
|  | FAT4-1-1R | ATCACCAGGCTTGCCACAG |  |
| 1 | FAT4-1-2F | CTGCTGGACGTGAATGACA | 1314 |
|  | FAT4-1-2R | GGTTACTATTGCCTGGTTGG |  |
| 1 | FAT4-1-3F | TCTACTCCCTGTTGGTTCTGG | 1385 |
|  | FAT4-1-3R | AGCCCTCTGCTCTTCTTCG |  |
| 1 | FAT4-1-4F | TGGCACTATTAGTCTGCTTGG | 1474 |
|  | FAT4-1-4R | TGTACCGATGGGAATGTTCT |  |
| 1 | FAT4-1-5F | TTAGCATTAGCCCAAACACTG | 1443 |
|  | FAT4-1-5R | CATCTTCTGGTAGCCTTCCTT |  |
| 2 | FAT4-2F | TTGCGTGGATTCCTGGTAG | 465 |
|  | FAT4-2R | TTTGGCTAAGTAACCTAAAGTGC |  |
| 3 | FAT4-3F | AAAGCCTTCCTTTGTCAAATAC | 440 |
|  | FAT4-3R | GATTAAGAGCAGCCGAAACAT |  |
| 4 | FAT4-4F | CACTGTATTTTCATAGCGGTTTT | 618 |
|  | FAT4-4R | ATTCTATTCCTCAACTTCCAACC |  |
| 5 | FAT4-5F | AACACCCTAGTAAATAGCAAGCA | 1380 |
|  | FAT4-5R | GTGGAACAAAATTCAAAAACACA |  |
| 6 | FAT4-6F | TTTTTTAATGGAGGCATTTTATT | 586 |
|  | FAT4-6R | TTTGTTTATTTTGAGACGAGTTT |  |
| 7 | FAT4-7F | CTTTTCGTGCAGAGTTACTAGGA | 532 |
|  | FAT4-7R | AGCAATGGATAACAAGAAGAGGA |  |
| 8 | FAT4-8F | ACAATATTAGAGCAAGTCCCCTG | 752 |
|  | FAT4-8R | AAGCATTTTTTCATTTCCATCAG |  |
| 9 | FAT4-9-1F | GAACGATTAGCAAGTTCTTTTCC | 1458 |
|  | FAT4-9-1R | CATTGTCATTGATGTCTGTCACA |  |
| 9 | FAT4-9-2F | CTGGTGAAATTAGAAGCGTTAG | 1438 |
|  | FAT4-9-2R | GGGAAATAGTCATCAGAGAGGA |  |
| 9 | FAT4-9-3F | CATCCTCCCTGATTTCTGACT | 1431 |
|  | FAT4-9-3R | TGTTTTCCATGACTTCTCCTT |  |
| 9 | FAT4-9-4F | CCCTTCCCATCTATAATCTCTC | 1632 |
|  | FAT4-9-4R | TCTCTACTTCAACTTCCTTGCC |  |
| 10 | FAT4-10F | TTCTAATTTCCTTATGTCAAGTAAA | 542 |
|  | FAT4-10R | CTAGTAATGACCAATGCAATGTATT |  |
| 11 | FAT4-11F | TTTATGCCAGTTTGTCAATTTTC | 554 |
|  | FAT4-11R | TTTCACGCACTAGGCTATATTCT |  |
| 12 | FAT4-12F | GCTGGAAAGGCTAATAGGGAC | 480 |
|  | FAT4-12R | AAACGCATTTGAAAGAACTGA |  |
| 13 | FAT4-13F | ATTCTACAGCATTTTATTTTCCC | 538 |
|  | FAT4-13R | CATACTTTTTTGCATTTTTTTTC |  |
| 14 | FAT4-14F | TCTTTTATGTGCGTTATTGTTCT | 600 |
|  | FAT4-14R | TATGATGGCTCTGTATGATGTTT |  |
| 15 | FAT4-15F | TTTAAGAGTAGAGTTGGGGGACA | 448 |
|  | FAT4-15R | AAGAAAAGGACAGAAATTATGGG |  |
| 16 | FAT4-16F | TATTCTATCTGCTGTGGACTGGT | 412 |
|  | FAT4-16R | AATTTTACTTCCCTTGTGACTCT |  |
| 17 | FAT4-17-1F | TGAGGTGATTGTGGAGCTTCTC | 1443 |
|  | FAT4-17-1R | ATACTTGGGTTTCTAGGGCGAG |  |
| 17 | FAT4-17-2F | ATGCAGACATCATTCAACACTA | 1414 |
|  | FAT4-17-2R | GAAACAATACACATACGCCACT |  |
|  |  |  |  |
|  |  |  |  |
