# Supplementary material for: Inferring the progression of multifocal liver cancer from spatial and temporal genomic heterogeneity
Source: Oncotarget. 2015 Dec 11;7(3):2867–77. doi: 10.18632/oncotarget.6558 (PMC4823077; doi:10.18632/oncotarget.6558)
Supplement: Supplementary file 13 [file oncotarget-07-2867-s013.docx]

| **Supplementary Table 12. Selection of anti-sequence for establishment of *FAT4* shRNA lentivirus.** | | |  |  |  |
| --- | --- | --- | --- | --- | --- |
| **No.** | **Targeted sequence** | **Knockdown efficiacy** |  | | |
| shRNA1 | GCATTGTTCTACTGGATAT | 87.00% |  | | |
| shRNA2 | CCATGGATCTCAATTCCAA | 81.01% |  | | |
| shRNA3 | GCAGCTTACCACAGCAAAT | 63.26% |  | | |
| shRNA4 | GGGACTAACAACCACGGAA | 60.10% |  | | |
| shRNA5 | GCTTCAAAGAATGCAGTTA | 57.07% |  | | |
| shRNA6 | GCTTGTAGTAACTCTCCTA | 68.82% |  | | |
|  |  |  |  | | |
|  |  |  |  |  |  |
