# Supplementary material for: Inferring the progression of multifocal liver cancer from spatial and temporal genomic heterogeneity
Source: Oncotarget. 2015 Dec 11;7(3):2867–77. doi: 10.18632/oncotarget.6558 (PMC4823077; doi:10.18632/oncotarget.6558)
Supplement: Supplementary file 14 [file oncotarget-07-2867-s014.docx]

| **Supplementary Table 13. Selection of TALE-VP64 sequence for establishment of endogenous *FAT4* overexpression in HCC cells.** | | | | |
| --- | --- | --- | --- | --- |
| **TALE NO.** | **Position of target sequence** | **Targeted sequence** | **RVDs** | **Relative Expression** |
| 1 | -49 to -32 | tgcttcaccccttccttct | NN-HD-NG-NG-HD-NI-HD-HD-HD-HD-NG-NG-HD-HD-NG-NG-HD-NG | High |
| 2 | -94 to -111 | tcagctgacggcttggtct | HD-NI-NN-HD-NG-NN-NI-HD-NN-NN-HD-NG-NG-NN-NN-NG-HD-NG | Lower |
| 3 | -149 to -131 | tgccggactggaggttct | NN-HD-HD-NN-NN-NI-HD-NG-NN-NN-NI-NN-NN-NG-NG-HD-NG | Lower |
| 4 | -203 to -186 | tgcactccgcgttcaact | NN-HD-NI-HD-NG-HD-HD-NN-HD-NN-NG-NG-HD-NI-NI-HD-NG | Medium |
| 5 | -247 to -229 | tgtcccgcggaatgccct | NN-NG-HD-HD-HD-NN-HD-NN-NN-NI-NI-NG-NN-HD-HD-HD-NG | Medium |
|  |  |  |  |  |
|  |  |  |  |  |
